# Context-Aware Biosensor Design Through Biology-Guided Machine Learning and Dynamical Modeling

**DOI:** 10.1021/acssynbio.4c00894

**Published:** 2025-06-03

**Authors:** Jonathan Tellechea-Luzardo, Hector Martin Lazaro, Christian Fernandez Perez, David Henriques, Irene Otero-Muras, Pablo Carbonell

**Affiliations:** † Institute of Industrial Control Systems and Computing (AI2), Universitat Politècnica de València (UPV), València 46022, Spain; ‡ Institute for Integrative Systems Biology I2SysBio, Universitat de Valencia-CSIC, Catedratico Agustin Escardino Benlloch 9, Paterna, Valencia 36208, Spain; § IIM-CSIC, Eduardo Cabello 6, Vigo 36208, Spain

**Keywords:** biosensor, scientific machine learning, dynamical modeling, context dependence, genetic circuit, synthetic biology

## Abstract

Addressing the challenge of achieving a global circular bioeconomy requires efficient and robust bio-based processes operating at different scales. These processes should also be competitive replacements for the production of chemicals currently obtained from fossil resources, as well as for the production of new-to-nature compounds. To that end, genetic circuits can be used to control cellular behavior and are instrumental in developing efficient cell factories. Whole-cell biosensors harbor circuits that can be based on allosteric transcription factors (TFs) to detect and elicit a response depending on the target molecule concentrations. By modifying regulatory elements and testing various genetic components, the responsive behavior of genetic biosensors can be finely tuned and engineered. While previous models have described and characterized the behavior of naringenin biosensors, additional data and resources are required to predict their dynamic response and performance in different contexts, such as under various gene expression regulatory elements, media, carbon sources, or media supplements. Tuning these conditions is pivotal in optimizing biosensor design for applications operating in varying conditions, such as fermentation processes. In this study, we assembled a library of FdeR biosensors, characterized their performance under different conditions, and developed a mechanistic model to describe their dynamic behavior under reference conditions, which guided a machine learning-based predictive model that accounts for context-dependent dynamic parameters. Such a Design-Build-Test-Learn (DBTL) pipeline allowed us to determine optimal condition combinations for the desired biosensor specifications, both for automated screening and dynamic regulation. The findings of this work contribute to a deeper understanding of whole-cell biosensors and their potential for precise measurement, screening, and dynamic regulation of engineered production pathways for valuable molecules.

## Graphical abstract

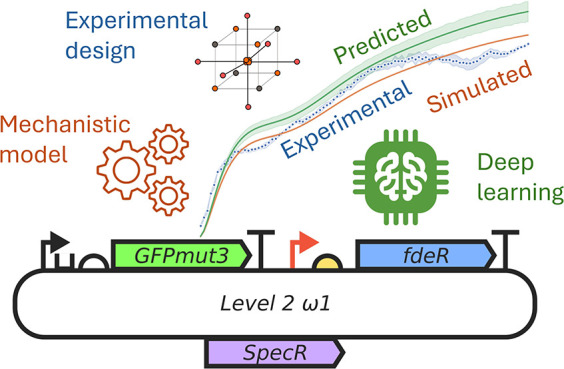

## Introduction

Achieving a global sustainable bioeconomy will require a boost in bio-based processes to become a competitive replacement for the production of chemicals that are currently obtained from fossil resources, as well as in the production of new-to-nature compounds. Addressing this challenge depends largely on improving current approaches to biomanufacturing, which are prone to failure when transferring them from the lab bench to industrial scaling-up, by making those processes more robust and efficient. Several initial steps in that direction have recently been taken through the establishment of automated biofoundries,^1^ allowing rapid prototyping and efficient application of the Design-Build-Test-Learn cycle in the early stages of process development.^2^

To ensure that the efficiency of production in terms of titer, rates, and yield is maintained at high levels when transferred to industry, next-generation biomanufacturing and precision fermentation will require the use of tightly regulated production routes so that their performance remains robust when moved from lab-controlled environments to harsh and variable conditions, such as those found in fermenters.^3^ To achieve this, synthetic biology uses various types of genetic circuits to control cell behavior. Whole-cell biosensors usually employ transcription factors (TFs) to measure the concentration of a target molecule that triggers the activation or inhibition of the TF. Typically, the triggered TF binds to an upstream region of a reporter gene, whose expression becomes dependent on the concentration of the ligand.^4^ Modifying the regulatory elements of the biosensor construct (promoters, RBSs, terminators, and the TF’s operator region),^5,6^ together with testing various origins of replication,^7^ can be used to engineer the desired response behavior (e.g., dynamic and operational ranges) of the plasmid biosensor and its response to varying concentrations of the target molecule.

TF-based biosensors have been used to measure various molecules in several species for different purposes. Three of the most common applications include environmental molecule detection^8,9^ bioproduction screening,^10–12^ and dynamic regulation of metabolic pathways.^13^ Among those molecules that can be detected, naringenin is a flavonoid compound at the core of the phenylpropanoid pathway, which has pharmaceutical, nutraceutical, and cosmetic applications.^14,15^ This molecule has previously been successfully bioproduced.^16–18^ Naringenin can be sensed using TF-based biosensors. FdeR (initially found inHerbaspirillum seropedicae) is a transcriptional regulator of the LysR family, acting as a gene expression activator when naringenin is present in the environment.^19^ FdeR has been engineered multiple times as a naringenin biosensor in Escherichia coli^6,7^ and Saccharomyces cerevisiae^20,21^ tuning the biosensor’s performance indicators. In order to characterize the naringenin biosensor, a model has been previously built describing the behavior of those biosensors.^7^ However, such a model was focused on characterizing the steady-state response of the biosensor under standard conditions, and more data and resources are needed to model their dynamic behavior under different contexts, including multiple combinations of gene expression regulatory elements, and in different media and carbon sources. These conditions may be essential in determining the best biosensor set of variables for specific purposes (e.g., screening, precise measurement, and dynamic regulation). For these reasons, in this work, we describe the assembly of an FdeR biosensor library, characterize it under different conditions, and develop a mechanistic-guided machine learning computational model that describes its behavior and predicts library combinations for designated biosensor behavior specifications.

## Results and Discussion

Biosensors, notably those based on allosteric transcription factors, are increasingly used for dynamic pathway regulation in metabolic engineering. However, selecting and assembling the appropriate biosensor DNA parts leading to the desired dynamic response, depending on the application—such as those used for screening or dynamic regulation—is a challenging task not always easily addressed because the biosensor’s behavior depends on the environmental conditions where it will be operating, often leading to unanticipated effects. Here, we investigated whether such a design process can be rationalized using an approach that leverages both the mechanistic knowledge of biosensor dynamics and predictive approaches based on machine learning.

We started with the prior knowledge available on the mechanisms that activate the biosensor response under controlled conditions. Based on this knowledge, we first built a library of genetic parts and selected a number of relevant environmental factors that might significantly impact the biosensor’s dynamic response. Then, an optimal experimental design was planned in order to perform those experiments that could provide the model with the most informative data about the biosensor response under different conditions. The combinations obtained as a result of the experimental design were assembled in a library, and their responses were quantified. Next, the dynamic responses were sampled multiple times using bagging to calibrate an ensemble of mechanistic models by optimally fitting their parameters. These parameter sets were subsequently used to build a predictive ensemble of models using deep learning (the overall workflow is shown in Figure S1).

The parameters defined the context in which the biosensor operates, including promoter strength, media conditions, and RBS tuning. In the case of transcriptional regulation, promoter strengths were optimally determined for each promoter type, independently of the other factors defining the context. We assumed that the medium primarily determines the RNA and protein production rates in the cell, as well as the mRNA degradation rate. The reason for that choice is that these two rates are known to vary according to the metabolic context of the cell.^22^ Finally, the growth and RBS strength were modeled as context-dependent for every factor, as we obtained poor results in terms of fitting when considered solely as medium-dependent.

### Construction and Functional Characterization of the Naringenin Biosensor Library

#### Genetic Library and Biosensor Construction

Characterizing and fine-tuning the dynamic behavior of transcription factor-based biosensors are essential for pathway dynamic regulation and precision biomanufacturing. Here, we applied our strategy for context-based optimization through a Design-Build-Test-Learn (DBTL) pipeline^2^ to characterize a naringenin biosensor’s dynamic response, a flavonoid with a large scope of industrial and commercial applications. In order to perform the functional characterization of the naringenin biosensor considered in this study, we built and engineered a combinatorial library of biosensors in the Escherichia coli chassis that consisted of the assembly of two modules, as described in the Methods section and shown in Figure 1. The first module, a naringenin-responsive transcription factor FdeR, was combinatorially built from a collection of DNA parts consisting of 4 promoters and 5 ribosome binding sites (RBS) of different strengths, as detailed in Table S1. The resulting FdeR modules were then assembled along with a second module containing the FdeR operator region and a GFP reporter gene. In this way, we were able to successfully build 17 constructs of the combinatorial library, as detailed in Table S2. The inability to assemble some constructs may be due to a lack of compatibility between high-strength combinations of promoters and RBS.

**Figure 1:**
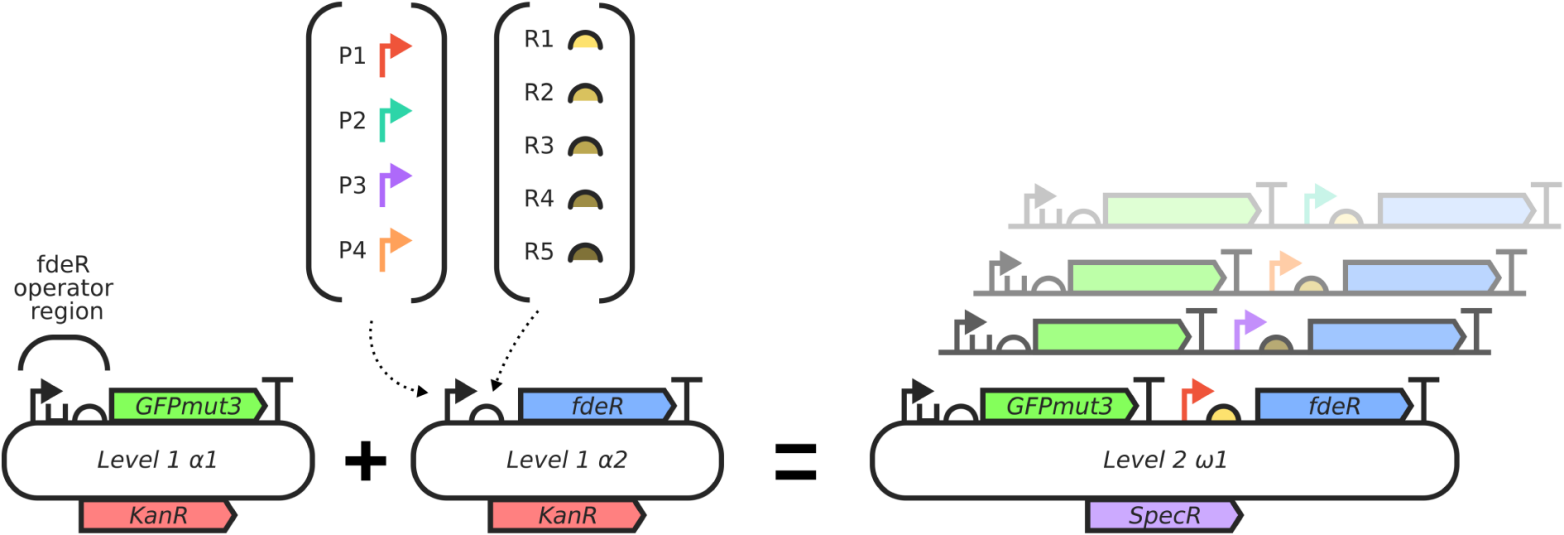
Scheme of the cloning process and final biosensor circuit.

The purpose of building the library was to study how the biosensor behavior might vary as a function of the selection of different transcriptional and translational regulators, and how this would eventually be affected by environmental conditions. Obtaining such valuable prior knowledge was a first step toward constructing a mechanistic model based on the observed behavior.

#### Biosensor Dynamic Response Characterization at Standard Conditions

The 17 available circuits were tested under the same conditions (M9, 0.4% glucose, 400 μM naringenin). The working reference concentration of 400 μM naringenin was determined based on the dose-response of the biosensor (see Figure S2). After 7 h, circuits with promoters P1 and P3 produced the highest fluorescence values, as shown in Figure 2. Meanwhile, promoter P4 produced the lowest outputs. For the model construction and characterization, construct fdeO-GFP-T1-P1-R4-fdeR-T1 was selected as the reference circuit for its representative behavior within the operating range compared to the other biosensors.

**Figure 2:**
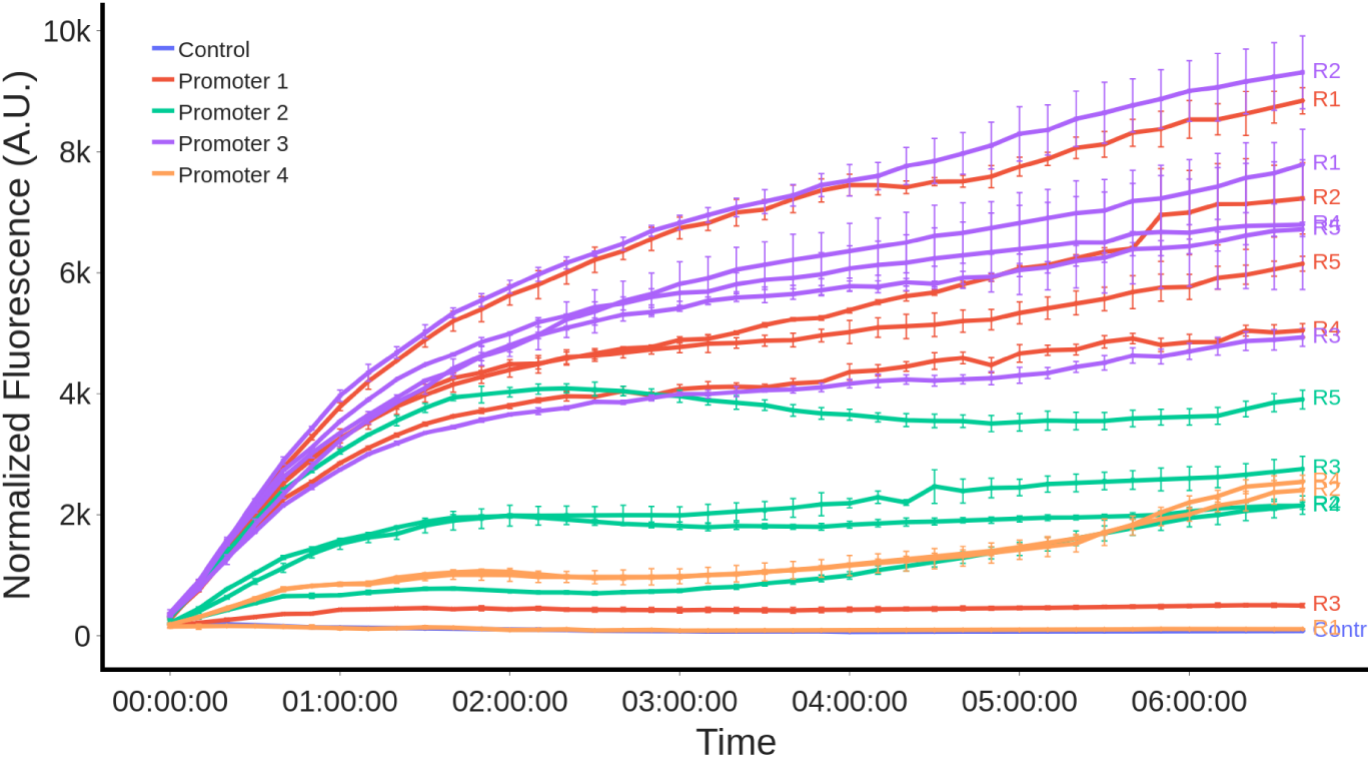
Normalized fluorescence profile of the 17 Level-2 biosensor constructs. The experiments used 400 μM naringenin induction, M9 as growth media, and glucose (0.4%) as carbon source. The line color determines the promoter used. The RBS used in each case is shown at the end of each fluorescence curve.

#### Effect of Environmental Context on the Dynamics

It has been extensively observed how the environmental context crucially affects the outcome of genetic circuits.^23^ We aim to incorporate the effects of environmental context conditions into the model for the biosensor. To initially demonstrate significant environmental effects on the dynamics, the reference construct was grown in 16 possible combinations of media and supplements (see Methods Section). As shown in Figure 3, the biosensor exhibited significant contextual dependencies. It is observed that M0 (M9 medium) produces the highest results of normalized fluorescence, followed by M2 (SOB). The supplements leading to the highest signal are S1 (glycerol) and especially S2 (sodium acetate), whereas S0 (glucose) produces the lowest normalized fluorescence outputs in all four media.

**Figure 3:**
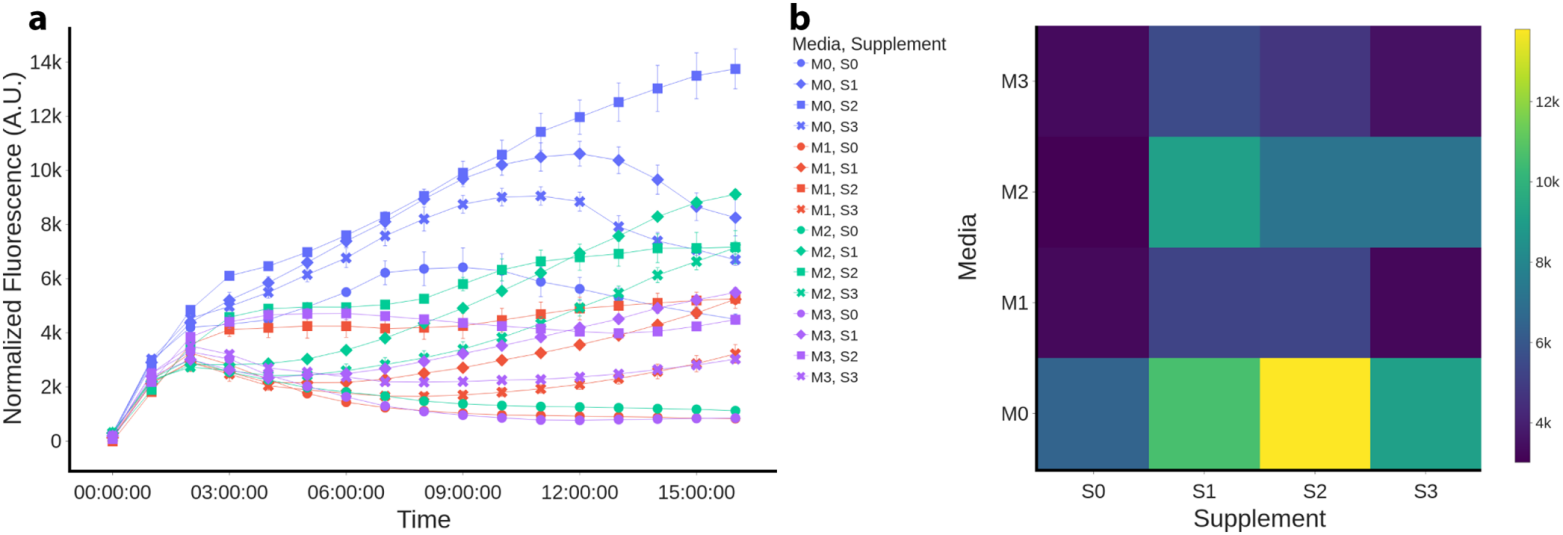
Media and supplements have a significant effect on the dynamics of the reference construct. (a) Normalized fluorescence over 16-h experiments, comparing media (color) and supplements (shape). (b) Normalized fluorescence heat map (media vs supplement) of the mean of the maximum values in each experiment.

### Development of a Biology-Guided Machine Learning Predictive Model for the Biosensor Dynamic Response

#### Initial Experimental Design

The observed biosensor dynamic responses for genetic and context variations showed, as expected, complex dependencies between the factors, i.e., promoters, RBSs, media, and supplements. In order to systematically explore more in-depth interactions among these factors across the design space, we selected an initial set of 32 experiments through *D*-optimal design of experiments (DoE) (see Methods Section and Table S3). An initial analysis of the responses from the 32 experiments was then carried out by studying the six pairwise combinations of the four factors (Figures 4 and 5). In the results from the experimental set, promoter P3 consistently exhibits higher fluorescence values across various RBSs, media, and supplements than other promoters. The median fluorescence in experiments using P3 was notably high, indicating that promoter P3 is more effective in driving GFP expression than the other promoters tested. P4 also showed relatively high fluorescence in some experiments. P1 and P2 generally exhibited lower fluorescence levels compared to P3 and P4. Regarding RBSs , the combinations containing R4 or R5 resulted in higher fluorescence levels in several experiments. However, in general terms, the selection of RBSs does not appear to have an apparent positive effect. Among the tested RBSs, RBS3 consistently performs poorly in all combinations with different promoters, media, and supplements. This suggests that RBS3 may be less effective in driving GFP fluorescence expression than the other RBSs. With respect to media, the M0 medium positively impacted some of its interactions with the other factors. When combined with different promoters, RBSs, and supplements, medium M0 tends to produce higher fluorescence values than other media. M0 medium may provide favorable conditions for GFP fluorescence expression or detection. Similarly, Supplement S2 positively affects all the combinations in which it appeared. When combined with different promoters, RBSs, and media, Supplement S2 consistently enhanced GFP fluorescence expression, leading to higher fluorescence values. This indicates that the S2 supplement may be a good addition to the growth media to increase fluorescence output, especially when used with M0.

**Figure 4:**
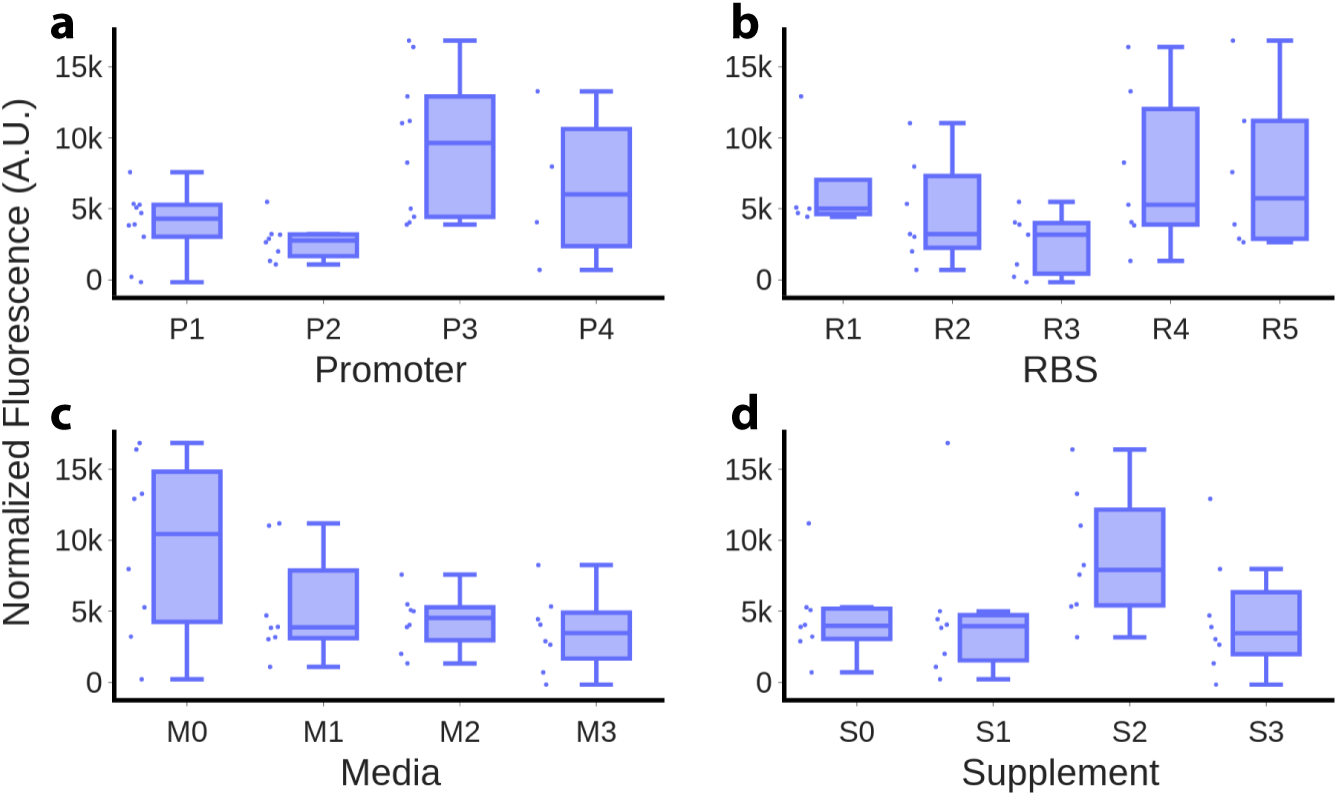
Isolated effect of the four DoE variables on the normalized fluorescence of the naringenin biosensor. (a) Effect of promoter selection. (b) Effect of ribosome binding site (RBS) selection. (c) Effect of media selection. (d) Effect of supplement selection.

**Figure 5:**
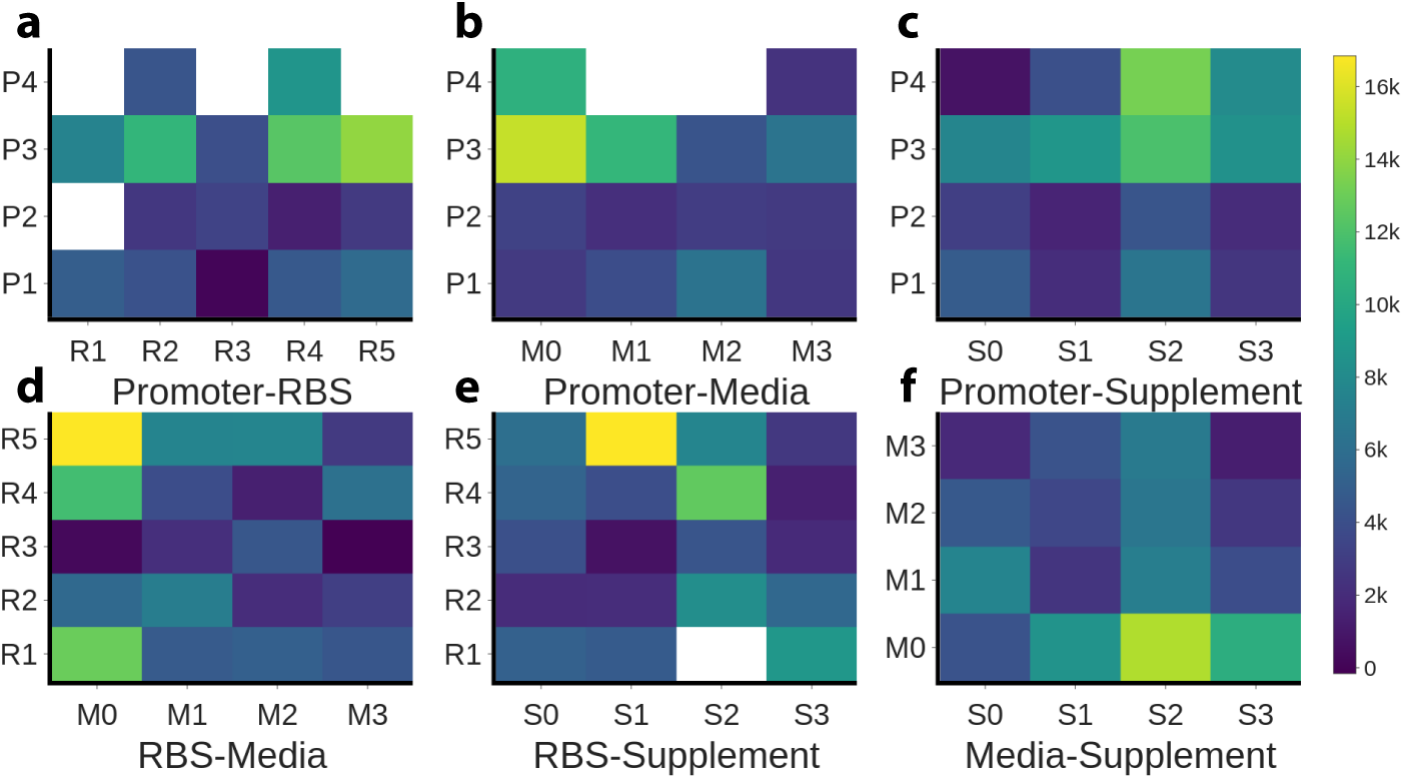
Heat map pairwise comparisons of the four factor combinations. The color intensity describes the normalized fluorescence value. In the case where a combination appears in more than one DoE experiment, the mean was calculated. White squares indicate combinations that do not appear in the 32 DoE experiments. (a) Promoter vs RBS. (b) Promoter vs media. (c) Promoter vs supplement. (d) RBS vs media. (e) RBS vs supplement. (f) Media vs supplement.

#### Mechanistic Modeling of the Biosensor Response under Nominal Conditions

A mechanistic model using ordinary differential equations was developed, based on prior mechanistic knowledge of the system, to capture the biosensor’s dynamics. The parameters and the assumed context dependency are shown in Table 1.

**Table 1:** Parameter Definitions and Variability in the Model

Parameter	Definition	Variability
*L*_1_	Asymptotic number of cells	Context-dependent
*a*_1_	Logistic growth rate	Context-dependent
*t*_1_	Logistic midpoint	Context-dependent
*k*_1_	First logistic gain coefficient	Global
*k*_2_	Second logistic time coefficient	Global
K_Pk_	Promoter-specific transcriptional strength	Promoter-dependent
*K*_lysis, *M*i_	Proteolysis rate constant for medium *M*_i_	Medium-dependent
*k*_deg_	Base degradation constant for mRNA	Global
*K*_mRNAdeg, *M*i_	mRNA degradation rate constant for medium *M*_0_	Medium-dependent
*K*_mRNAp_, _*M*i_	mRNA production rate constant for medium *M*_i_	Medium-dependent
*K*_P, *M*i_	Protein production rate constant for medium *M*_i_	Medium-dependent
vmax_PROM_	Maximum promoter activity	Global
RBS_Mi, Sj, Pk, Rm_	RBS strength depending on combinations of factors	Context-dependent
*k*_deg, GFP_	Degradation rate for GFP	Global
*k*_FDER_	Translation rate for FDER	Global
*K*_NFDER_	Michaelis constant for FDER	Global

Initially, the bacterial growth was captured by using a double logistic function with parameters *L*_1_, *a*_1_, *a*_2_, *b*_1_, and *b*_2_ as follows:

1 $$N=\frac{L_{1}}{1+e^{-a_{1}(t-t_{1})}}+\frac{k_{1}}{1+e^{-(k_{1}a_{1})\cdot (t-k_{2}t_{1})}}$$

Based on the previous expression, the dilution rate was estimated as μ = 1/*N dN*/*dt*, which accounts for the dilution effect by population growth as the number of cells *N* increases.

To define the biosensor model, mRNA production and degradation rates (*K*_mRNAp_, *M*_i_, *K*_mRNAdeg_, *M*_i_), as well as protein production and proteolysis rates ( $K_{P,M_{i}}$ , *K*_lysis_), are considered specific to each type of medium, *M*_*i*_. Regarding the transcriptional and translational regulation of the biosensor, a promoter strength parameter $K_{P_{k}}$ is defined for each constitutive promoter, *P*_k_, whereas the RBS strength is made specific for each context, i.e., medium, substrate, promoter, and RBS sequence (*M*_i_, *S*_j_, *P*_k_, *R*_m_). The reason for that choice is that these two rates are known to vary according to the metabolic context of the cell^22^ Finally, the growth and RBS strength were modeled as context-dependent for every factor, as we obtained poor results in terms of fitting when considered solely as medium-dependent. In this way, transcription and translation of the transcription factor FdeR are modeled as follows:

2 $$\frac{d\left(\mathrm{mRNA}\right)}{dt}=\mathrm{vmaxPROM}\cdot K_{\mathrm{mRNAp},M_{i}}-\mu \cdot \mathrm{mRNA}-K_{\mathrm{mRNAdeg}}\cdot \mathrm{mRNA}$$

3 $$\frac{d\left(\mathrm{FDER}\right)}{dt}=k_{\mathrm{FDER}}\cdot R\cdot \frac{{\mathrm{RBS}}_{M_{i},S_{j},P_{k},R_{m}}\cdot \mathrm{mRNA}}{\mathrm{mRNA}+K_{\mathrm{NFDER}}}-k_{\deg,\mathrm{FDER}}\cdot \mathrm{FDER}\cdot K_{\mathrm{lysis},M_{i}}-\mu \cdot \mathrm{FDER}$$

Finally, the measured normalized fluorescence of the reporter GFP/OD can be related to the activation of the transcription factor FdeR by the naringenin presence through the following equation:

4 $$\frac{d\left(\mathrm{GFP}/\mathrm{OD}\right)}{dt}=vmax_{\mathrm{NAR}}\cdot K_{P,M_{i}}\cdot R\cdot \frac{{\mathrm{FDER}}^{2}}{{\mathrm{FDER}}^{2}+0.5^{2}}-k_{\deg,\mathrm{GFP}}\cdot \mathrm{GFP}/\mathrm{OD}\cdot K_{\mathrm{lysis},M_{i}}-\mu \cdot \mathrm{GFP}/\mathrm{OD}$$

where vmax_NAR_ is the maximum rate of GFP production, defining an upper limit on GFP synthesis under optimal conditions. $K_{P,M_{i}}$ is the rate of protein synthesis, a term modified by the medium conditions, influencing the speed at which GFP is produced. *R* denotes ribosomal resources available for protein synthesis, as *R* represents ribosomes in this context, directly impacting GFP production based on ribosomal availability. The Hill equation introduces a saturation effect on GFP/OD production, where feed-dependent rate (FDER) models nutrient availability, and the constant 0.5 acts as the half-saturation value, reducing the production rate when FDER is low. This value is fixed because no data were collected on the concentrations of FDER. *k*_deg_, GFP is the rate constant for the degradation of GFP, representing how quickly GFP is broken down within the cell. *K*_lysis_, *M*_i_ refers to proteolytic activity and scales the GFP degradation according to the medium conditions. μ is the specific growth rate, causing dilution of GFP concentration due to cell growth, modeled by the term μ· GFP/OD.

Available ribosomes, denoted by *R*, are described by

$$R=A\cdot \left(1-R_{\mathrm{basal}}\right)+\left(1-A\right)\cdot R_{\mathrm{basal}}+A\cdot R_{\mathrm{basal}}$$

To describe this, *A* is a coefficient adjusting the ribosomal response according to cell growth, and *R*_basal_ represents the baseline ribosome level when no external stimuli are applied, serving as the steady-state ribosome concentration under default conditions.

The model calibration is formulated as a Least Square Optimization Problem, and solved by a Global Optimization algorithm (a detailed description is provided in the Methods Section). Fits obtained for the experiments are provided in Figures S3–S5. Figure 6 compares the performance obtained in the OD and GFP/OD models based on fitted and predicted values for one experiment and for the full experimental set.

**Figure 6:**
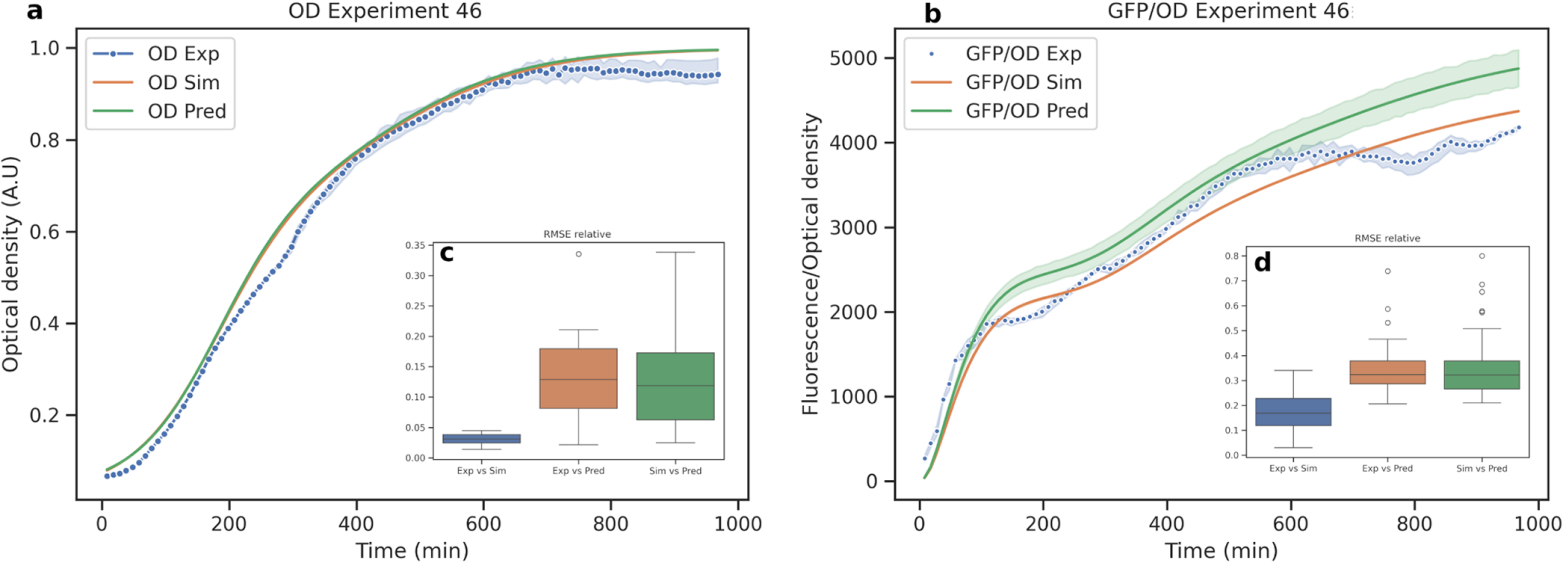
Fitting and prediction results obtained in the cross-validation for Experiment 46. (a) Experimental growth curves (OD) vs simulated and predicted response. (b) Experimental reporter curve (GFP/OD) vs simulated and predicted responses in the ensemble modeling obtained by bagging. (c) Comparison of RMSE performance for OD curves vs simulated and predicted responses. (d) Comparison of RMSE performance for GFP/OD vs simulated and predicted responses.

#### Predictive Modeling and Validation of the Biosensor under Variable Conditions

Based on the previous observations, we assumed different levels of variation in the parameters of the biosensor mechanistic dynamic model depending on the context, as shown in Table 1, because of design choices or due to the varying environments found in different processes, from microplates to fermentor volumes. Starting from such mechanistic modeling assumptions, our goal was to establish a machine-learning workflow to learn from the parametric sets fitted through the model calibration in order to predict the biosensor’s dynamic response. Two different strategies were used to obtain the predictive model: one for the cell biomass (OD) and the other for the biosensor reporter (GFP/OD). In the case of the OD, our objective was to test whether the mechanistic model worked correctly. To this end, a model was trained and validated using the experimental data from the optimally designed initial set of 32 experiments (R1). An additional set of 16 experiments was kept for validation. Two parameters of the double logistic model were predicted, *a*_1_ and *t*_1_. Table 2 shows the coefficient of determination *R* ^2^ score obtained in the leave-one-out cross-validation in each training round for each of the parameters. Notably, the performance of the fitting drastically increased from the first round of 32 experiments to the second round of 48 experiments (R2), showing how augmented designs can substantially increase the overall performance.

**Table 2:** *R* ^2^ Score Obtained for the Parameters of the Growth Model

Parameter	Round 1	Round 2
a1	0.4375	0.6554
t1	0.3817	0.4837

For our purposes, rather than focusing on the accuracy of the predictions with respect to the biomass-fitted parameters, a more decisive parameter for evaluating the goodness of the predictions was the agreement between the time-course measurements of cell population growth and those obtained by the model based on the predicted parameters. Tables 3 and 4 show the root mean squared error (RMSE) mean and standard deviation obtained during training and subsequent validation. The tables include the RMSEs obtained by comparing the experimental biosensor responses with the simulated response from the calibrated model, the simulated vs the predicted response from the machine learning model, and the experimental vs the simulated response.

**Table 3:** Mean RMSE in Biomass Growth Prediction for the Initial 32 Experiments (Round 1)

	Exp vs Sim	Sim vs Pred	Exp vs Pred
Round 1	3.57%	8.68%	9.48%
Validation Round 1	3.14%	12.27%	13.06%

**Table 4:** RMSE Standard Deviation in Biomass Growth Prediction for the Initial 32 Experiments (Round 1)

	Exp vs Sim	Sim vs Pred	Exp vs Pred
Round 1	0.018	0.064	0.056
Validation Round 1	0.001	0.055	0.053

As previously mentioned, to improve the performance of the model, it was decided to increase the number of training experiments from 32 to 48 (R2). As in the previous case, Tables 5 and 6 show the mean and standard deviation of RMSE obtained during the training and validation of the model. Figure 6a shows, for instance, the simulated responses from the fitting and predicted responses that were obtained for experiment 46, compared with the experimental data in a leave-one-out cross-validation.

**Table 5:** Mean RMSE in Biomass Growth Prediction for the Set of 48 Experiments (Round 2)

	Exp vs Sim	Sim vs Pred	Exp vs Pred
Round 2	4.01%	7.33%	8.02%
Validation Round 2	3.13%	12.68%	13.62%

**Table 6:** RMSE Standard Deviation in Biomass Growth Prediction for the Set of 48 Experiments (Round 2)

	Exp vs Sim	Sim vs Pred	Exp vs Pred
Round 2	0.022	0.050	0.044
Validation Round 2	0.009	0.080	0.074

For the GFP/OD to reduce variance and avoid overfitting, we followed a bagging sampling strategy, where the fitting of the model to the experimental data was repeated multiple times with a total of 300 iterations, obtaining multiple values for both the global and context-dependent parameters defined in Table 1. The resulting set of parameters for the ensemble of fitted models was then used to train a deep learning model with the ability to predict the parameters for any given experimental and genetic combinations. The performance obtained in the predictive model was of *Q* ^2^ = 0.91 using a leave-one-out cross-validation. Regarding the RMSE, the obtained relative error in the predictive models from the leave-one-out cross-validation was 0.65%. The predictive model learned by the neural network based on the bagging strategy showed a performance that was close to the one obtained by fitting a mechanistic model. More interestingly, the predictive model of the biosensor dynamics showed, on average, almost no degradation in performance for the cross-validation (see Methods Section), suggesting that the machine learning algorithm was able to learn and successfully infer the context effects due to both environmental and genetic variations.

#### Selection of a Tunable Biosensor for Desired Specifications

Once our models were experimentally validated, we used them to select the best biosensor design under desired specifications. More precisely, we considered two design problems: (a) optimal biosensor design for high-throughput screening, i.e., in the case of a biosensor that is used for screening and selection, high reporter gain should be considered the desired behavior; (b) optimal biosensor design for dynamic regulation, i.e., a biosensor that is part of a feedback loop for dynamic regulation and, therefore, a quick response time is required to ensure that the regulated pathway is kept under the desired operating constraints. In order to perform this evaluation, the dynamic responses of each model were simulated, and their steady-state gain was compared with the observed one. As Table 7 shows, 8 out of 10 top high-gain constructs in the experimental test set were successfully predicted by the models (*p*-value ≤0.05 according to Kendall’s rank correlation test). Predicted and measured (observed) relative values for GFP/OD with respect to the maximum measured gain across all experiments are given, along with their rank according to the experimental values. Interestingly, the predictive model suggested that M0 medium and promoters P1 and P3 are design choices favoring the highest gains in the biosensors, while factors such as substrate and RBS do not seem to have a defined impact. Therefore, the predictions provided additional design clues for the biosensor, complementing previous observations from the experiments.

**Table 7:** Predicted Top 10 High-Gain Biosensors, Experimentally Observed vs Predicted

Predicted value (%)	Observed value (%)	Rank	Experiment	Media	Substrate	Promoter	RBS
100	100	1	E25	M0	S2	P3	R4
95.83	87.03	3	E34	M0	S2	P1	R1
86.59	79.66	5	E28	M0	S1	P3	R5
75.47	54.12	7	E33	M0	S1	P1	R1
71.20	44.07	11	E20	M0	S3	P3	R1
69.28	63.03	6	E41	M0	S3	P1	R5
65.22	80.55	4	E31	M0	S2	P4	R4
60.31	42.49	12	E36	M0	S1	P1	R2
59.37	98.32	2	E48	M0	S3	P2	R5
53.70	40.06	10	E37	M0	S3	P1	R2

Next, we tested the ability of the predictive models to select a biosensor with a desired dynamic response specification. To that end, we measured the half-times of the responses in the experimental test set and compared their ranking with those obtained from the predictive model, finding again that 8 out of the top 10 fastest responses in the combinatorial library were successfully predicted (*p*-value ≤0.05 according to Kendall’s rank correlation test), as shown in Table 8. Similar to the case of high-gain biosensors, predicted and observed relative time responses are shown, along with their experimental ranked positions. Based on the predictive model results, M3 is the medium that enables the fastest response, whereas other design choices, like genetic modification, did not show a well-defined trend.

**Table 8:** Predicted Top Fast-Response Biosensors, Experimentally Observed vs Predicted

Predicted value (s ×10^3^)	Observed value (s ×10^3^)	Rank	Experiment	Media	Substrate	Promoter	RBS
0.48	0.48	1	E17	M3	S0	P2	R5
0.48	0.48	1	E29	M3	S0	P4	R2
0.48	0.48	1	E45	M3	S0	P2	R3
1.08	1.68	2	E18	M3	S3	P2	R5
1.08	0.48	1	E52	M3	S0	P3	R2
1.08	1.68	3	E43	M3	S3	P2	R2
1.08	8.28	14	E64	M3	S3	P4	R4
1.68	0.48	1	E54	M3	S3	P3	R2
1.68	1.68	3	E53	M3	S1	P3	R2
1.68	5.28	9	E50	M3	S1	P2	R5

## Conclusions

Transcription factor-based biosensors are increasingly becoming an essential part of the design toolkit of bio-based biomanufacturing pathways due to their ability to provide single-cell screening and dynamic regulation capabilities. Therefore, more in-depth knowledge and predictive capabilities for the biosensors’ behavior under different conditions are currently sought. In this study, we aimed to characterize how a transcription factor-based biosensor’s dynamic response can be modulated by two different types of factors: (a) intrinsic effects arising from the designed architecture of the genetic circuit of the biosensor, which focused on transcriptional regulation based on promoter selection and translational regulation from the RBS, and (b) extrinsic effects, which in our case focused on exchanging the growth media and the supplemented substrate as the main carbon sources. Considering the effects of these sets of factors can provide us with valuable knowledge about their impact on the design of the biosensor, as well as the effect of external perturbations due to different contexts, such as growth in well plates vs growth in bioreactors. This is an important aspect that needs to be taken into account, especially if the biosensors will be used as part of a pathway dynamic regulation strategy. Even though, to date, applications of biosensors do not model their dynamics in detail, we believe that as more biomanufacturing applications requiring dynamic regulation are implemented, modeling such behavior will become an essential part of the pipeline for bioproduction pathway design.

As we have shown in this study, mechanistic models, even if useful to characterize the biosensor under controlled conditions, might not be able to capture some of the context-related complexity and uncertainties of the biosensor behavior under varying operating environments. Therefore, our strategy has been to augment those models through a customized machine-learning approach that is guided by the mechanistic model arising from biological principles. The novelty of the procedure is 2-fold. Besides combining mechanistic and machine learning-based approaches for biosensor modeling, to overcome the limitations of the size of the data set, we proposed a bagging strategy to generate an augmented training set. In that way, we started with the definition of a mechanistic dynamic model based on the observed behavior of the biosensor, whose parameters were fitted under different combinations of intrinsic and extrinsic factors chosen through optimal experimental design. An ensemble of calibrated parameters, obtained by multiple sampling of the experimental data, was used to train and cross-validate a predictive model using deep learning, fulfilling two rounds of the Design-Build-Test-Learn (DBTL) cycle. The resulting predictive model was then experimentally validated for two application cases: the selection of the best biosensor design for screening, and for pathway dynamic regulation. In that way, we combined a biology-guided mechanistic and predictive modeling strategy. Combining both approaches synergistically allowed us to achieve an improved selection of biosensors with designated properties and dynamic specifications.

## Methods

### Media Formulations

LB (Tryptone 10 g/L, NaCl 10 g/L, Yeast extract 5 g/L) was used as a general-purpose transformation and propagation medium. M9 (sodium phosphate-dibasic 6 g/L, potassium phosphate-monobasic 3 g/L, NaCl 0.5 g/L, ammonium chloride 1 g/L, casamino acids 2 g/L, magnesium sulfate 2 mM, and calcium chloride 0.1 mM) was used for the initial characterization approach and baseline biosensor parameter determination, using 0.4% glucose as the carbon source. Alongside LB and M9, SOB (tryptone 20 g/L, Yeast extract 5 g/L, NaCl 0.5 g/L, potassium chloride 0.186 g/L, and magnesium chloride 0.96 g/L) and sLB (Condalab’s—CAT1163 proprietary peptone mixture, Yeast extract, and salts) were used during the DoE phase of experiments to further characterize the biosensors under different media conditions. For clarity, the media were labeled as M0 (M9), M1 (LB), M2 (SOB), and M3 (sLB). Similarly, the supplements were designated as S0 (glucose), S1 (glycerol), S2 (sodium acetate), and S3 (sorbitol). All sugar supplements were used at a final concentration of 0.4%. The different antibiotics used in this study were used at standard E. coli growth concentrations.

### Strains, Plasmids, and Sequences

Lucigen’s E-cloni 10G cells were used for plasmid assembly and propagation. This strain was also used to obtain fluorescence and biosensor performance data. All the part sequences can be found in Table S1. All the plasmids used and built in this study can be found in Table S2.

### Biosensor Library Assembly

Golden Braid^24^ assembly (a Golden Gate^25^ standard) was used to build the biosensor circuits. A detailed explanation of the vectors, regulatory parts, and the assembly method can be found in Boada et al. (2019).^26^ The FdeR operator and the FdeR gene had to be domesticated into the cloning method using the pBiosensor358 from ref 7. Both parts were PCR-amplified using primers containing the Golden Braid adapters. The FdeR gene amplicon was amplified using the CDS adapters. The FdeR operator PCR product contained the left promoter adapter and the right RBS adapter (since the fdeO contains both the native promoter and RBS). Afterward, the domesticated DNA parts in pL0C were assembled in pAlpha vectors with the specific promoters, RBS, and terminators to build single transcriptional units. Pairs of transcriptional units were assembled in pOmega vectors to build the final biosensor circuits (containing GFP under the control of the fdeO operator region and the FdeR TF downstream of different promoters and RBSs).

pBiosensor358 and the Golden Braid vectors, along with level-0 parts, were kindly provided by the BioRetroSynth Lab (INRAE, Paris) and the Synthetic Biology and Biosystems Control Lab (UPV, Valencia), respectively.

### Biosensor Characterization Experiments

10G cells carrying the complete biosensor constructs were grown overnight in LB/spectinomycin. OD was normalized in the specific experiment media the following day to approximately 0.05. Cells were then induced with different concentrations of naringenin dissolved in 100% ethanol. The GFP fluorescence emission experiments were carried out in a BioTek Cytation 3 plate reader with an excitation wavelength of 488 nm and an emission wavelength of 530 nm. Measurements were taken every 10 min.

### Optimal Experimental Design

The experimental design was based on a *D*-optimal design strategy,^27^ which seeks to maximize the information from the experiments by minimizing the covariance of the parameter estimates for the assumed model. The design space consisted of 2 genetic regulatory factors, i.e., promoter and ribosome binding site (RBS), and 2 context factors, i.e., growth media and carbon source. The levels of the factors were considered categorical with no prior information; therefore, the assumed model for the design was defined as a linear combination of inputs obtained by one-hot encoding of the factors. The size of the initial library was chosen to consist of 32 experiments, followed by an augmented design based on a second batch of 32 additional experiments.

### Model Fitting

The calibration of the mechanistic model was formulated as minimizing a cost function defining the goodness of the fit of the model with respect to the experimental data set, and subject to the biosensor dynamics. Considering Least Squares Estimation, with the cost function defined by the least-squares error between the data and the model output, the optimization problem reads as follows:

5 $$\underset{k}{\min}\int_{0}^{t}{\left(y^{m}\left(t\right)-y\left(k,t\right)\right)}^{T}W(\left(y^{m}\left(t\right)-y\left(k,t\right)\right)dt$$

6 $$\mathrm{subject to}:f\left(\frac{dx}{dt},x,k,v,t\right)=0$$

7 $$k^{L}\leq k\leq k^{U},k\in R_{\geq 0}$$

where *x* is the vector of state variables, *y* ^m^ represents experimental measurements of the output variables *y*, the vector *k* contains the parameters being estimated (decision variables of the optimization problem), with lower and upper bounds defined by *k* ^L^ and *k* ^U^ respectively, and *v* contains those parameters not being estimated. *W* is the matrix of weights accounting for heteroscedasticity in the error variances. This formulation corresponds to a nonlinear programming problem with differential-algebraic constraints. In order to efficiently solve this optimization problem of multimodal and nonconvex nature, we use the hybrid solver Enhanced Scatter Search (eSS) in MEIGO^28^ combining global and local optimization.

In practice, the range of parameter values *k* that align with noisy experimental data is not unique, meaning the parameters carry some degree of uncertainty. Significant parametric uncertainty can lead to limitations in the model’s ability to accurately predict scenarios beyond those used for parameter estimation.

To address model uncertainty, we employed an ensemble approach. Specifically, we used the bootstrap smoothing technique, commonly referred to as bootstrap aggregation or the bagging method in predictive modeling.^29,30^ Bagging is a well-established and effective ensemble method for model averaging that helps reduce the variability of unstable estimators or classifiers.

The underlying idea is to consider a family of models with different parameter values *k* = [*k*_1_ ... *k*_N_]^T^ compatible with the training data yy ^m^, when using the model to predict untested experimental setups. The matrix of parameter values *k* consistent with the data is obtained using *N* realizations of the data obtained by bootstrap.^31^ Each data realization has the same size as the complete data set, but it is constructed by sampling uniformly from all replicates (3 biological replicates per sampling time). Typically, the family of solutions, *k*, is then used to make *N* predictions (dynamic simulations) about a given experimental scenario. However, in this work, instead, we used the inferred parameters as the starting point for the machine learning strategy described below.

### Biology-Guided Machine Learning

A machine learning-based predictive model was built from the mechanistic model, where the training set consisted of the different combinations of factors obtained through the optimal design of experiments *X* as inputs, and the outputs were the vectors of parameters *k* obtained through the model calibration of the mechanistic model. The inputs were transformed through one-hot encoding, except for the case of promoters, where their prior quantitative values for each promoter-wise model were determined through Bayesian optimization.^32^ The resulting input matrix was standardized using StandardizedScaler from the scikit-learn Python package.^33^ The learning algorithm was based on support vector regression (SVR) from scikit-learn, with hyperparameter optimization for the kernel and regularization parameter *C*. Models were validated through leave-one-out cross-validation. Tests for the ranked experiments in the test set were evaluated through a one-tailed Kendall’s Tau rank correlation coefficient.

The predictive model for the GFP/OD was built using the Keras Python package. The neural network consists of 4 hidden layers, with dropout and batch normalization. This model was trained using the data obtained from the previous bagging approach with 300 iterations. The inputs included the context of each experiment, along with global and context-dependent parameters obtained during the fitting process. The model was validated through cross-validation, using 8 of the 64 experiments as the validation set.

## Supplementary Material



## Notes

Figure S1: visual description of the method. Figure S2: dose-response curve for the reference biosensor construct. Figure S3: experimental vs simulated vs predicted growth curves, measured as ODs, of the biosensor for the initial 32 combinatorial library. Figure S4: experimental vs simulated vs predicted growth curves, measures as ODs, of the biosensor for the second round 48 combinatorial library. Figure S5: experimental vs simulated vs predicted responses of the biosensor, measured as GFP/OD, for the 64 experiments. Table S1: DNA parts sequences for the biosensor library. Table S2: collection of plasmids for the first module. Table S3: design of experiments for the combinatorial libraries (PDF)
