# Supplementary material for: Context-Aware Biosensor Design Through Biology-Guided Machine Learning and Dynamical Modeling
Source: ACS Synth Biol. 2025 Jun 3;14(6):2094–104. doi: 10.1021/acssynbio.4c00894 (PMC12186671; doi:10.1021/acssynbio.4c00894)
Supplement: Supplementary file 1 [file sb4c00894_si_001.pdf]

Supporting Information for:

Context-aware biosensor design through biology-guided machine learning and dynamical modeling

Jonathan Tellechea-Luzardo[1], Hèctor Martín Lázaro[1], Christian Fernández Pérez[2], David Henriques[3], Irene Otero-Muras[2], Pablo Carbonell[1,2,*]

[1] Institute of Industrial Control Systems and Computing (AI2), Universitat Politécnica de València (UPV), 46022 València, Spain

[2] Institute for Integrative Systems Biology I2SysBio, Universitat de València-CSIC, Catedrático Agustín Escardino Benlloch 9, Paterna, 46980 València, Spain

[3] IIM-CSIC, Eduardo Cabello 6, 36208 Vigo, Spain

* Corresponding author: pablo.carbonell@csic.es

Contents

Table S1	DNA parts sequences for the biosensor library
Table S2	Collection of DNA parts for the first module
Table S3	Design of experiments for the combinatorial libraries
Figure S1	Visual description of the method
Figure S2	Dose-response curve for the reference biosensor construct
Figure S3	Experimental vs simulated vs predicted responses of the biosensor OD for the initial 32 combinatorial library, using leave-one-out cross validation
Figure S4	Experimental vs simulated vs predicted responses of the biosensor OD for the second round 48 combinatorial library, using leave-one-out cross validation
Figure S5	Experimental vs simulated vs predicted responses of the biosensor GFP/OD for the experiments, using leave-one-out cross validation

Supplementary Table S1. DNA parts sequences for the biosensor library.

Part Name	Part type	Part description	Additional refere	Part sequence
P1	Promoter	iGEM registry part number: BBa_J23106; Constitutive promoter from the Anderson collection.		tttacggctagctcagtcctaggtatagtgctagc
P2	Promoter	iGEM registry part number: BBa_R0061; Promoter derived from the lux operon. It is constitutive without LuxR and becomes repressible in the presence of LuxR and HSL	https://doi.org/10.	ttgacacctgtaggatcgtacaggtataat
P3	Promoter	iGEM registry part number: BBa_R0062; It is HSL inducible but shows basal expression level without LuxR/HSL		tactagacctgtaggatcgtacaggtttacgcaagaaaatggtttgttatagtcgaataaa
P4	Promoter	iGEM registry part number: BBa_J23102; Constitutive promoter from the Anderson collection.		ttgacagctagctcagtcctaggtactgtgctagc
R1	RBS	iGEM registry part number: BBa_B0030		attaaagaggagaaa
R2	RBS	iGEM registry part number: BBa_B0032		tcacacaggaaag
R3	RBS	iGEM registry part number: BBa_B0034		aaagaggagaaa
R4	RBS	iGEM registry part number: J61100		gaaagaggggacaa
R5	RBS	iGEM registry part number: J61101		aaagacaggacc
fdeO	Operator	FdeR operator region containing native promoter and RBS. FdeR binds to this region once activated by naringenin triggering the expression of the downstream gene	https://doi.org/10.	ctccgttgttgtgcttgttcttgccgaccctcggatagacgacggatggggtggtcaatgtattgatgccgtccatatcatgaatcaaaacaatccatttgatcaatatcaagctcactcttaagcttcactcatccgctgcatggccccaccagaaagggctggcgcggcaagccggcggcgcactcgcactggatgcgccgctgttgagcctggccatgacaacgcgccgatagcggccacaccccgccaggcagggtaggagacaaggag
fdeR	CDS	FdeR gene from *Herbaspirillum seropedicae* SmR1.	https://doi.org/10.	atgcgtttcaacaagctcgacctcaatcttctggtcgccctggatgcactgctcacggagatgagcatcagccgcgccgccgaaaagatccatctgagccagtcggccatgagcaatgccctggcgcggctgcgcgagtatttcgatgatgaattgctgatccaggtgggccggcgcatggagcccacgccgcgcgccgaggtgctcaaggatgcggtgcatgatgtgctgcggcgtatcgatggctccatcgcggcgctgccggccttcgtgccggccgagtccacgcgcgagtttcgcatctcggtttcggactttacgctctccgtcctcatcccccgggtgctggcgcgcgcgcacgccgagggcaagcacatccgctttgccctgatgccgcaggtgcaagacccgacccgctcgctggatcgggccgaggtggacctgctggtcttgccgcaggaattctgcacgcccgatcatcctgccgaagaggtcttccgcgaacggcatgtctgcgtggtctggcgcgacagtgcgctggcgcaaggcgagctgacgctggaacgctacatggcctcaggccatgtggtgatggtgccgcctggggccaatgcgtcgtcggtggaggcgtggatggccaggaagctgggctttgcgcgccgggtggaagtgaccagcttcagcttcgcttctgcgctggcgctggtacaggggacggaccgcatcgccacggtgcatgcccggctggcgcagctgctggctccgcaatggccggtggtgatcaaggagagtccgctgtcgctgggcgagatgcggcagatgatgcagtggcatcgctaccgcagcaatgatcctggcatccagtggctgcgtcgggtgtttctggagagtgcgcaggagatggatgcggcgctgccaggcatctgctga
GFPmut3b	CDS	iGEM registry part number: BBa_E0040; Green Fluorescent Protein gene		atgcgtaaaggagaagaacttttcactggagttgtcccaattcttgttgaattagatggtgatgttaatgggcacaaattttctgtcagtggagagggtgaaggtgatgcaacatacggaaaacttacccttaaatttatttgcactactggaaaactacctgttccatggccaacacttgtcactactttcggttatggtgttcaatgctttgcgagatacccagatcatatgaaacagcatgactttttcaagagtgccatgcccgaaggttatgtacaggaaagaactatatttttcaaagatgacgggaactacaagacacgtgctgaagtcaagtttgaaggtgatacccttgttaatagaatcgagttaaaaggtattgattttaaagaagatggaaacattcttggacacaaattggaatacaactataactcacacaatgtatacatcatggcagacaaacaaaagaatggaatcaaagttaacttcaaaattagacacaacattgaagatggaagcgttcaactagcagaccattatcaacaaaatactccaattggcgatggccctgtccttttaccagacaaccattacctgtccacacaatctgccctttcgaaagatcccaacgaaaagagagatcacatggtccttcttgagtttgtaacagctgctgggattacacatggcatggatgaactatacaaataataa
Term1	Terminator	iGEM registry part number: BBa_B0015; Bacterial transcription terminator		ccaggcatcaaataaaacgaaaggctcagtcgaaagactgggcctttcgttttatctgttgtttgtcggtgaacgctctctactagagtcacactggctcaccttcgggtgggcctttctgcgtttata

Supplementary Table S2. Collection of DNA parts for the first module, a naringenin-responsive transcription factor FdeR.

Name	Description	Antibiotic	Source
pBiosensor358	FdeR-based naringenin responsive biosensor.	Ampicillin	Trabelsi et al. 2018
pL0C	Level 0 acceptor vector.	Chloramphenicol	SB2CL
pL0C_P1	Level 0 vectors carrying the P1 promoter	Chloramphenicol	SB2CL
pL0C_P2	Level 0 vectors carrying the P2 promoter	Chloramphenicol	SB2CL
pL0C_P3	Level 0 vectors carrying the P3 promoter	Chloramphenicol	SB2CL
pL0C_P4	Level 0 vectors carrying the P4 promoter	Chloramphenicol	SB2CL
pL0C_R1	Level 0 vectors carrying the R1 RBS	Chloramphenicol	SB2CL
pL0C_R2	Level 0 vectors carrying the R2 RBS	Chloramphenicol	SB2CL
pL0C_R3	Level 0 vectors carrying the R3 RBS	Chloramphenicol	SB2CL
pL0C_R4	Level 0 vectors carrying the R4 RBS	Chloramphenicol	SB2CL
pL0C_R5	Level 0 vectors carrying the R5 RBS	Chloramphenicol	SB2CL
pL0C_T1	Level 0 vector carrying the T1 terminator.	Chloramphenicol	SB2CL
pL0C_GFP	Level 0 vector carrying the GFPmut3b gene.	Chloramphenicol	SB2CL
pL0C_fdeO	Level 0 vector carrying the fdeR operator region.	Chloramphenicol	This study
pL0C_fdeR	Level 0 vector carrying the fdeR gene.	Chloramphenicol	This study
pAlpha1	Level 1 acceptor vector modified from Golden Braid original vectors. It contains RFP dropout gene for cloning purposes.	Kanamycin	SB2CL
pA1_P1-R1-GFP-T1	Level 1 vector carrying the P1-R1-GFP circuit	Kanamycin	This study
pA1_P2-R1-GFP-T1	Level 1 vector carrying the P2-R1-GFP circuit	Kanamycin	This study
pA1_P3-R1-GFP-T1	Level 1 vector carrying the P3-R1-GFP circuit	Kanamycin	This study
pA1_P4-R1-GFP-T1	Level 1 vector carrying the P4-R1-GFP circuit	Kanamycin	This study
pA1_fdeO-GFP-T1	Level 1 vector carrying the fdeO_GFP_T1 circuit.	Kanamycin	This study
pAlpha2	Level 1 acceptor vector modified from Golden Braid original vectors. It contains RFP dropout gene for cloning purposes.	Kanamycin	SB2CL
pA2_P1-R1-fdeR-T1	Level 1 vector carrying the P1-R1-fdeR-T1 circuit	Kanamycin	This study
pA2_P1-R2-fdeR-T1	Level 1 vector carrying the P1-R2-fdeR-T1 circuit	Kanamycin	This study
pA2_P1-R3-fdeR-T1	Level 1 vector carrying the P1-R3-fdeR-T1 circuit	Kanamycin	This study
pA2_P1-R4-fdeR-T1	Level 1 vector carrying the P1-R4-fdeR-T1 circuit	Kanamycin	This study
pA2_P1-R5-fdeR-T1	Level 1 vector carrying the P1-R5-fdeR-T1 circuit	Kanamycin	This study
pA2_P2-R2-fdeR-T1	Level 1 vector carrying the P2-R2-fdeR-T1 circuit	Kanamycin	This study
pA2_P2-R3-fdeR-T1	Level 1 vector carrying the P2-R3-fdeR-T1 circuit	Kanamycin	This study
pA2_P2-R4-fdeR-T1	Level 1 vector carrying the P2-R4-fdeR-T1 circuit	Kanamycin	This study
pA2_P2-R5-fdeR-T1	Level 1 vector carrying the P2-R5-fdeR-T1 circuit	Kanamycin	This study
pA2_P3-R1-fdeR-T1	Level 1 vector carrying the P3-R1-fdeR-T1 circuit	Kanamycin	This study
pA2_P3-R2-fdeR-T1	Level 1 vector carrying the P3-R2-fdeR-T1 circuit	Kanamycin	This study
pA2_P3-R3-fdeR-T1	Level 1 vector carrying the P3-R3-fdeR-T1 circuit	Kanamycin	This study
pA2_P3-R4-fdeR-T1	Level 1 vector carrying the P3-R4-fdeR-T1 circuit	Kanamycin	This study
pA2_P3-R5-fdeR-T1	Level 1 vector carrying the P3-R5-fdeR-T1 circuit	Kanamycin	This study
pA2_P4-R1-fdeR-T1	Level 1 vector carrying the P4-R1-fdeR-T1 circuit	Kanamycin	This study
pA2_P4-R2-fdeR-T1	Level 1 vector carrying the P4-R2-fdeR-T1 circuit	Kanamycin	This study
pA2_P4-R4-fdeR-T1	Level 1 vector carrying the P4-R4-fdeR-T1 circuit	Kanamycin	This study
pOmega1	Level 2 acceptor vector modified from Golden Braid original vectors. It contains a spectinomycin resistance gene, pUC origin of replication and a RFP dropout gene for cloning purposes.	Spectinomycin	SB2CL
pO1_fdeO-GFP-T1_P1-R1-fdeR-T1	Level 2 vector carrying the fdeO-GFP-T1_P1-R1-fdeR-T1 circuit	Spectinomycin	This study
pO1_fdeO-GFP-T1_P1-R2-fdeR-T1	Level 2 vector carrying the pO1_fdeO-GFP-T1_P1-R2-fdeR-T1 circuit	Spectinomycin	This study
pO1_fdeO-GFP-T1_P1-R3-fdeR-T1	Level 2 vector carrying the pO1_fdeO-GFP-T1_P1-R3-fdeR-T1 circuit	Spectinomycin	This study
pO1_fdeO-GFP-T1_P1-R4-fdeR-T1	Level 2 vector carrying the pO1_fdeO-GFP-T1_P1-R4-fdeR-T1 circuit	Spectinomycin	This study

Supplementary Table S3. Design of experiments for the combinatorial libraries. **(A)** First iteration library. **(B)** Second iteration library. **(C)** Validation library.

A

Experiment	Media	Substrate	Promoter	RBS
E1	M2	S0	P1	R1
E2	M1	S3	P1	R1
E3	M3	S2	P1	R2
E4	M1	S3	P1	R2
E5	M3	S3	P1	R3
E6	M0	S1	P1	R3
E7	M0	S0	P1	R4
E8	M1	S1	P1	R4
E9	M2	S2	P1	R5
E10	M1	S0	P1	R5
E11	M0	S0	P2	R2
E12	M2	S1	P2	R2
E13	M1	S2	P2	R3
E14	M1	S1	P2	R3
E15	M2	S2	P2	R3
E16	M2	S3	P2	R4
E17	M3	S0	P2	R5
E18	M3	S3	P2	R5
E19	M3	S1	P3	R1
E20	M0	S3	P3	R1
E21	M2	S1	P3	R1
E22	M1	S2	P3	R2
E23	M2	S3	P3	R3
E24	M2	S0	P3	R3
E25	M0	S2	P3	R4
E26	M3	S2	P3	R4
E27	M1	S0	P3	R5
E28	M0	S1	P3	R5
E29	M3	S0	P4	R2
E30	M0	S3	P4	R2
E31	M0	S2	P4	R4
E32	M3	S1	P4	R4

B

Experiment	Media	Substrate	Promoter	RBS
E33	M0	S1	P1	R1
E34	M0	S2	P1	R1
E35	M0	S0	P1	R2
E36	M0	S1	P1	R2
E37	M0	S3	P1	R2
E38	M1	S2	P1	R3
E39	M2	S1	P1	R3
E40	M2	S2	P1	R4
E41	M0	S3	P1	R5
E42	M3	S1	P2	R2
E43	M3	S3	P2	R2
E44	M1	S3	P2	R3
E45	M3	S0	P2	R3
E46	M0	S0	P2	R4
E47	M1	S1	P2	R4
E48	M0	S3	P2	R5

C

Experiment	Media	Substrate	Promoter	RBS
E49	M1	S3	P2	R5
E50	M3	S1	P2	R5
E51	M0	S0	P3	R2
E52	M3	S0	P3	R2
E53	M3	S1	P3	R2
E54	M3	S3	P3	R2
E55	M0	S0	P3	R3
E56	M1	S1	P3	R3
E57	M2	S1	P3	R3
E58	M1	S3	P3	R4
E59	M2	S0	P3	R4
E60	M2	S1	P3	R4
E61	M2	S3	P3	R4
E62	M2	S1	P4	R2
E63	M2	S2	P4	R4
E64	M3	S3	P4	R4

Supplementary Figure S1. Visual description of the method. Starting from the biosensor backbone for the desired target (naringenin), a combinatorial library based on *D*-optimal design of experiments for context factors was built and tested. A mechanistic model was defined for growth and the reporter response (GFP/OD) and calibrated from the experimental data. A bootstrap data aggregation (bagging) strategy was used to generate distributions of the parameters for training and validate a deep learning model. The resulting model can be used to predict the response under a new given context.

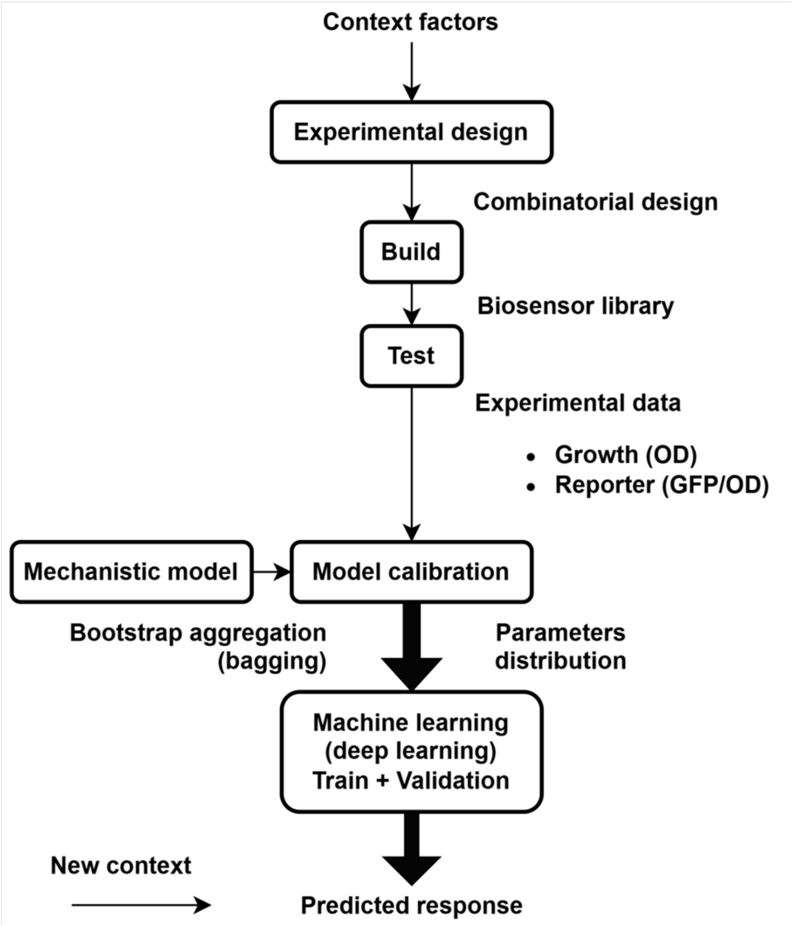

Supplementary Figure S2. Dose-response curve for the reference biosensor construct. To characterize the steady-state response of the biosensor against different concentrations, a dose-response curve was obtained growing overnight the reference construct (fdeO-GFP-T1-P1-R4-fdeR-T1) in different naringenin concentrations (0, 1, 5, 10, 50, 100, 250, 500, 750, and 1000 µM). The means of the maximum normalized fluorescence values of each experiment were used to obtain the dose-response curve shown in the Figure. Notably, the inducer's first detectable concentration was 5 µM, a concentration that is discernible from the background signal. The biosensor signal positively correlated with naringenin concentration throughout the tested range. Based on these findings, a working reference concentration of 400 µM naringenin was determined for subsequent experiments. It is worth mentioning that higher concentrations (750 and 1000 µM) may lead to naringenin precipitation issues in the media which explains the higher variance observed at these concentrations.

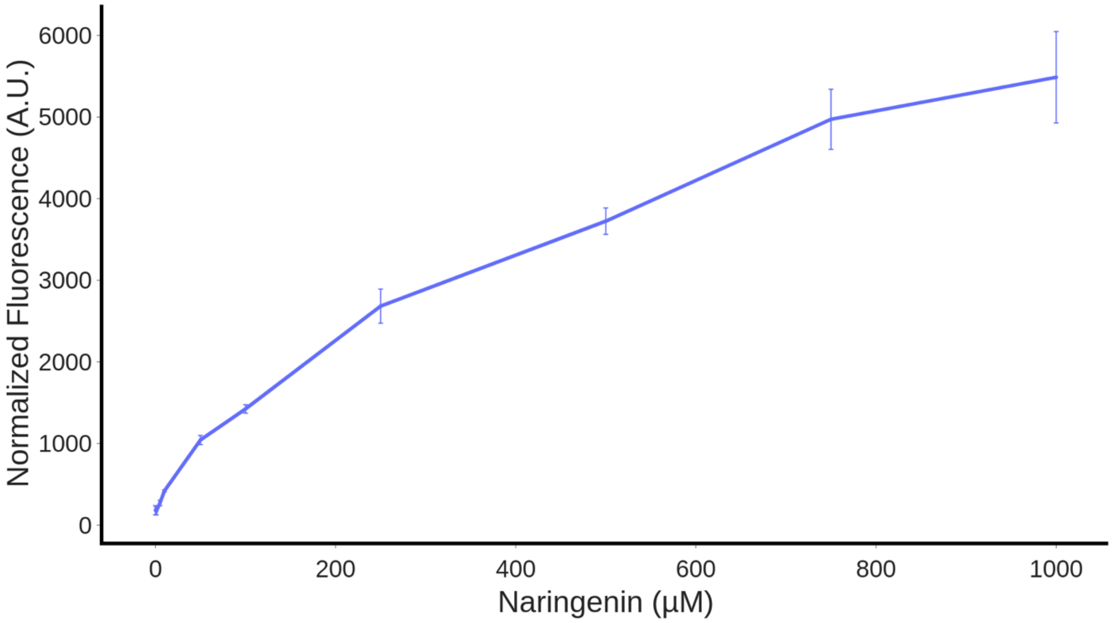

Supplementary Figure S3. Experimental (blue dotted) vs simulated (orange) vs predicted (green) growth curves of the biosensor, measured as ODs, for the initial 32 combinatorial library, using leave-one-out cross validation. Experiment 5 from the DoE library was removed from the training set due to assembly issues (resulting in a non-functional response), whereas experiments 6 and 29 were kept because they were dynamically functional, although their readings were of lower intensity than the rest of the library due to potential experimental issues.

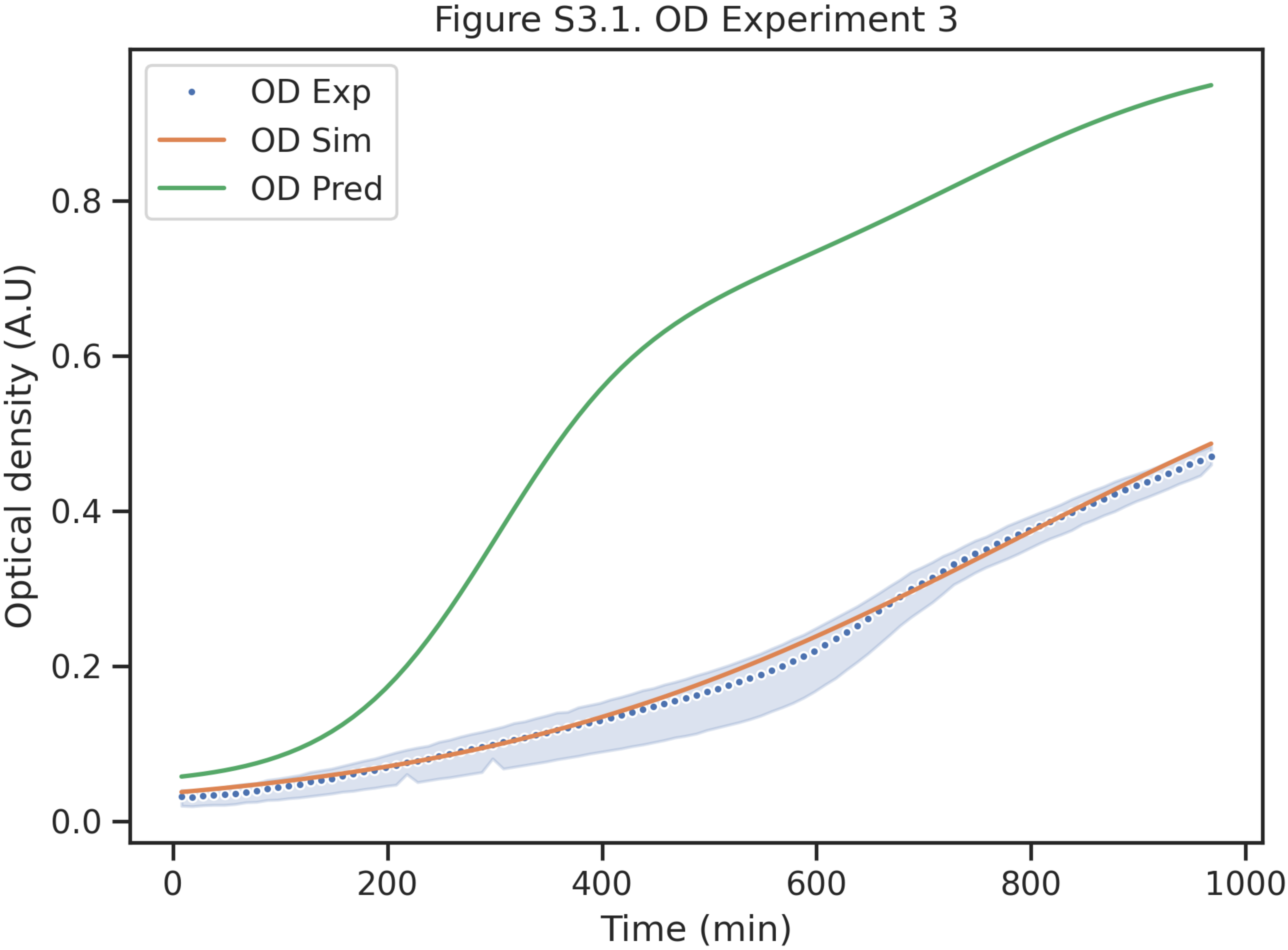
Figure S3.1. OD Experiment 3
OD Exp
OD Sim
OD Pred
Optical density (A.U)
0.0
0.2
0.4
0.6
0.8
0
200
400
600
800
1000
Time (min)

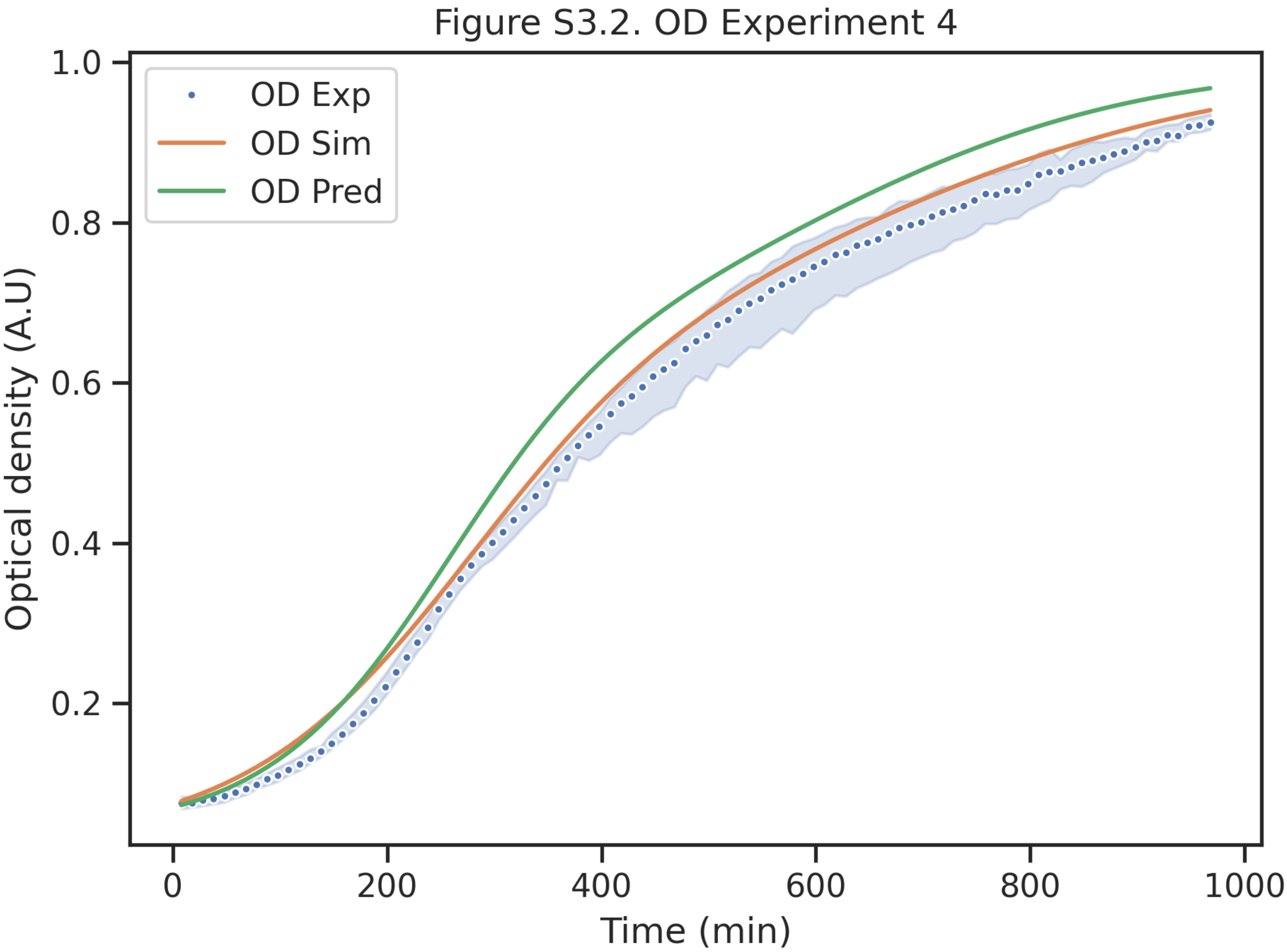
Figure S3.2. OD Experiment 4
OD Exp
OD Sim
OD Pred
Optical density (A.U)
Time (min)
1.0
0.8
0.6
0.4
0.2
0
200
400
600
800
1000

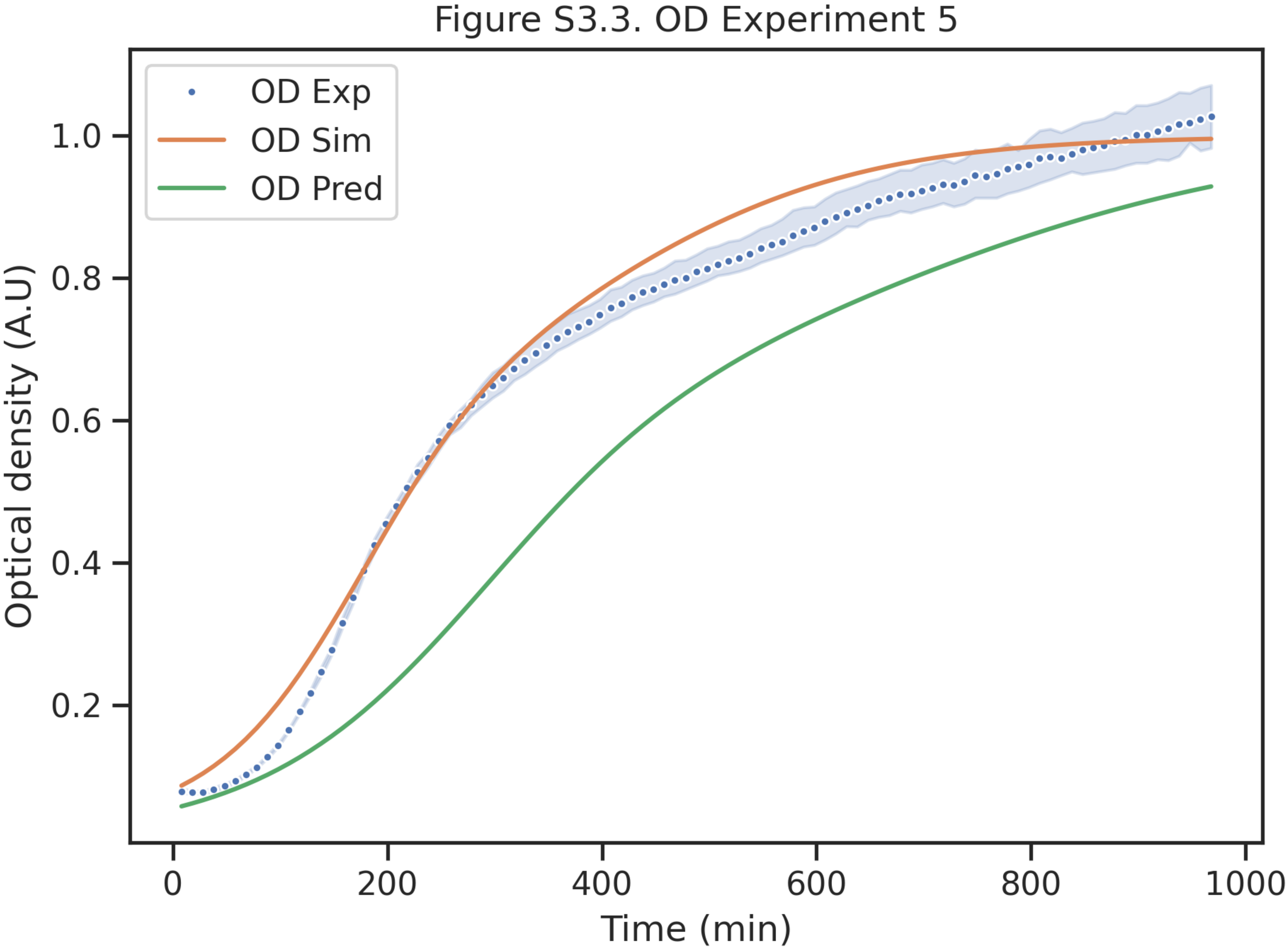
Figure S3.3. OD Experiment 5
OD Exp
OD Sim
OD Pred
Optical density (A.U)
0.2
0.4
0.6
0.8
1.0
0
200
400
600
800
1000
Time (min)

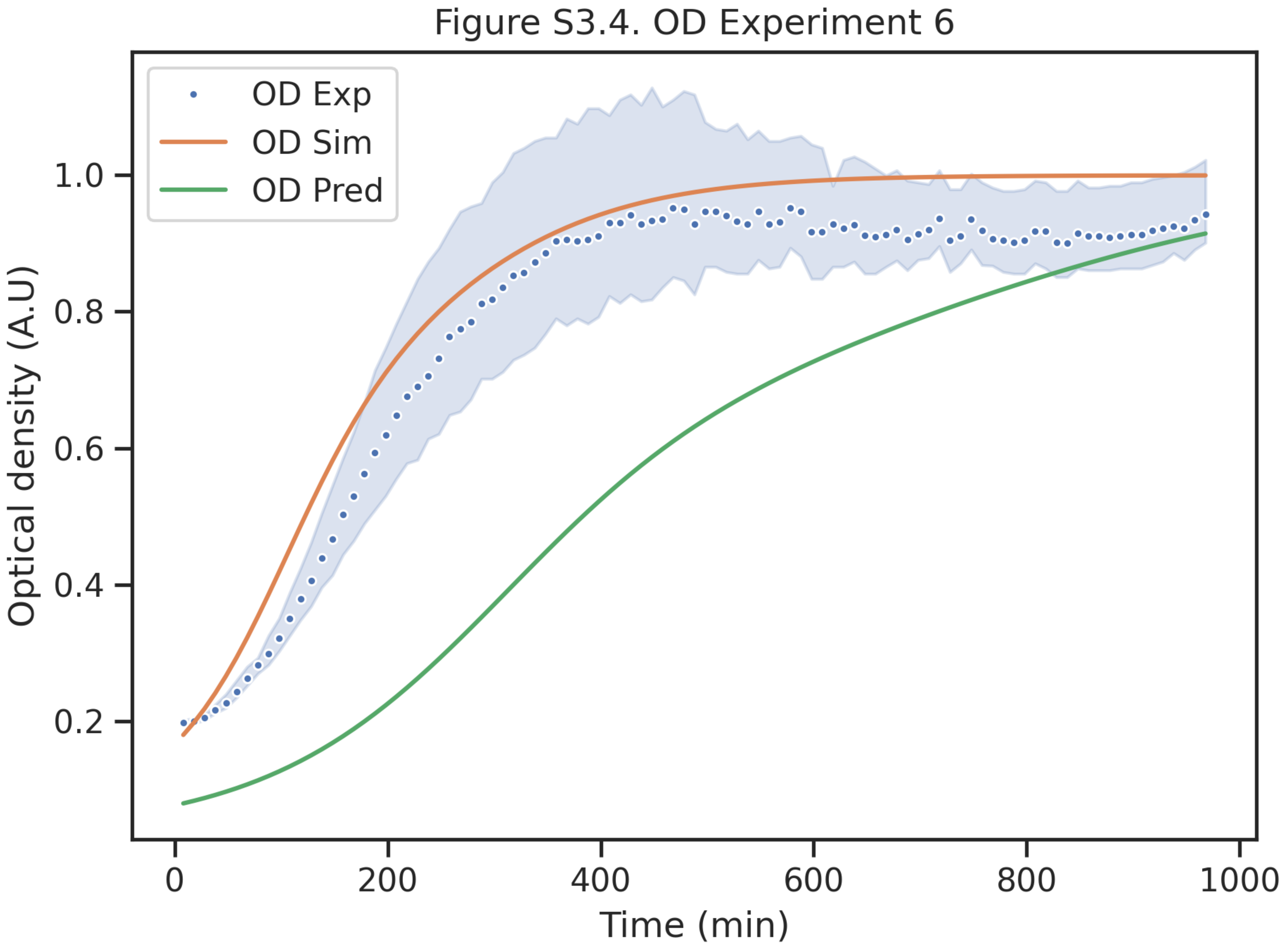

Figure S3.4. OD Experiment 6
OD Exp
OD Sim
OD Pred
Optical density (A.U)
Time (min)
0.2
0.4
0.6
0.8
1.0
0
200
400
600
800
1000

Figure S3.5. OD Experiment 7

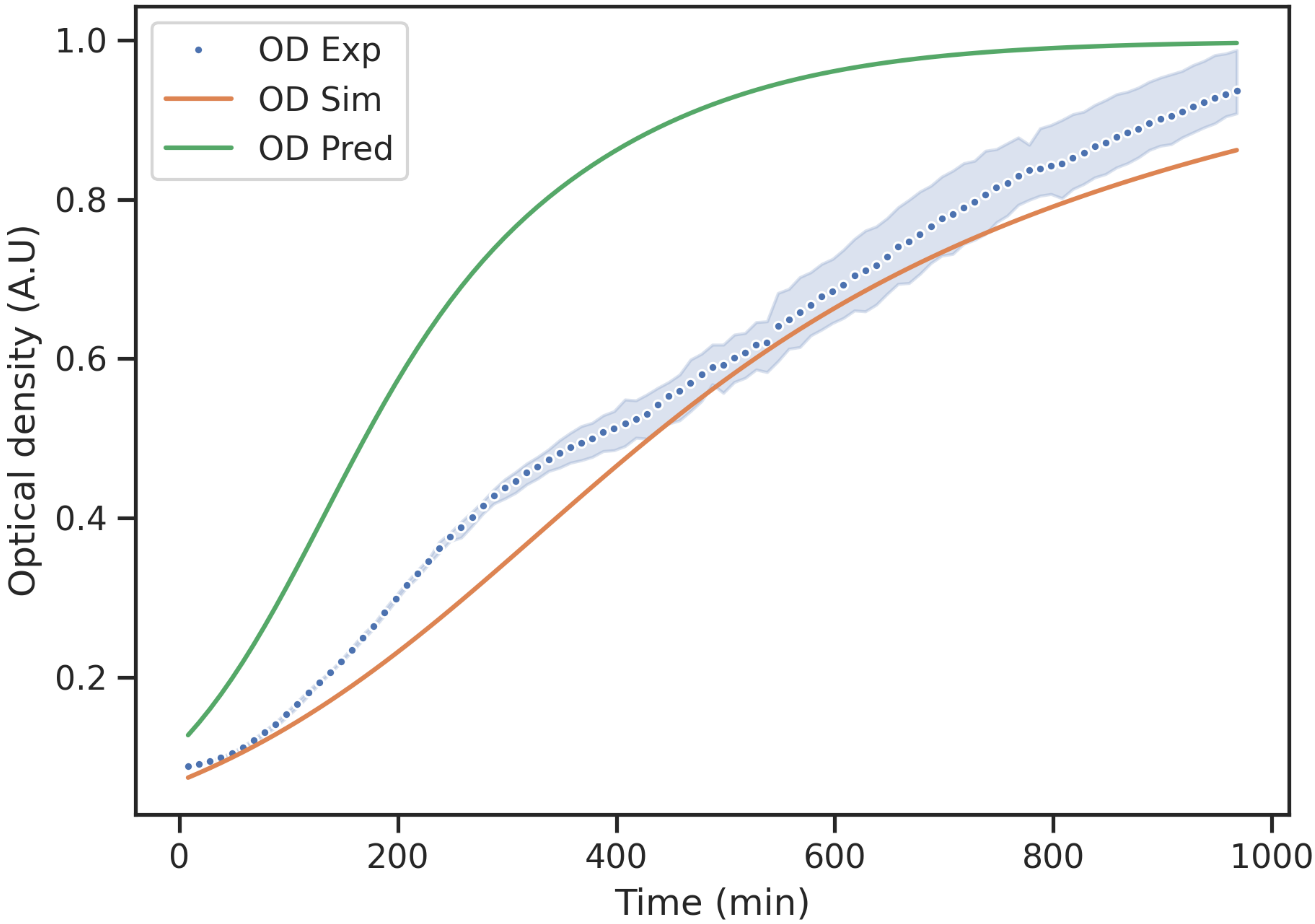

Figure S3.6. OD Experiment 8

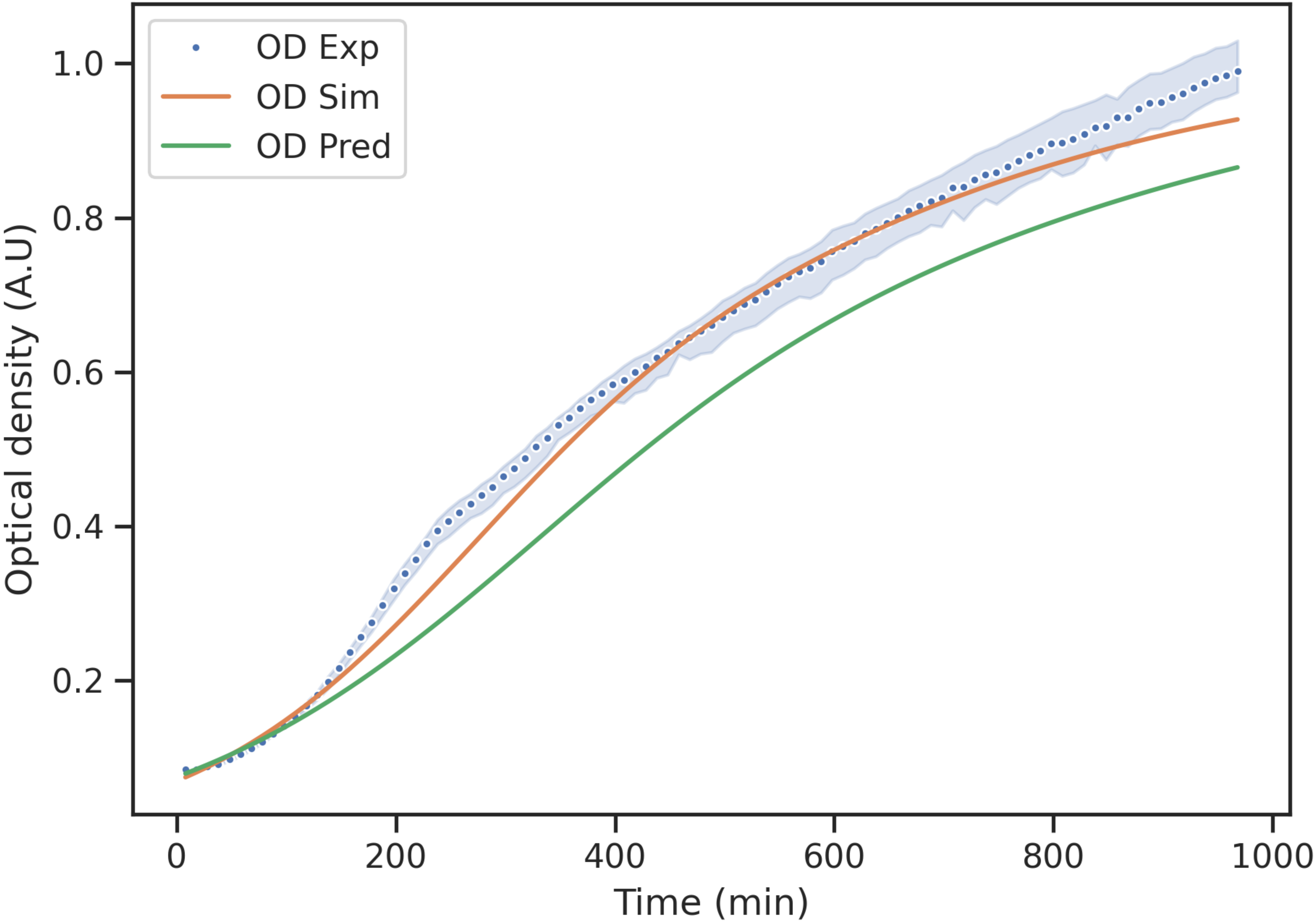

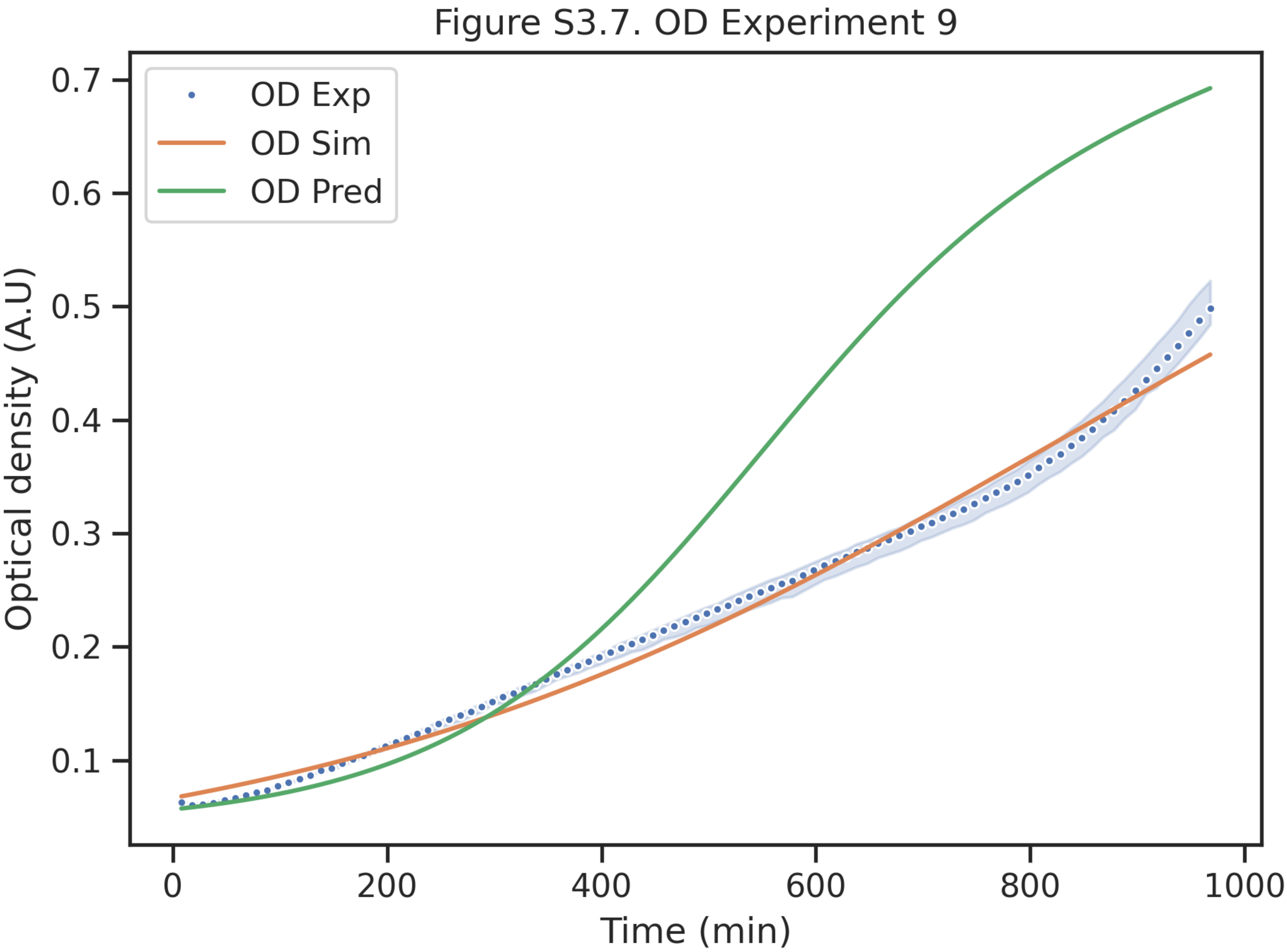
Figure S3.7. OD Experiment 9
OD Exp
OD Sim
OD Pred
Optical density (A.U)
Time (min)
0.1
0.2
0.3
0.4
0.5
0.6
0.7
0
200
400
600
800
1000

Figure S3.8. OD Experiment 10

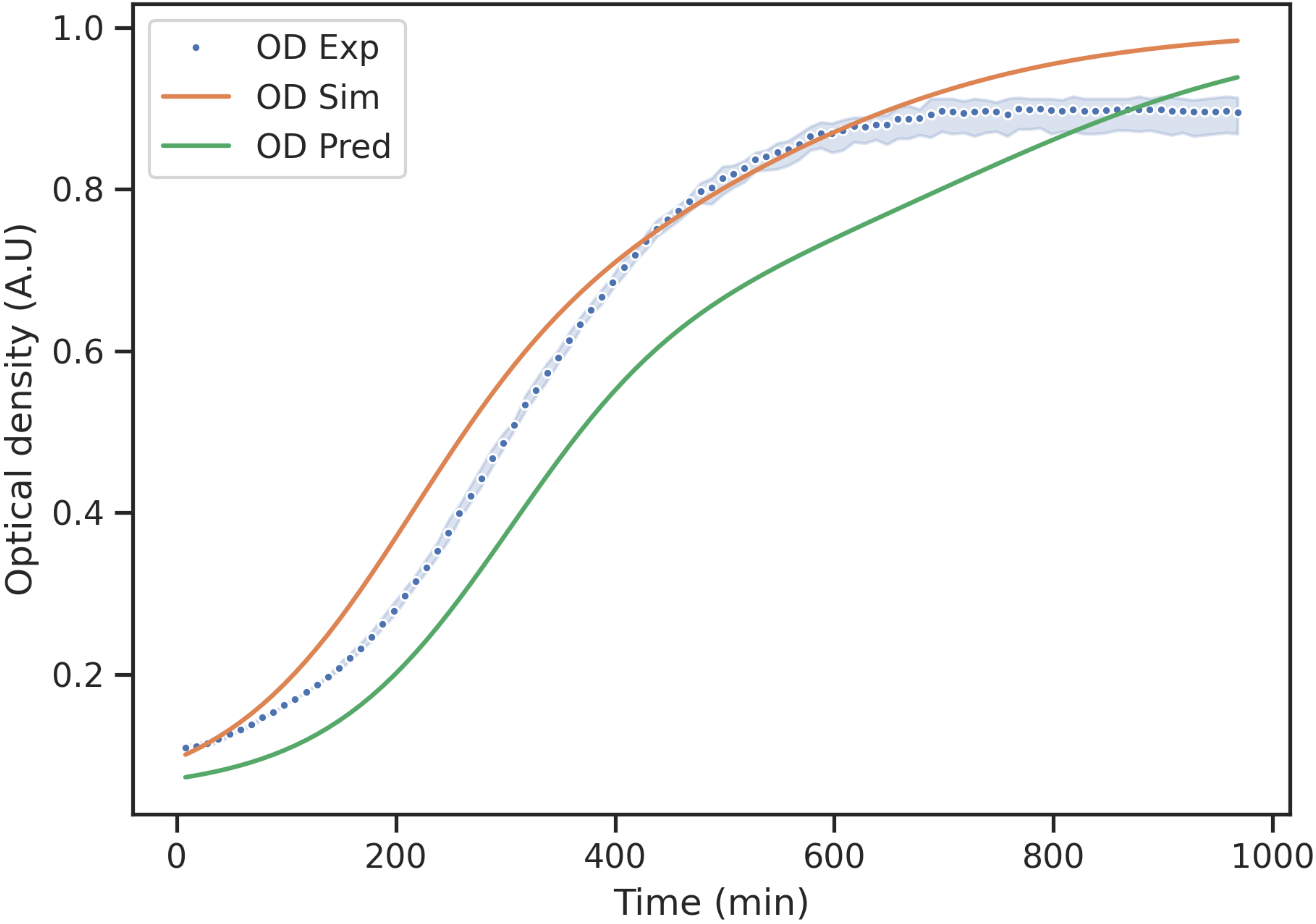

Figure S3.9. OD Experiment 11

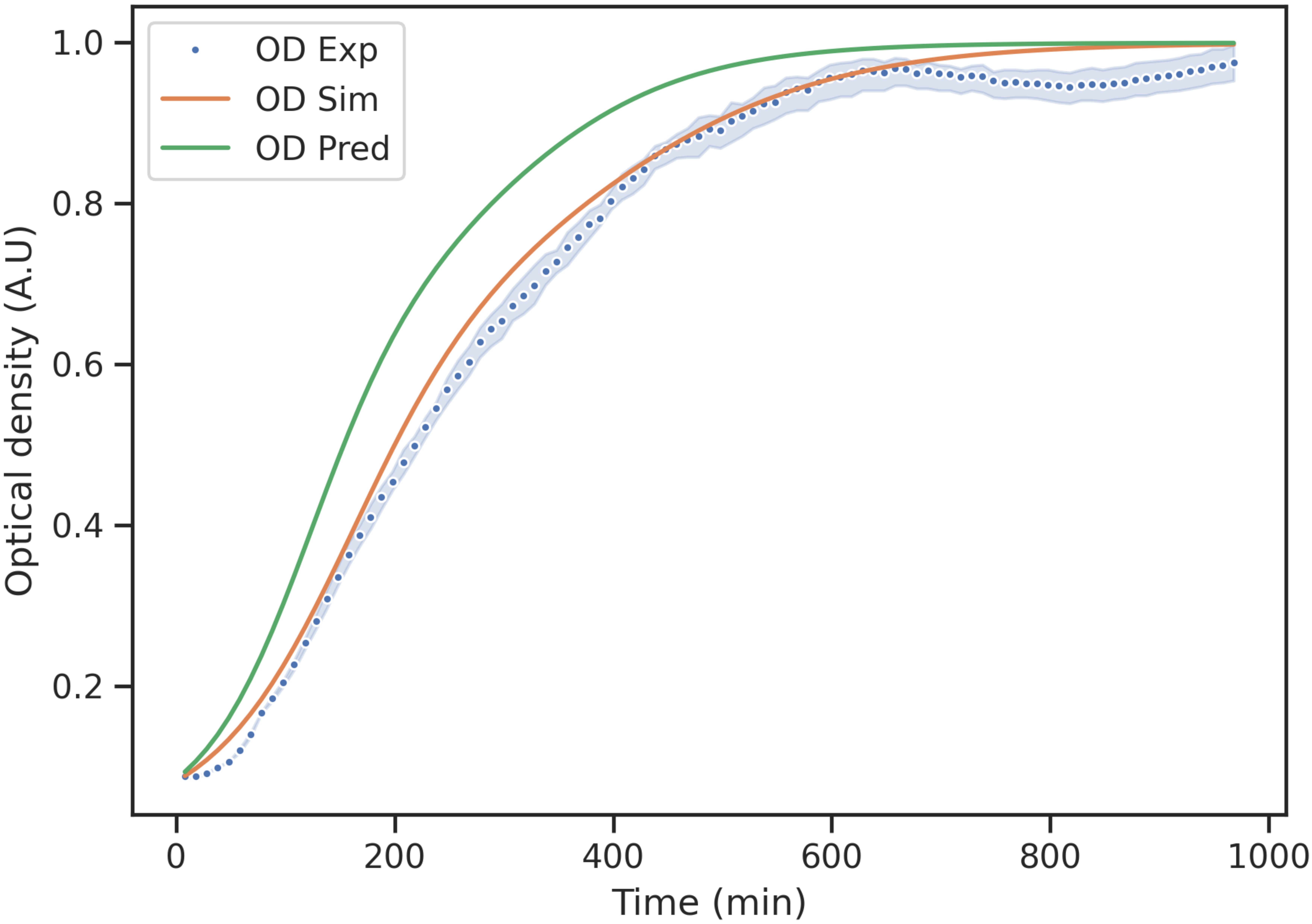

Figure S3.10. OD Experiment 12

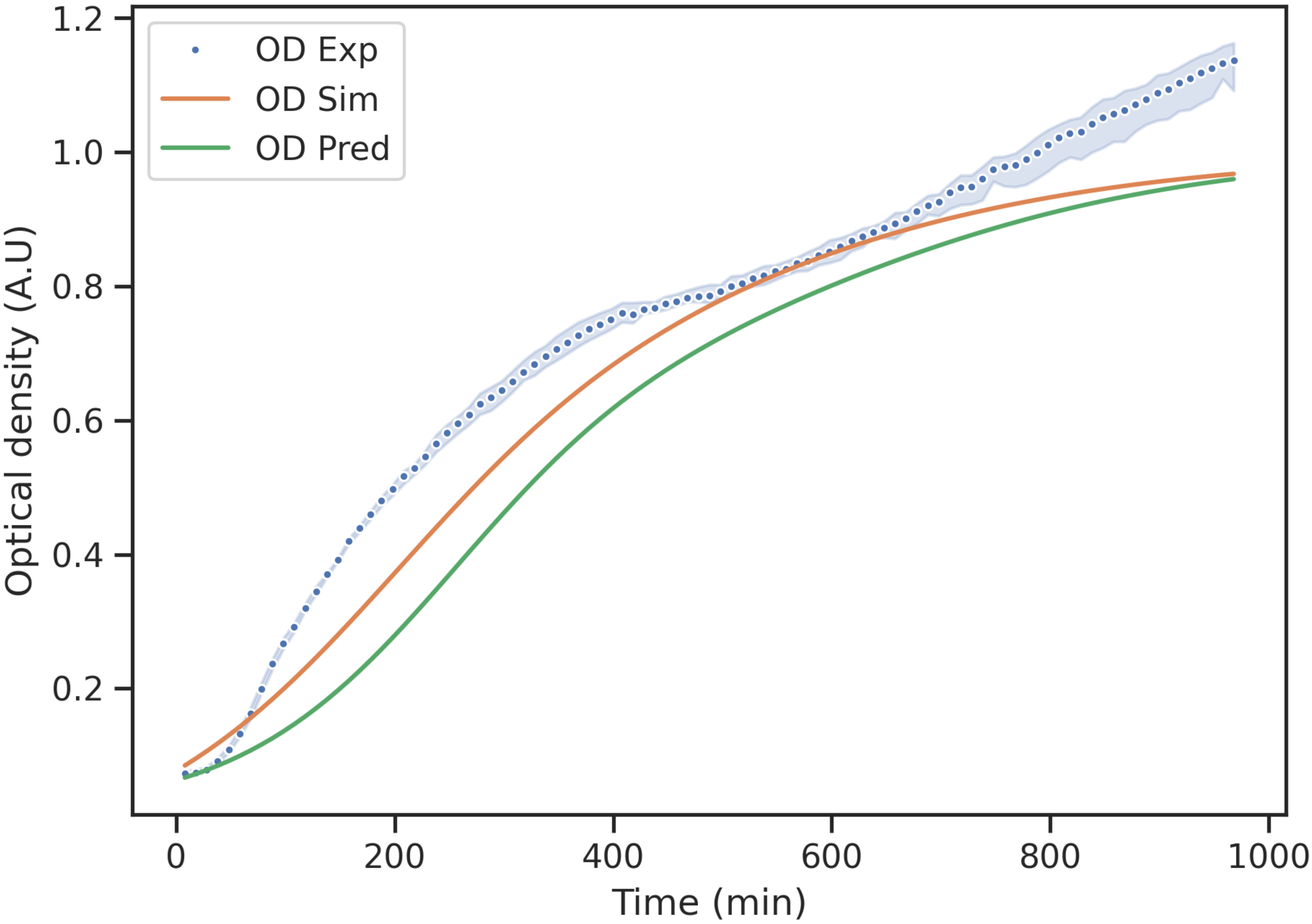

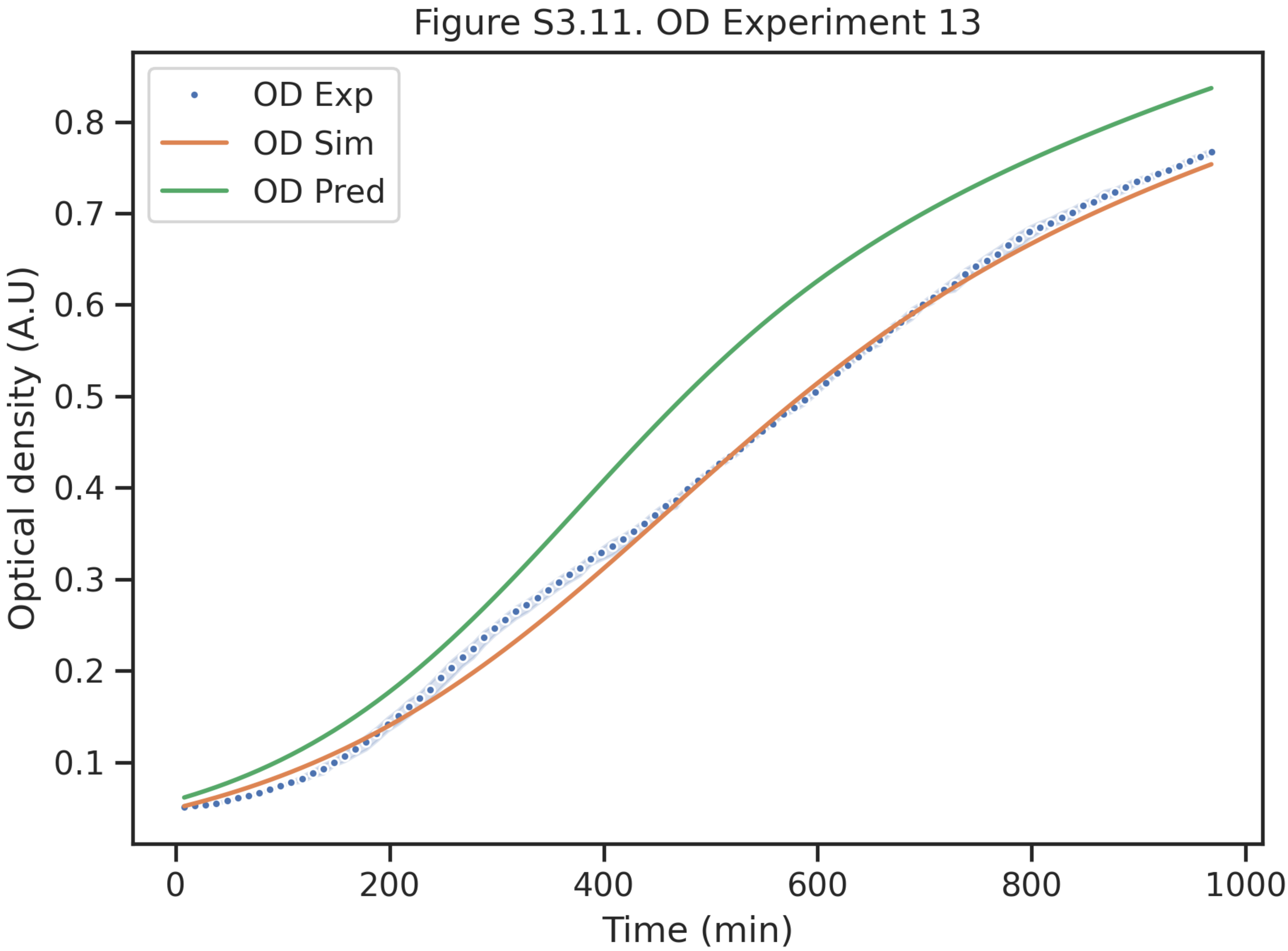
Figure S3.11. OD Experiment 13
OD Exp
OD Sim
OD Pred
Optical density (A.U)
0.1
0.2
0.3
0.4
0.5
0.6
0.7
0.8
0
200
400
600
800
1000
Time (min)

Figure S3.12. OD Experiment 14

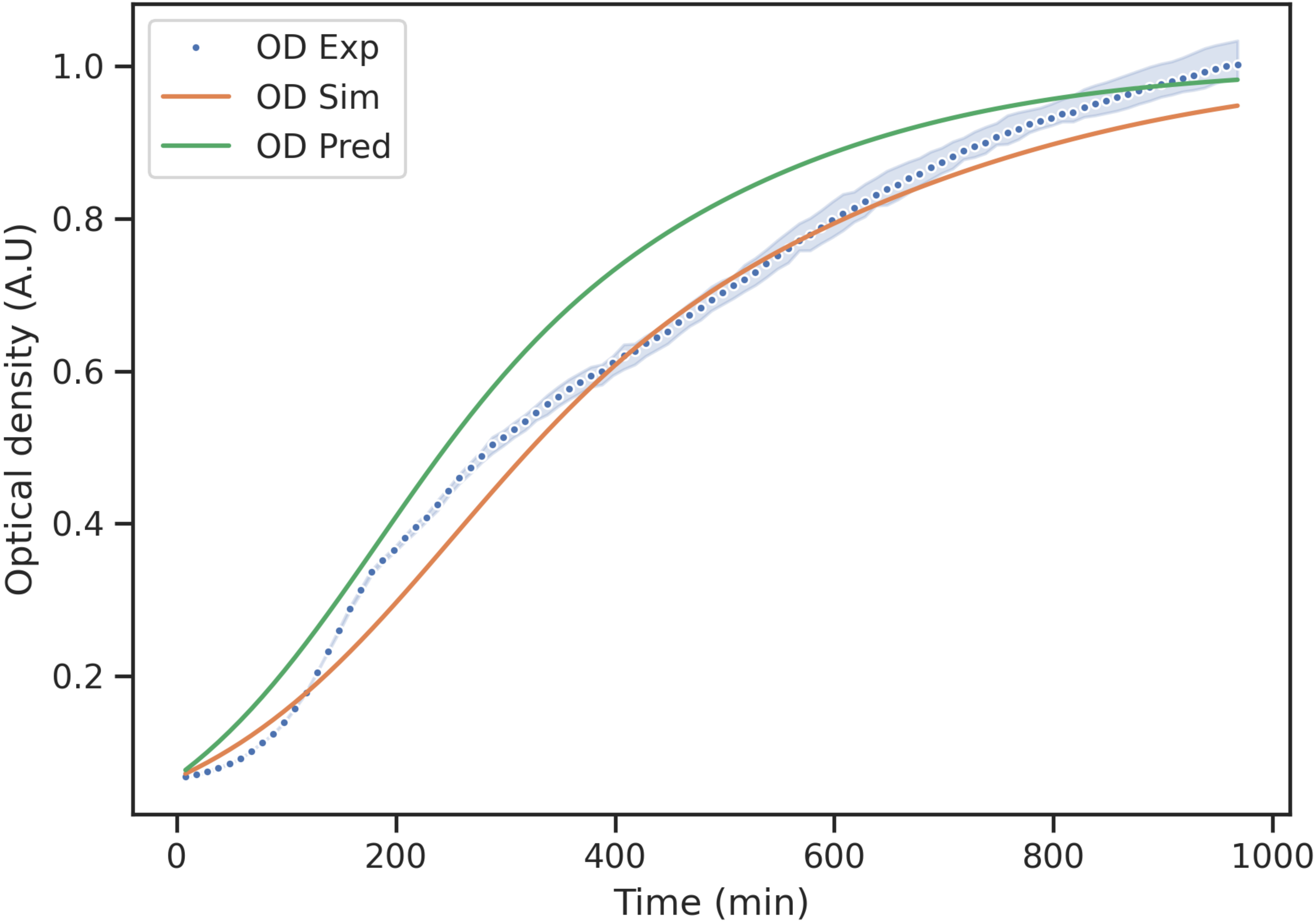

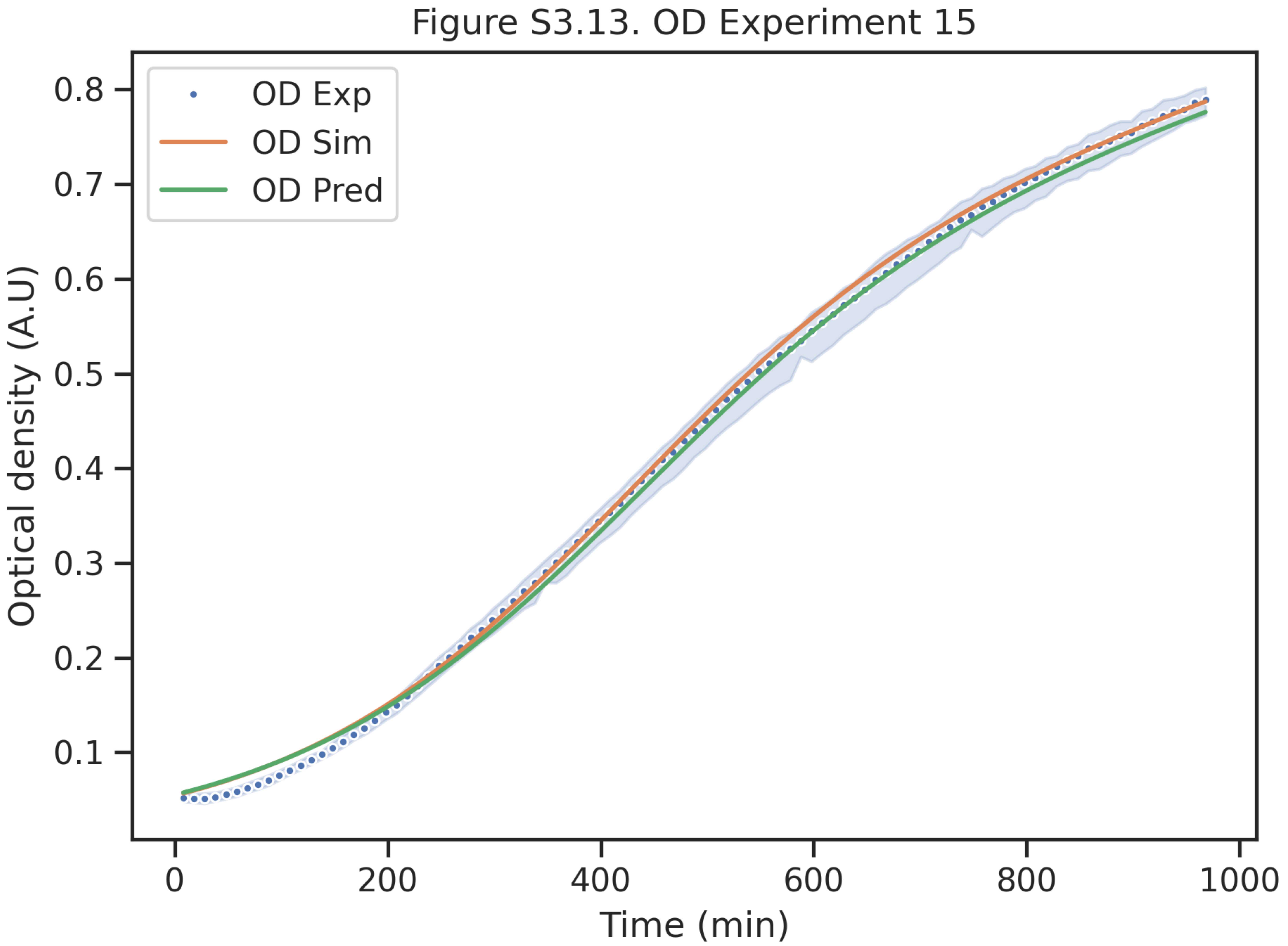
Figure S3.13. OD Experiment 15
OD Exp
OD Sim
OD Pred
Optical density (A.U)
0.1
0.2
0.3
0.4
0.5
0.6
0.7
0.8
0
200
400
600
800
1000
Time (min)

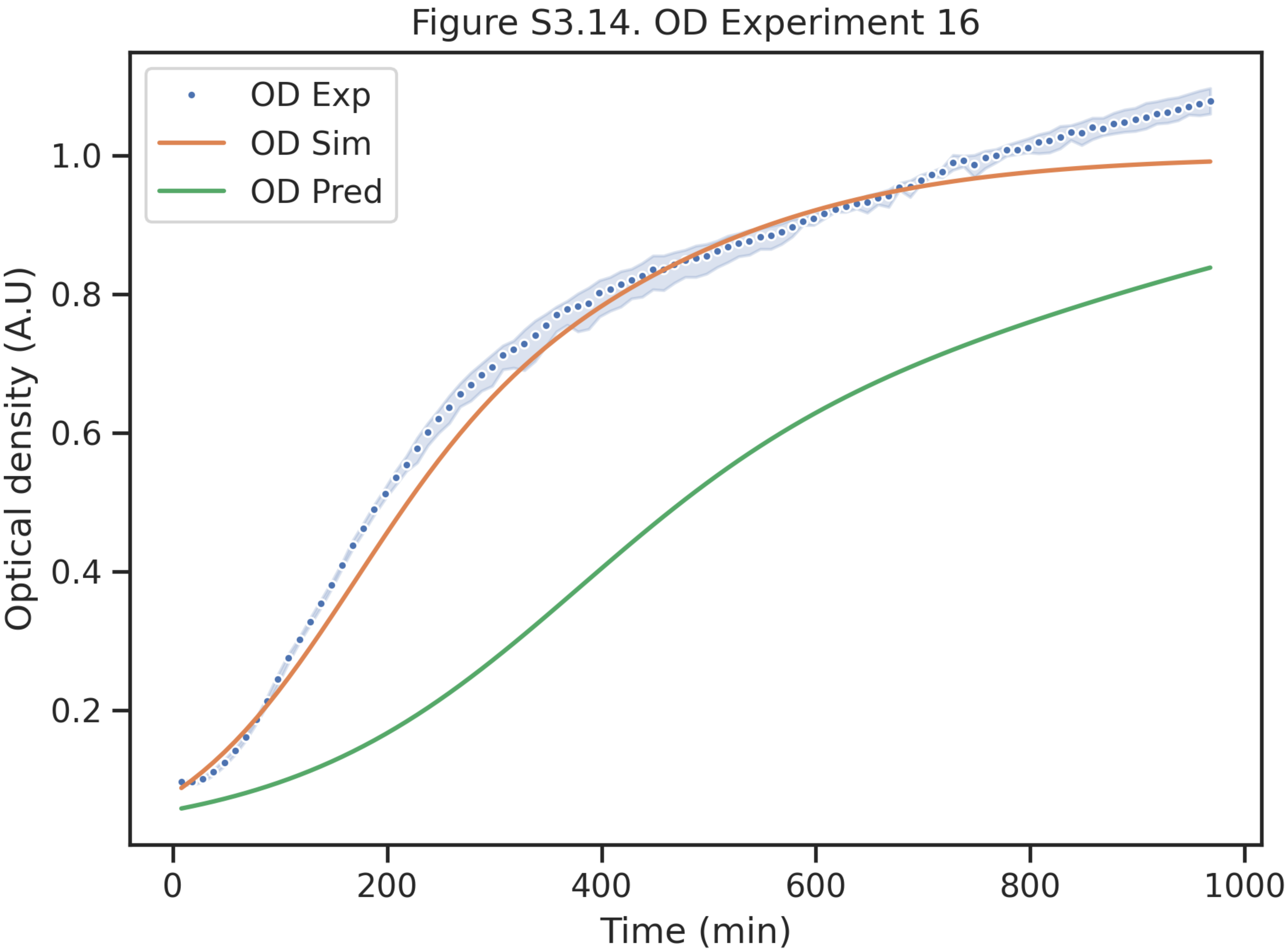
Figure S3.14. OD Experiment 16
OD Exp
OD Sim
OD Pred
Optical density (A.U)
1.0
0.8
0.6
0.4
0.2
0
200
400
600
800
1000
Time (min)

Figure S3.15. OD Experiment 17

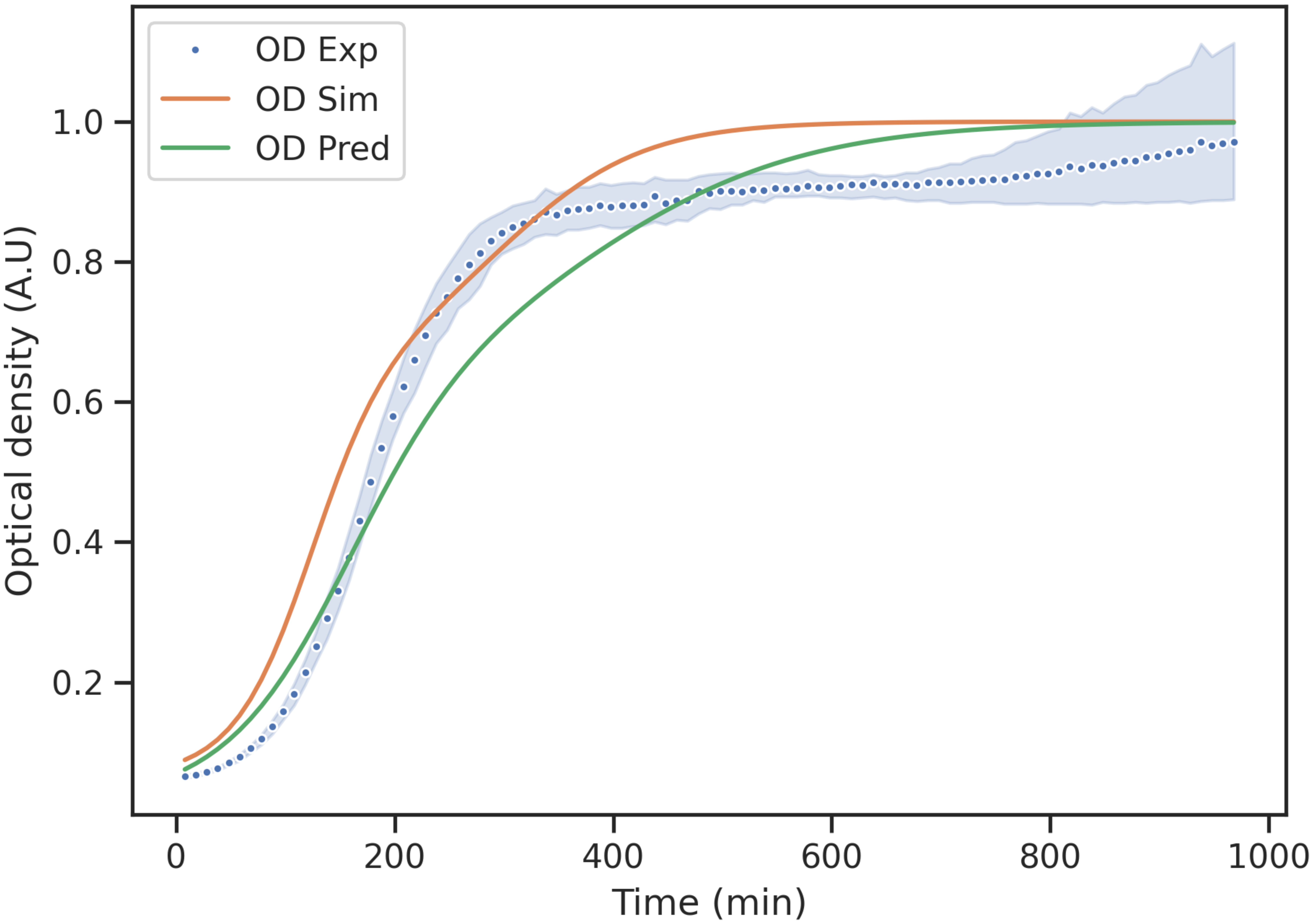

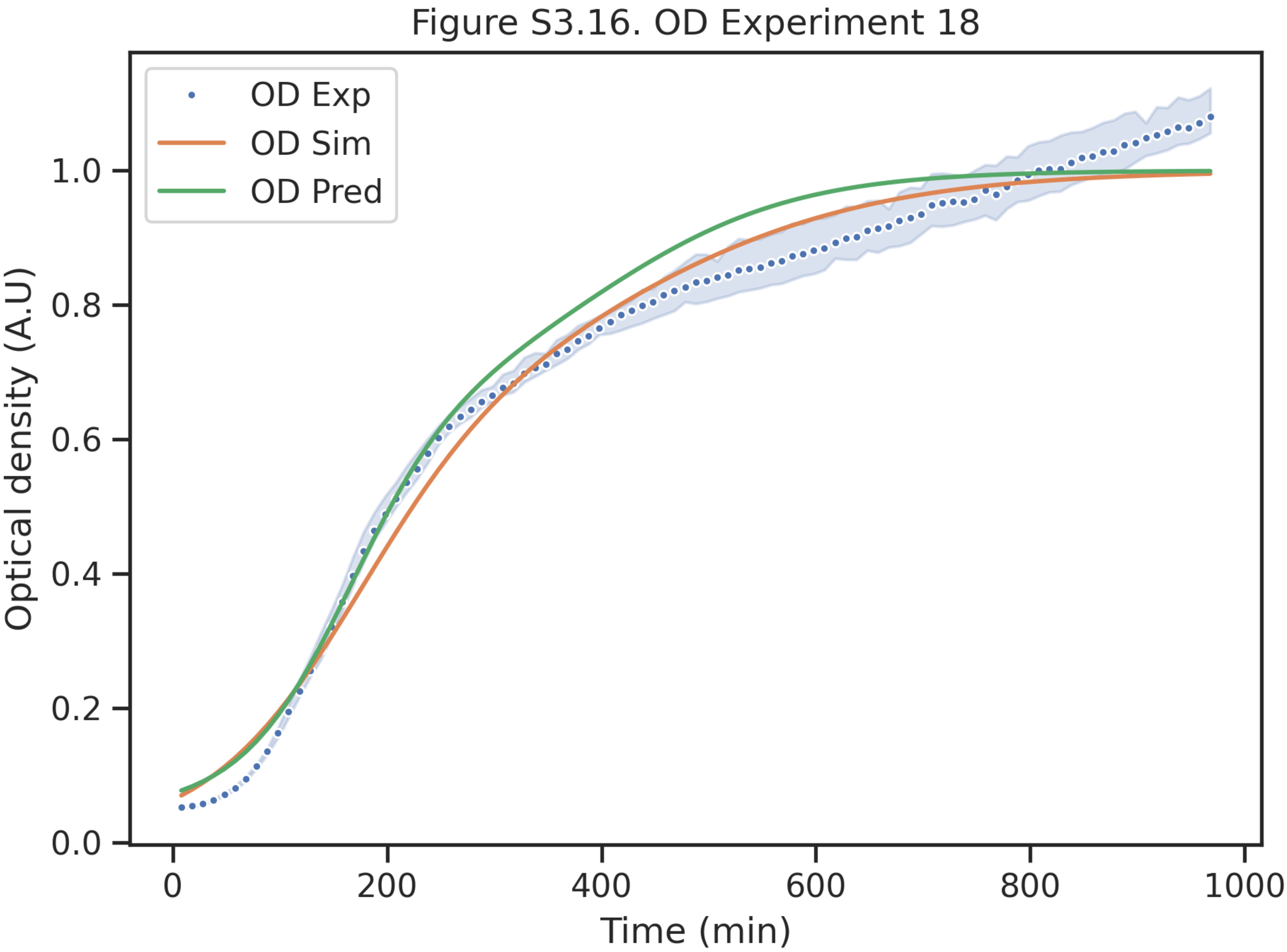
Figure S3.16. OD Experiment 18
OD Exp
OD Sim
OD Pred
1.0
0.8
0.6
0.4
0.2
0.0
Optical density (A.U)
0
200
400
600
800
1000
Time (min)

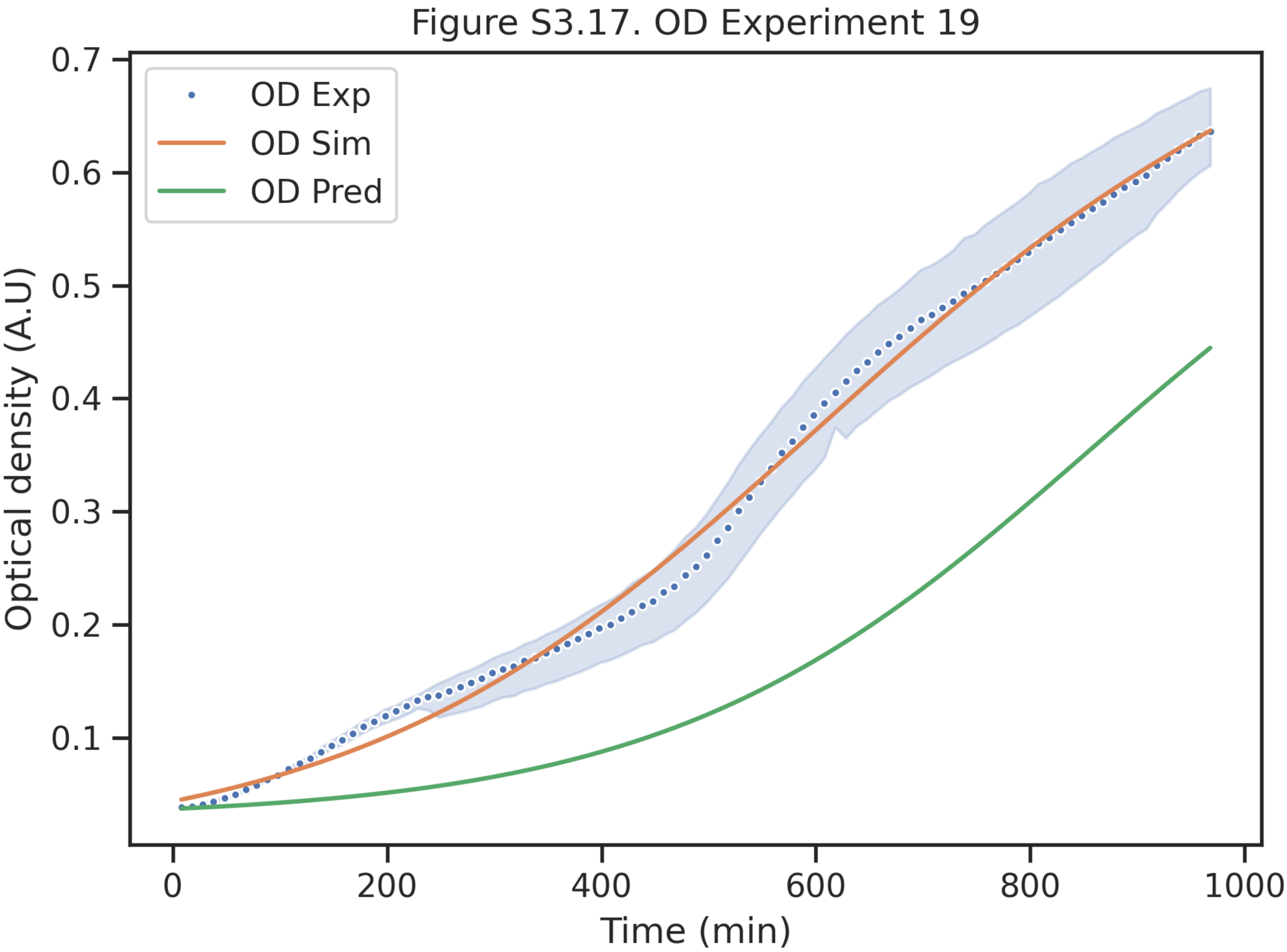

Figure S3.17. OD Experiment 19
OD Exp
OD Sim
OD Pred
Optical density (A.U)
0.1
0.2
0.3
0.4
0.5
0.6
0.7
0
200
400
600
800
1000
Time (min)

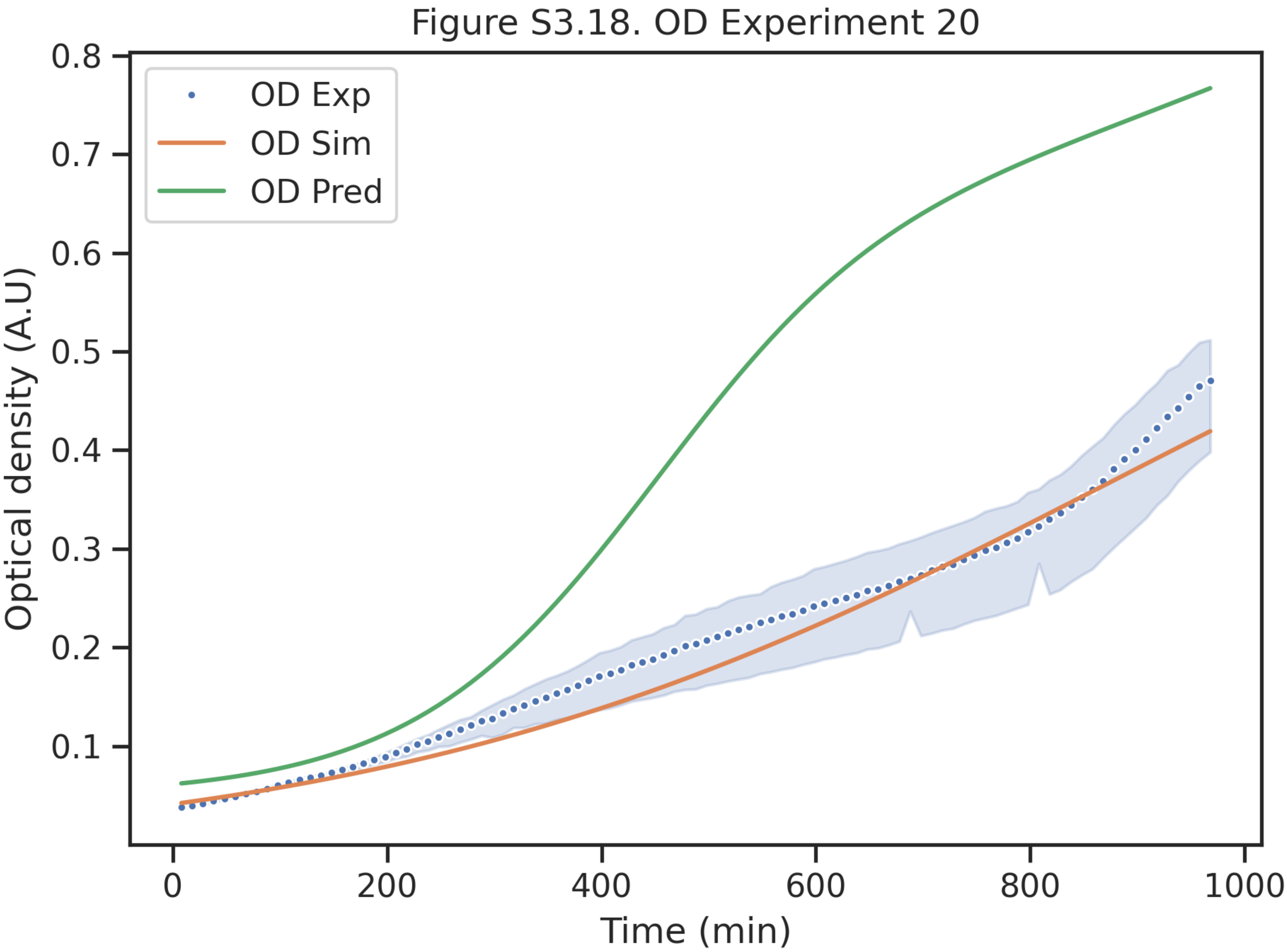

Figure S3.18. OD Experiment 20
OD Exp
OD Sim
OD Pred
Optical density (A.U)
0.8
0.7
0.6
0.5
0.4
0.3
0.2
0.1
0
200
400
600
800
1000
Time (min)

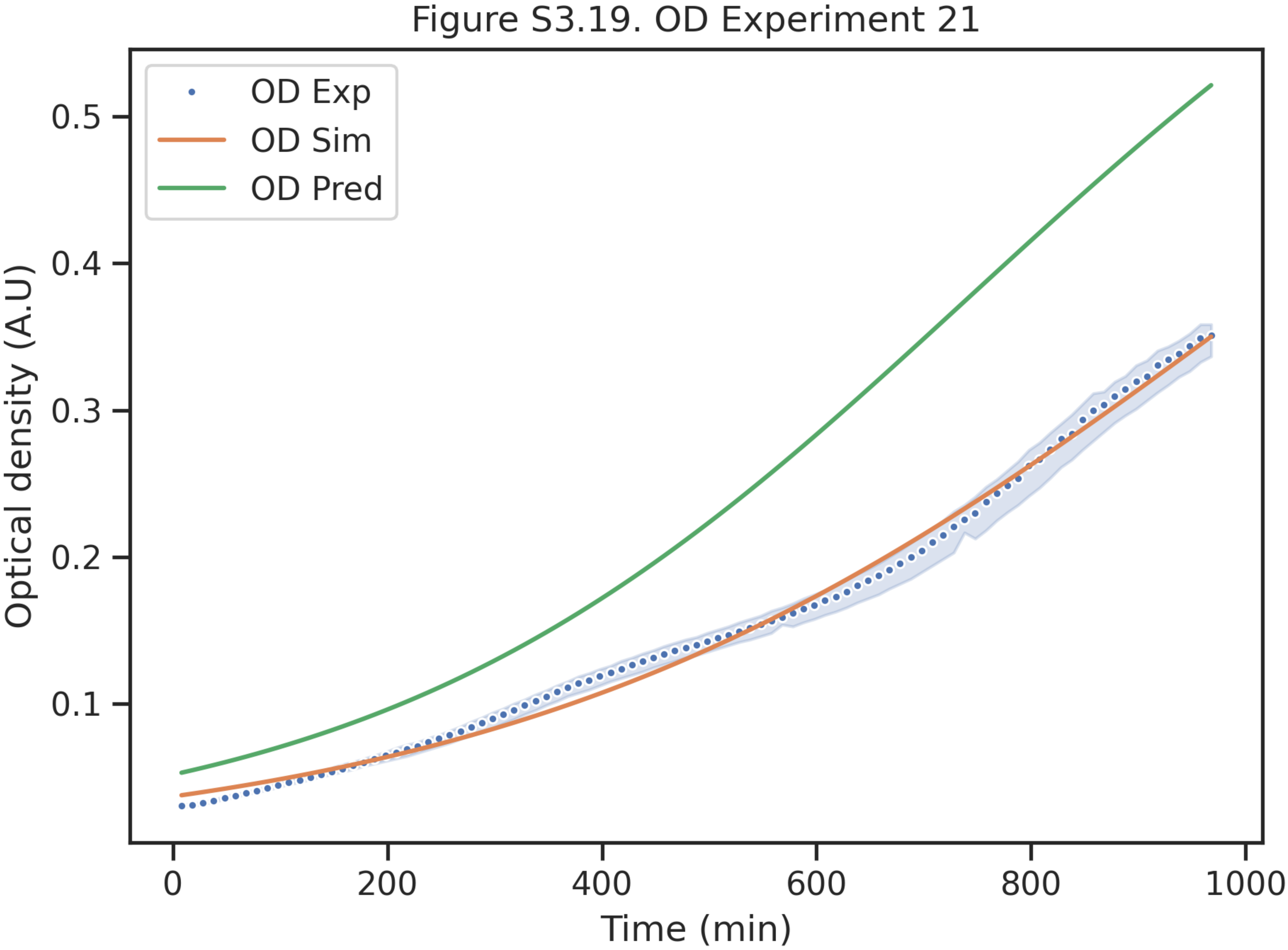

Figure S3.19. OD Experiment 21
OD Exp
OD Sim
OD Pred
Optical density (A.U)
0.1
0.2
0.3
0.4
0.5
0
200
400
600
800
1000
Time (min)

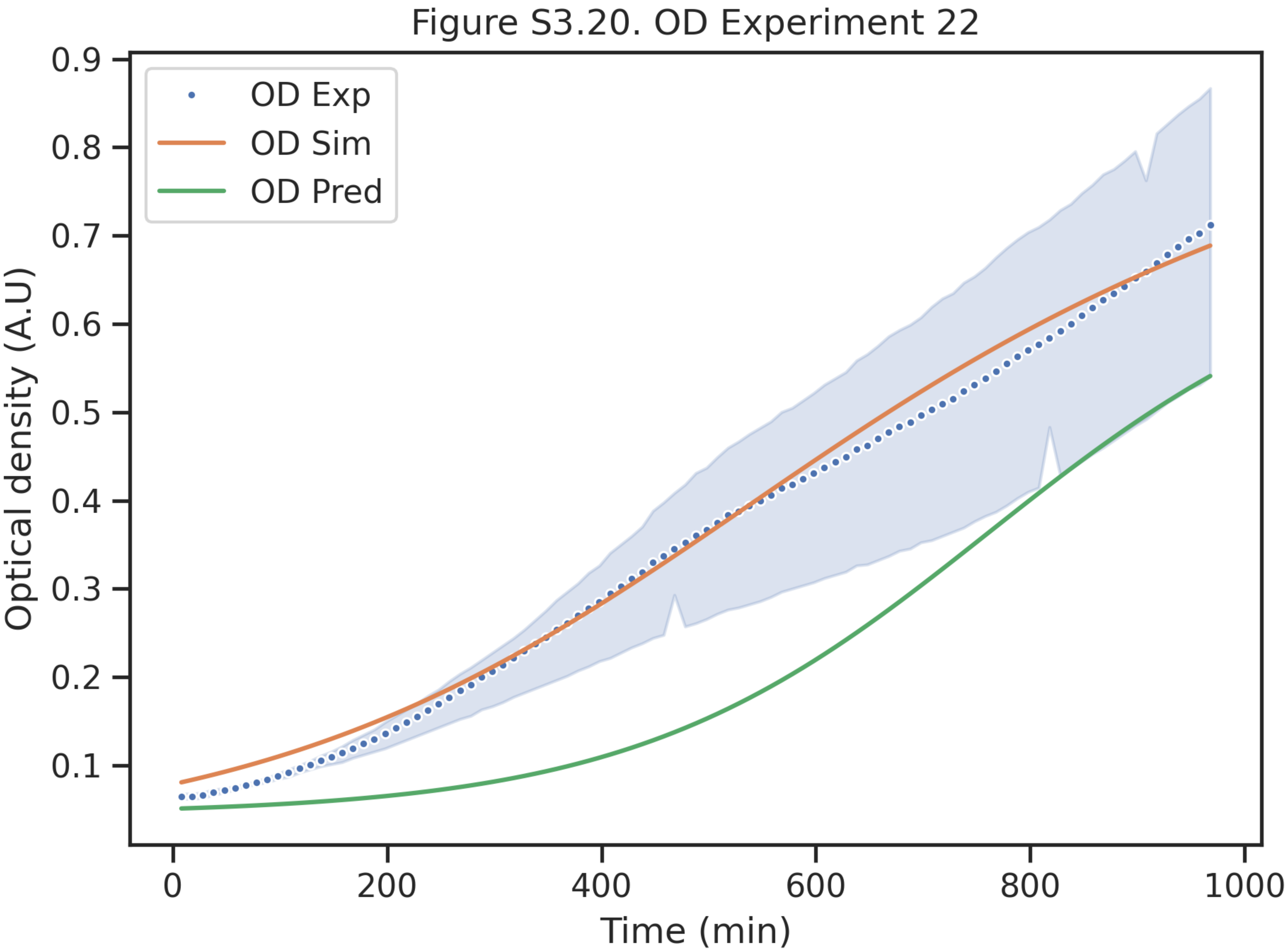

Figure S3.20. OD Experiment 22
OD Exp
OD Sim
OD Pred
Optical density (A.U)
0.1
0.2
0.3
0.4
0.5
0.6
0.7
0.8
0.9
0
200
400
600
800
1000
Time (min)

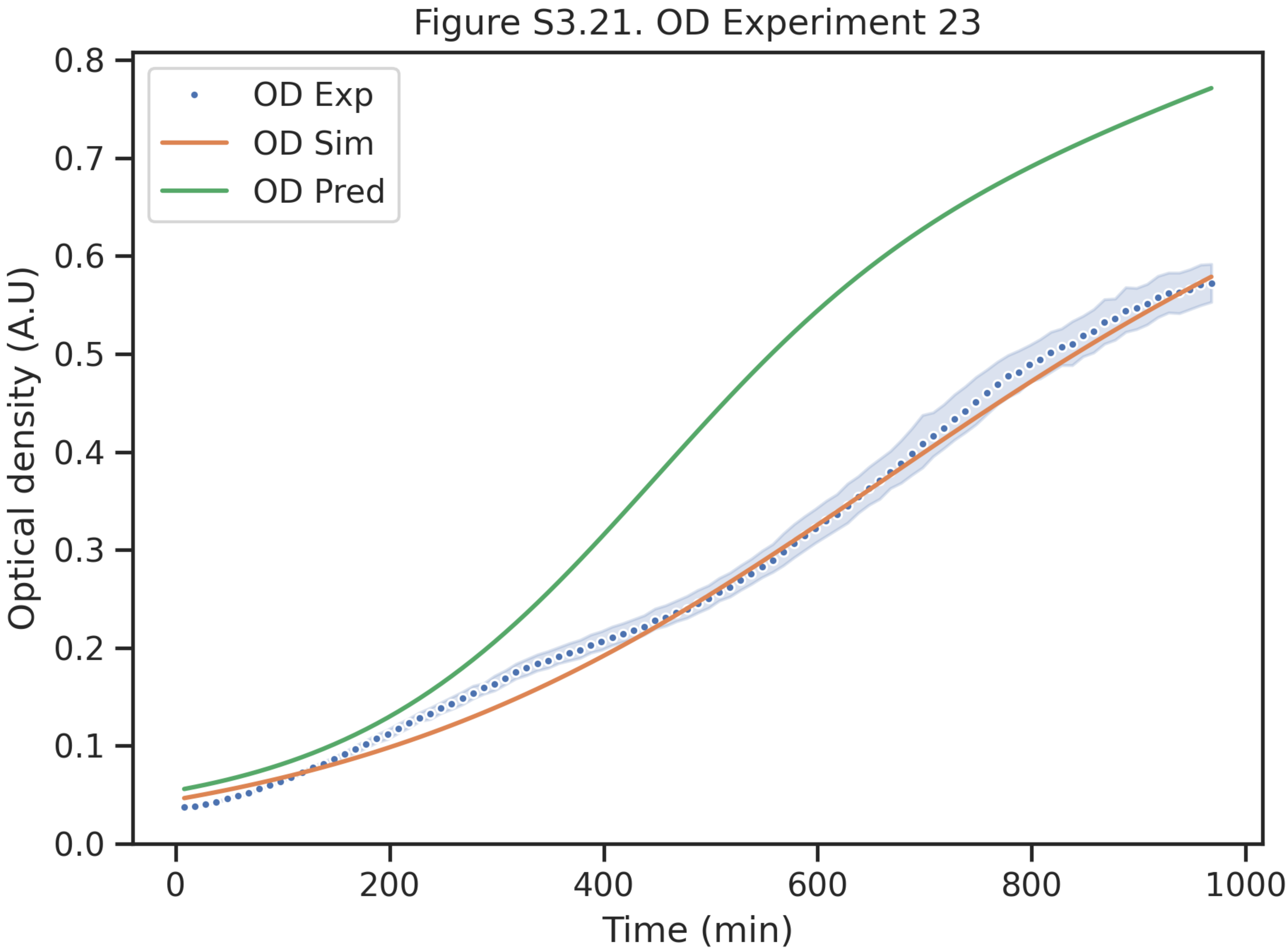

Figure S3.21. OD Experiment 23
OD Exp
OD Sim
OD Pred
Optical density (A.U)
0.0
0.1
0.2
0.3
0.4
0.5
0.6
0.7
0.8
0
200
400
600
800
1000
Time (min)

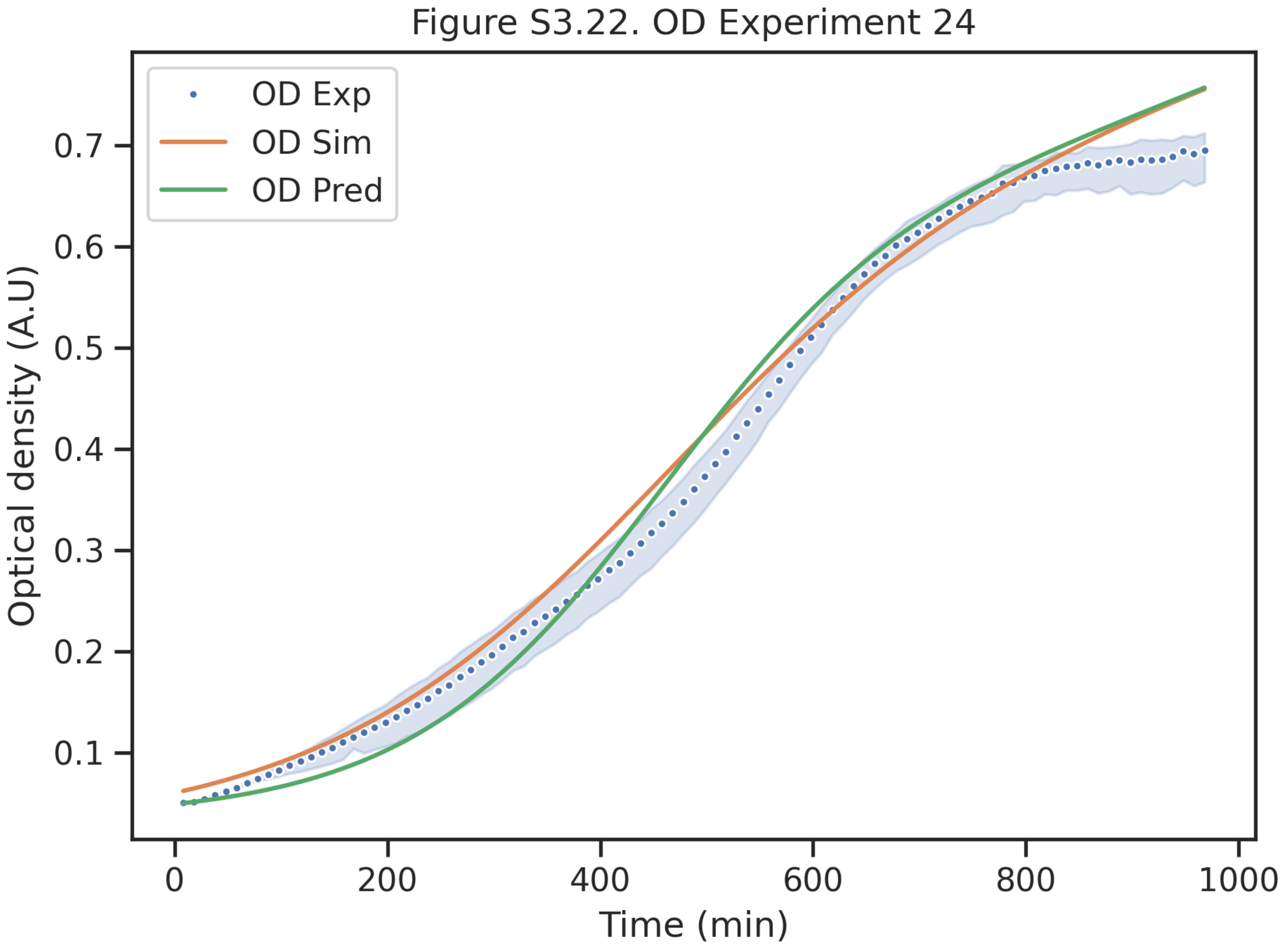
Figure S3.22. OD Experiment 24
OD Exp
OD Sim
OD Pred
Optical density (A.U)
0.1
0.2
0.3
0.4
0.5
0.6
0.7
0
200
400
600
800
1000
Time (min)

Figure S3.23. OD Experiment 25

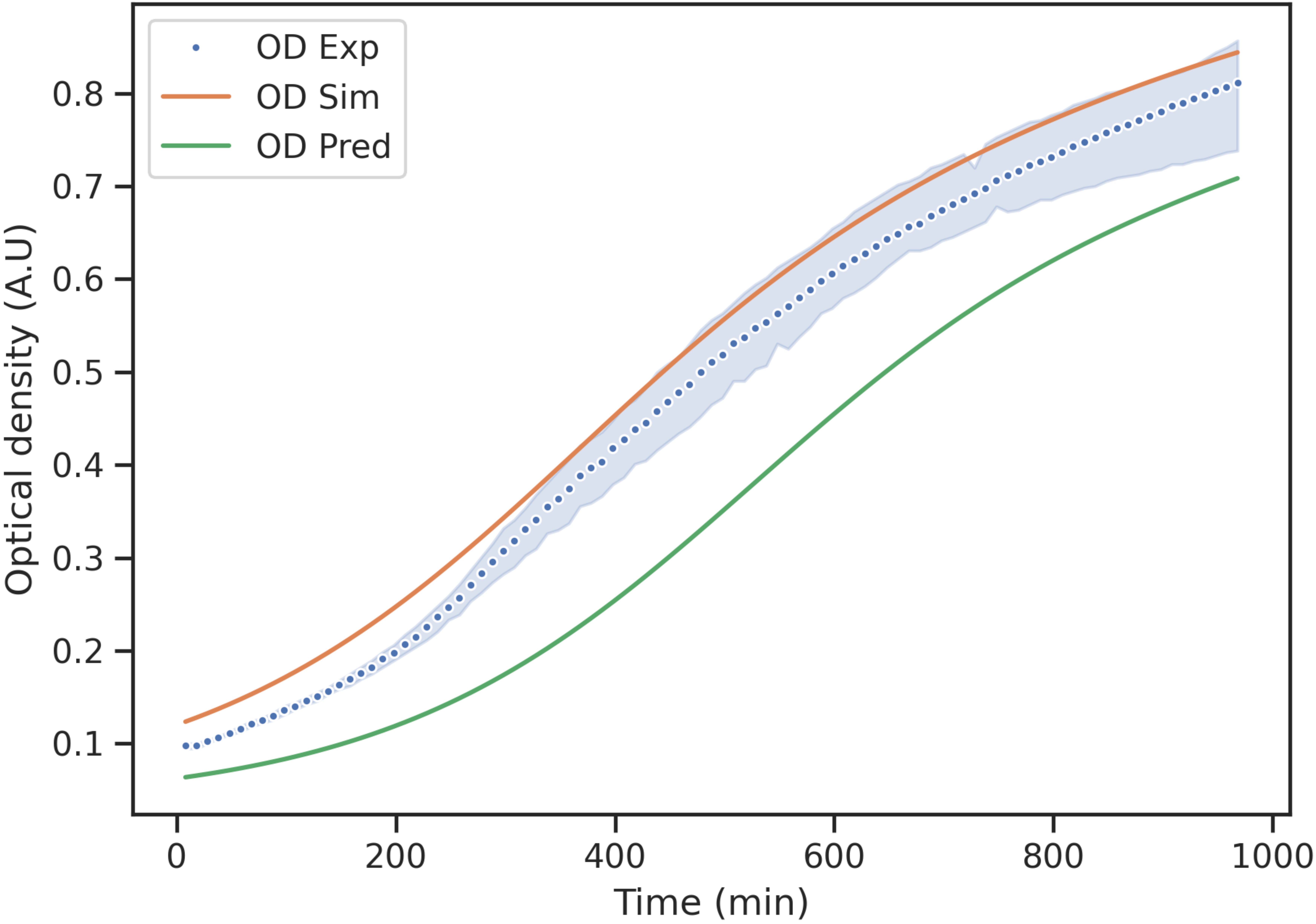

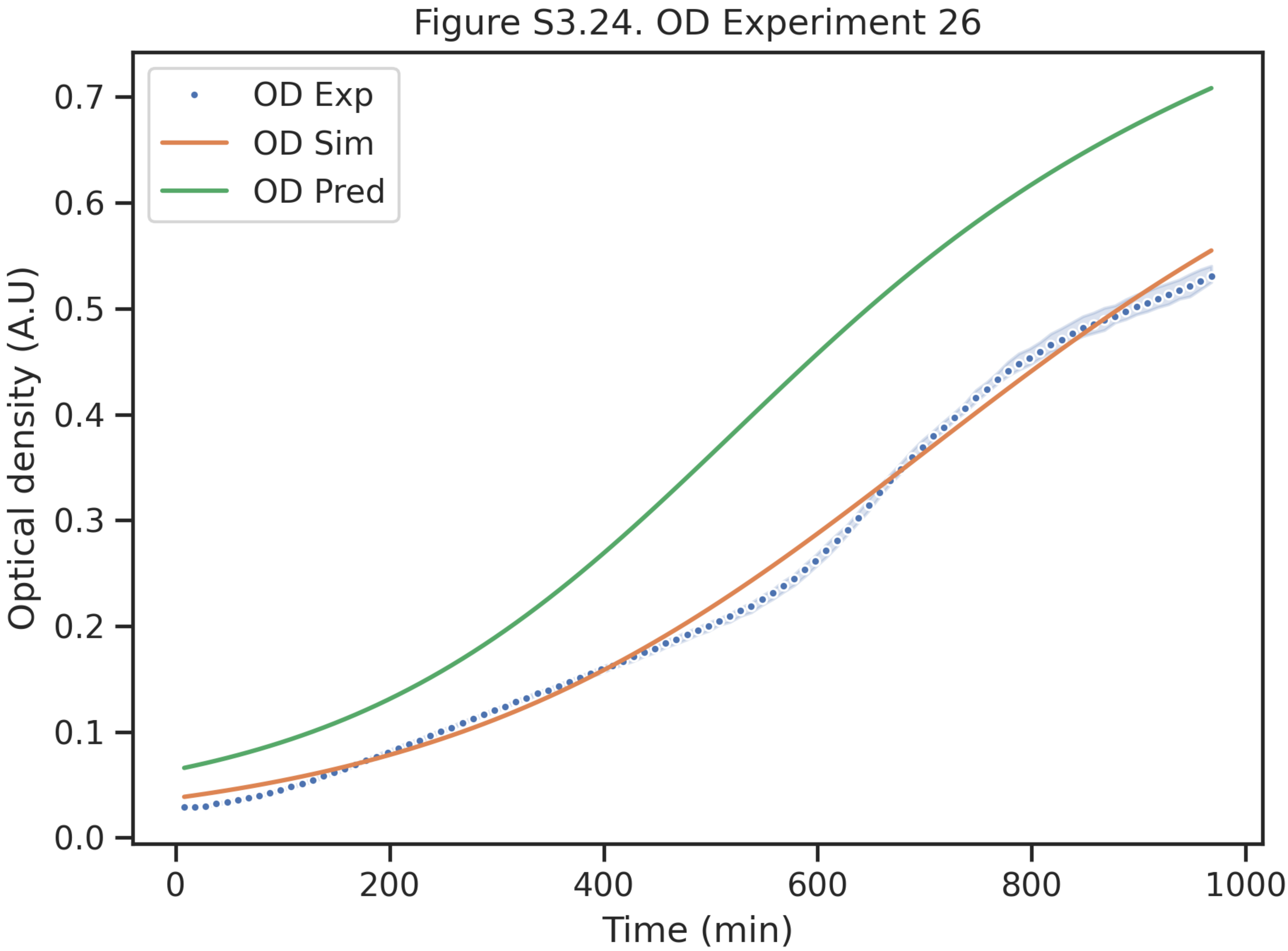
Figure S3.24. OD Experiment 26
OD Exp
OD Sim
OD Pred
Optical density (A.U)
Time (min)
0.0
0.1
0.2
0.3
0.4
0.5
0.6
0.7
0
200
400
600
800
1000

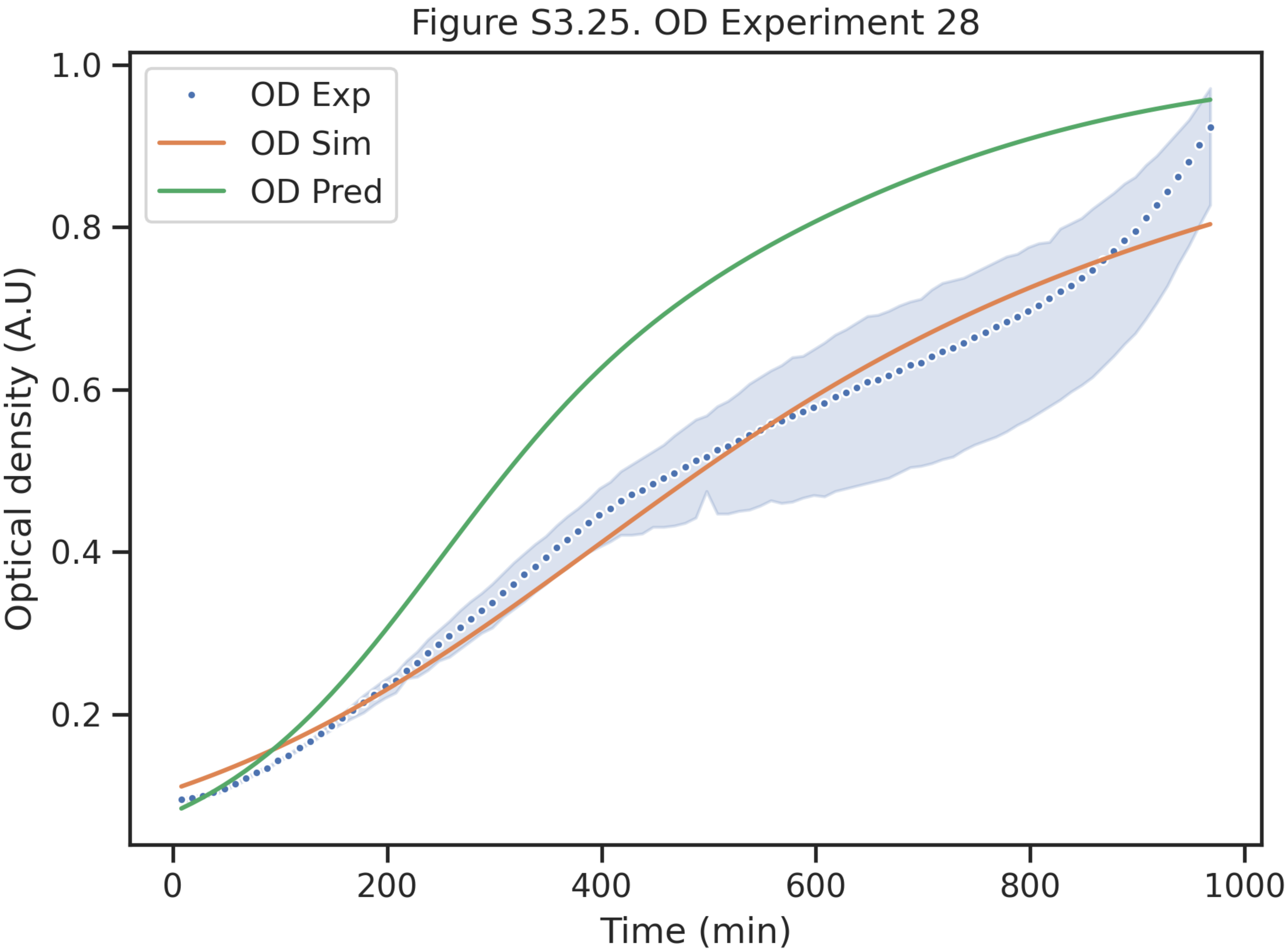
Figure S3.25. OD Experiment 28
OD Exp
OD Sim
OD Pred
Optical density (A.U)
Time (min)
1.0
0.8
0.6
0.4
0.2
0
200
400
600
800
1000

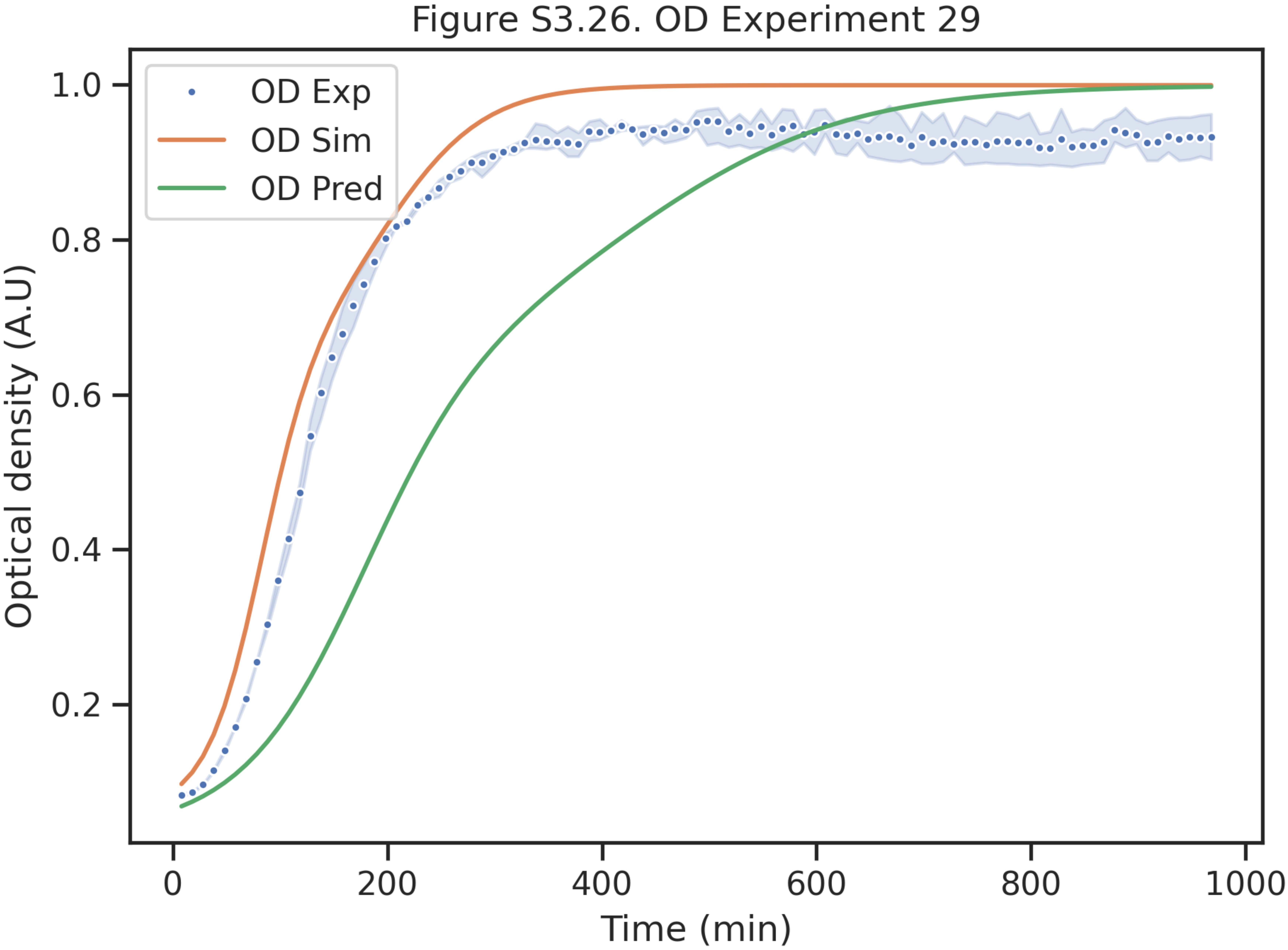
Figure S3.26. OD Experiment 29
OD Exp
OD Sim
OD Pred
Optical density (A.U)
Time (min)
0
200
400
600
800
1000
0.2
0.4
0.6
0.8
1.0

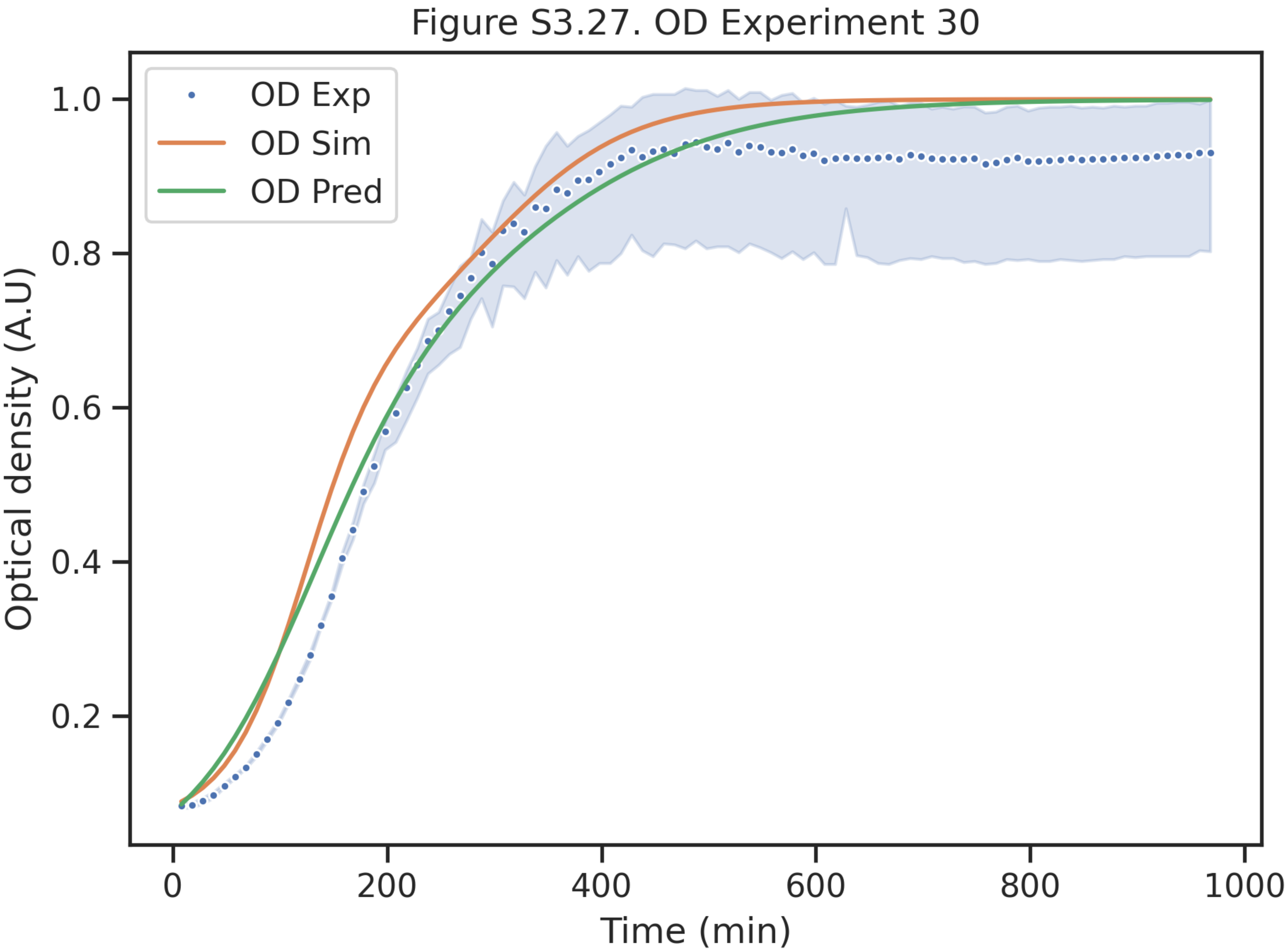

Figure S3.27. OD Experiment 30
OD Exp
OD Sim
OD Pred
1.0
0.8
0.6
0.4
0.2
Optical density (A.U)
0
200
400
600
800
1000
Time (min)

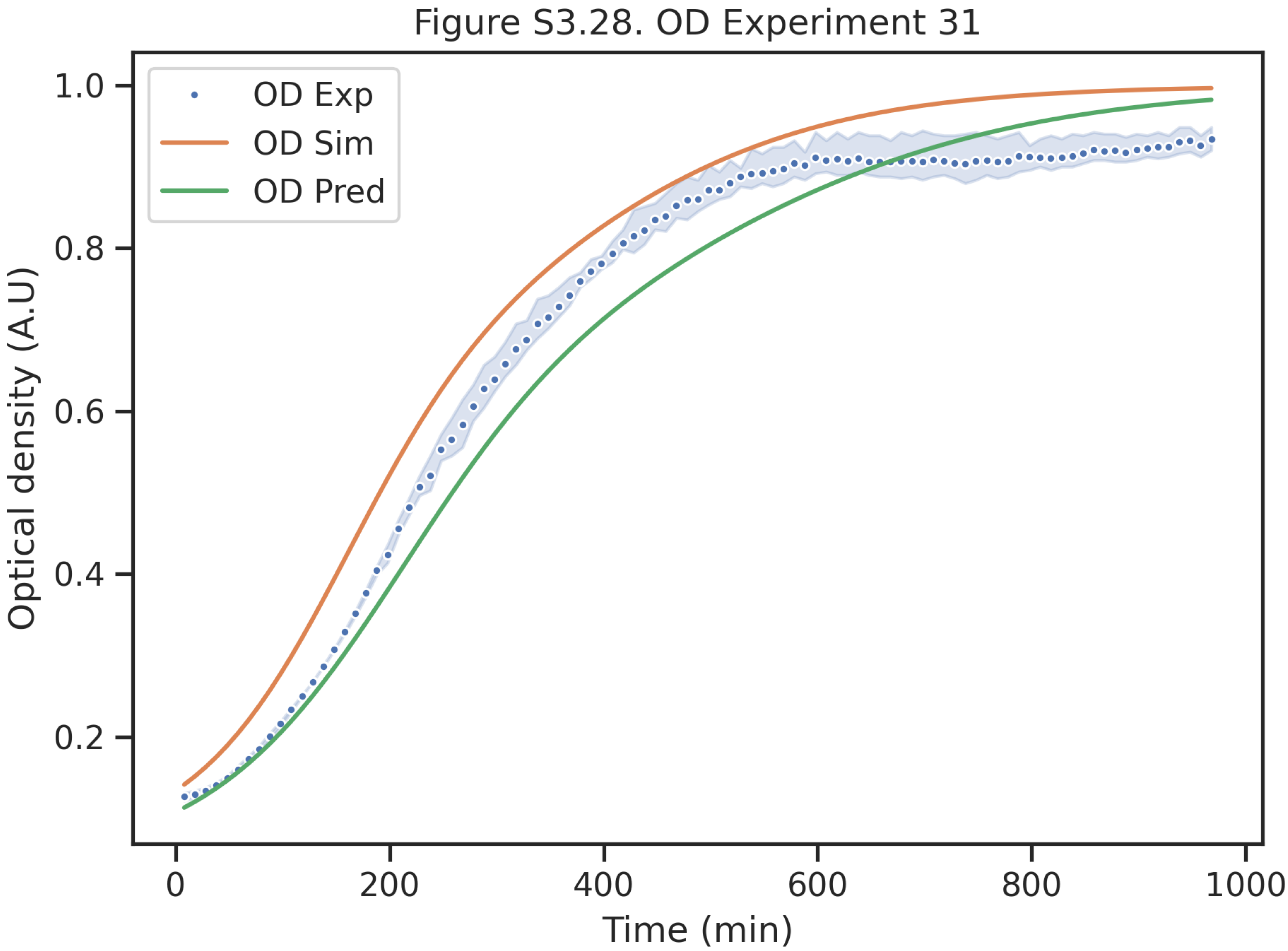
Figure S3.28. OD Experiment 31
OD Exp
OD Sim
OD Pred
Optical density (A.U)
Time (min)
0.2
0.4
0.6
0.8
1.0
0
200
400
600
800
1000

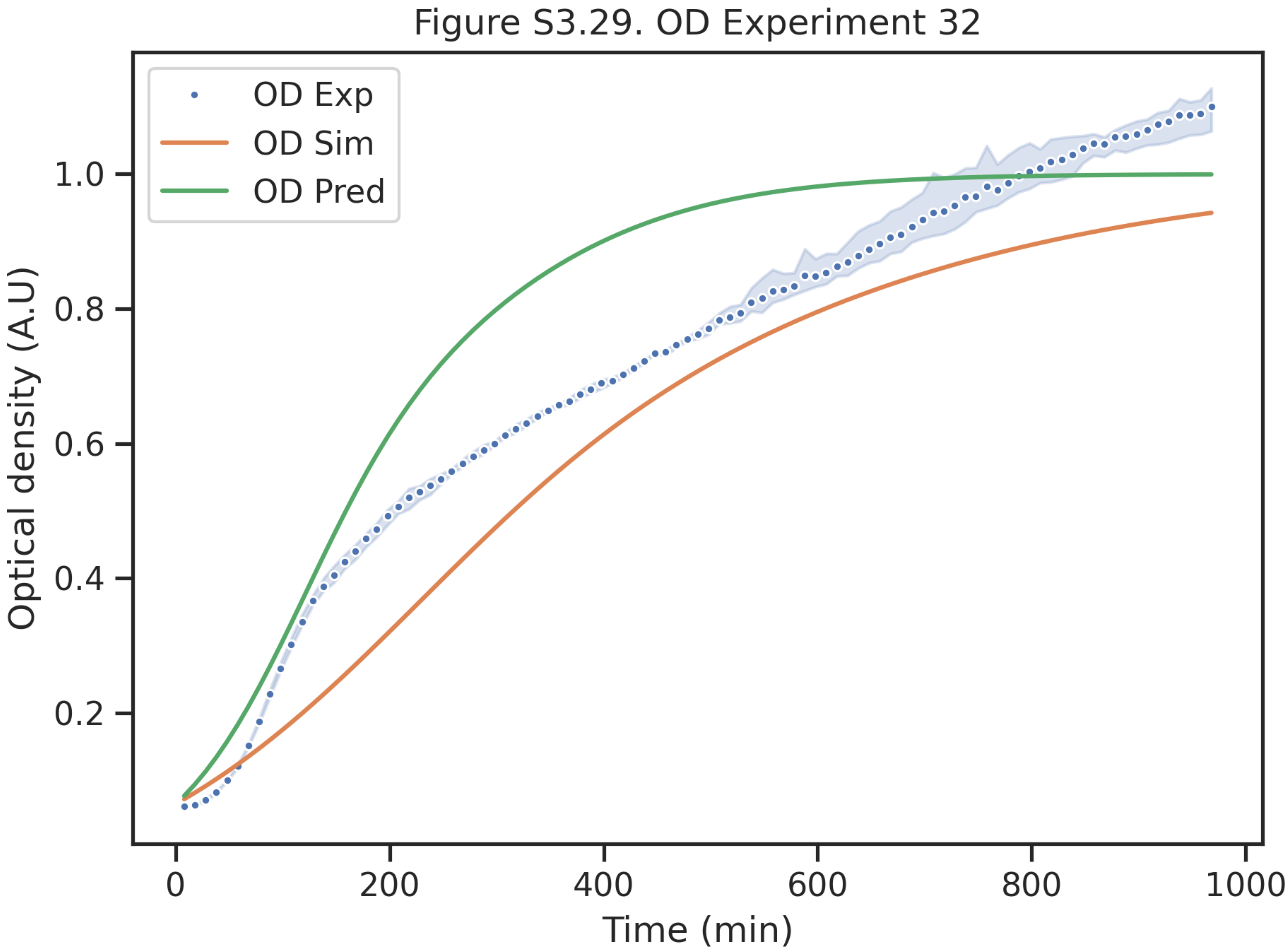
Figure S3.29. OD Experiment 32
OD Exp
OD Sim
OD Pred
1.0
0.8
0.6
0.4
0.2
Optical density (A.U)
0
200
400
600
800
1000
Time (min)

Supplementary Figure S4. Experimental (blue dotted) vs simulated (orange) vs predicted (green) growth curves of the biosensor, measured as ODs, for the initial 48 combinatorial library, using leave-one-out cross validation. Experiments 5 and 38 from the DoE library were removed from the training set due to assembly issues (resulting in a non-functional response), whereas experiments 6, 29, and 45 were kept because they were dynamically functional, although their readings were of lower intensity than the rest of the library due to potential experimental issues.

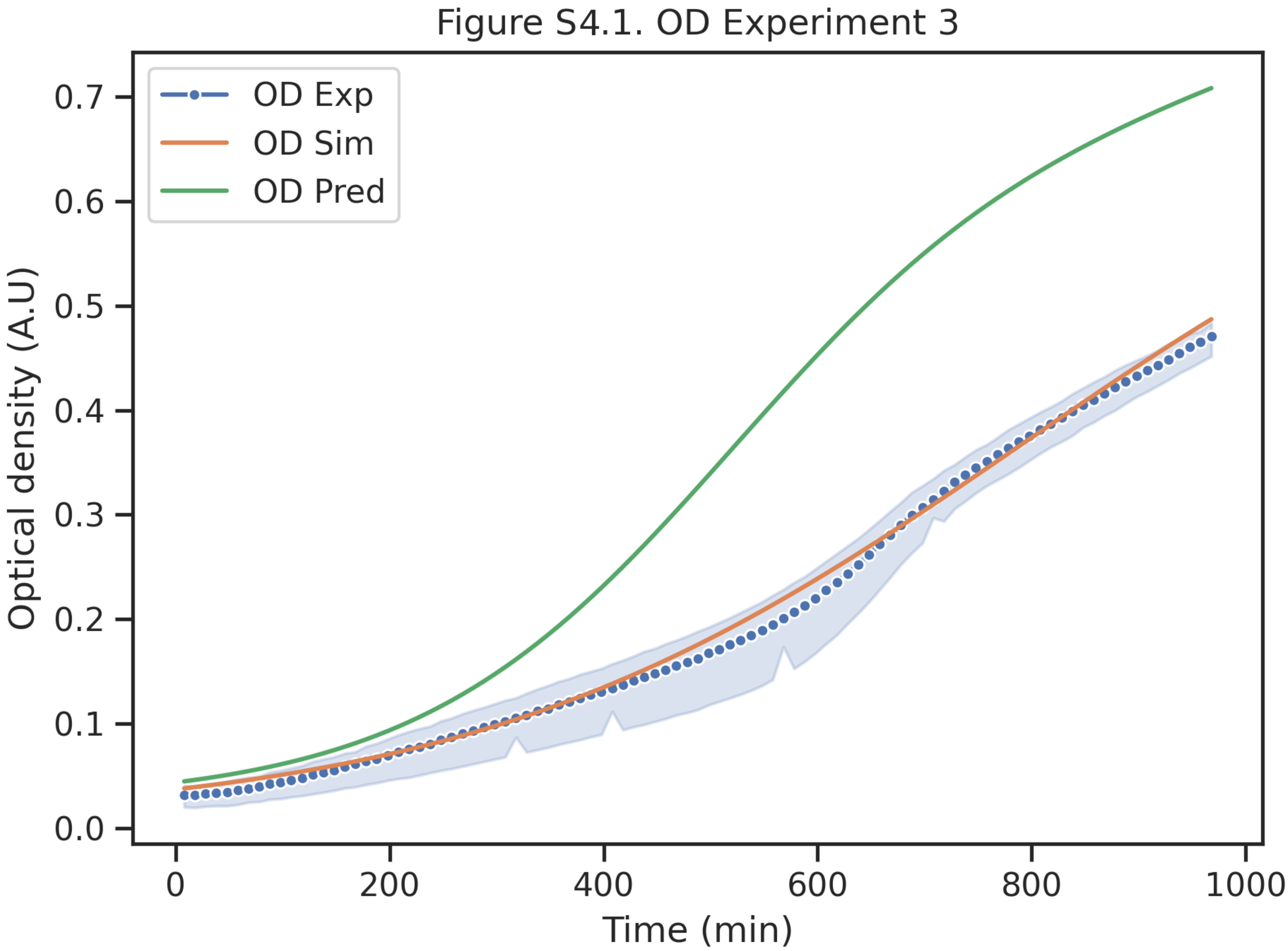
Figure S4.1. OD Experiment 3
OD Exp
OD Sim
OD Pred
0.7
0.6
0.5
0.4
0.3
0.2
0.1
0.0
Optical density (A.U)
0
200
400
600
800
1000
Time (min)

Figure S4.2. OD Experiment 4

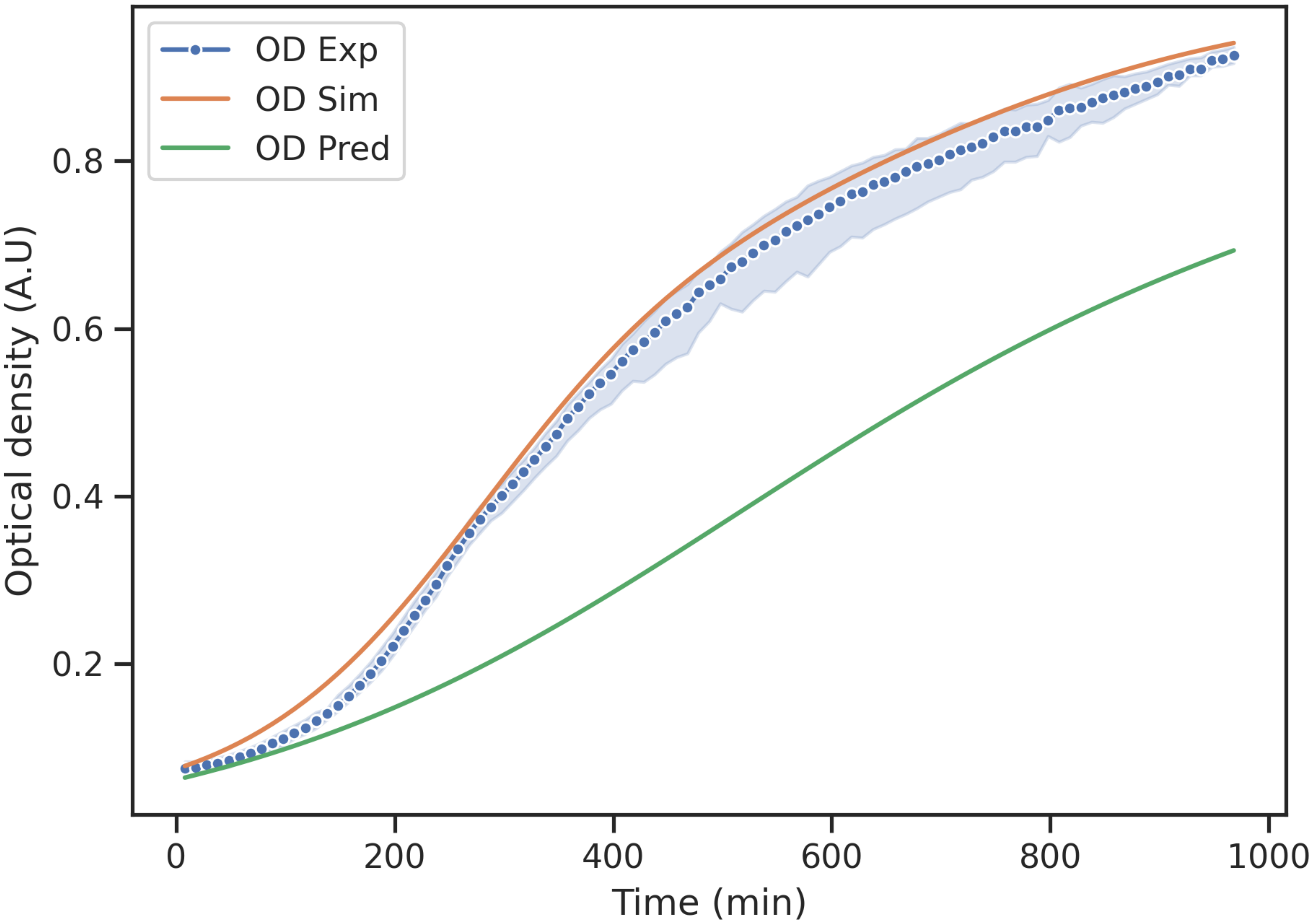

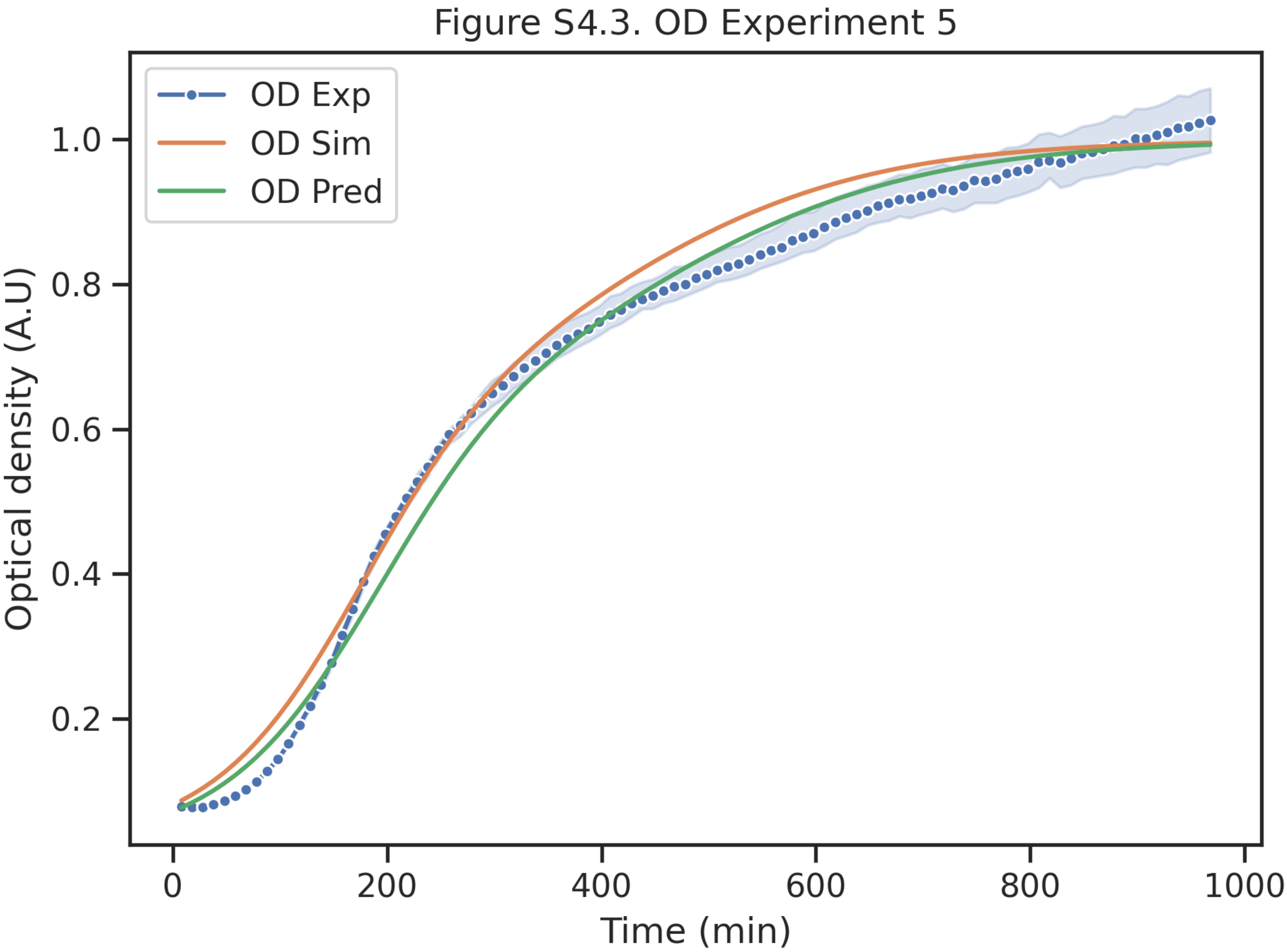
Figure S4.3. OD Experiment 5
OD Exp
OD Sim
OD Pred
1.0
0.8
0.6
0.4
0.2
Optical density (A.U)
0
200
400
600
800
1000
Time (min)

Figure S4.4. OD Experiment 6

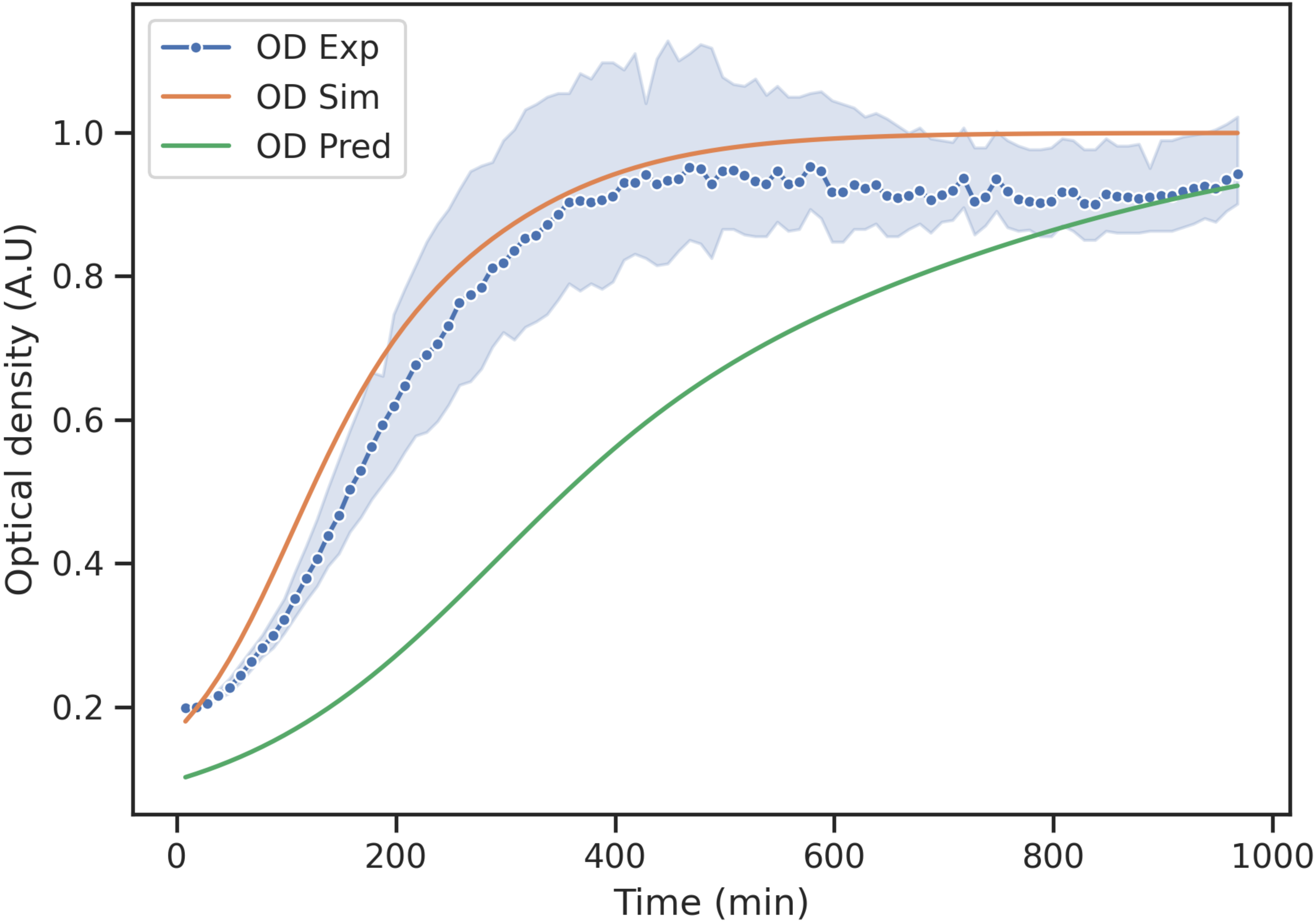

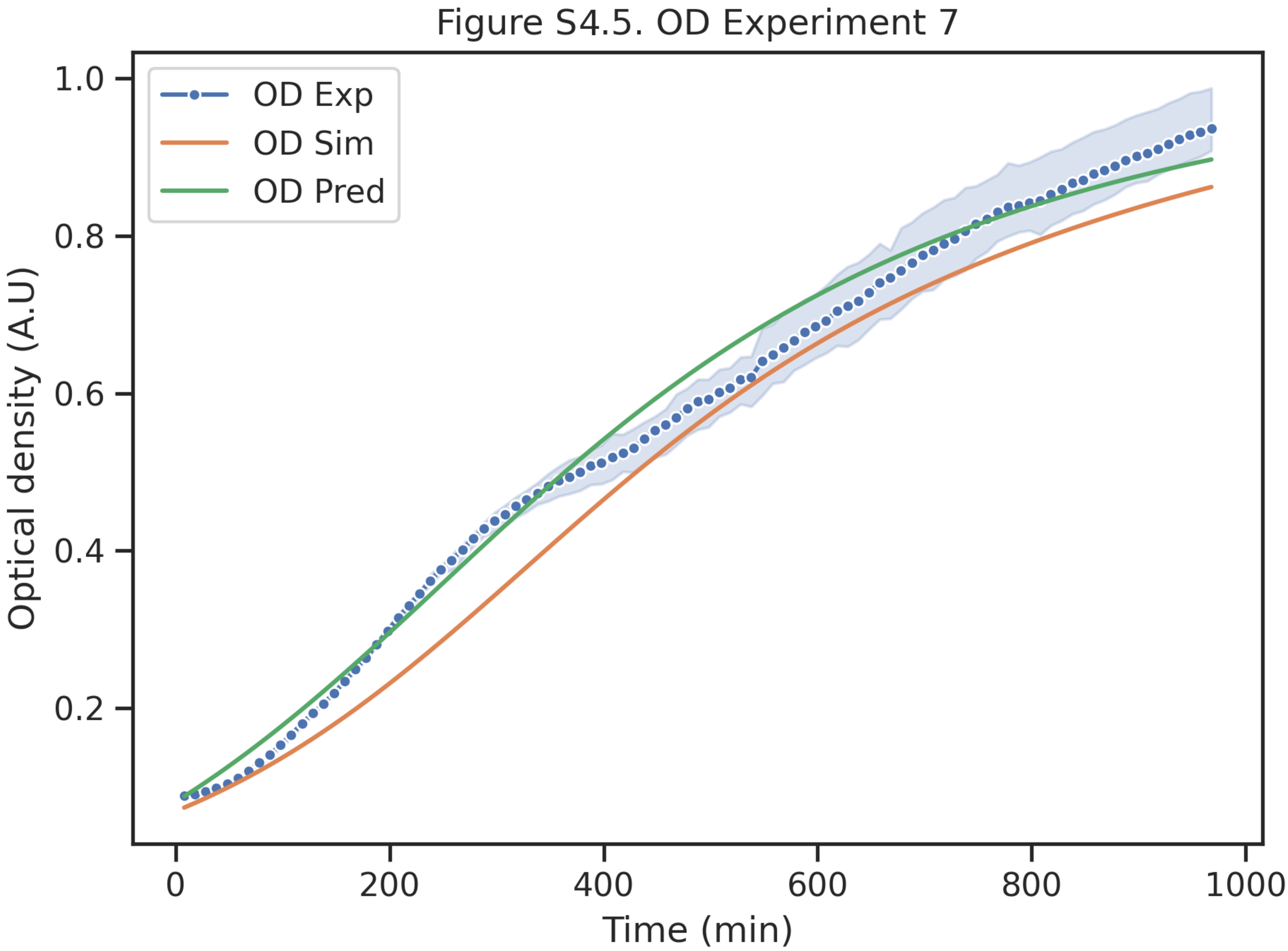
Figure S4.5. OD Experiment 7
OD Exp
OD Sim
OD Pred
Optical density (A.U)
Time (min)
0.2
0.4
0.6
0.8
1.0
0
200
400
600
800
1000

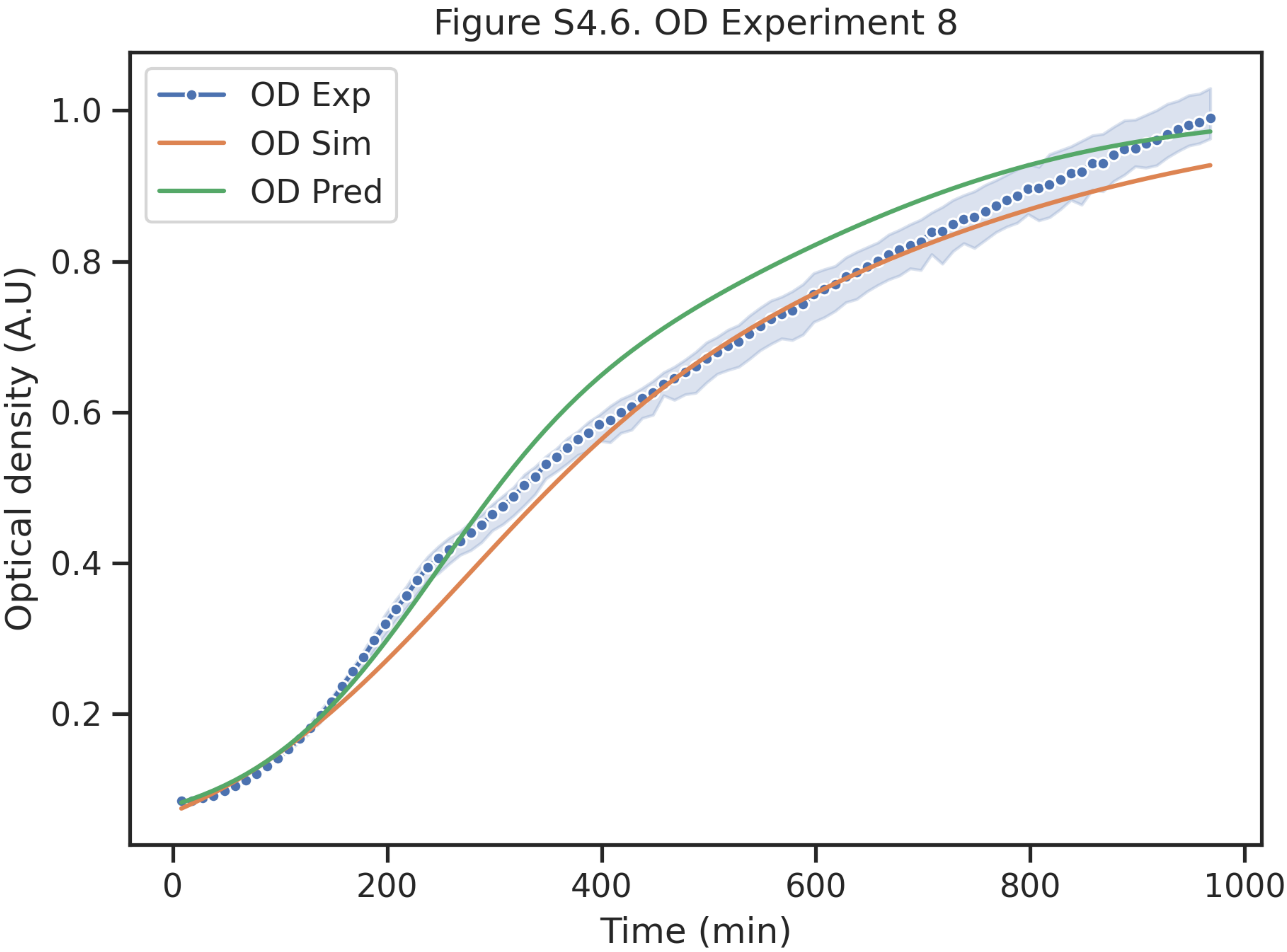
Figure S4.6. OD Experiment 8
OD Exp
OD Sim
OD Pred
1.0
0.8
0.6
0.4
0.2
Optical density (A.U)
0
200
400
600
800
1000
Time (min)

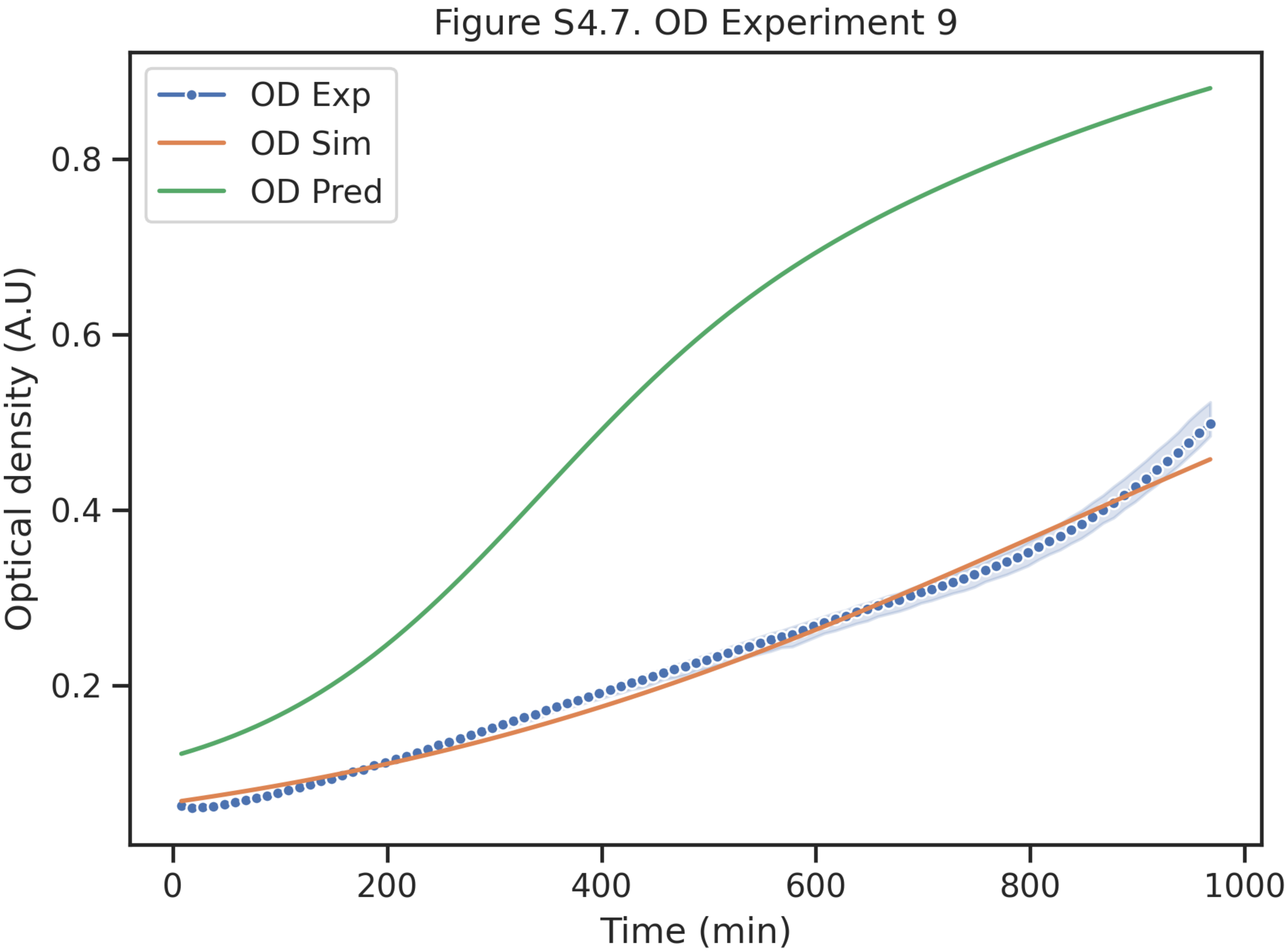
Figure S4.7. OD Experiment 9
OD Exp
OD Sim
OD Pred
Optical density (A.U)
0.2
0.4
0.6
0.8
0
200
400
600
800
1000
Time (min)

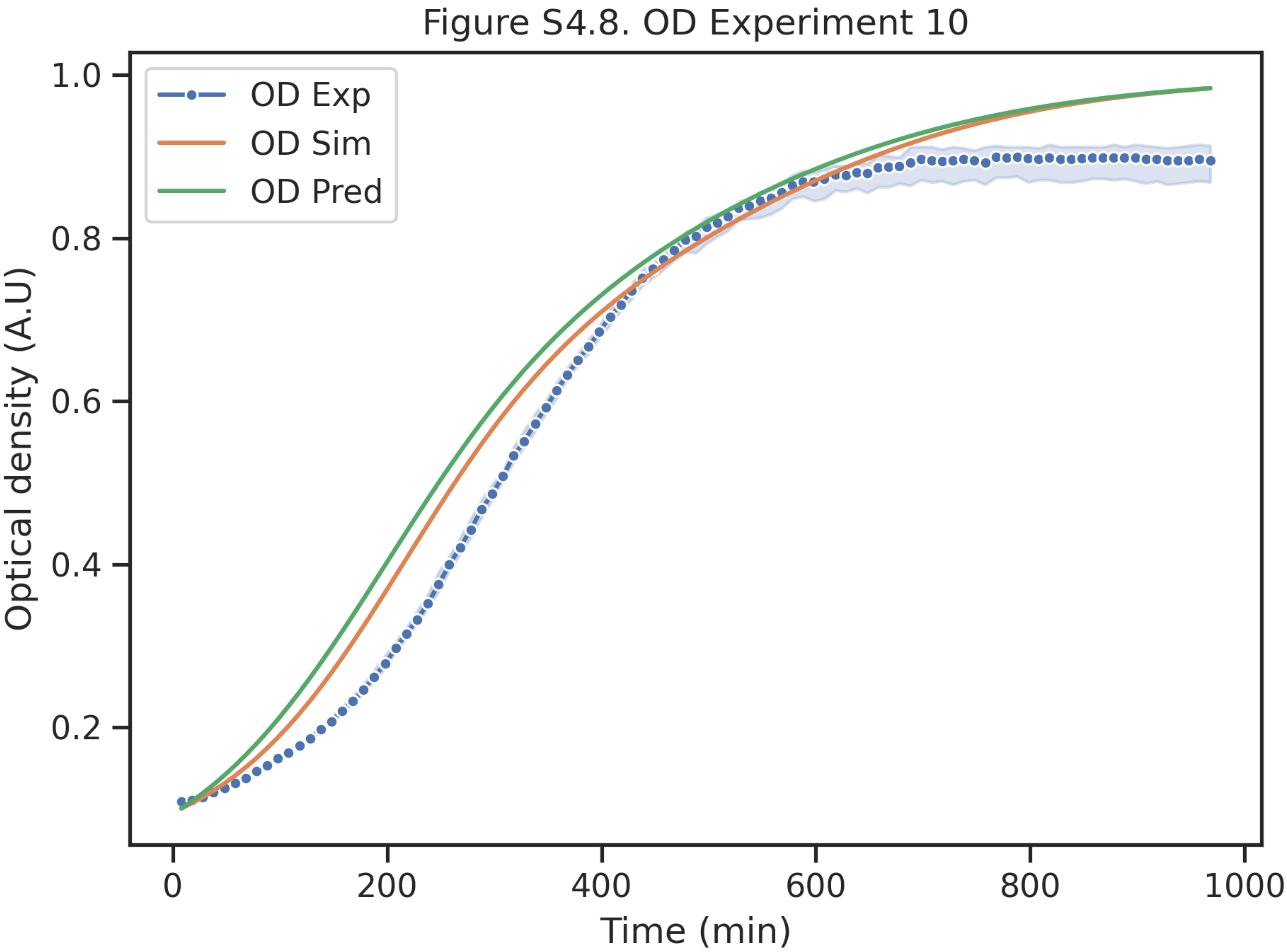
Figure S4.8. OD Experiment 10
OD Exp
OD Sim
OD Pred
Optical density (A.U)
1.0
0.8
0.6
0.4
0.2
0
200
400
600
800
1000
Time (min)

Figure S4.9. OD Experiment 11

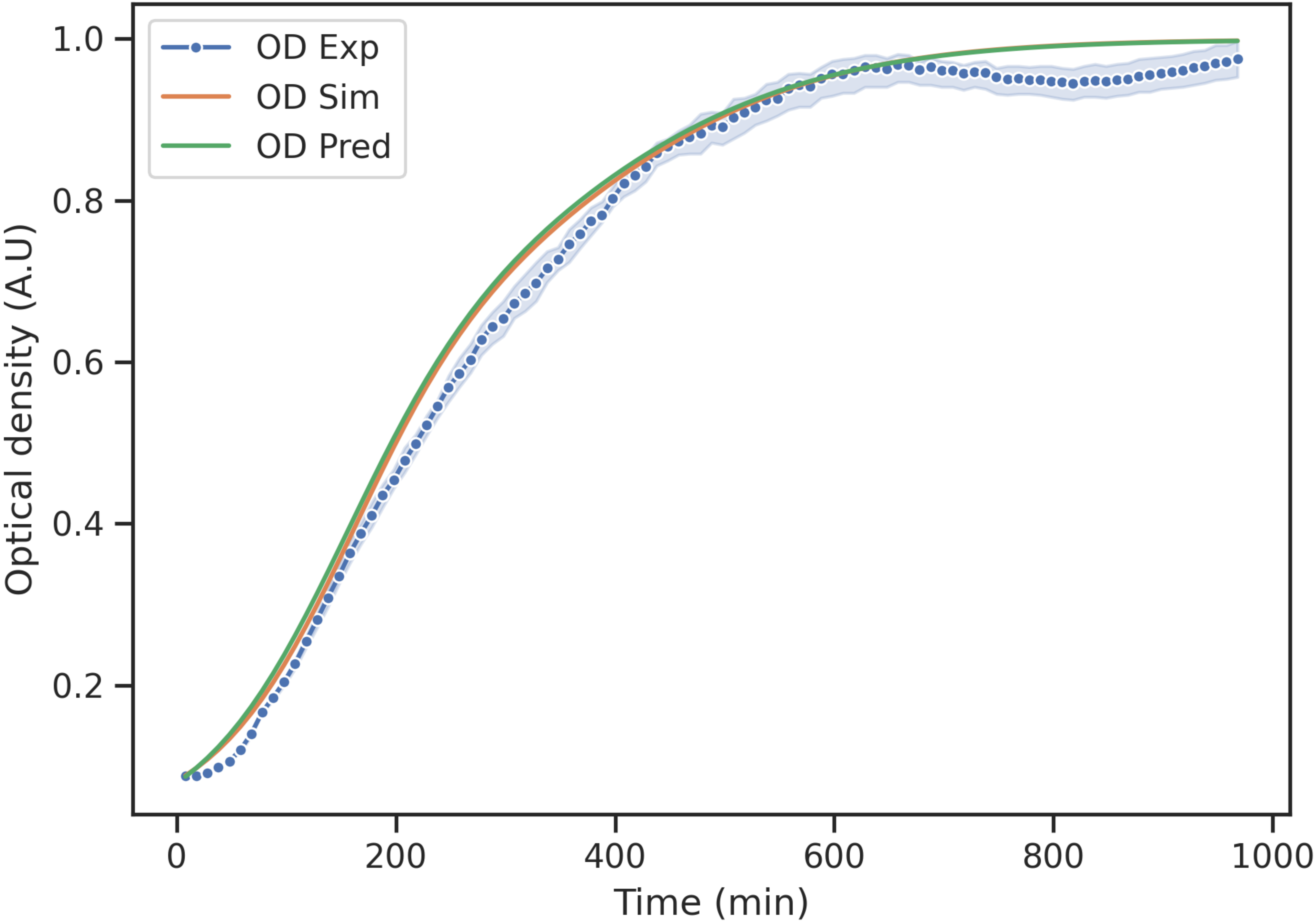

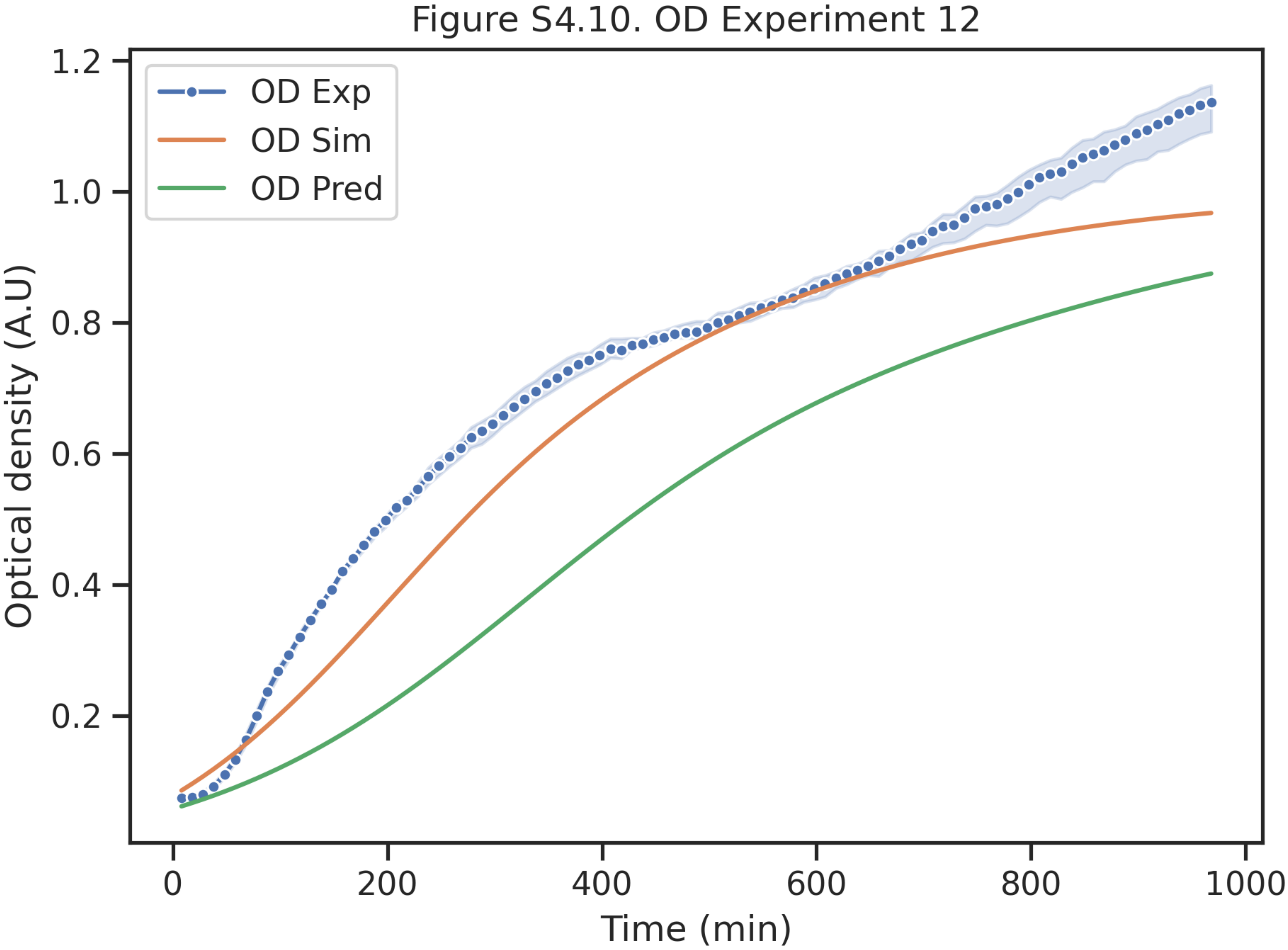

Figure S4.10. OD Experiment 12
OD Exp
OD Sim
OD Pred
Optical density (A.U)
Time (min)
1.2
1.0
0.8
0.6
0.4
0.2
0
200
400
600
800
1000

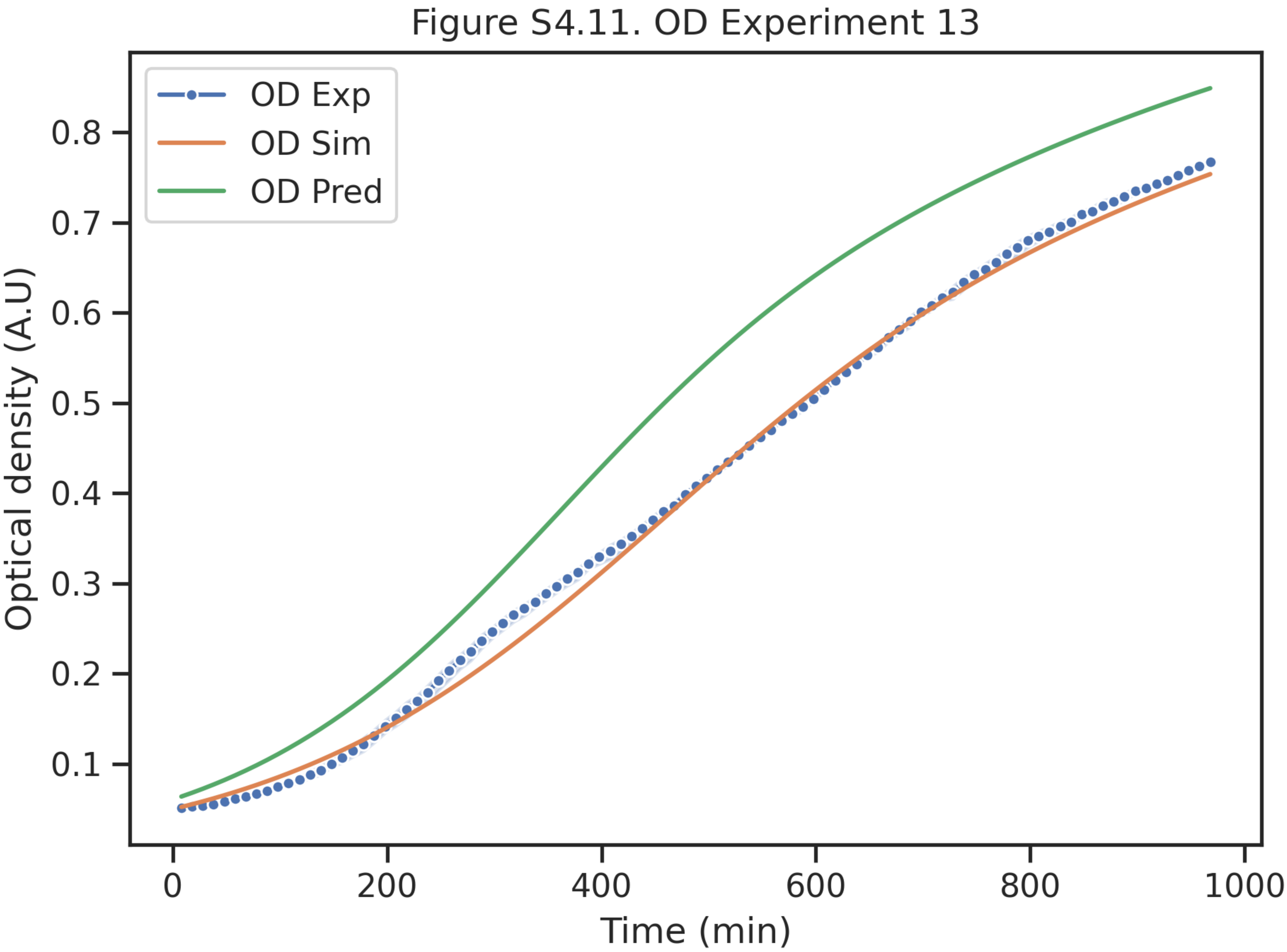

Figure S4.11. OD Experiment 13
OD Exp
OD Sim
OD Pred
Optical density (A.U)
0.1
0.2
0.3
0.4
0.5
0.6
0.7
0.8
0
200
400
600
800
1000
Time (min)

Figure S4.12. OD Experiment 14

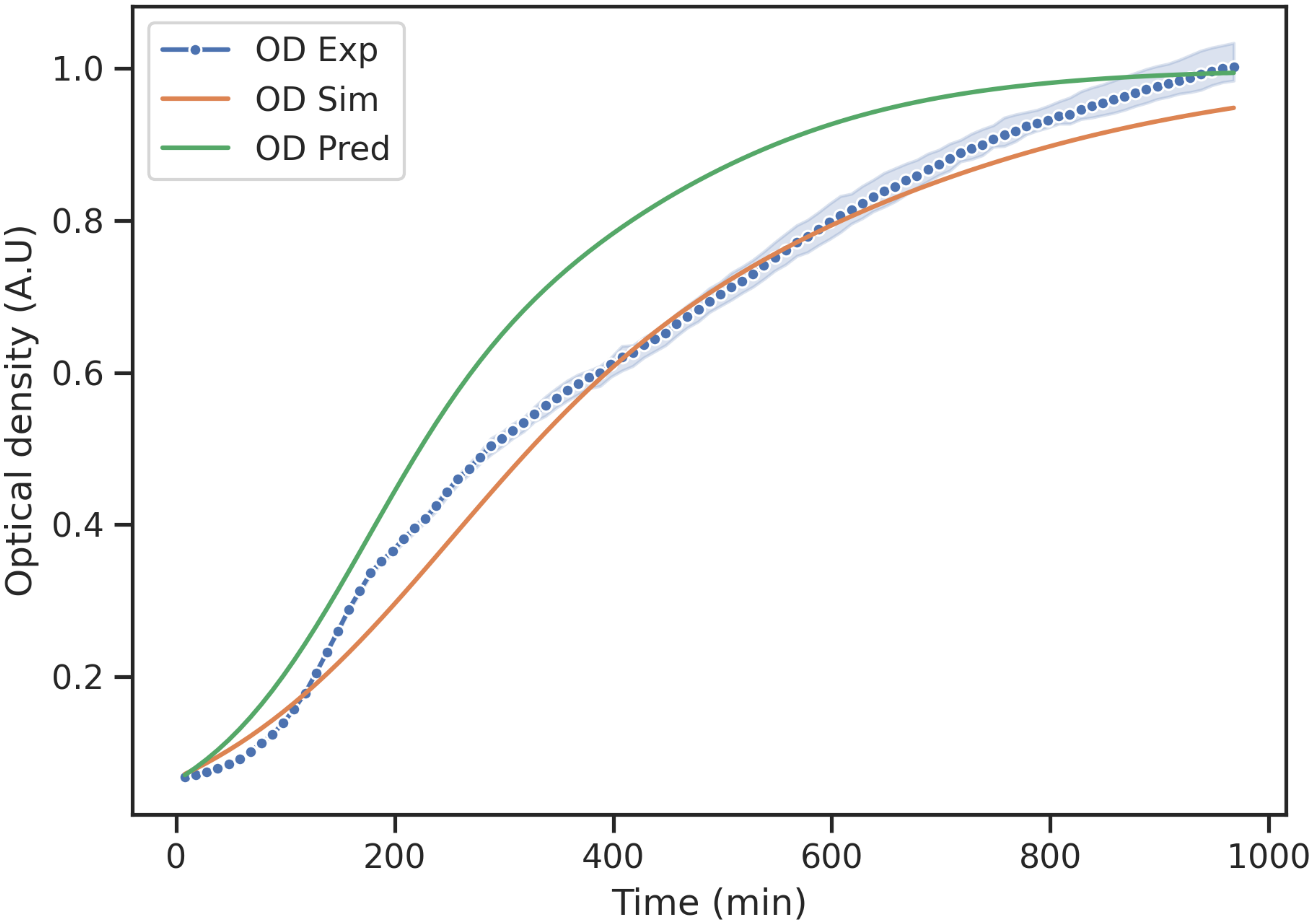

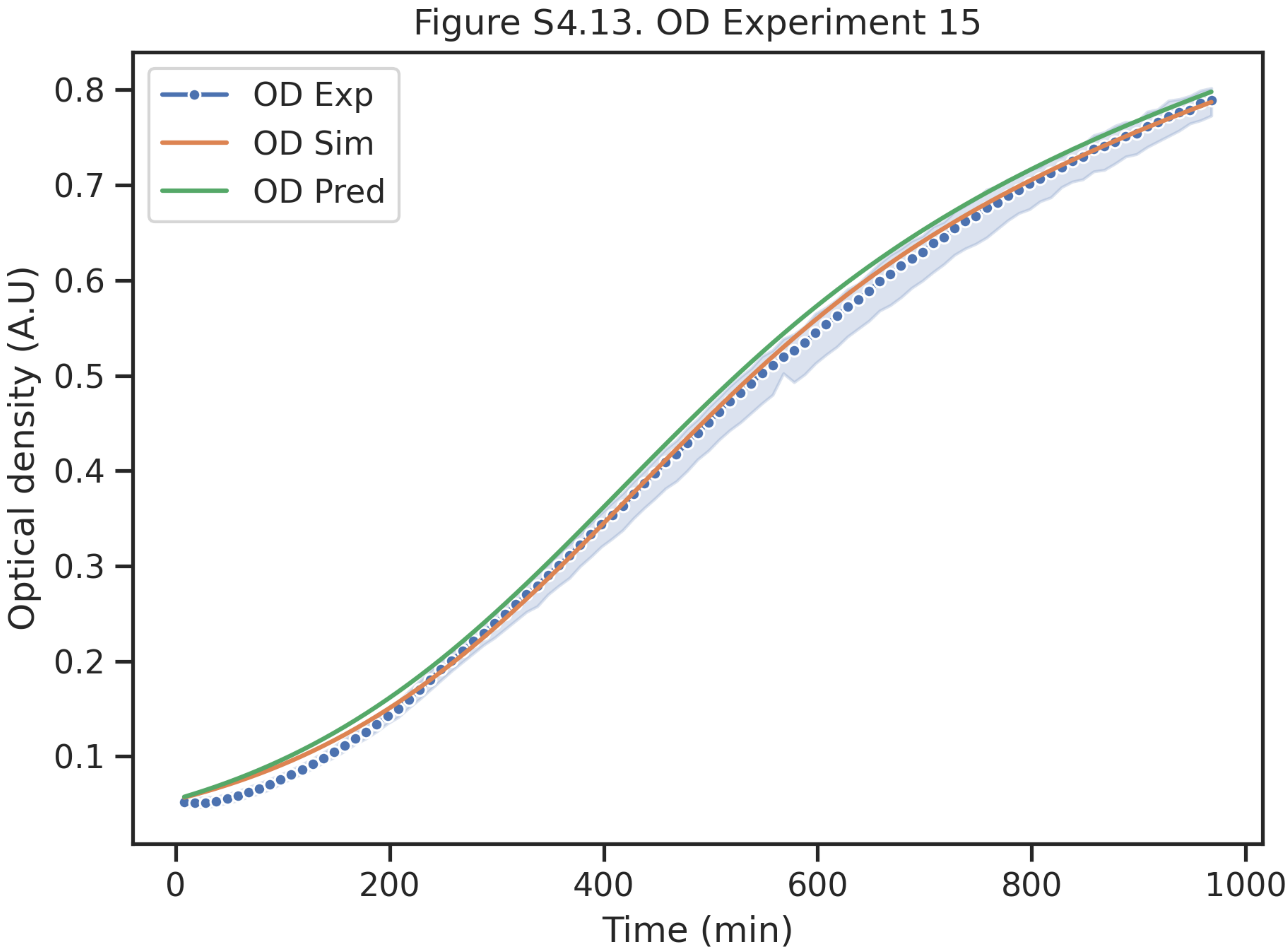
Figure S4.13. OD Experiment 15
OD Exp
OD Sim
OD Pred
Optical density (A.U)
Time (min)
0.1
0.2
0.3
0.4
0.5
0.6
0.7
0.8
0
200
400
600
800
1000

Figure S4.14. OD Experiment 16

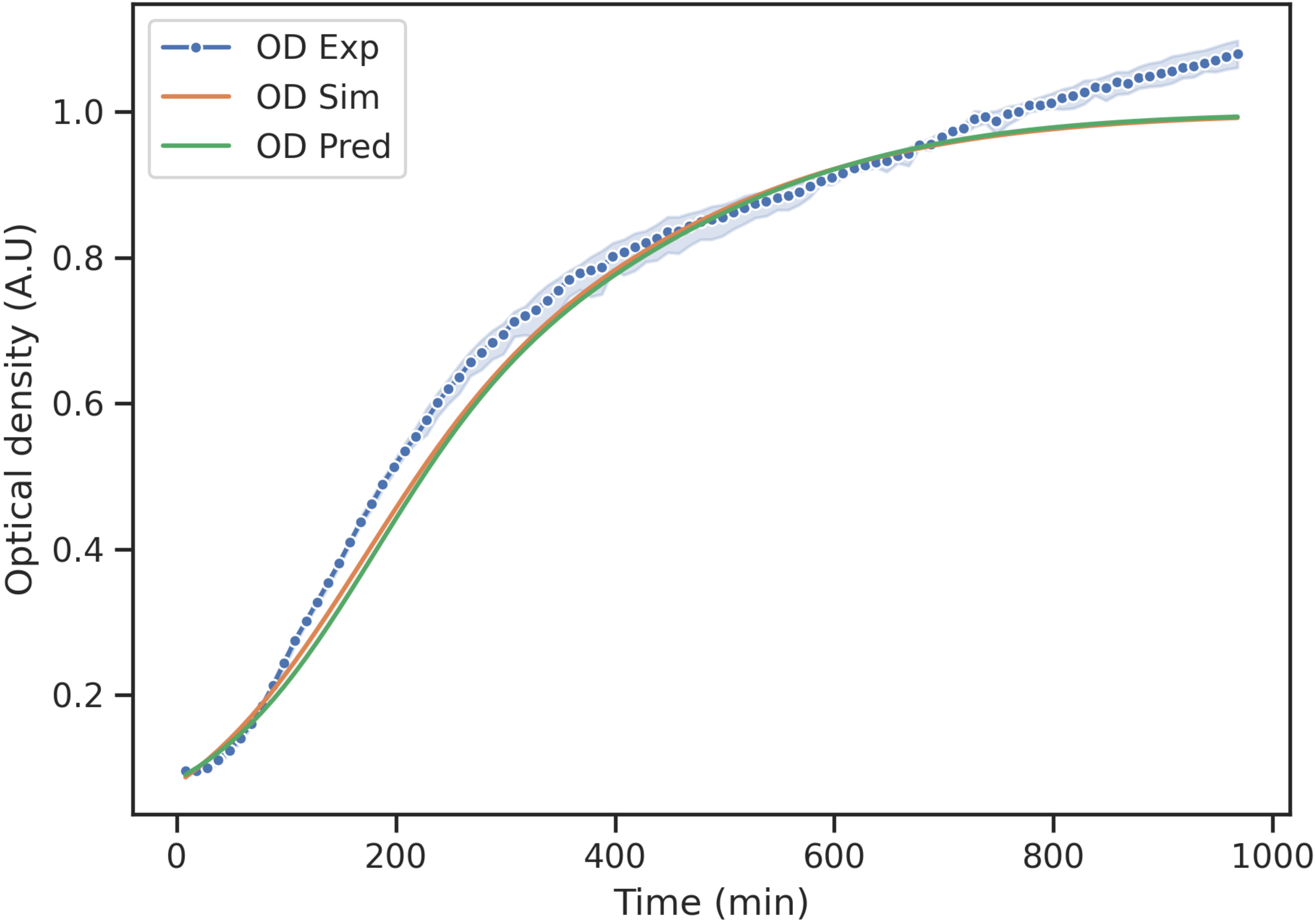

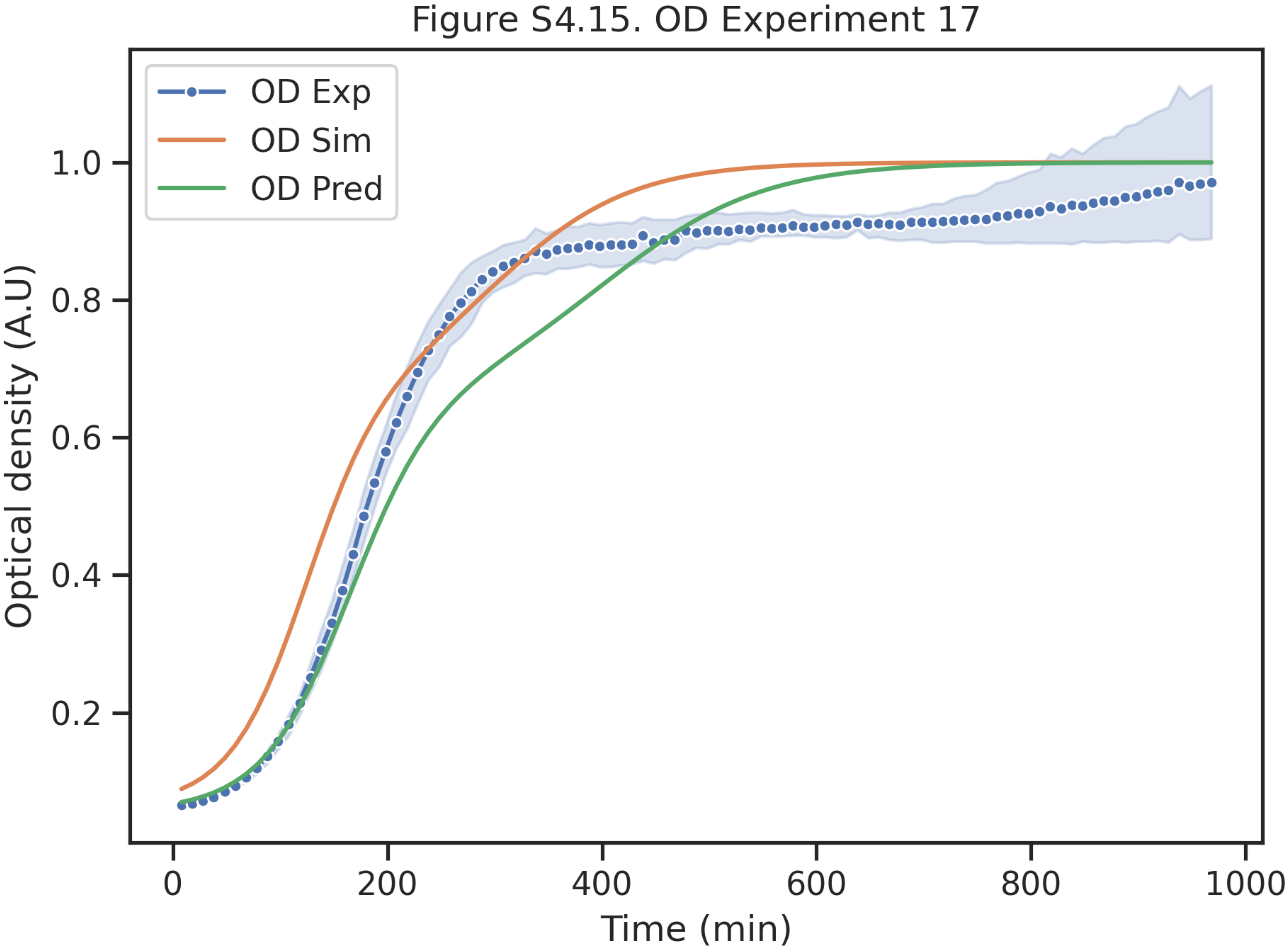
Figure S4.15. OD Experiment 17
OD Exp
OD Sim
OD Pred
Optical density (A.U)
1.0
0.8
0.6
0.4
0.2
0
200
400
600
800
1000
Time (min)

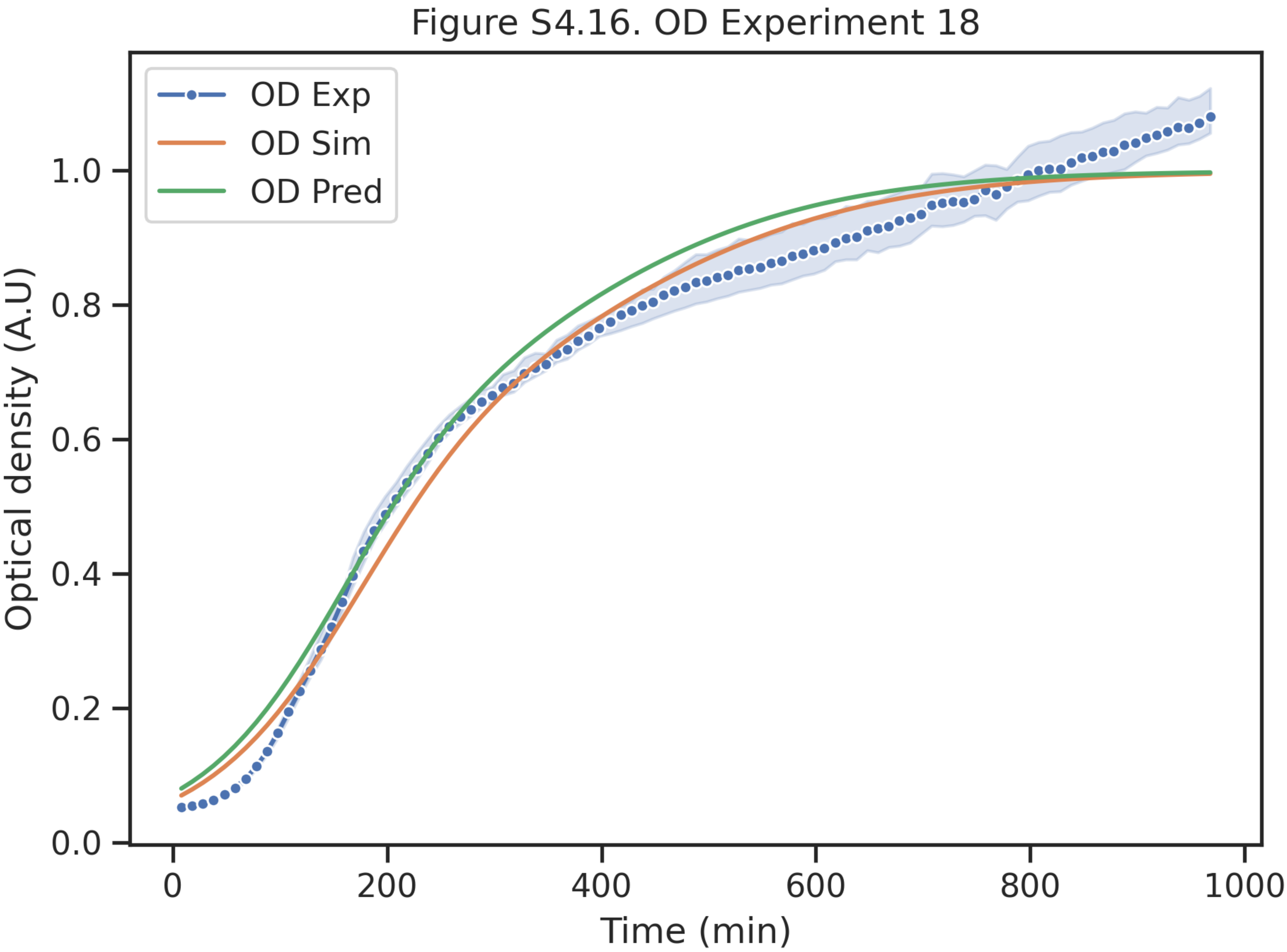

Figure S4.16. OD Experiment 18
OD Exp
OD Sim
OD Pred
Optical density (A.U)
Time (min)
0.0
0.2
0.4
0.6
0.8
1.0
0
200
400
600
800
1000

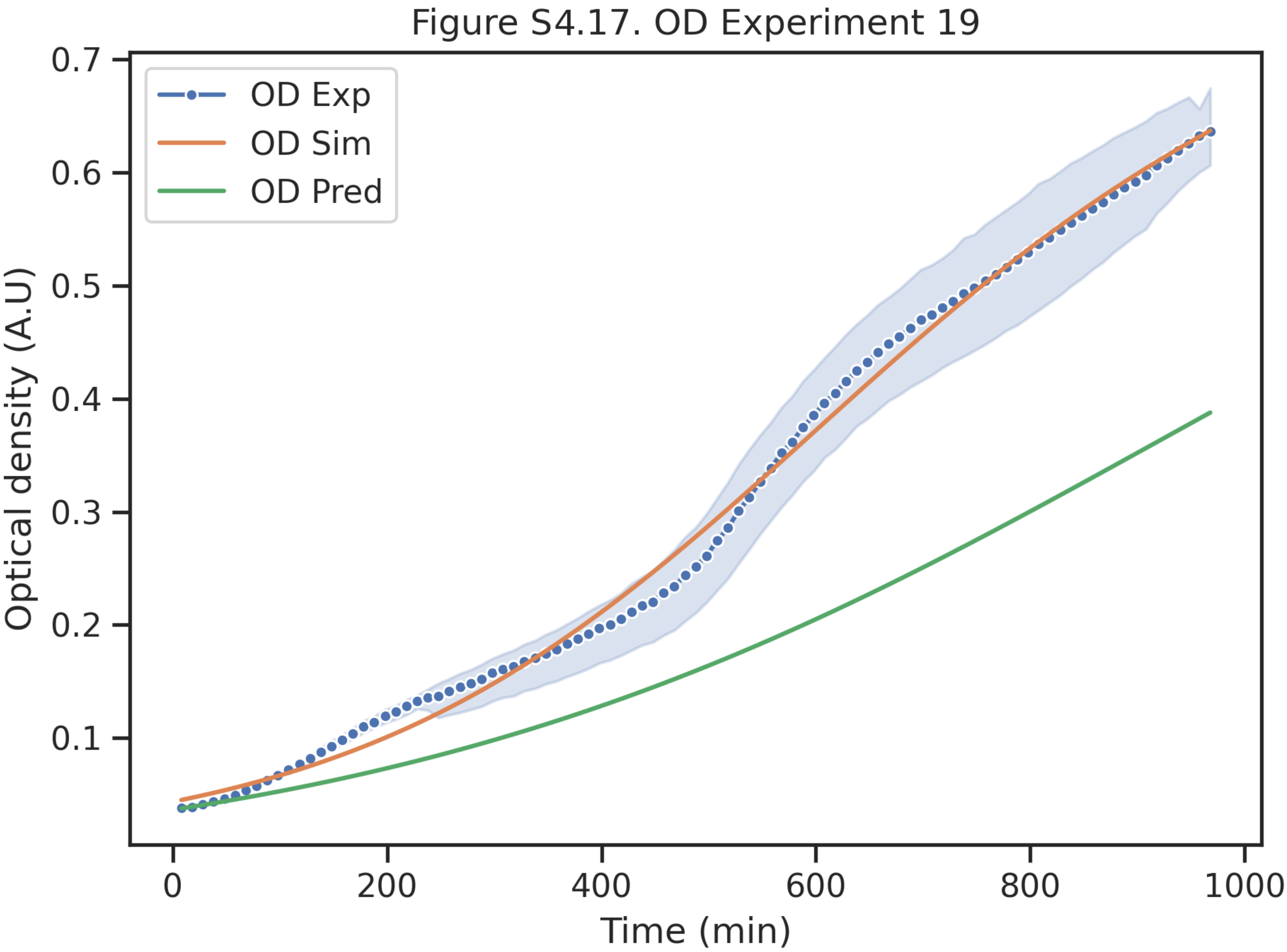

Figure S4.17. OD Experiment 19
OD Exp
OD Sim
OD Pred
Optical density (A.U)
0.1
0.2
0.3
0.4
0.5
0.6
0.7
0
200
400
600
800
1000
Time (min)

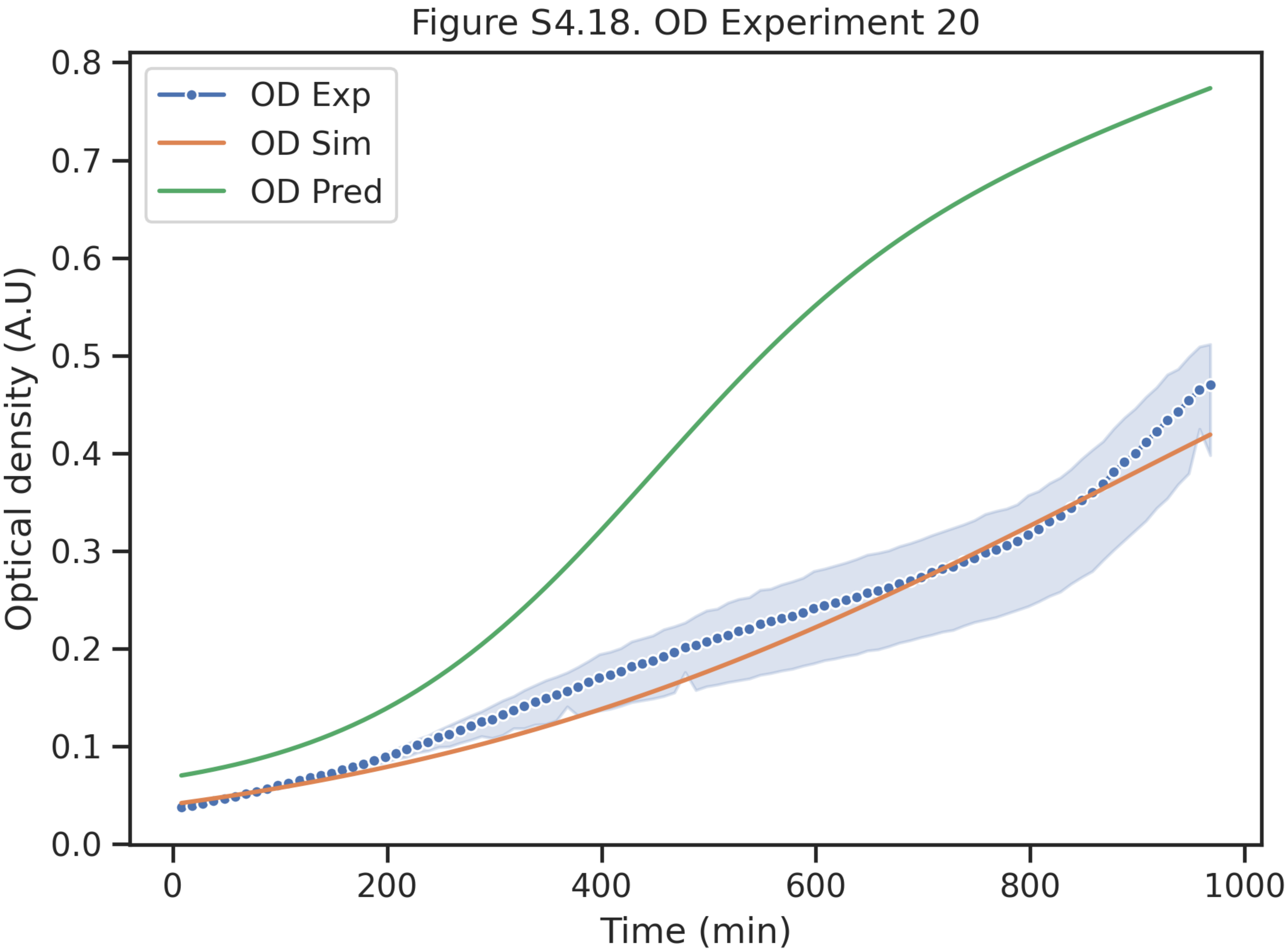
Figure S4.18. OD Experiment 20
OD Exp
OD Sim
OD Pred
Optical density (A.U)
Time (min)
0.0
0.1
0.2
0.3
0.4
0.5
0.6
0.7
0.8
0
200
400
600
800
1000

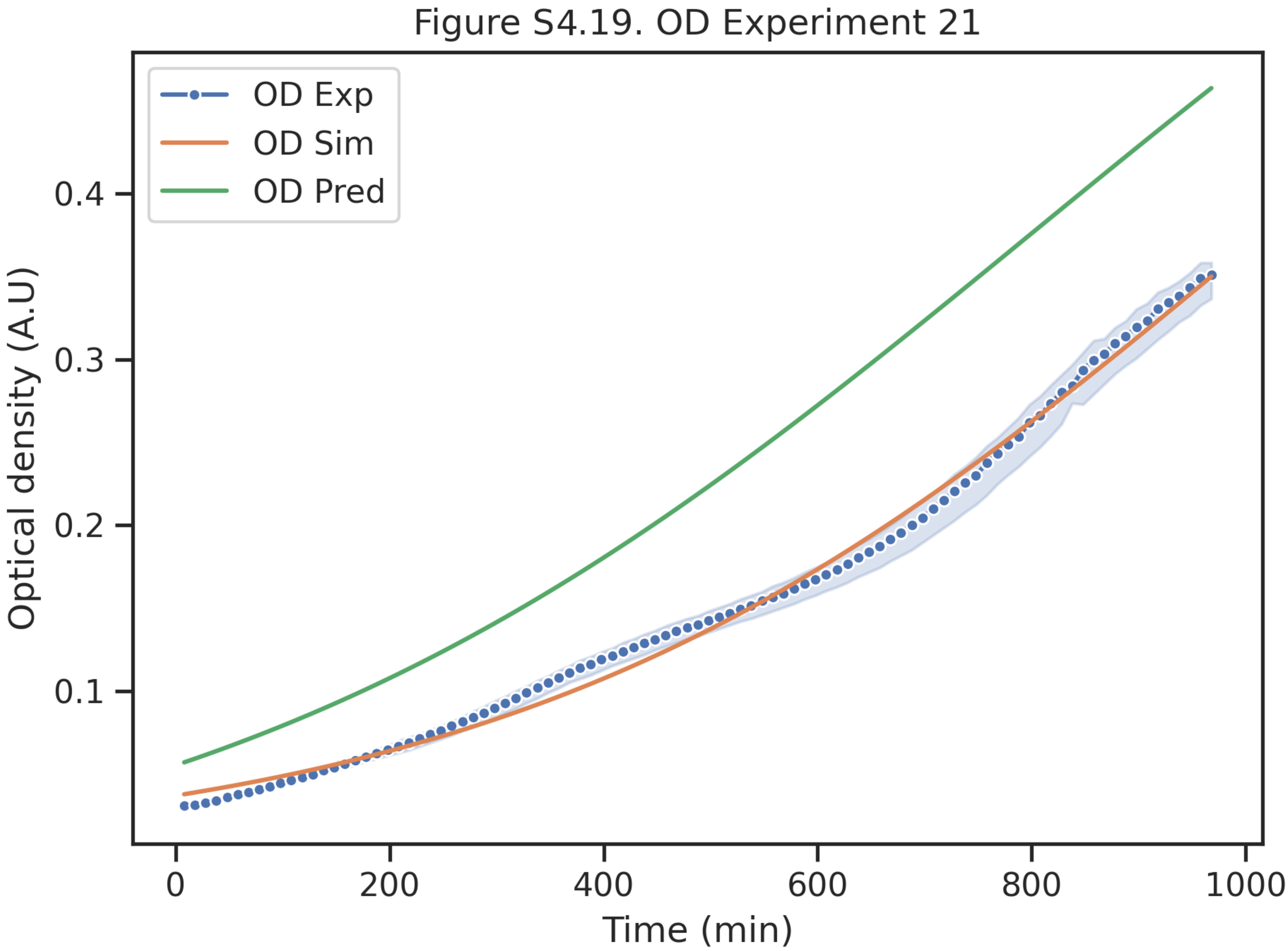
Figure S4.19. OD Experiment 21
OD Exp
OD Sim
OD Pred
Optical density (A.U)
Time (min)
0.1
0.2
0.3
0.4
0
200
400
600
800
1000

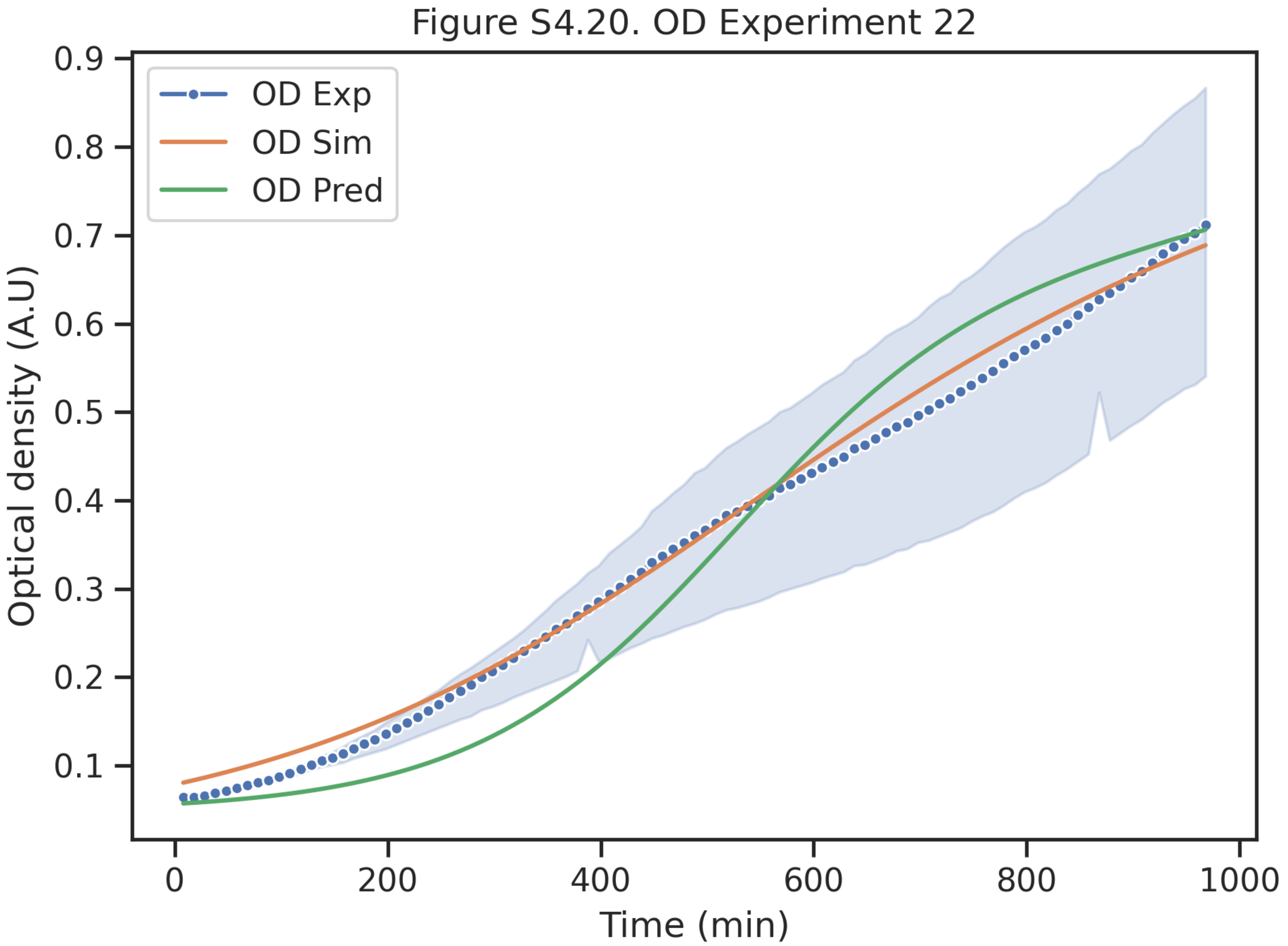

Figure S4.20. OD Experiment 22
OD Exp
OD Sim
OD Pred
Optical density (A.U)
0.1
0.2
0.3
0.4
0.5
0.6
0.7
0.8
0.9
0
200
400
600
800
1000
Time (min)

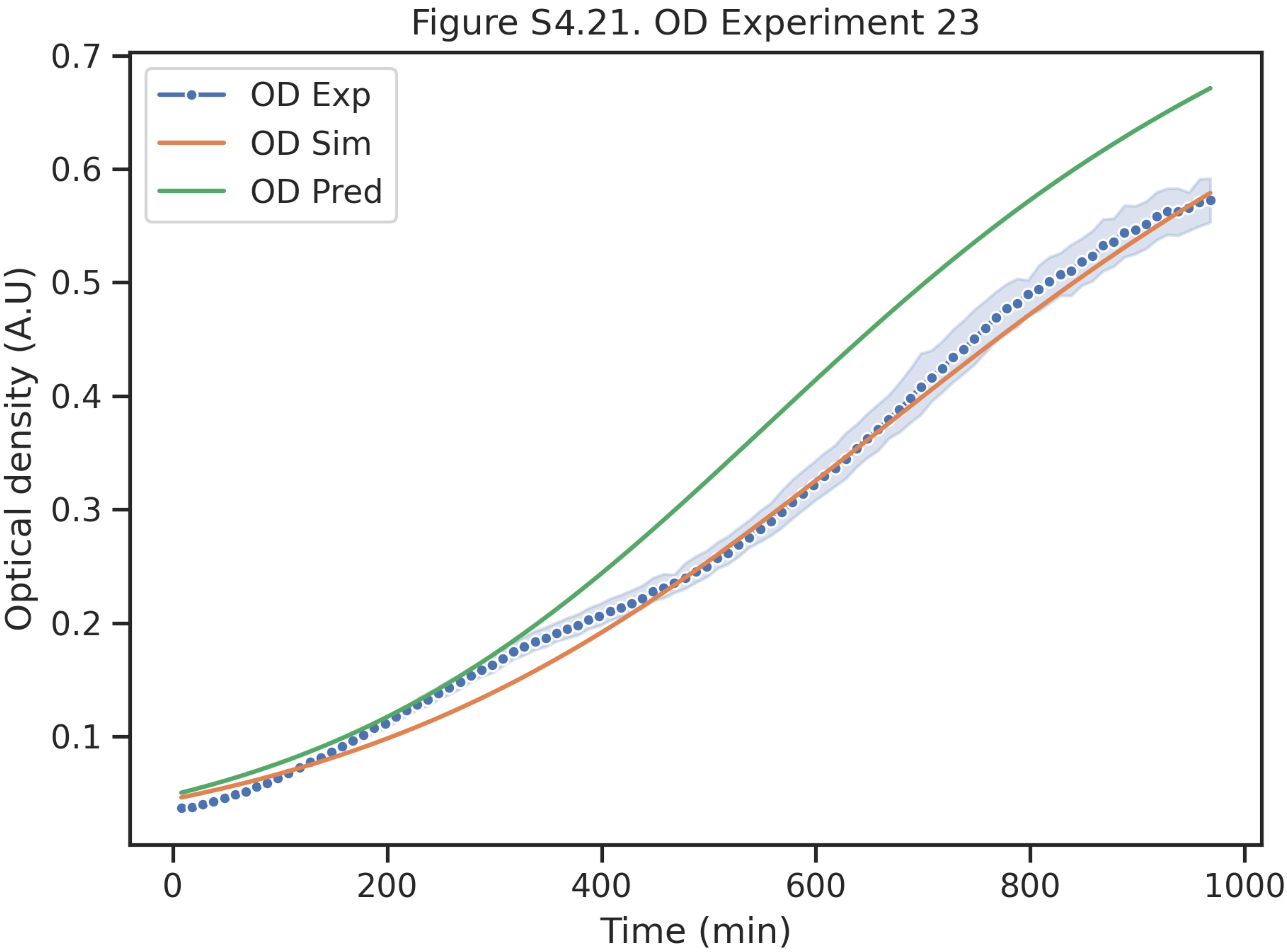
Figure S4.21. OD Experiment 23
OD Exp
OD Sim
OD Pred
Optical density (A.U)
0.7
0.6
0.5
0.4
0.3
0.2
0.1
0
200
400
600
800
1000
Time (min)

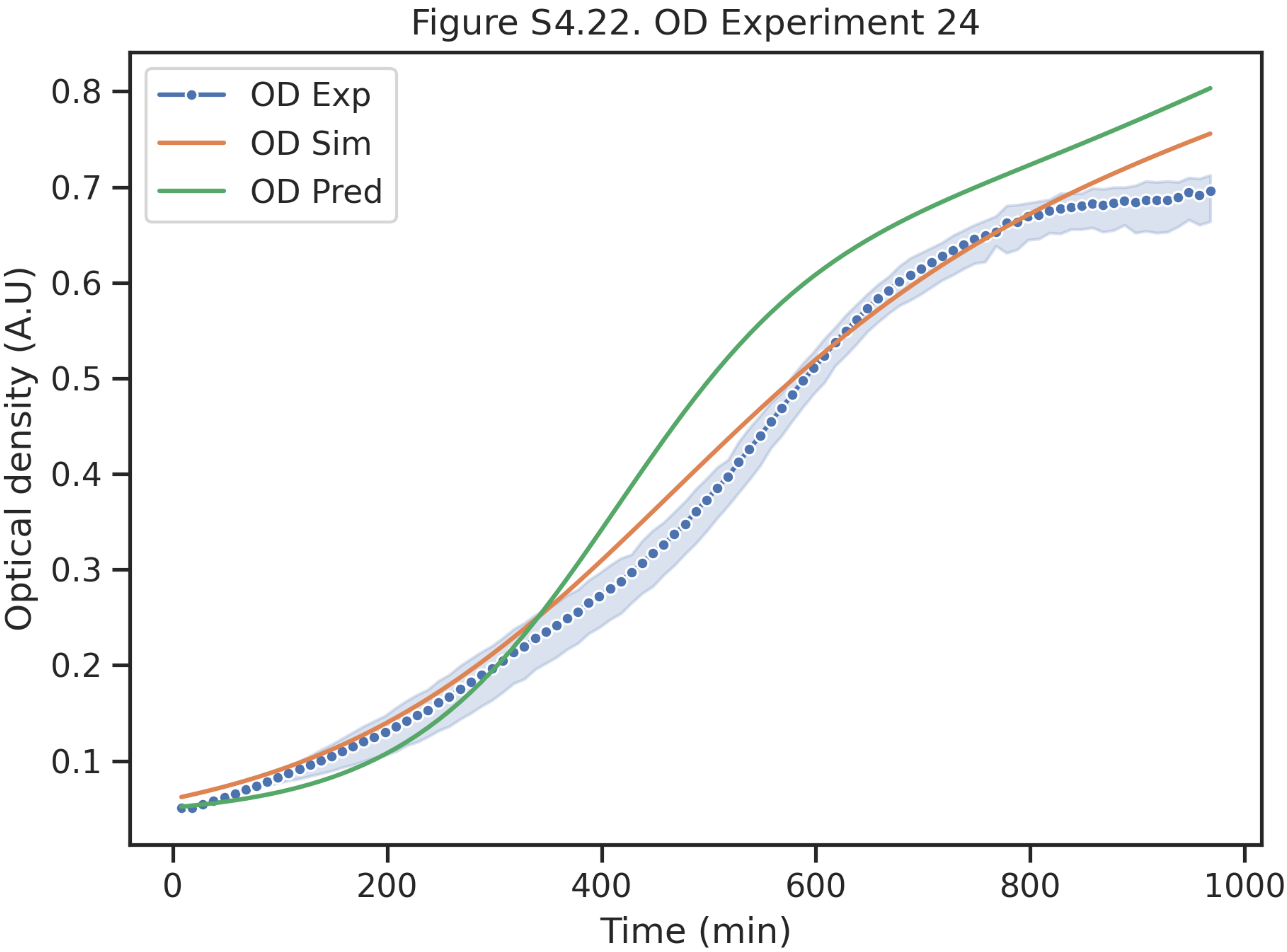

Figure S4.22. OD Experiment 24
OD Exp
OD Sim
OD Pred
Optical density (A.U)
0.1
0.2
0.3
0.4
0.5
0.6
0.7
0.8
0
200
400
600
800
1000
Time (min)

Figure S4.23. OD Experiment 25

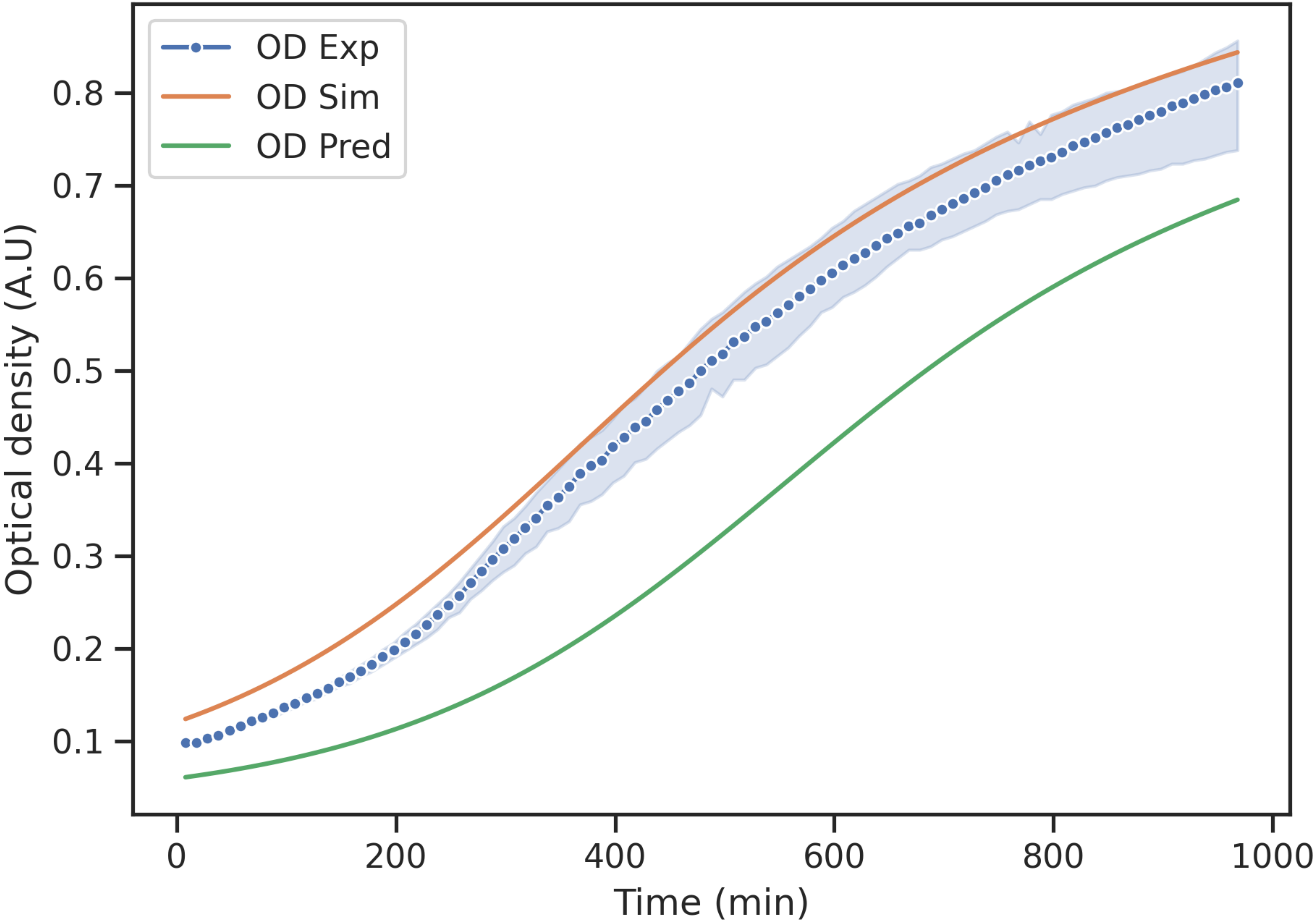

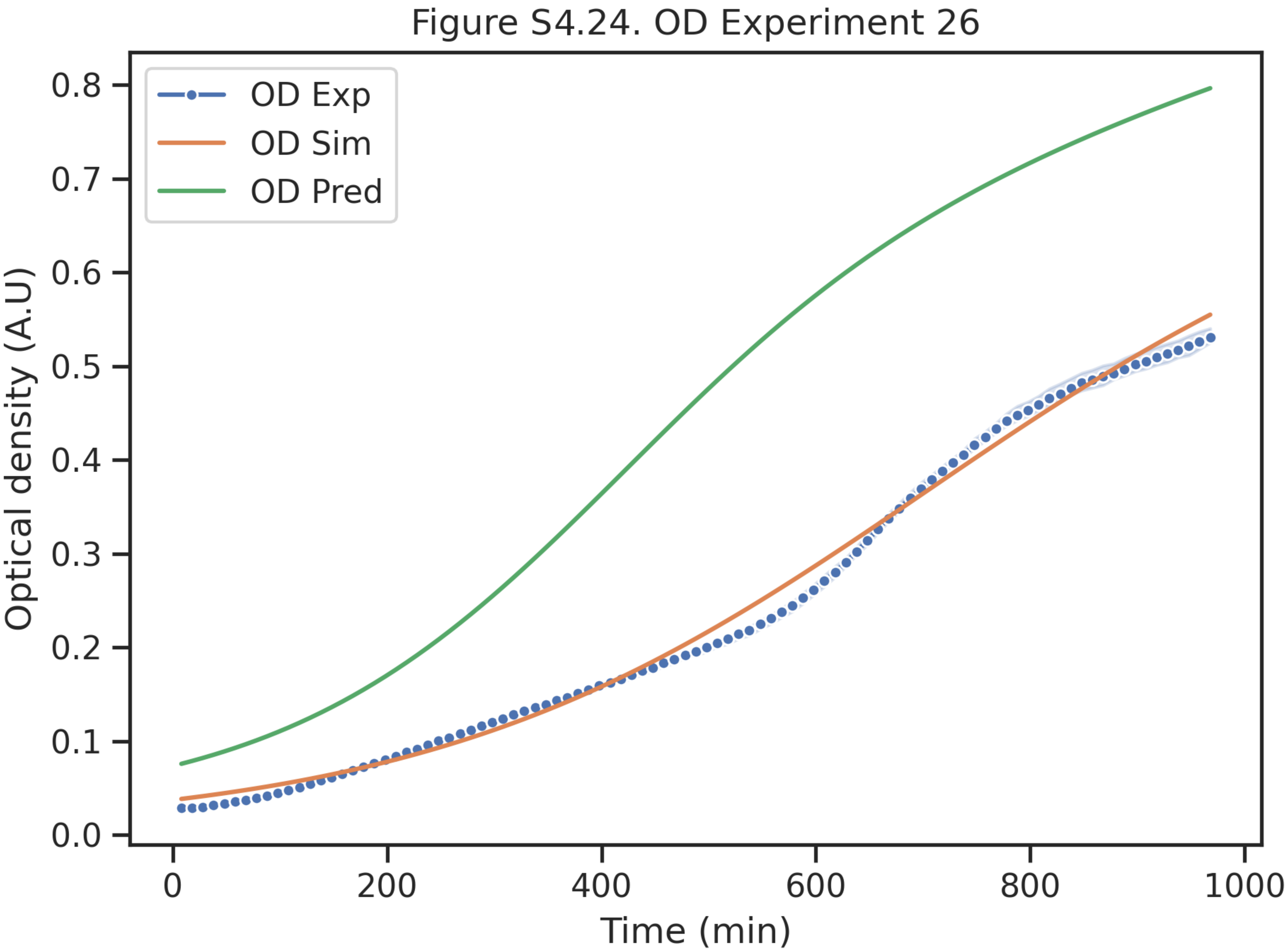
Figure S4.24. OD Experiment 26
OD Exp
OD Sim
OD Pred
Optical density (A.U)
Time (min)
0.0
0.1
0.2
0.3
0.4
0.5
0.6
0.7
0.8
0
200
400
600
800
1000

Figure S4.25. OD Experiment 28

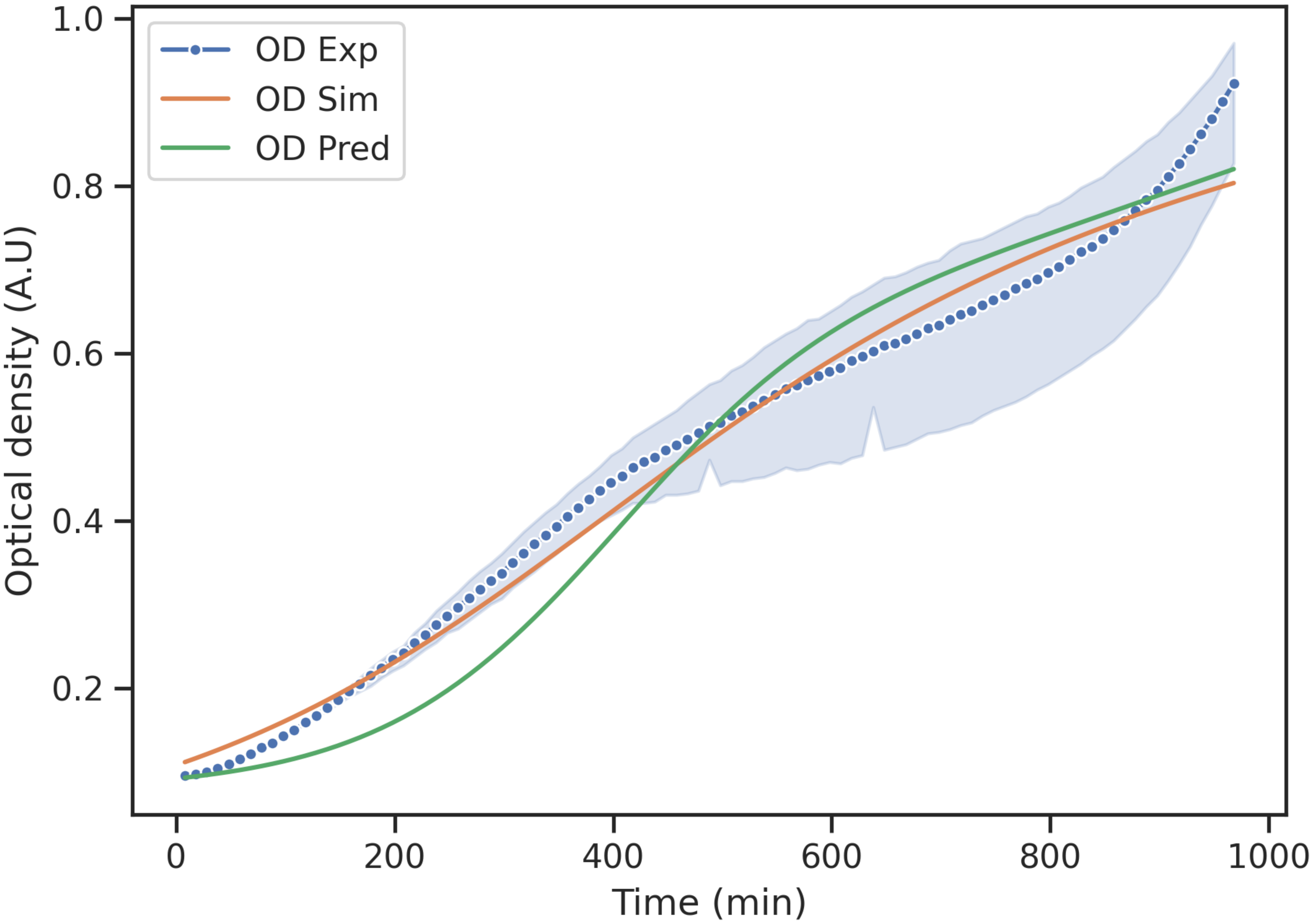

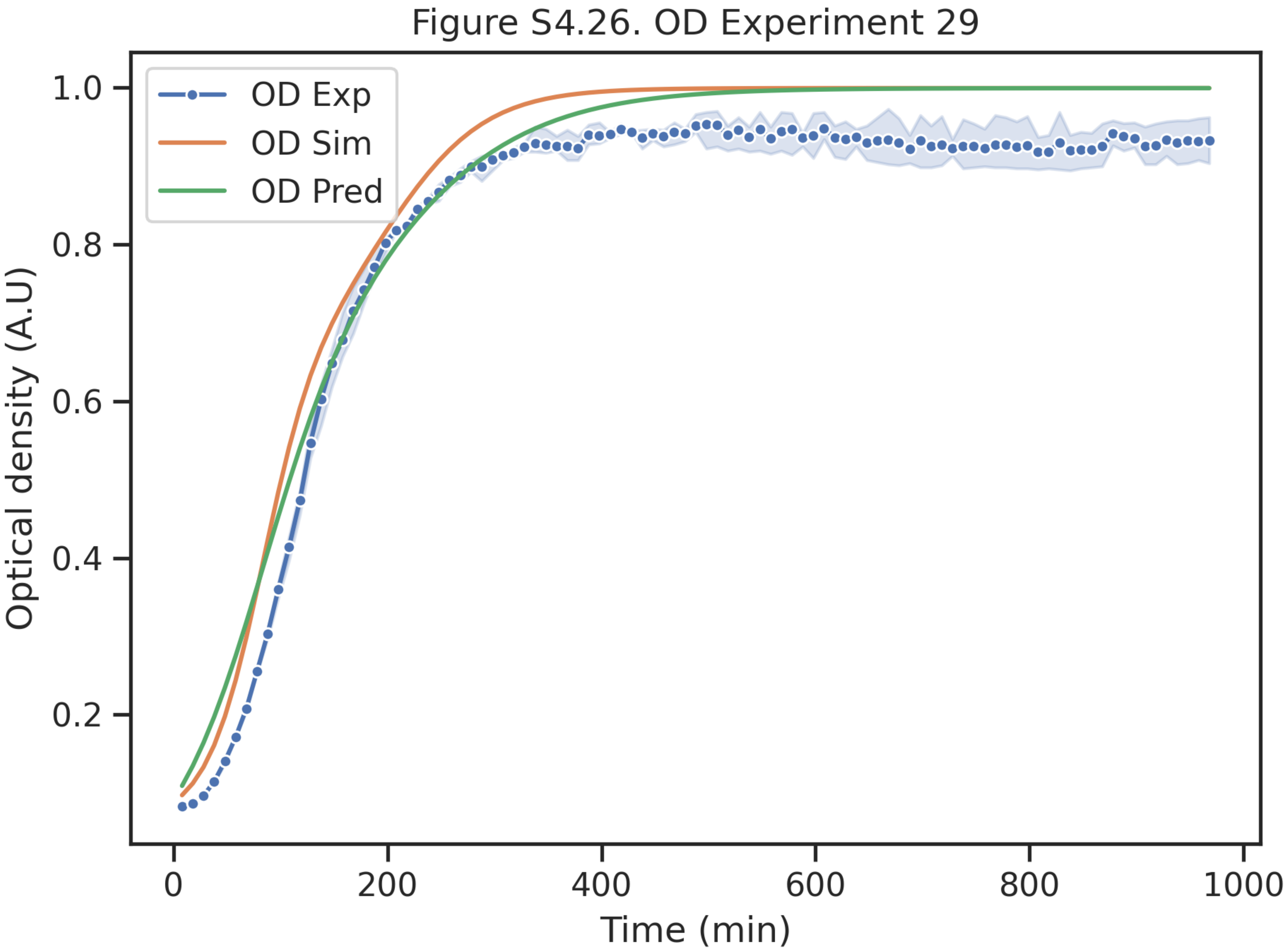
Figure S4.26. OD Experiment 29
OD Exp
OD Sim
OD Pred
Optical density (A.U)
Time (min)
1.0
0.8
0.6
0.4
0.2
0
200
400
600
800
1000

Figure S4.27. OD Experiment 30

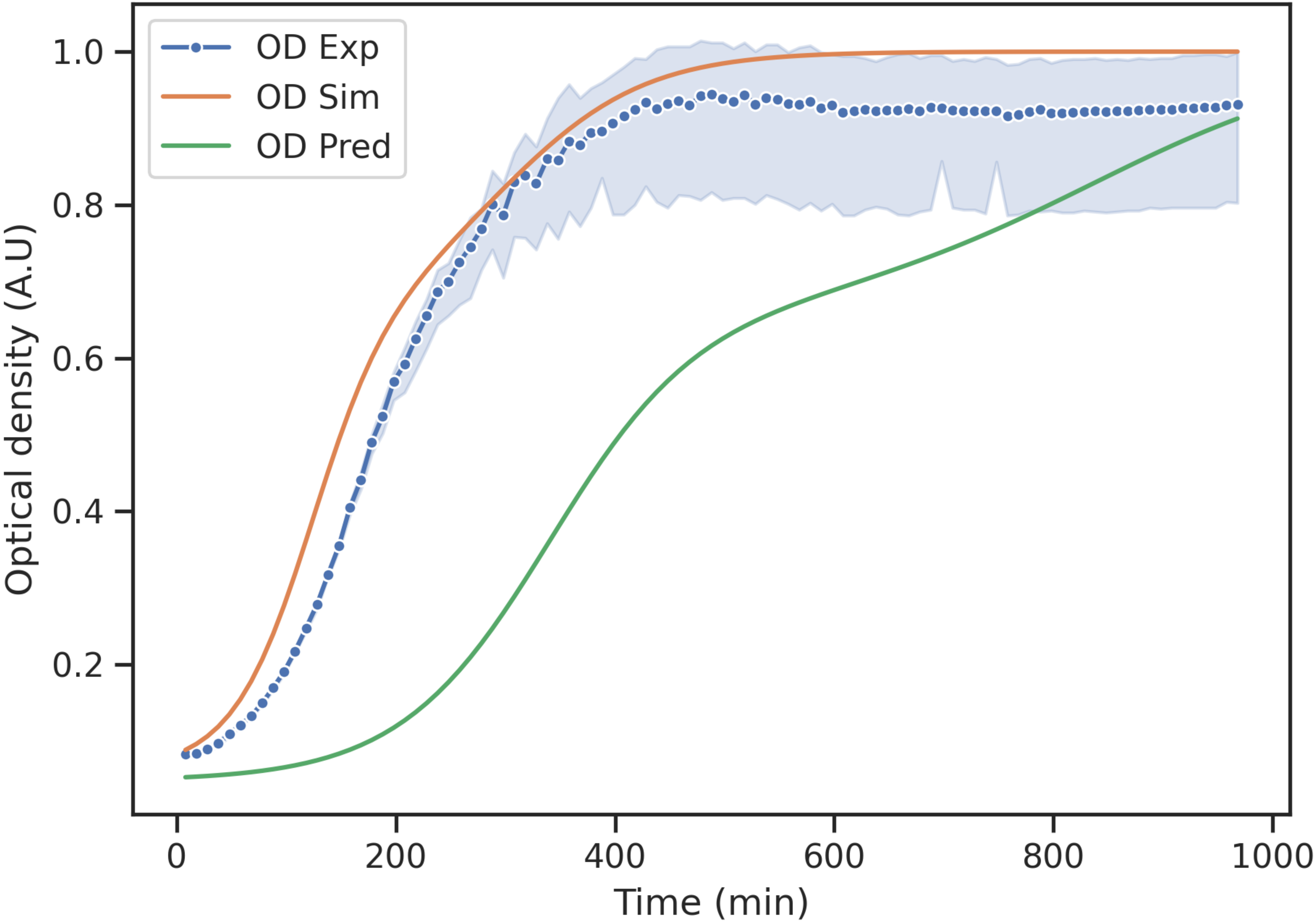

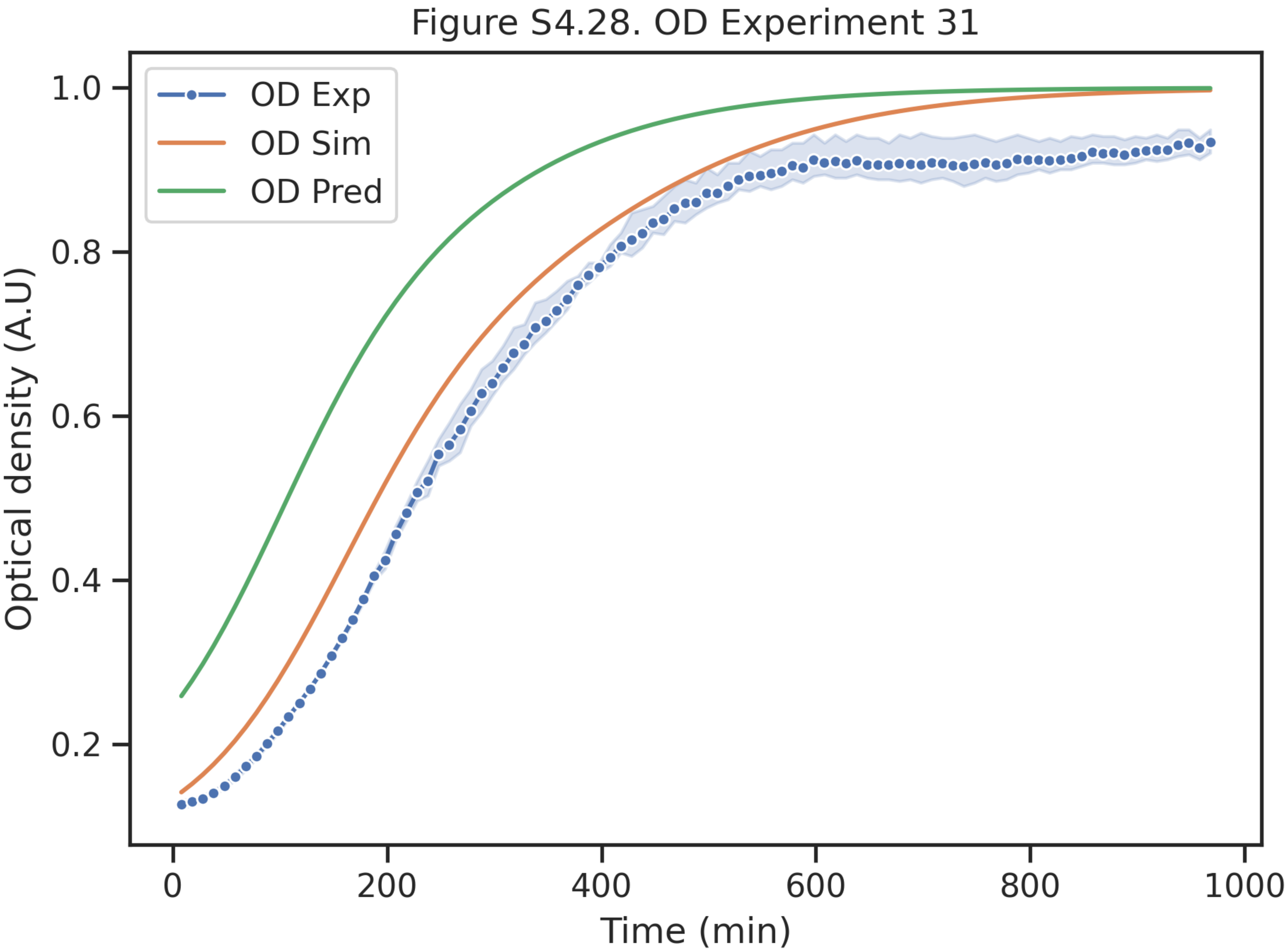

Figure S4.28. OD Experiment 31
OD Exp
OD Sim
OD Pred
Optical density (A.U)
1.0
0.8
0.6
0.4
0.2
0
200
400
600
800
1000
Time (min)

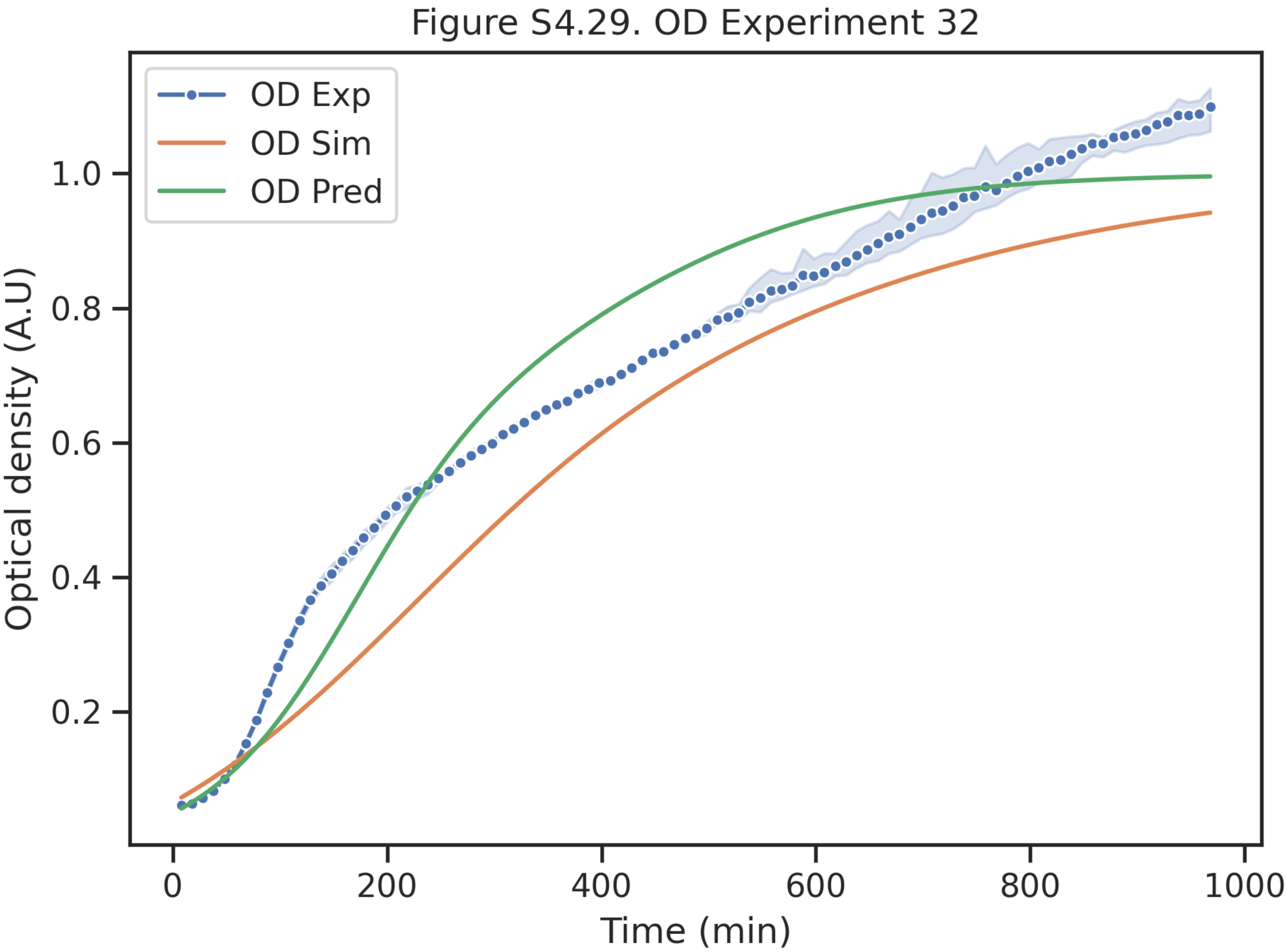

Figure S4.29. OD Experiment 32
OD Exp
OD Sim
OD Pred
Optical density (A.U)
1.0
0.8
0.6
0.4
0.2
0
200
400
600
800
1000
Time (min)

Figure S4.30. OD Experiment 33

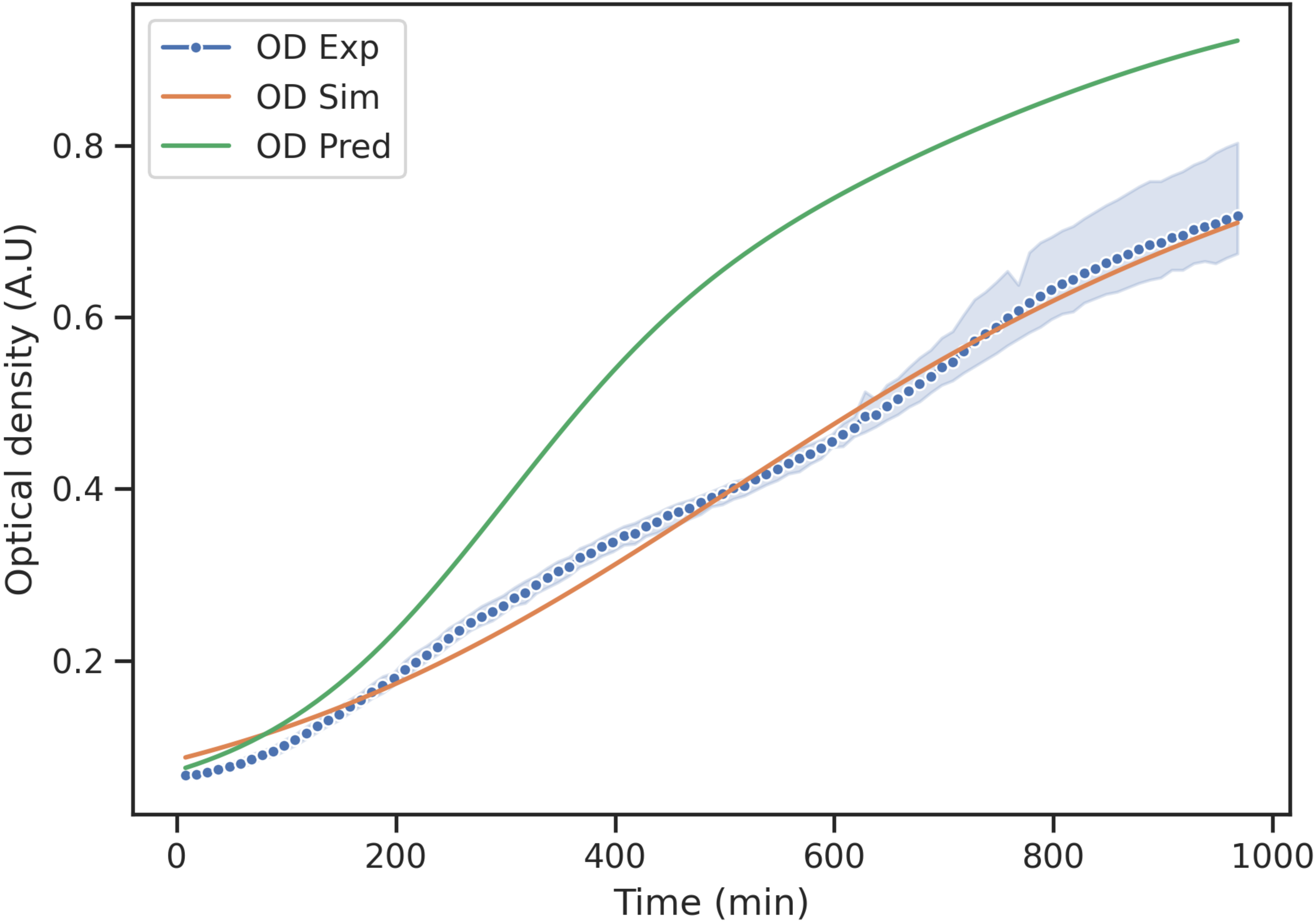

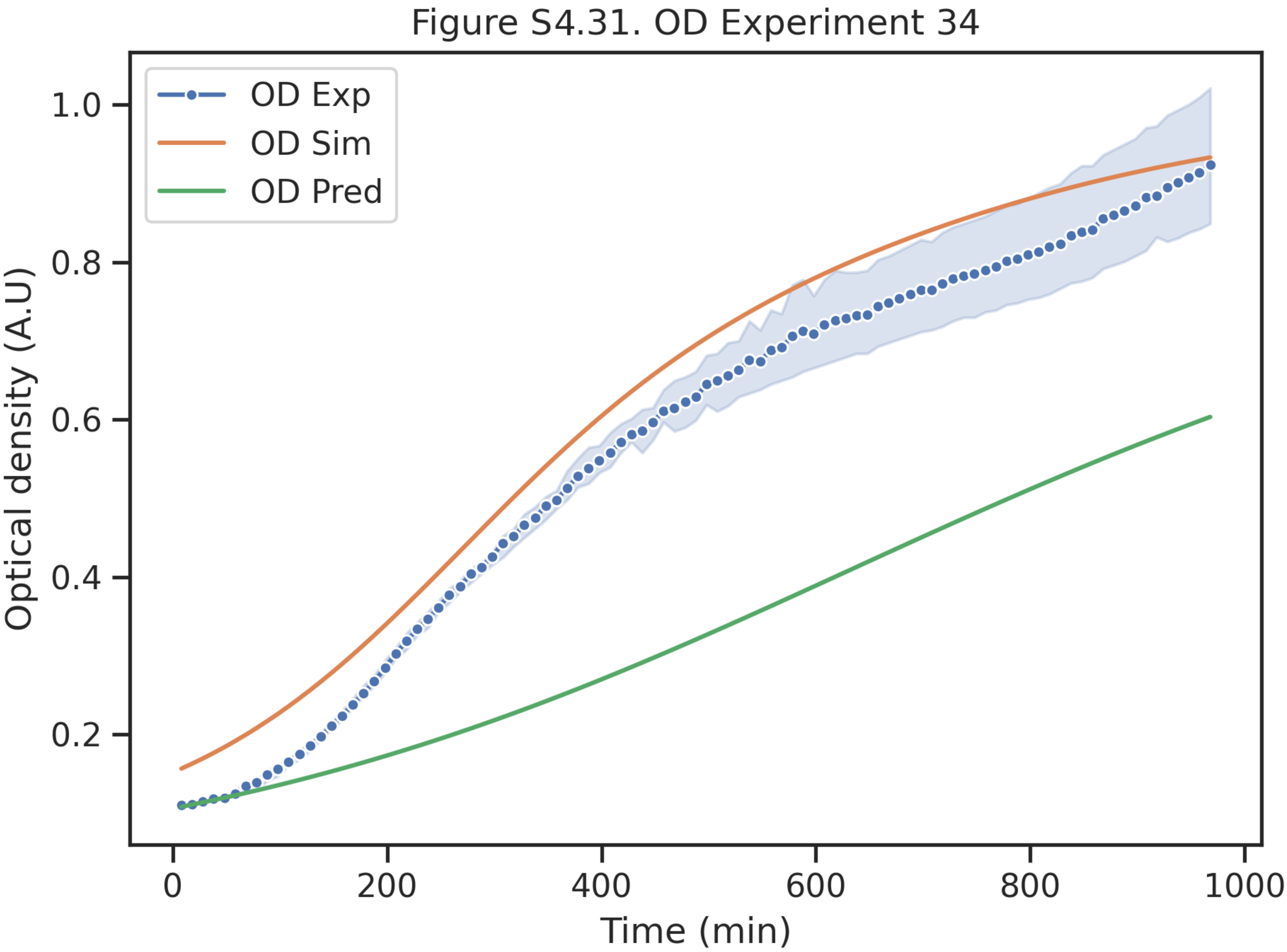

Figure S4.31. OD Experiment 34
OD Exp
OD Sim
OD Pred
Optical density (A.U)
1.0
0.8
0.6
0.4
0.2
0
200
400
600
800
1000
Time (min)

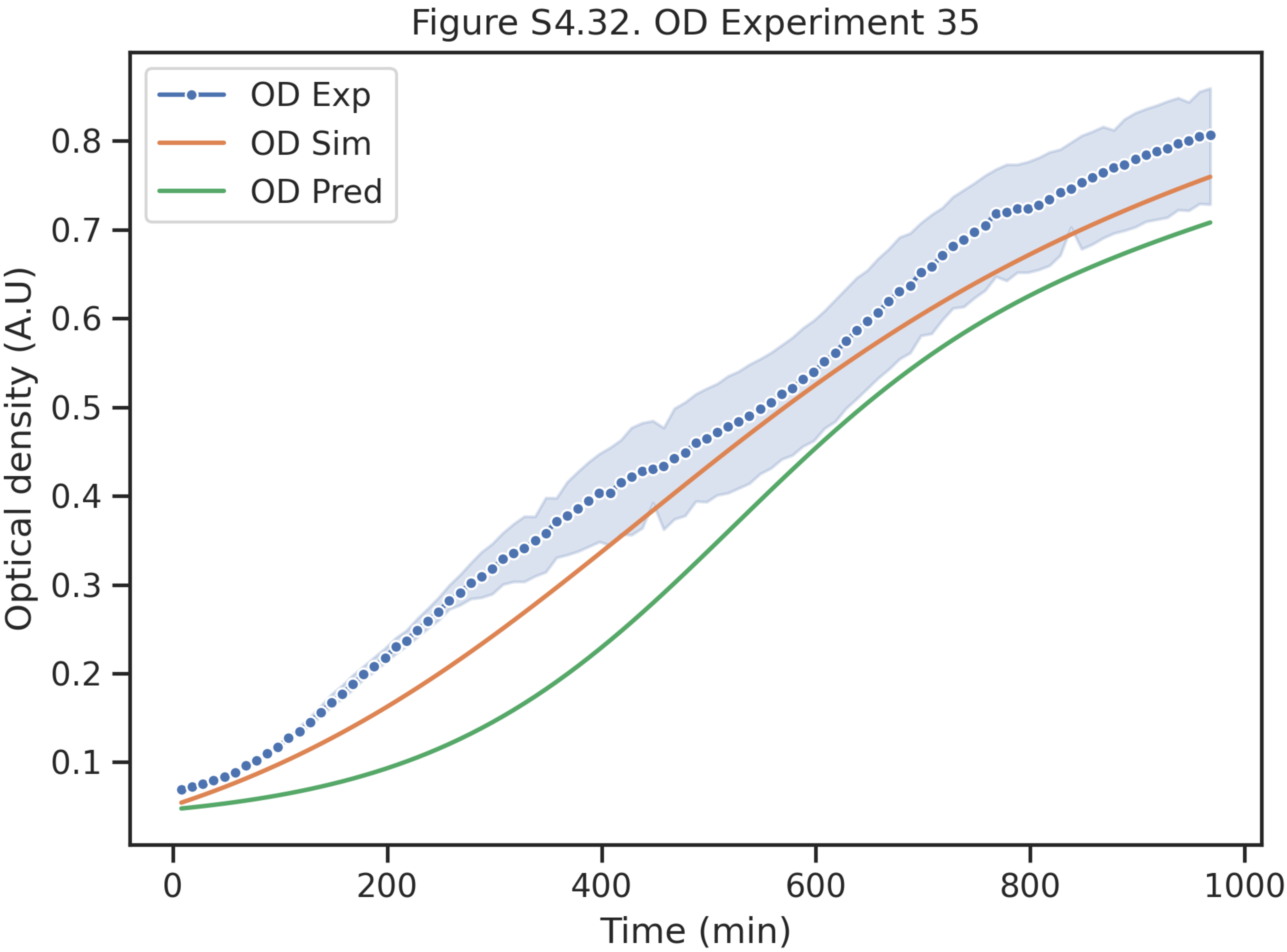
Figure S4.32. OD Experiment 35
OD Exp
OD Sim
OD Pred
Optical density (A.U)
0.1
0.2
0.3
0.4
0.5
0.6
0.7
0.8
0
200
400
600
800
1000
Time (min)

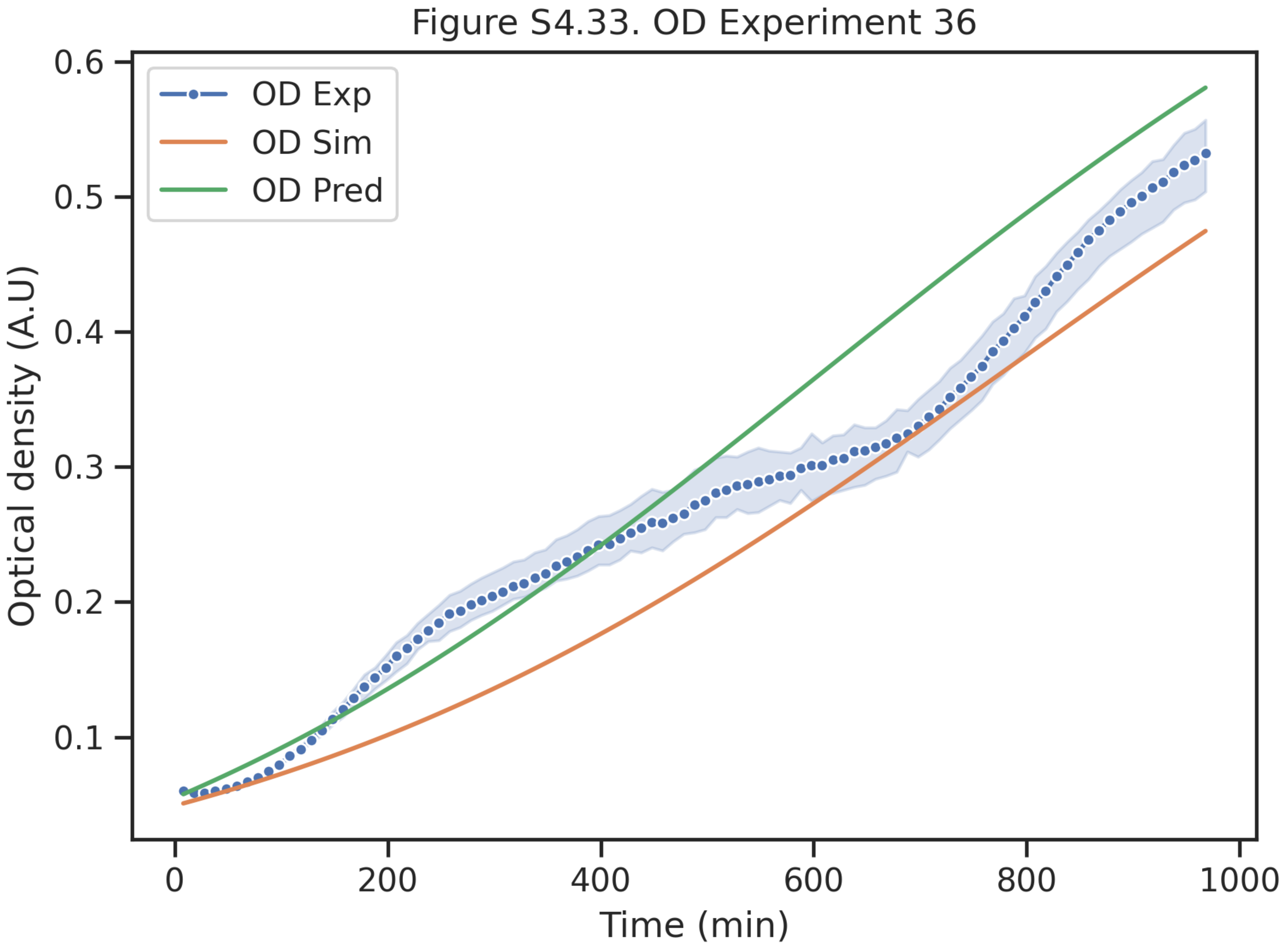

Figure S4.33. OD Experiment 36
OD Exp
OD Sim
OD Pred
Optical density (A.U)
0.1
0.2
0.3
0.4
0.5
0.6
0
200
400
600
800
1000
Time (min)

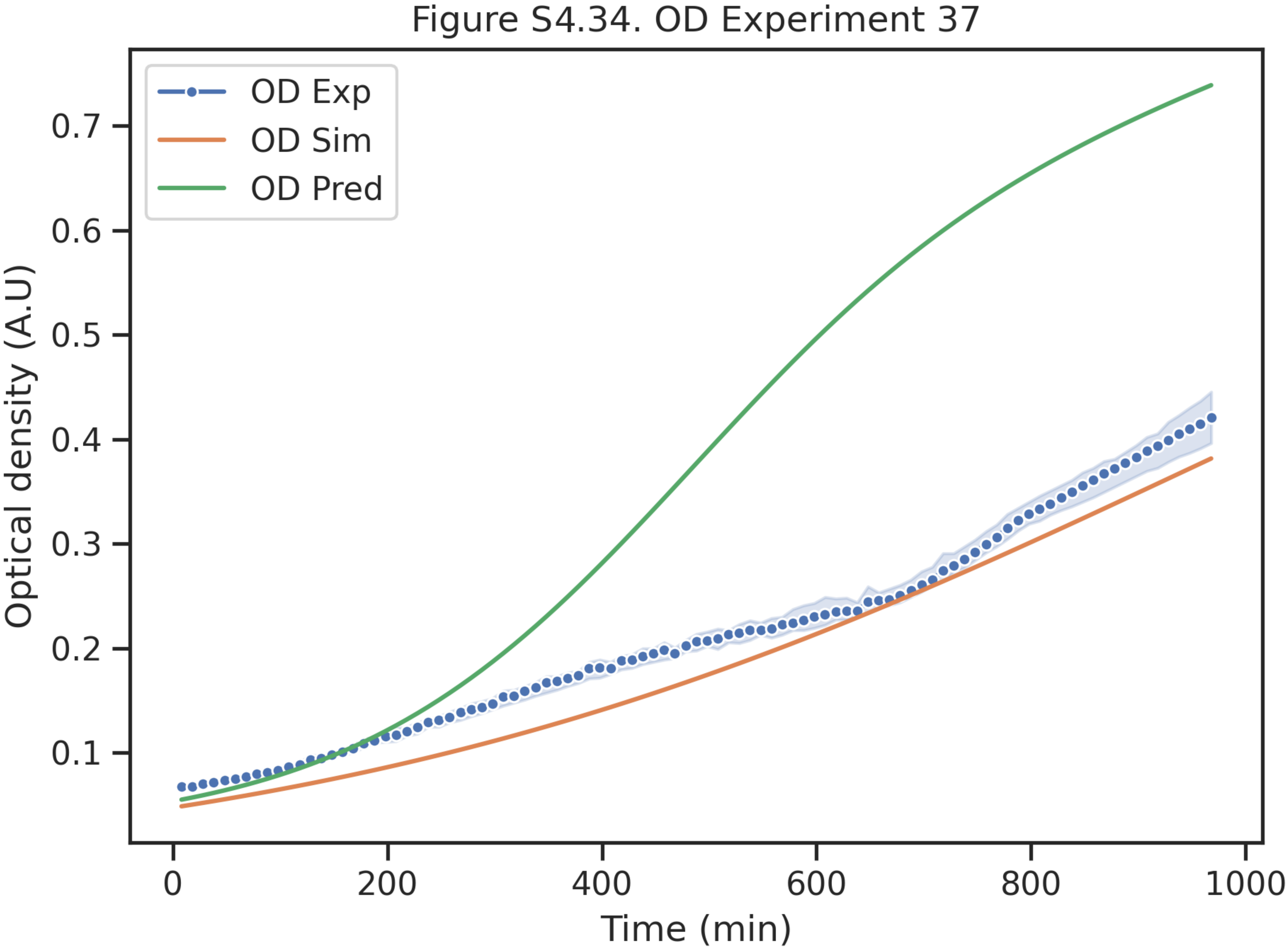
Figure S4.34. OD Experiment 37
OD Exp
OD Sim
OD Pred
Optical density (A.U)
0.1
0.2
0.3
0.4
0.5
0.6
0.7
0
200
400
600
800
1000
Time (min)

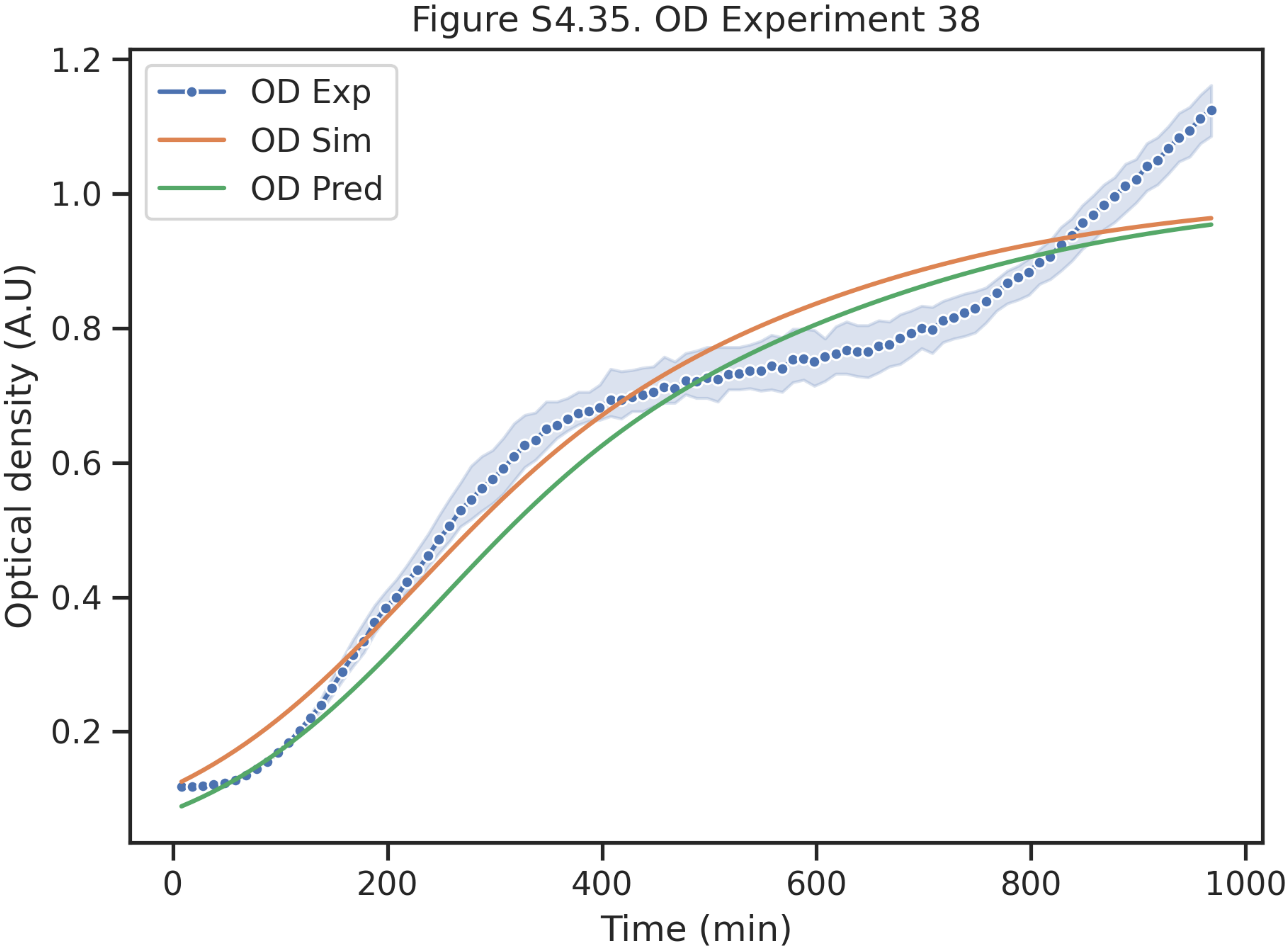
Figure S4.35. OD Experiment 38
OD Exp
OD Sim
OD Pred
Optical density (A.U)
Time (min)
0.2
0.4
0.6
0.8
1.0
1.2
0
200
400
600
800
1000

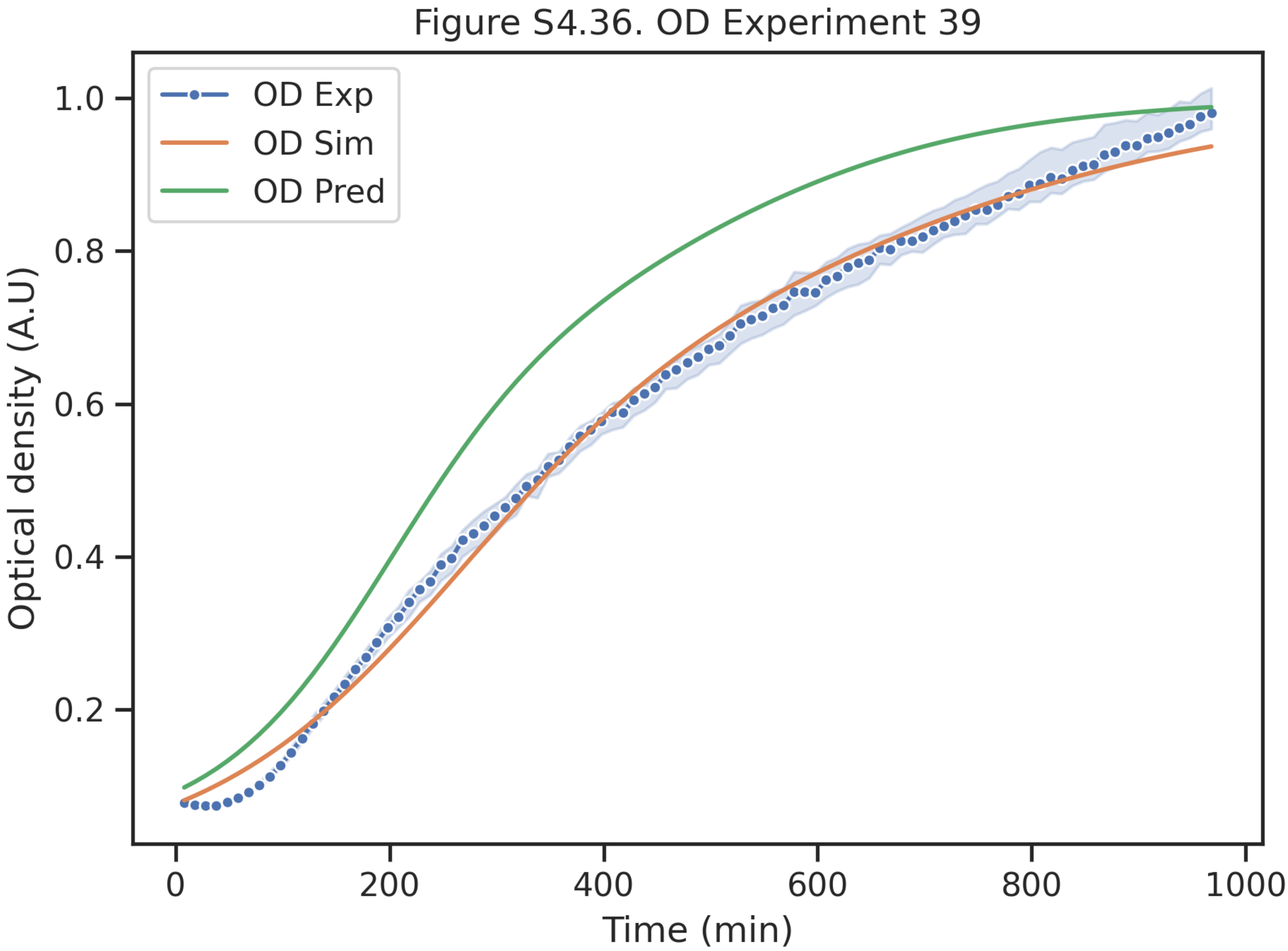

Figure S4.36. OD Experiment 39
OD Exp
OD Sim
OD Pred
1.0
0.8
0.6
0.4
0.2
Optical density (A.U)
0
200
400
600
800
1000
Time (min)

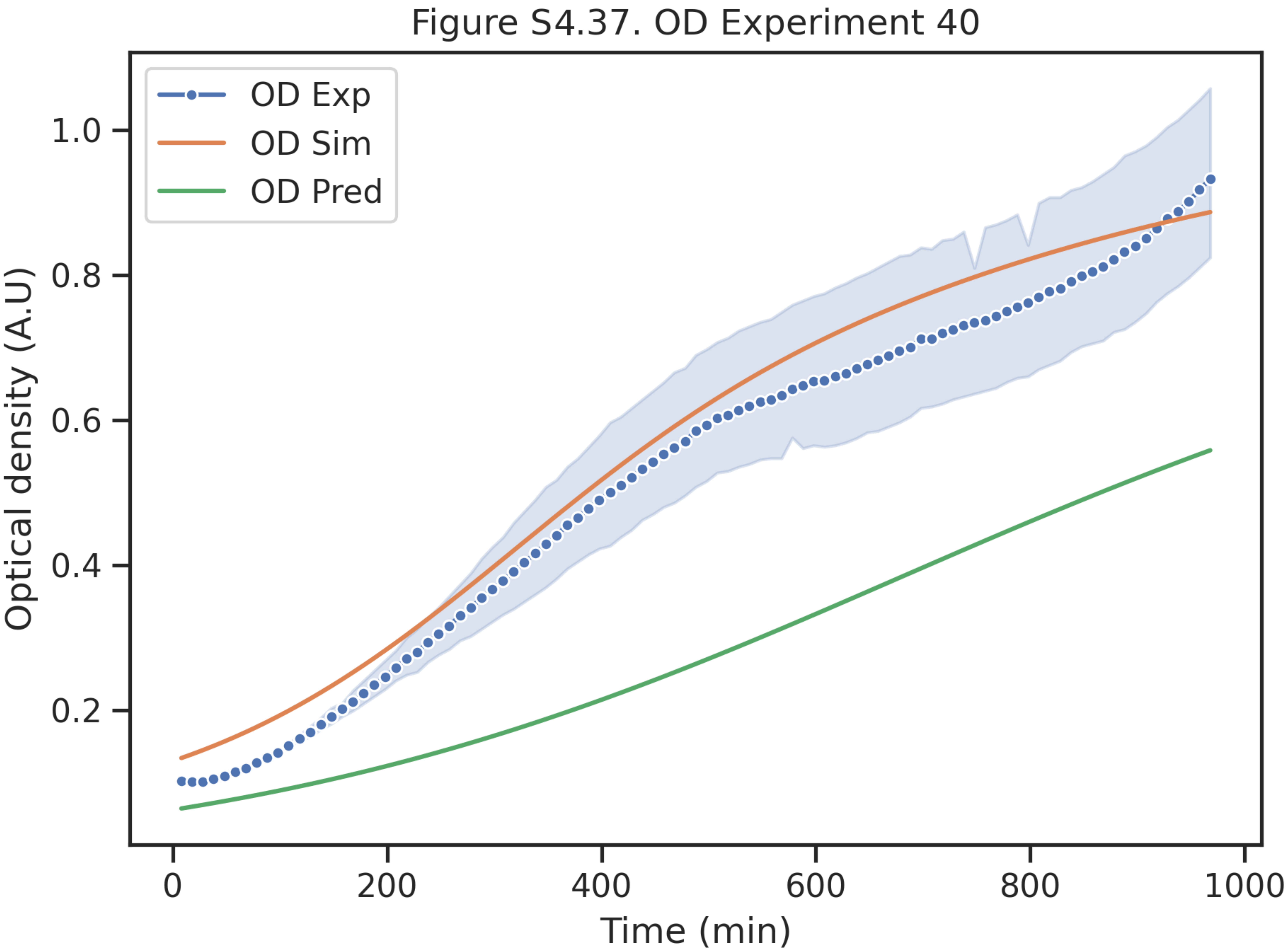
Figure S4.37. OD Experiment 40
OD Exp
OD Sim
OD Pred
1.0
0.8
0.6
0.4
0.2
Optical density (A.U)
0
200
400
600
800
1000
Time (min)

Figure S4.38. OD Experiment 41

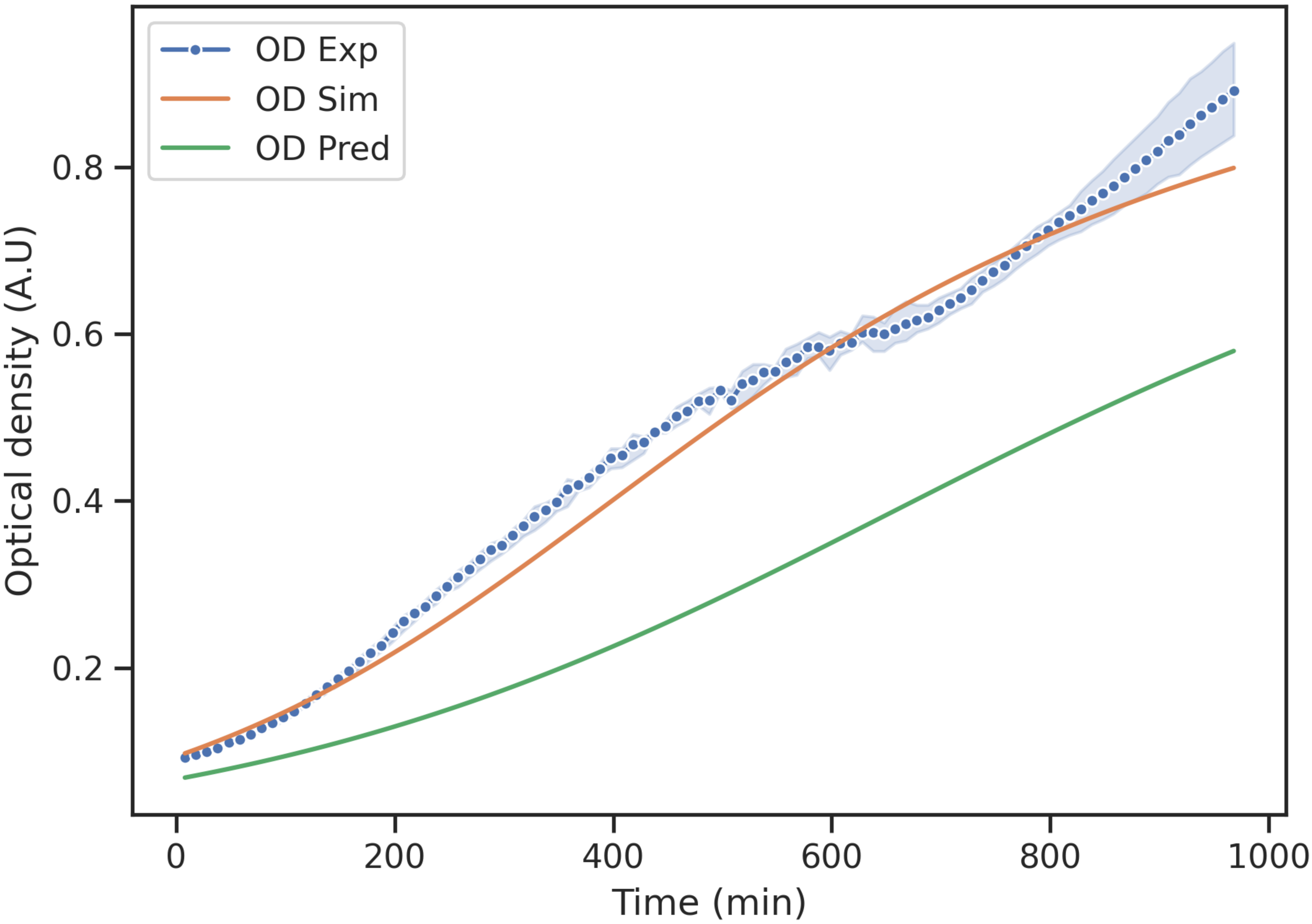

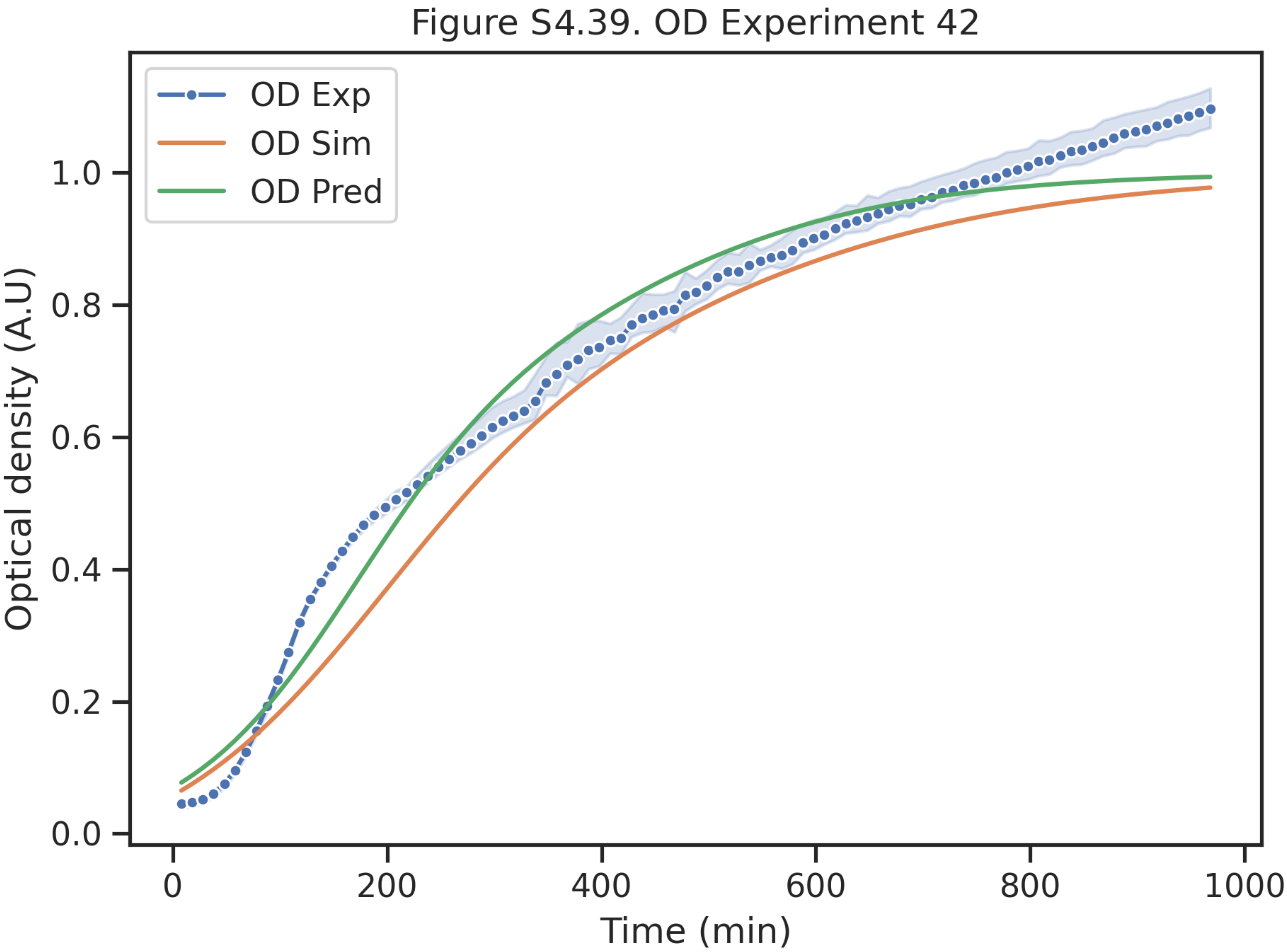
Figure S4.39. OD Experiment 42
OD Exp
OD Sim
OD Pred
Optical density (A.U)
0.0
0.2
0.4
0.6
0.8
1.0
0
200
400
600
800
1000
Time (min)

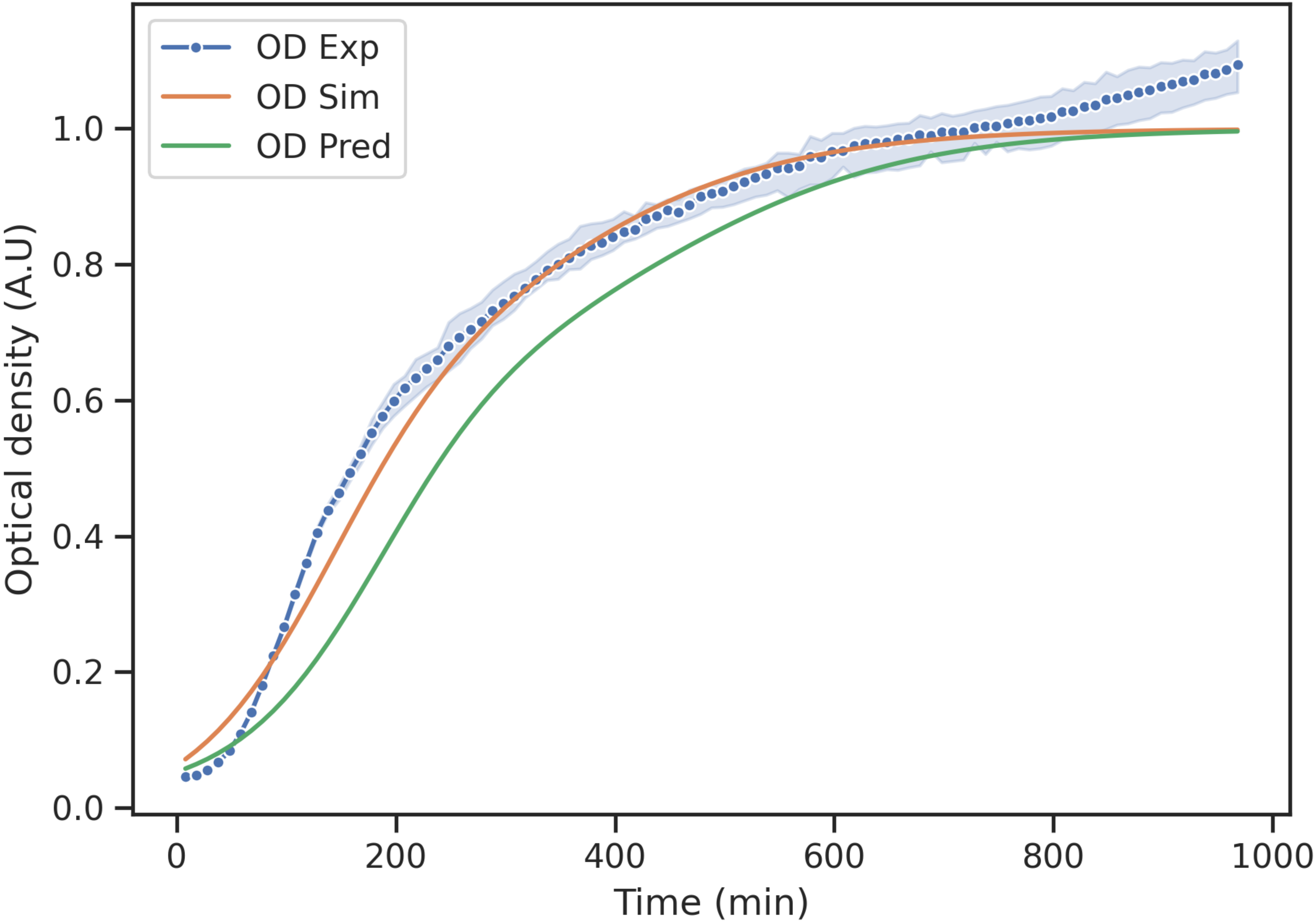

Figure S4.40. OD Experiment 43

Figure S4.41. OD Experiment 44

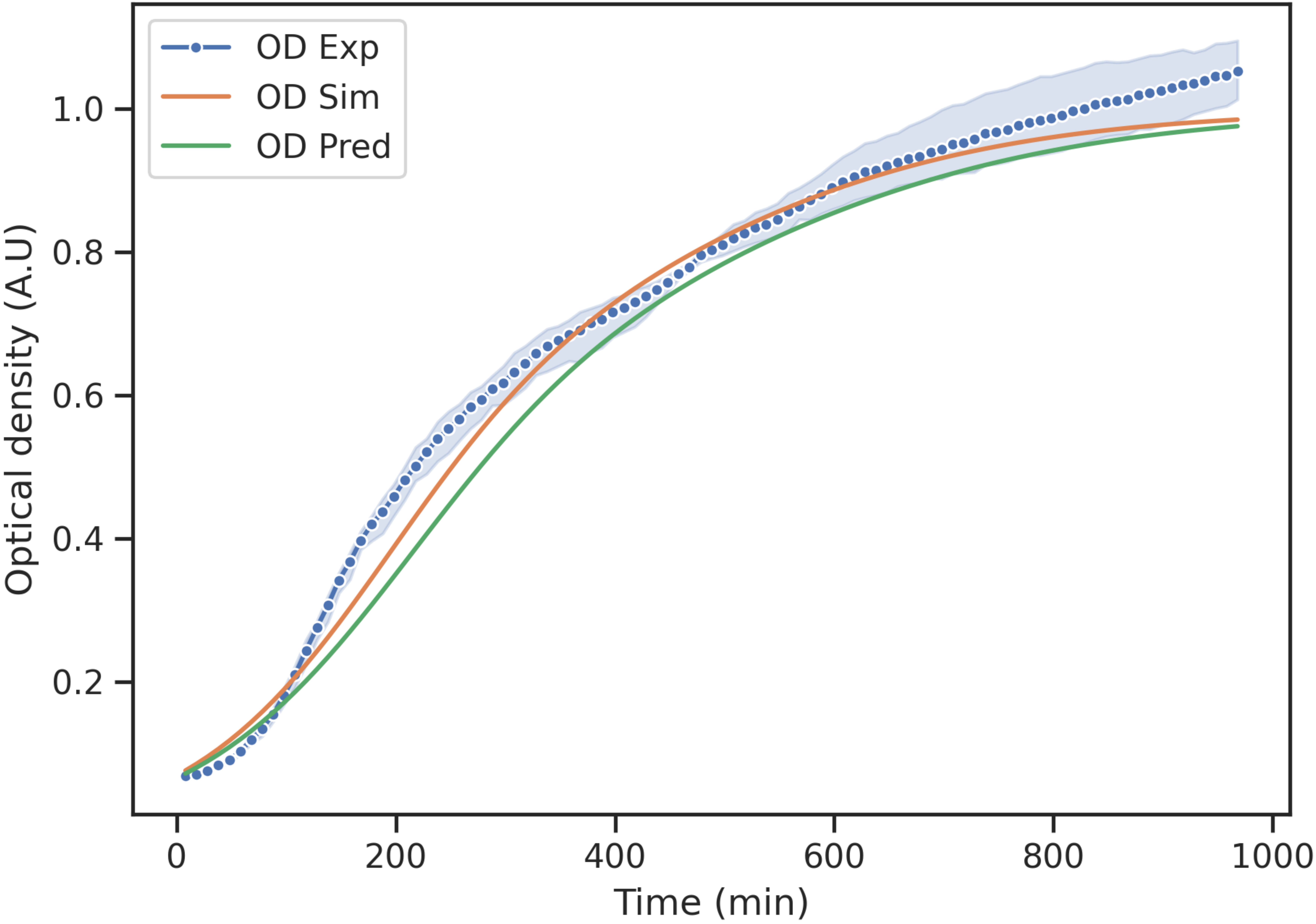

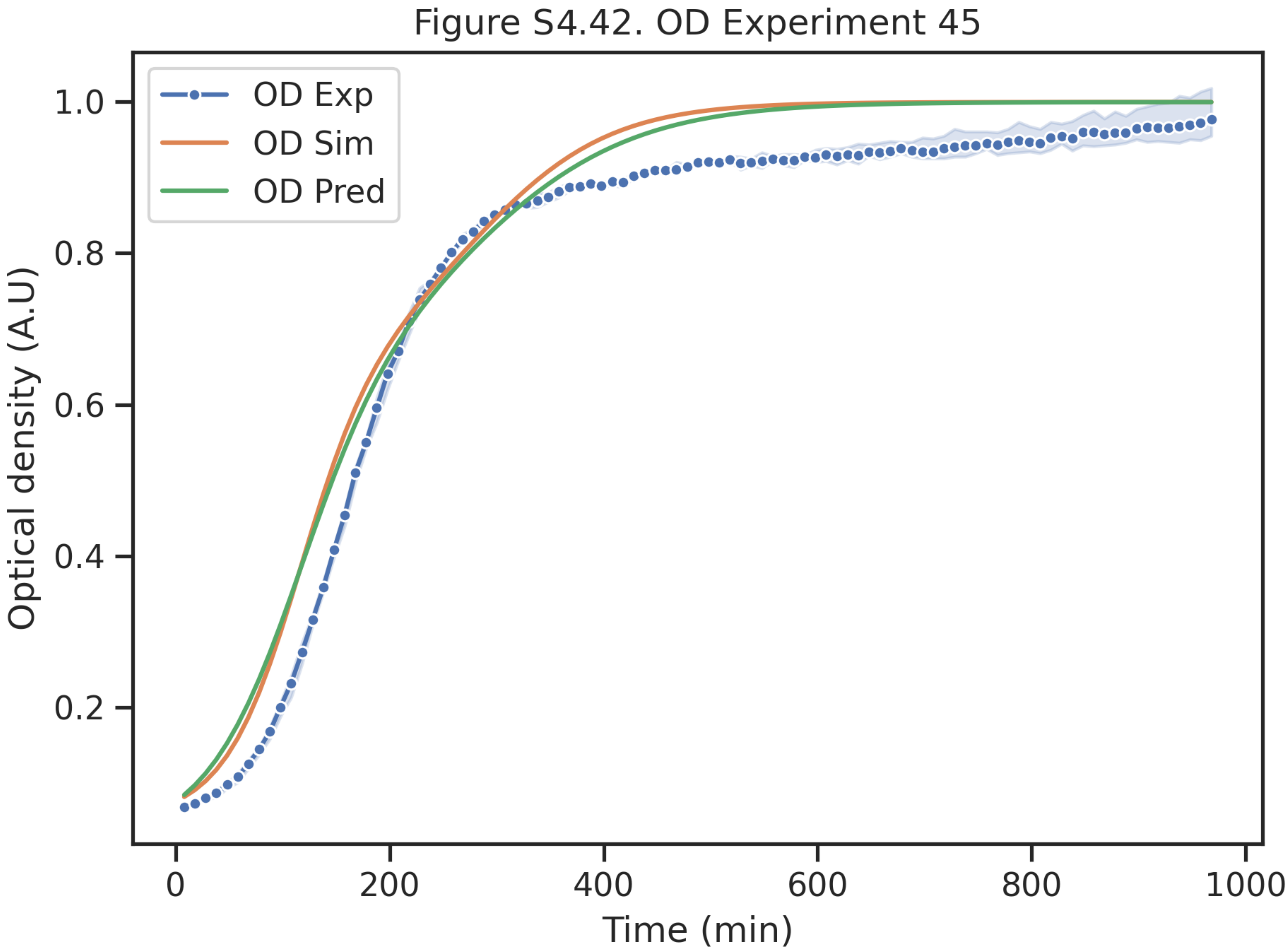

Figure S4.42. OD Experiment 45
OD Exp
OD Sim
OD Pred
Optical density (A.U)
Time (min)
0.2
0.4
0.6
0.8
1.0
0
200
400
600
800
1000

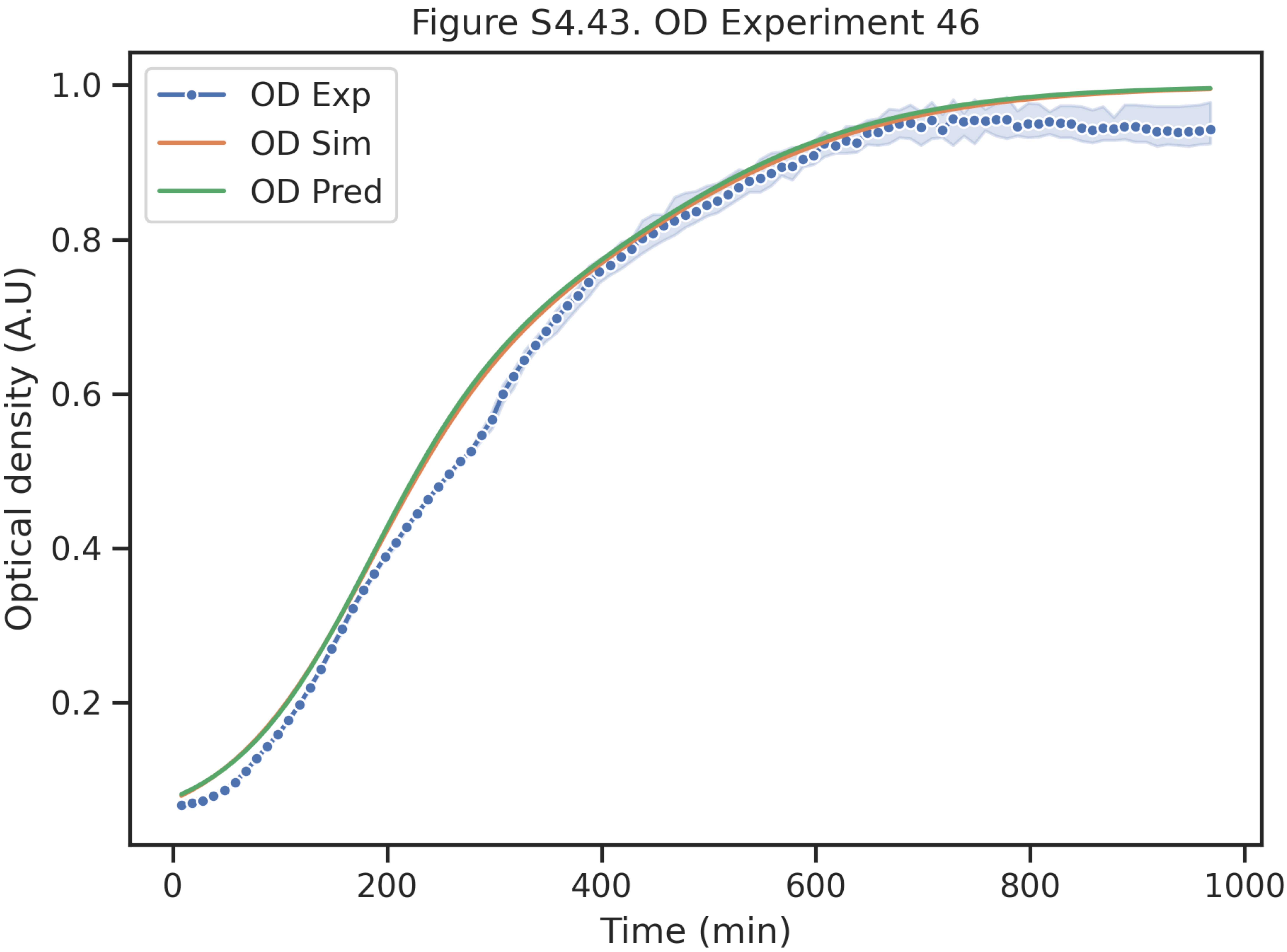
Figure S4.43. OD Experiment 46
OD Exp
OD Sim
OD Pred
1.0
0.8
0.6
0.4
0.2
Optical density (A.U)
0
200
400
600
800
1000
Time (min)

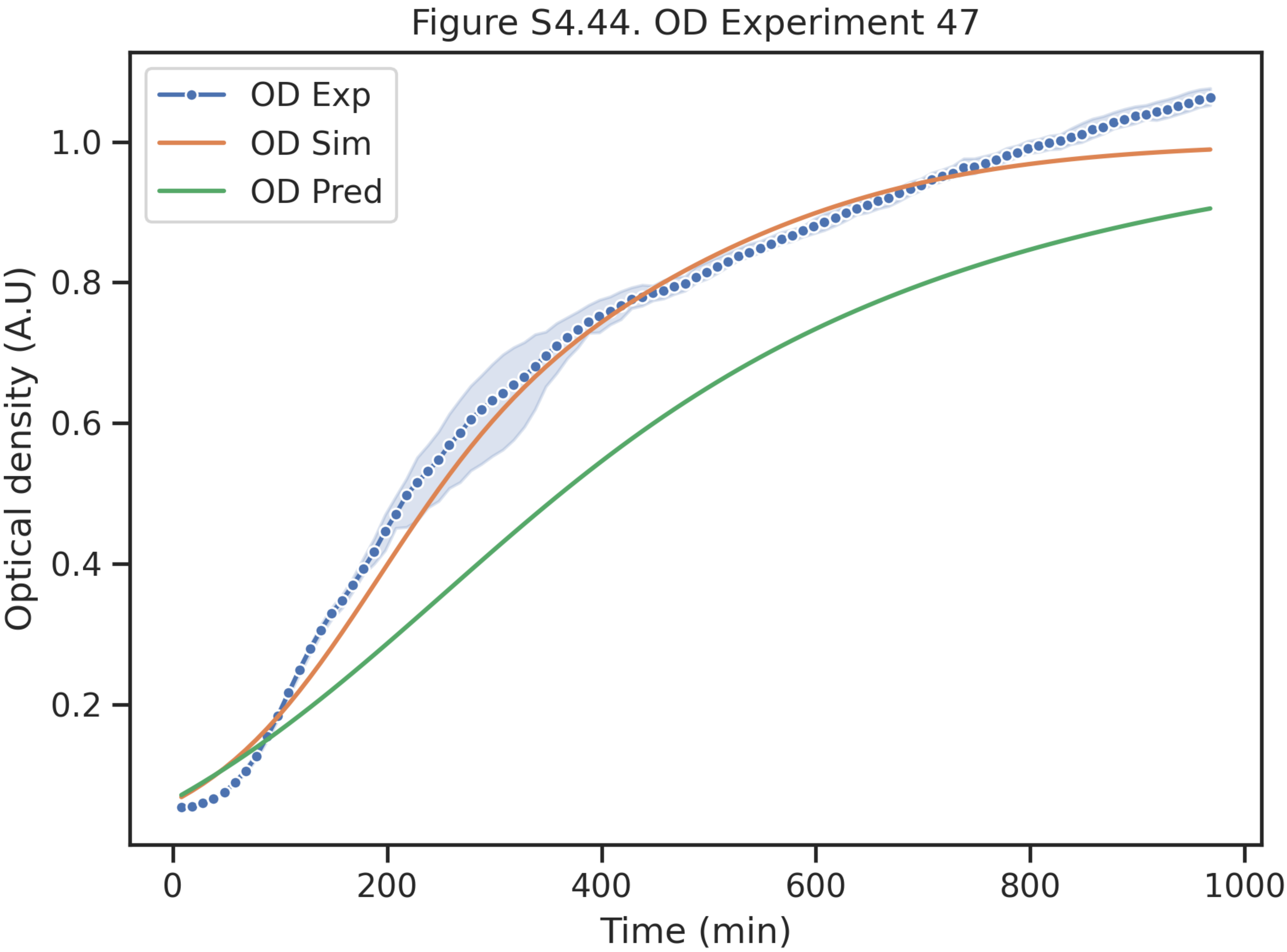
Figure S4.44. OD Experiment 47
OD Exp
OD Sim
OD Pred
Optical density (A.U)
1.0
0.8
0.6
0.4
0.2
0
200
400
600
800
1000
Time (min)

Figure S4.45. OD Experiment 48

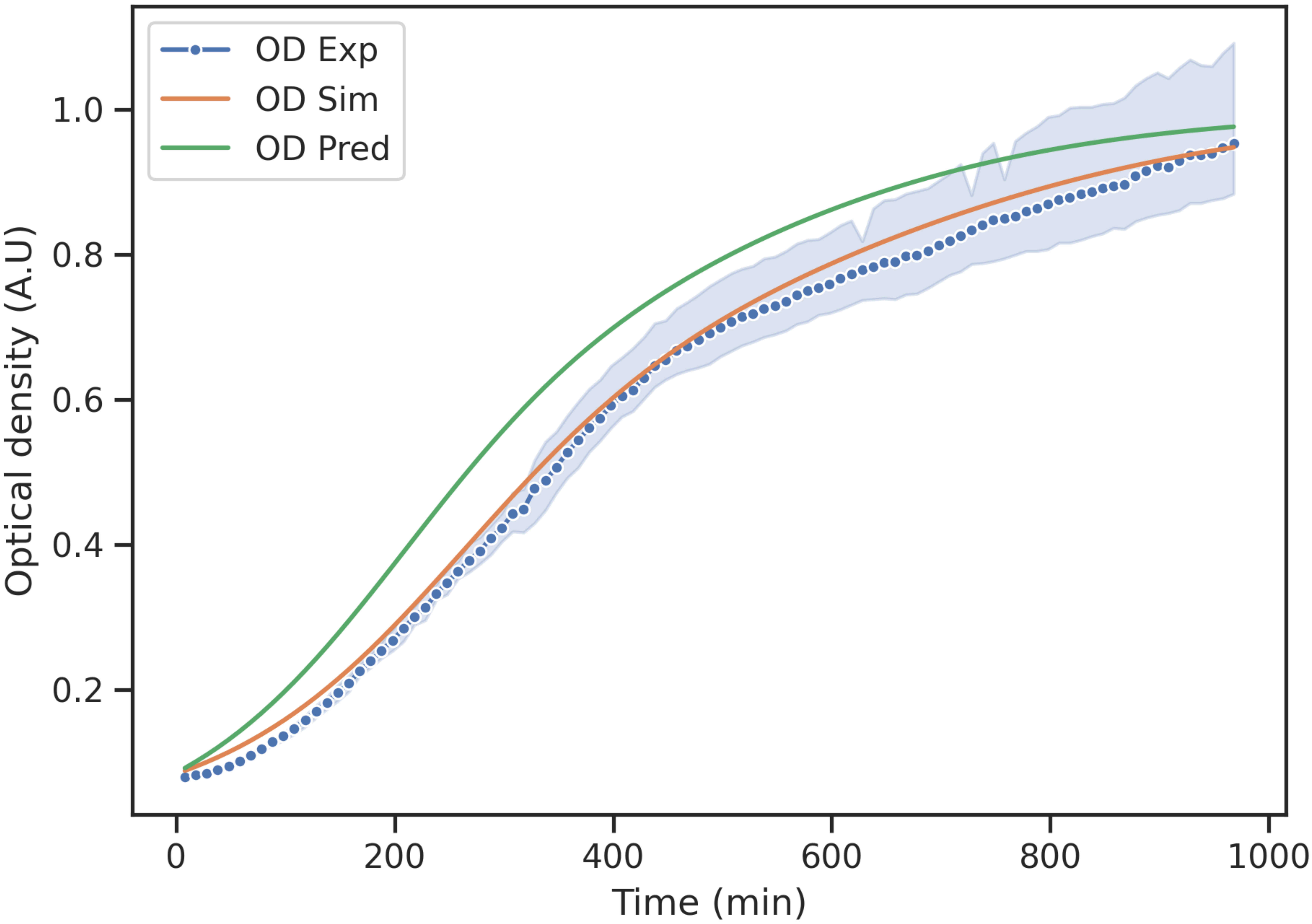

Supplementary Figure S5. Experimental (blue dotted) vs simulated (orange) vs predicted (green) responses of the biosensor GFP/OD for the experiments, with leave-one-out validation. Bands are shown for the experimental errors, as well as for the ensemble simulations and predictions. Experiment 5 from the DoE library was removed from the set due to assembly issues resulting in a non-functional response).

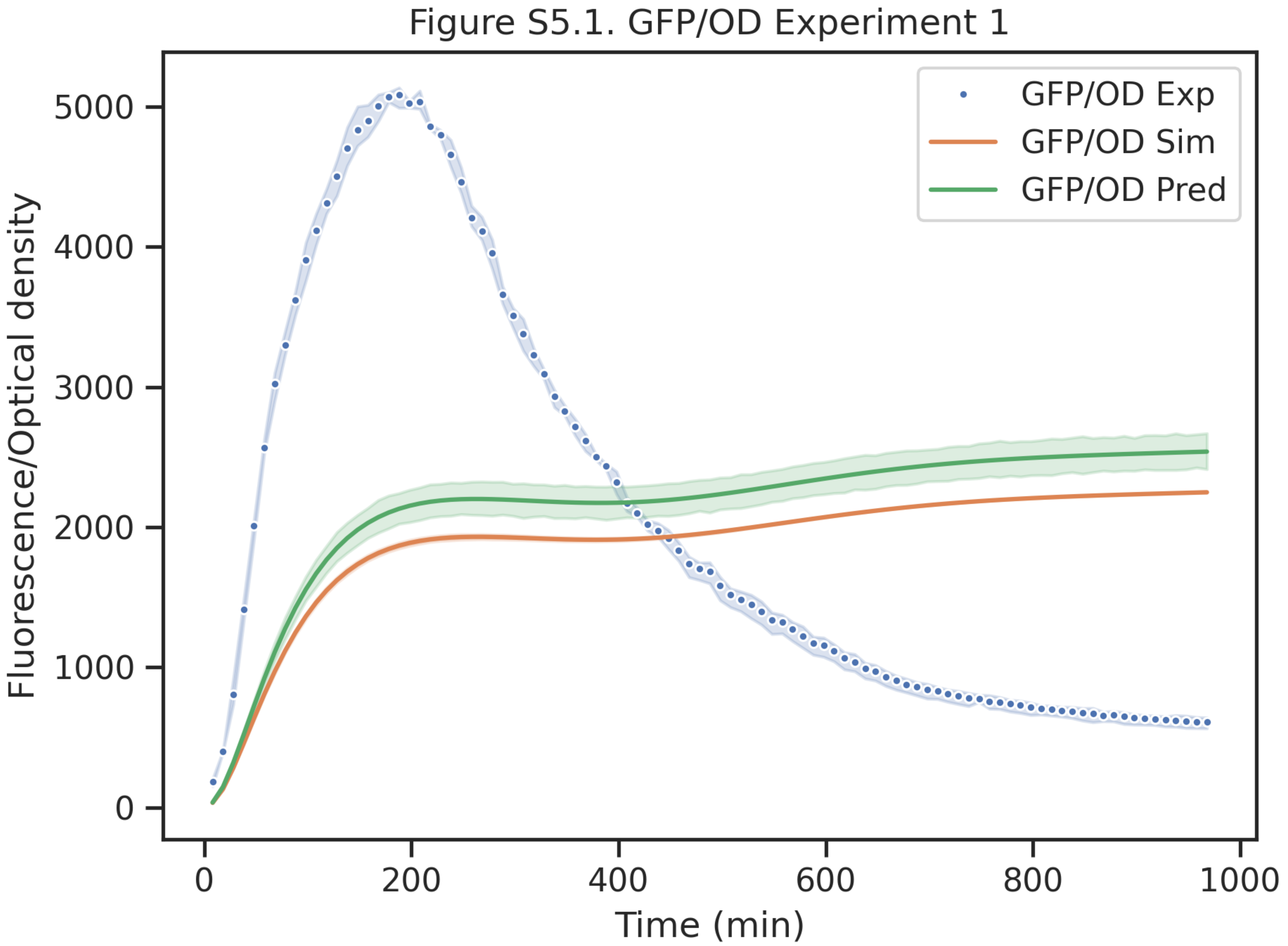
Figure S5.1. GFP/OD Experiment 1
GFP/OD Exp
GFP/OD Sim
GFP/OD Pred
Fluorescence/Optical density
Time (min)
0
1000
2000
3000
4000
5000
0
200
400
600
800
1000

Figure S5.2. GFP/OD Experiment 2

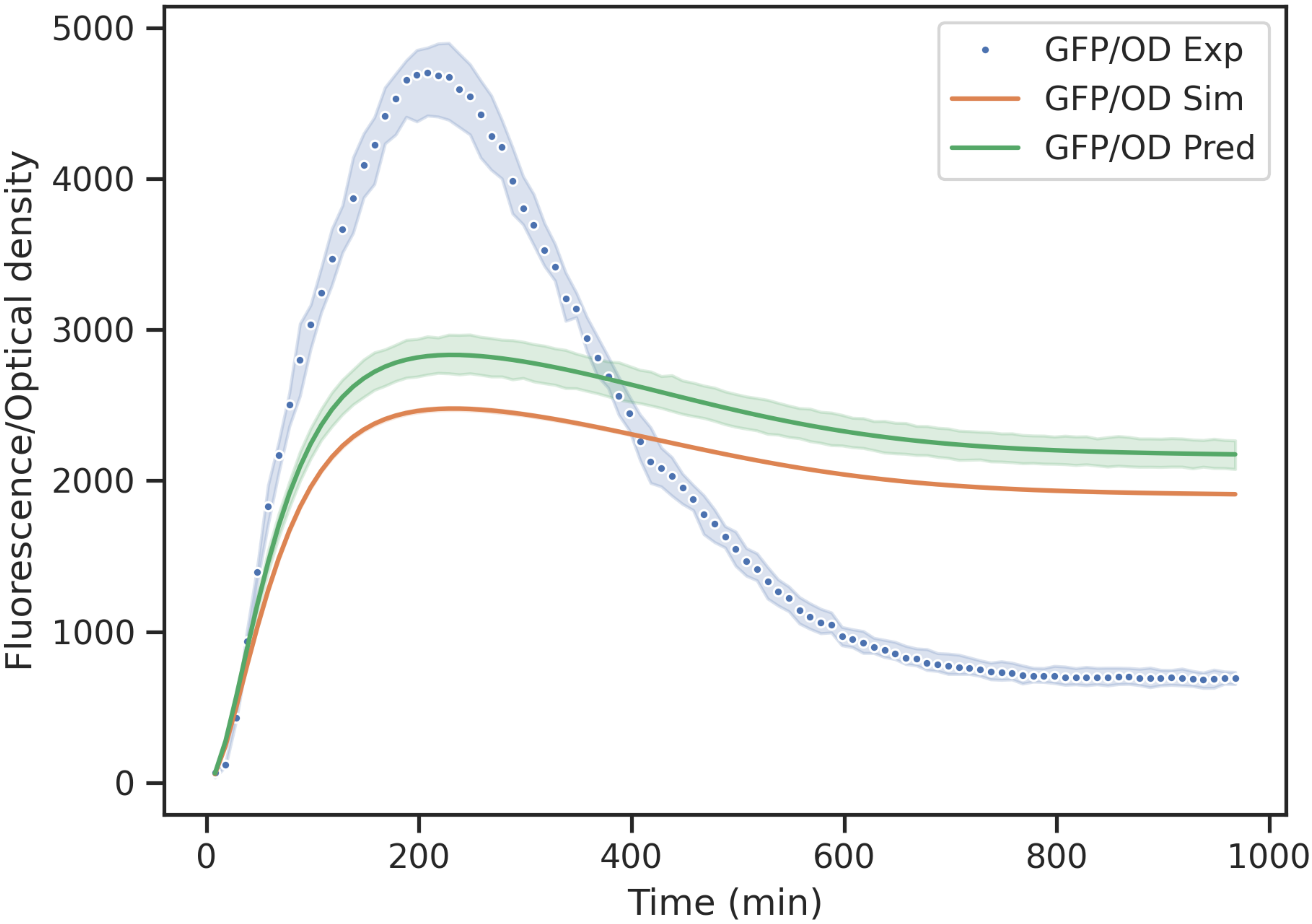

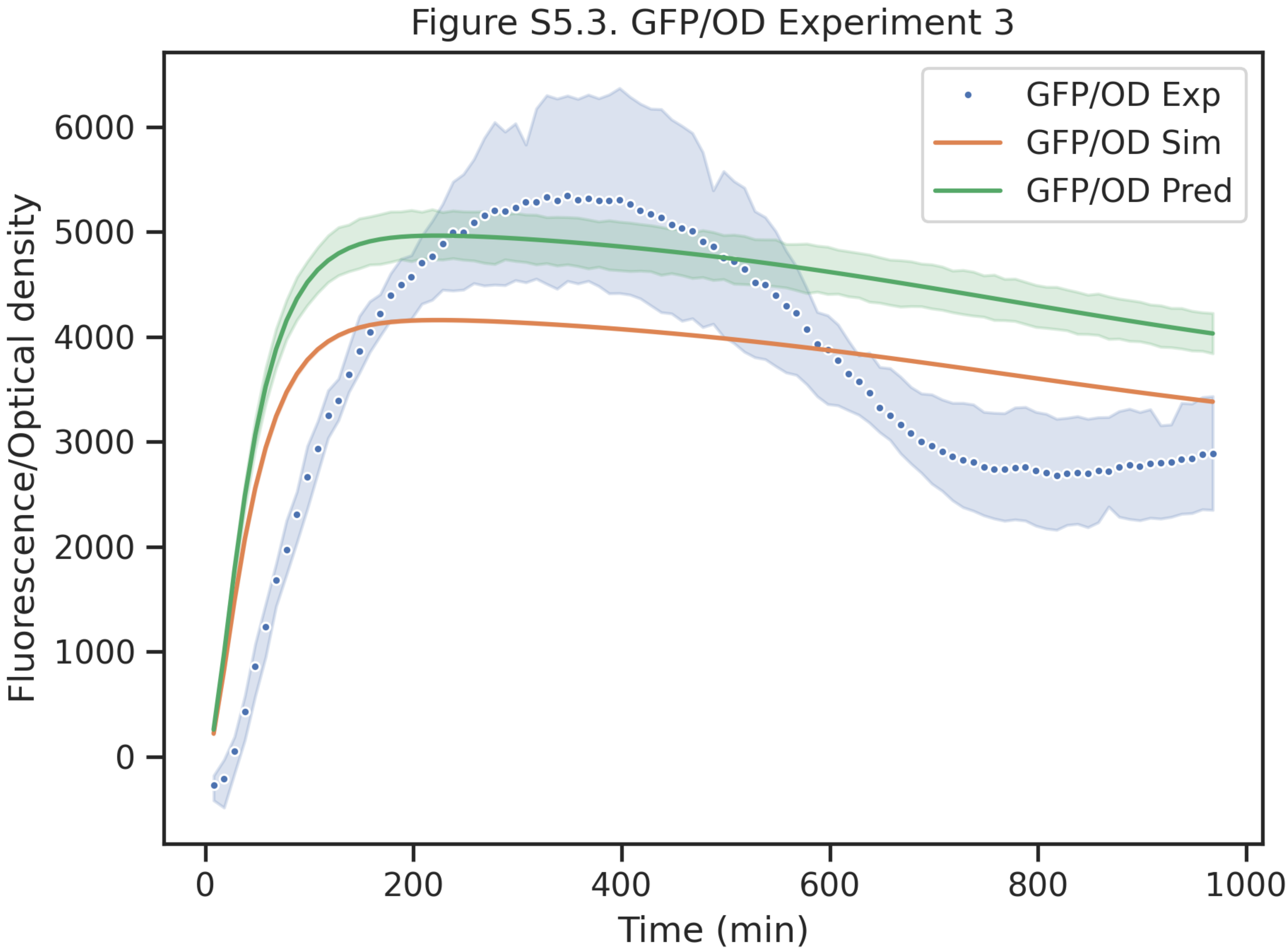
Figure S5.3. GFP/OD Experiment 3
GFP/OD Exp
GFP/OD Sim
GFP/OD Pred
Fluorescence/Optical density
Time (min)
0
1000
2000
3000
4000
5000
6000
0
200
400
600
800
1000

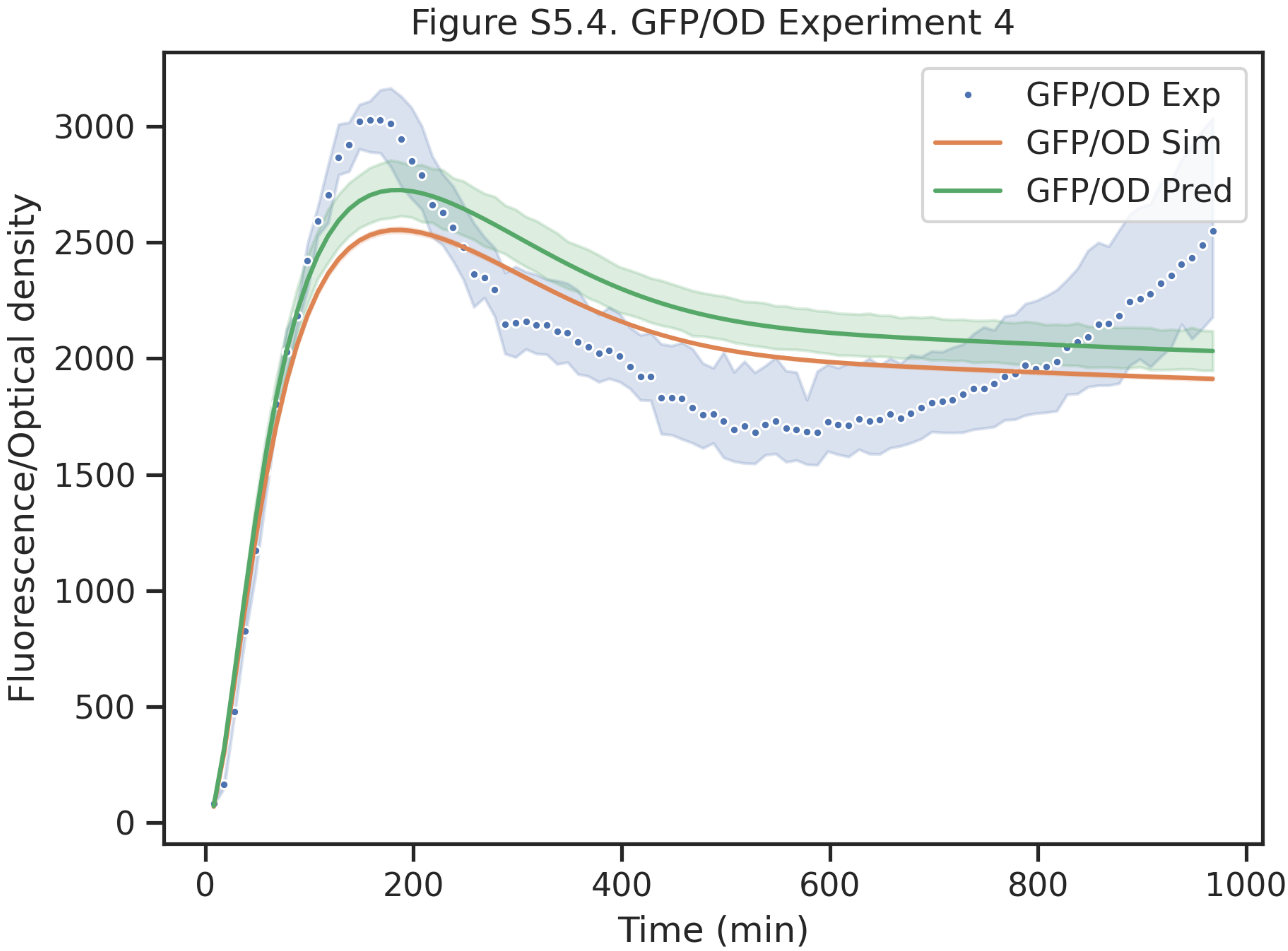
Figure S5.4. GFP/OD Experiment 4
GFP/OD Exp
GFP/OD Sim
GFP/OD Pred
Fluorescence/Optical density
Time (min)
0
500
1000
1500
2000
2500
3000
0
200
400
600
800
1000

Figure S5.5. GFP/OD Experiment 5

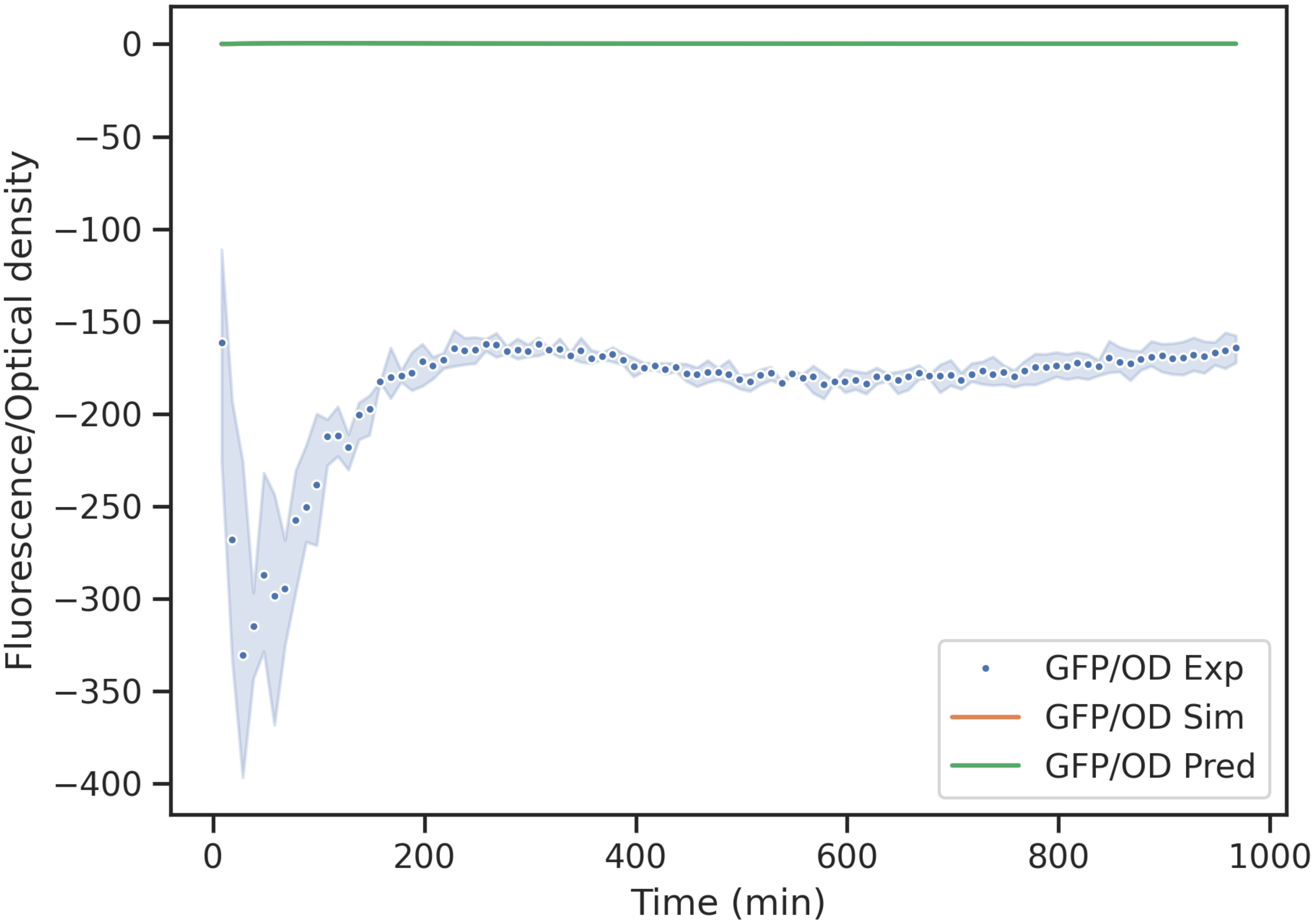

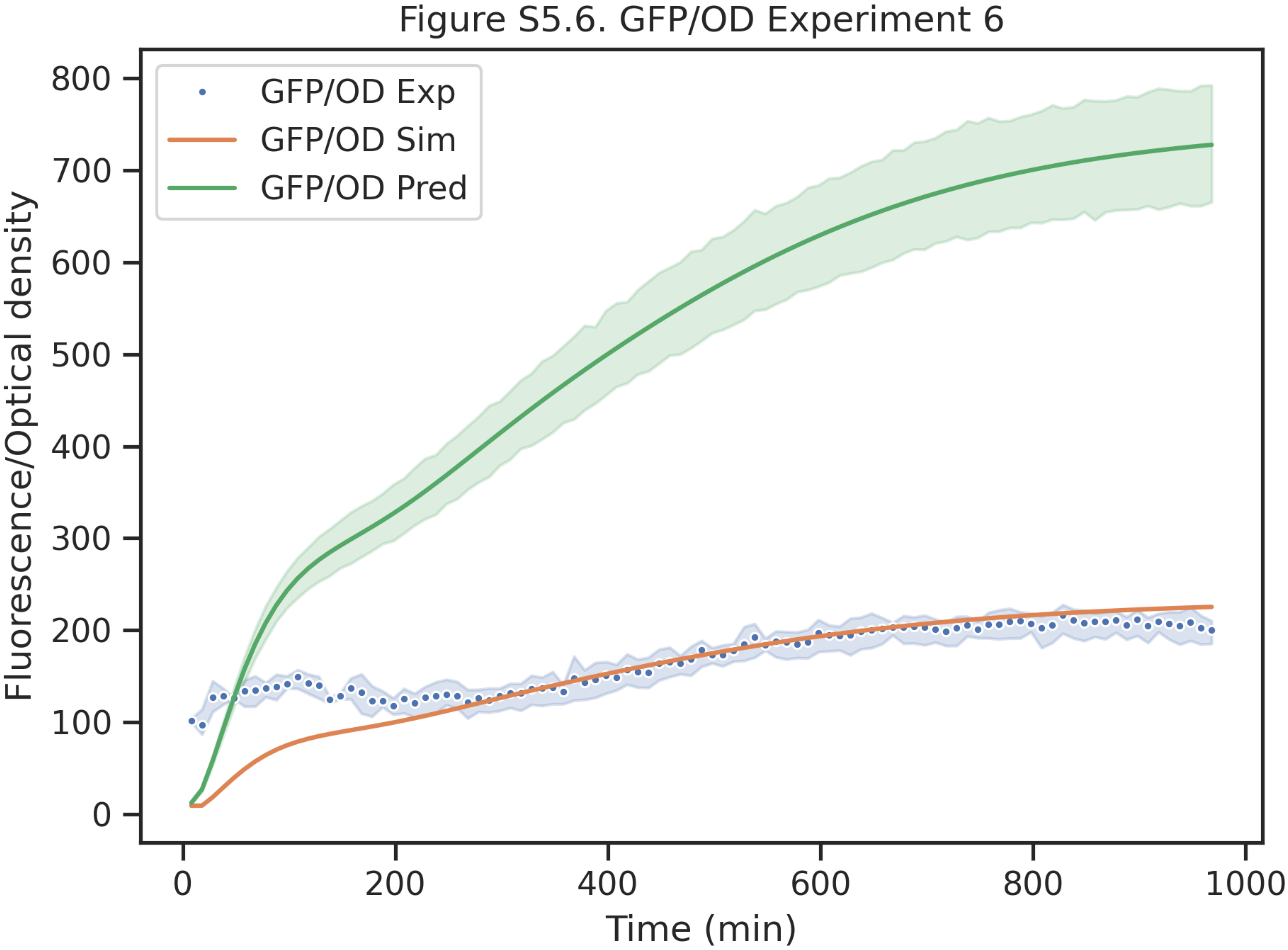
Figure S5.6. GFP/OD Experiment 6
GFP/OD Exp
GFP/OD Sim
GFP/OD Pred
Fluorescence/Optical density
Time (min)
0
100
200
300
400
500
600
700
800
0
200
400
600
800
1000

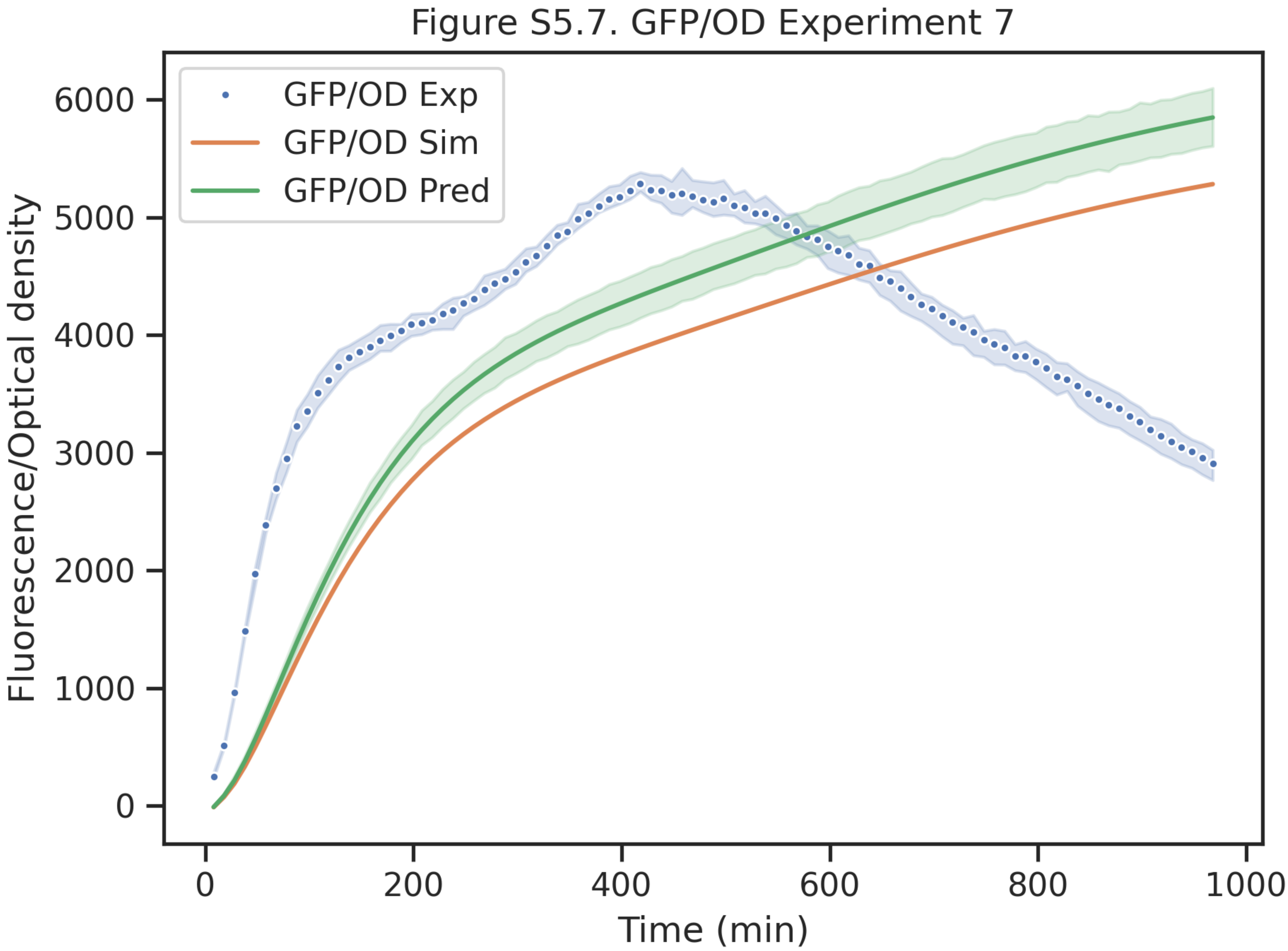
Figure S5.7. GFP/OD Experiment 7
GFP/OD Exp
GFP/OD Sim
GFP/OD Pred
Fluorescence/Optical density
Time (min)
0
1000
2000
3000
4000
5000
6000
0
200
400
600
800
1000

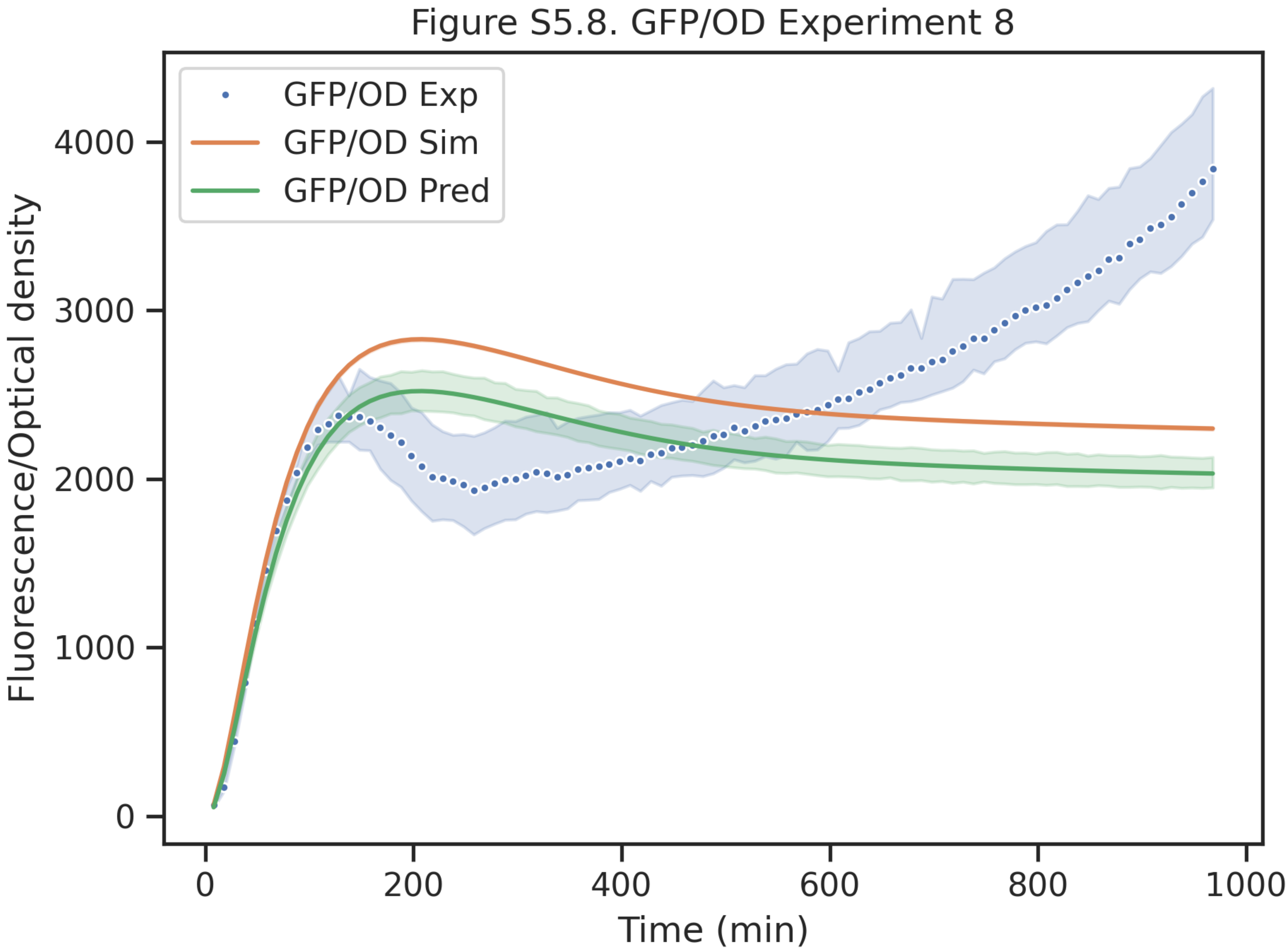
Figure S5.8. GFP/OD Experiment 8
GFP/OD Exp
GFP/OD Sim
GFP/OD Pred
Fluorescence/Optical density
0
1000
2000
3000
4000
0
200
400
600
800
1000
Time (min)

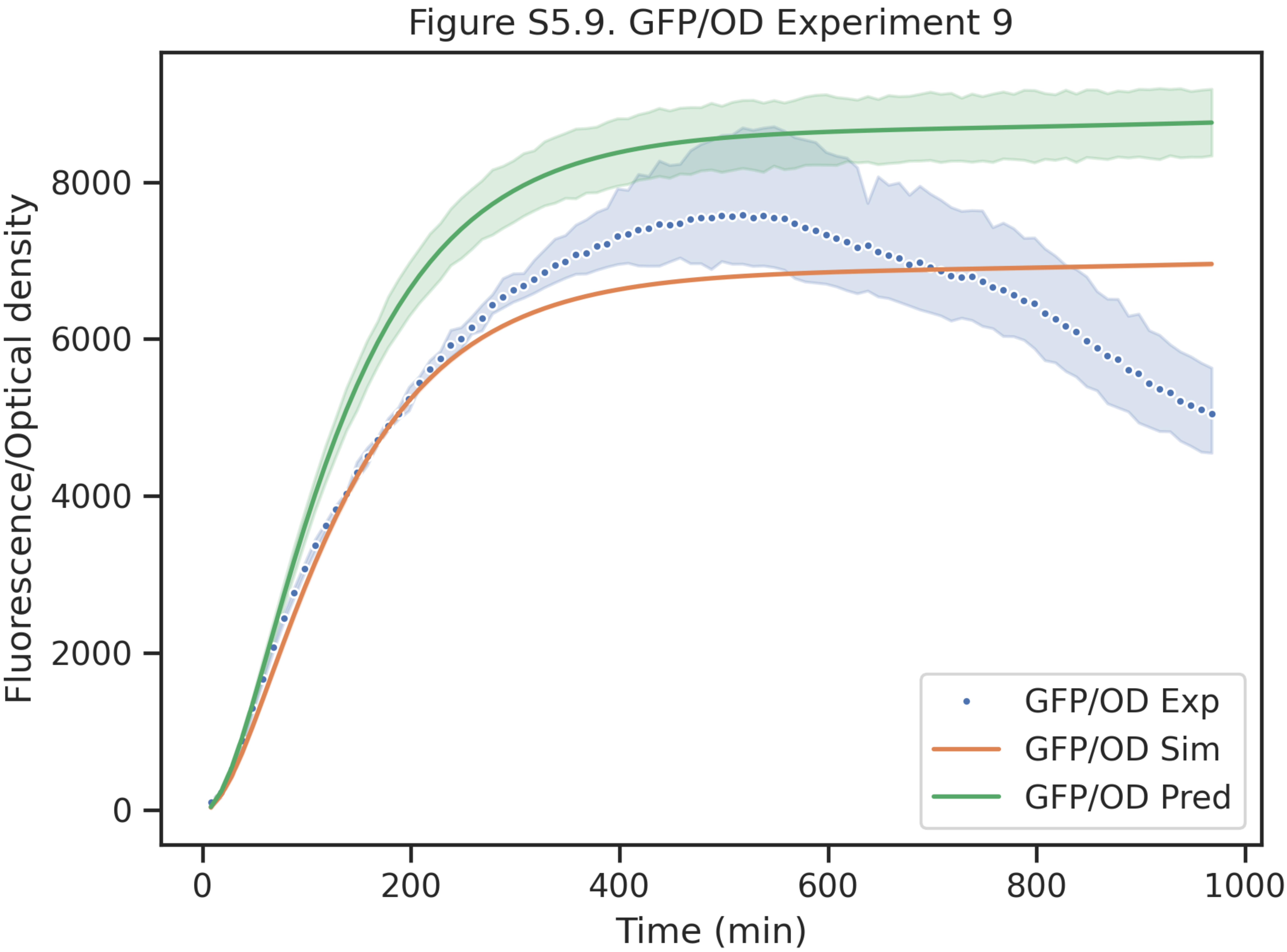

Figure S5.9. GFP/OD Experiment 9
Fluorescence/Optical density
Time (min)
0
2000
4000
6000
8000
0
200
400
600
800
1000
GFP/OD Exp
GFP/OD Sim
GFP/OD Pred

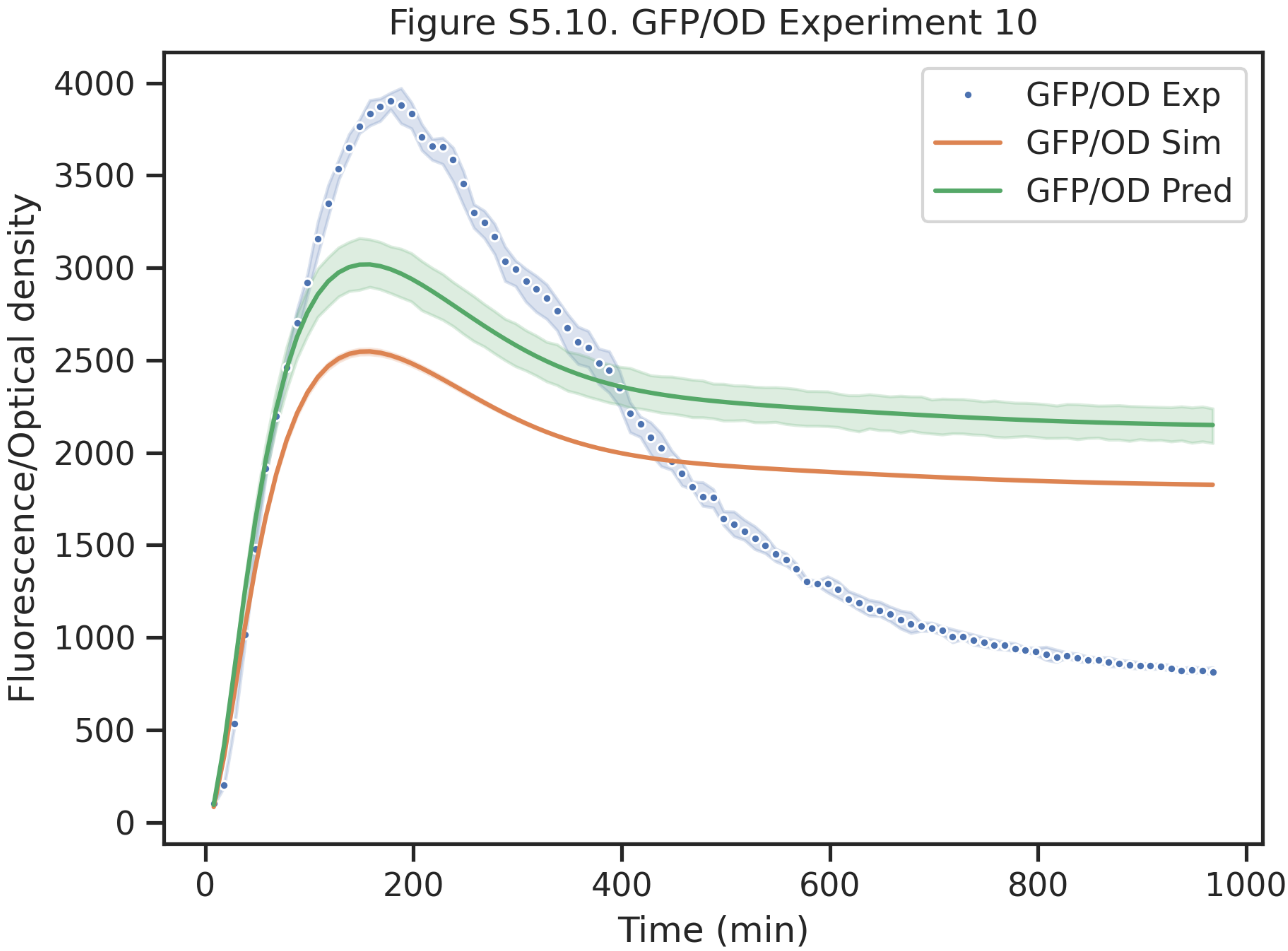
Figure S5.10. GFP/OD Experiment 10
Fluorescence/Optical density
Time (min)
GFP/OD Exp
GFP/OD Sim
GFP/OD Pred
0
500
1000
1500
2000
2500
3000
3500
4000
0
200
400
600
800
1000

Figure S5.11. GFP/OD Experiment 11

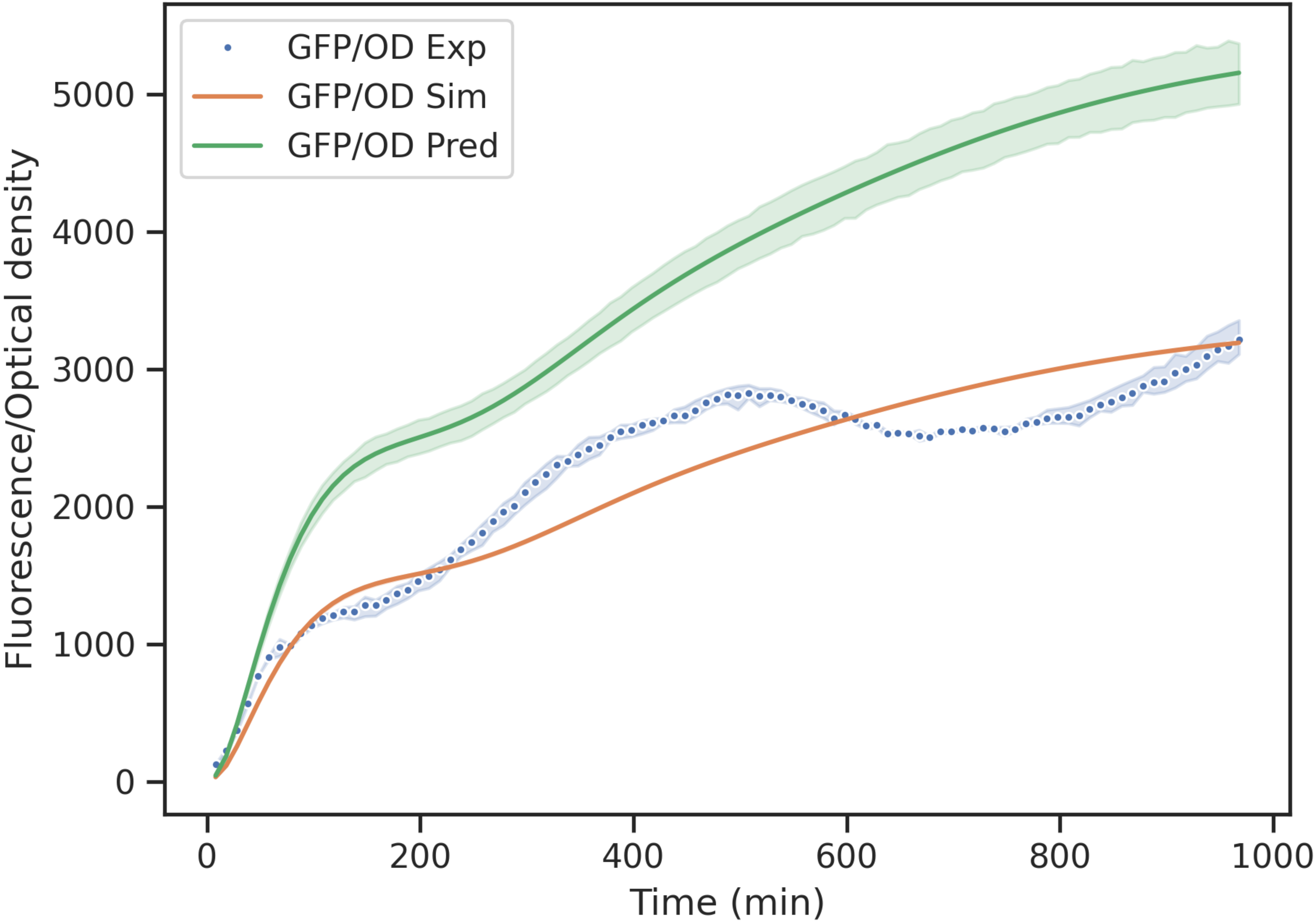

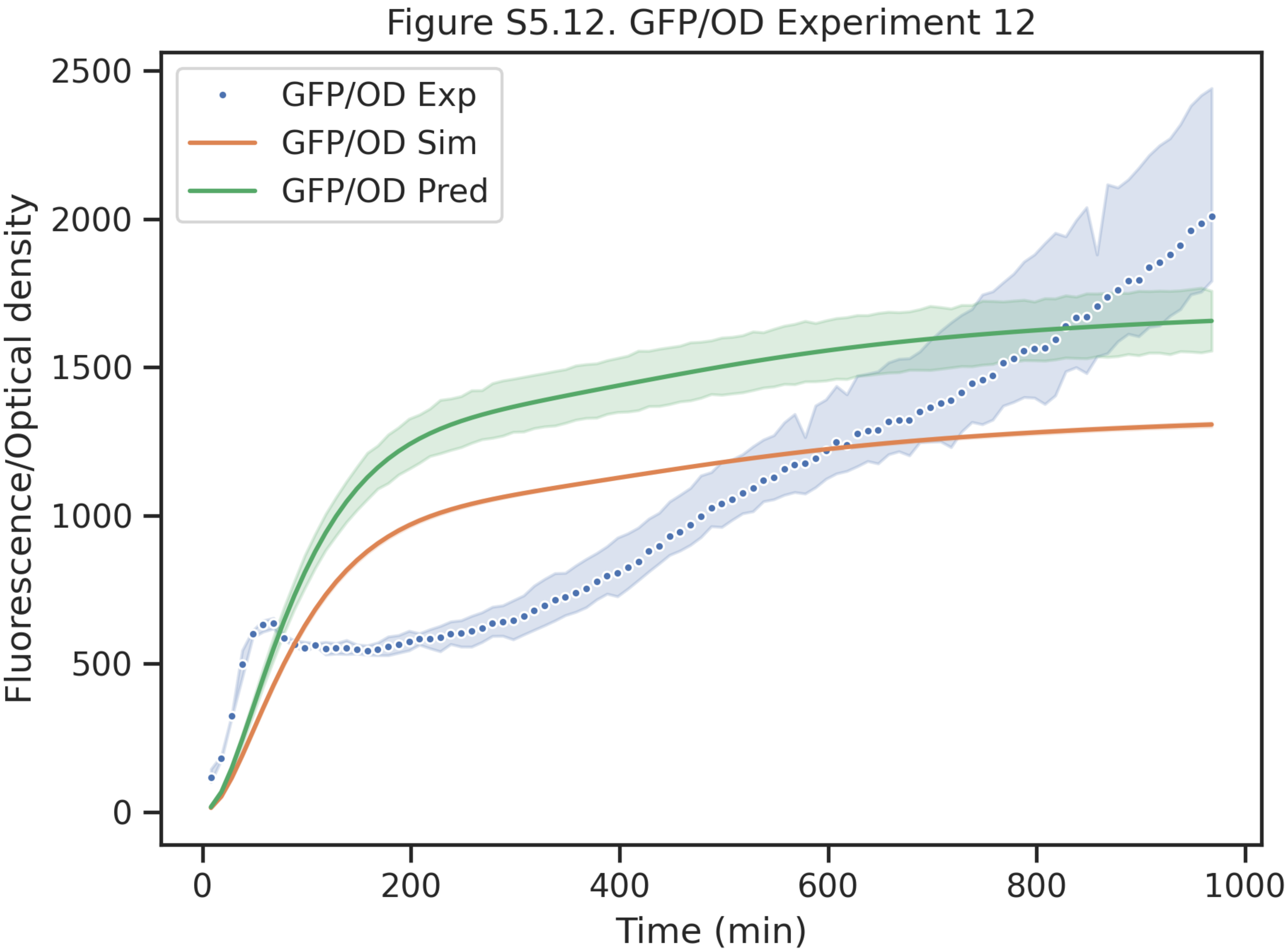

Figure S5.12. GFP/OD Experiment 12
GFP/OD Exp
GFP/OD Sim
GFP/OD Pred
Fluorescence/Optical density
Time (min)
0
500
1000
1500
2000
2500
0
200
400
600
800
1000

Figure S5.13. GFP/OD Experiment 13

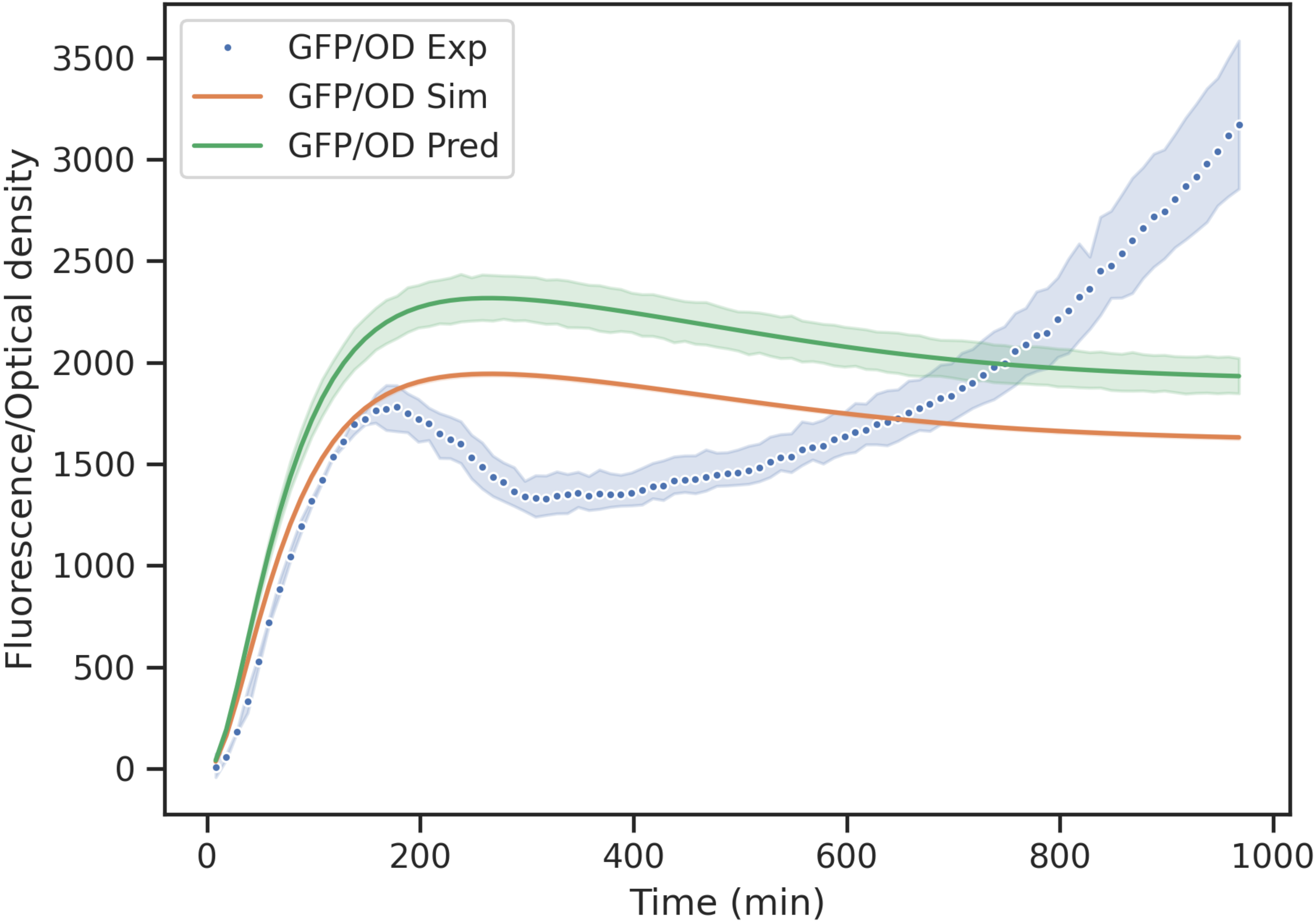

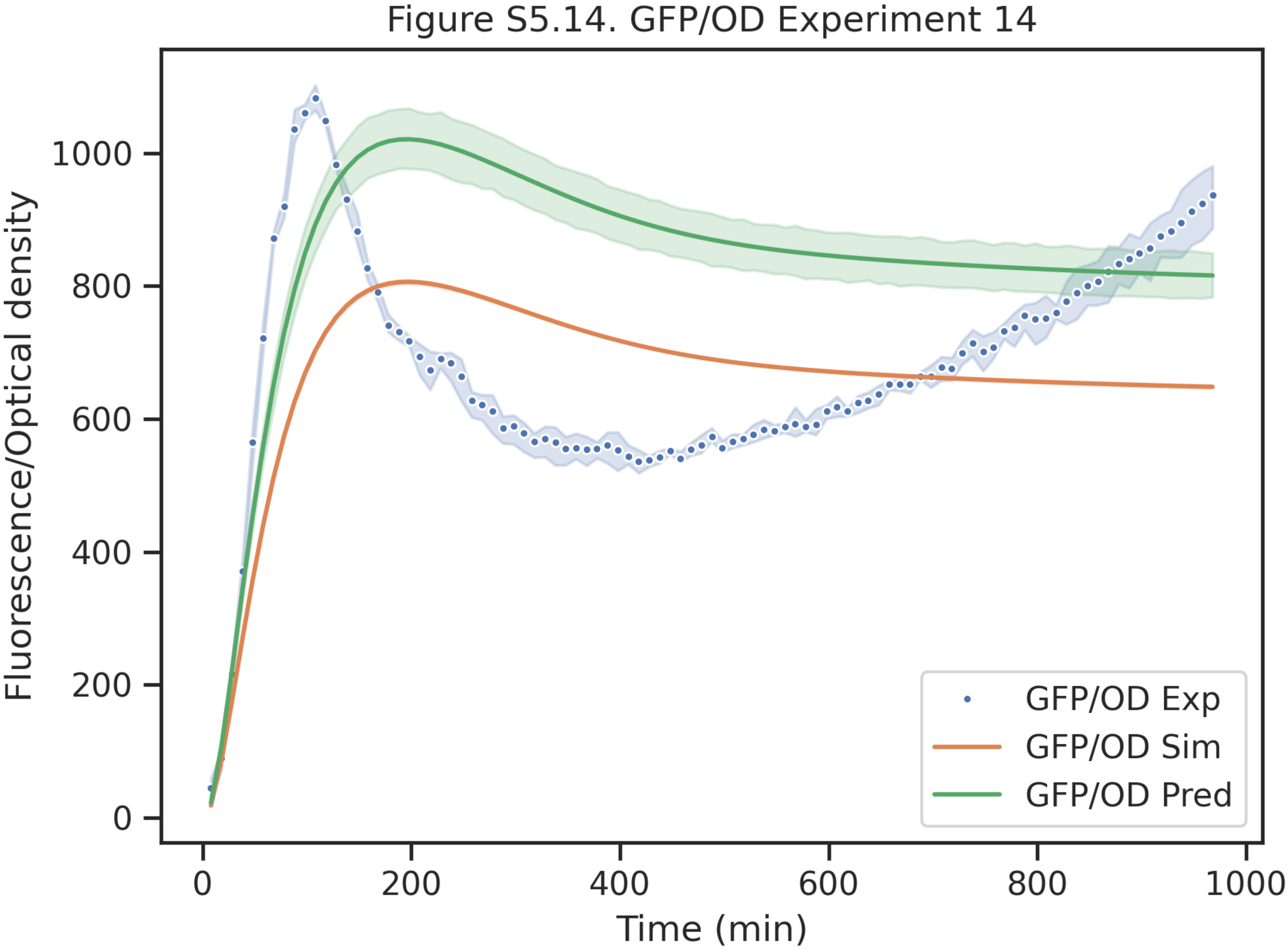
Figure S5.14. GFP/OD Experiment 14
Fluorescence/Optical density
Time (min)
0
200
400
600
800
1000
0
200
400
600
800
1000
GFP/OD Exp
GFP/OD Sim
GFP/OD Pred

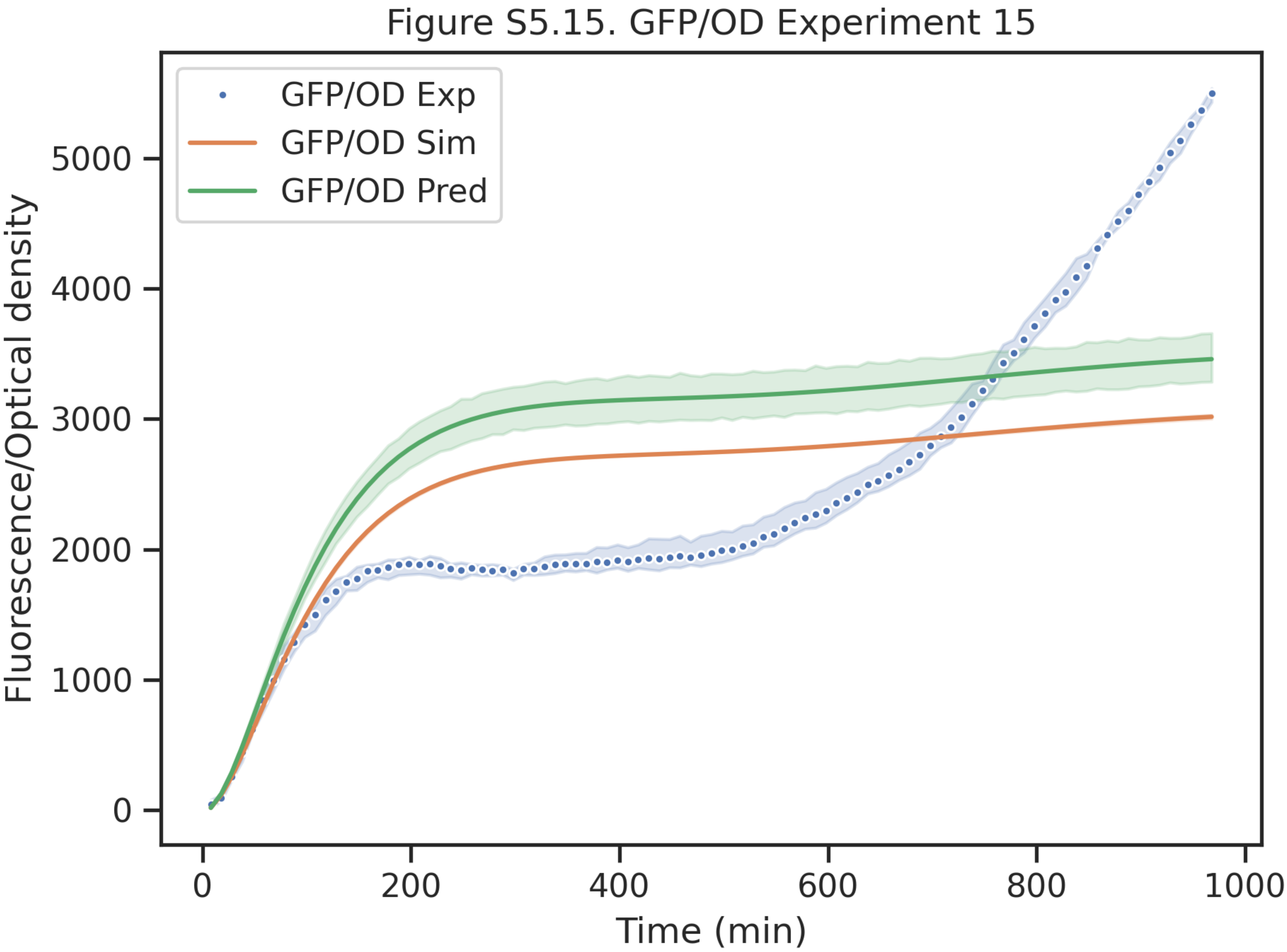
Figure S5.15. GFP/OD Experiment 15
GFP/OD Exp
GFP/OD Sim
GFP/OD Pred
Fluorescence/Optical density
0
1000
2000
3000
4000
5000
Time (min)
0
200
400
600
800
1000

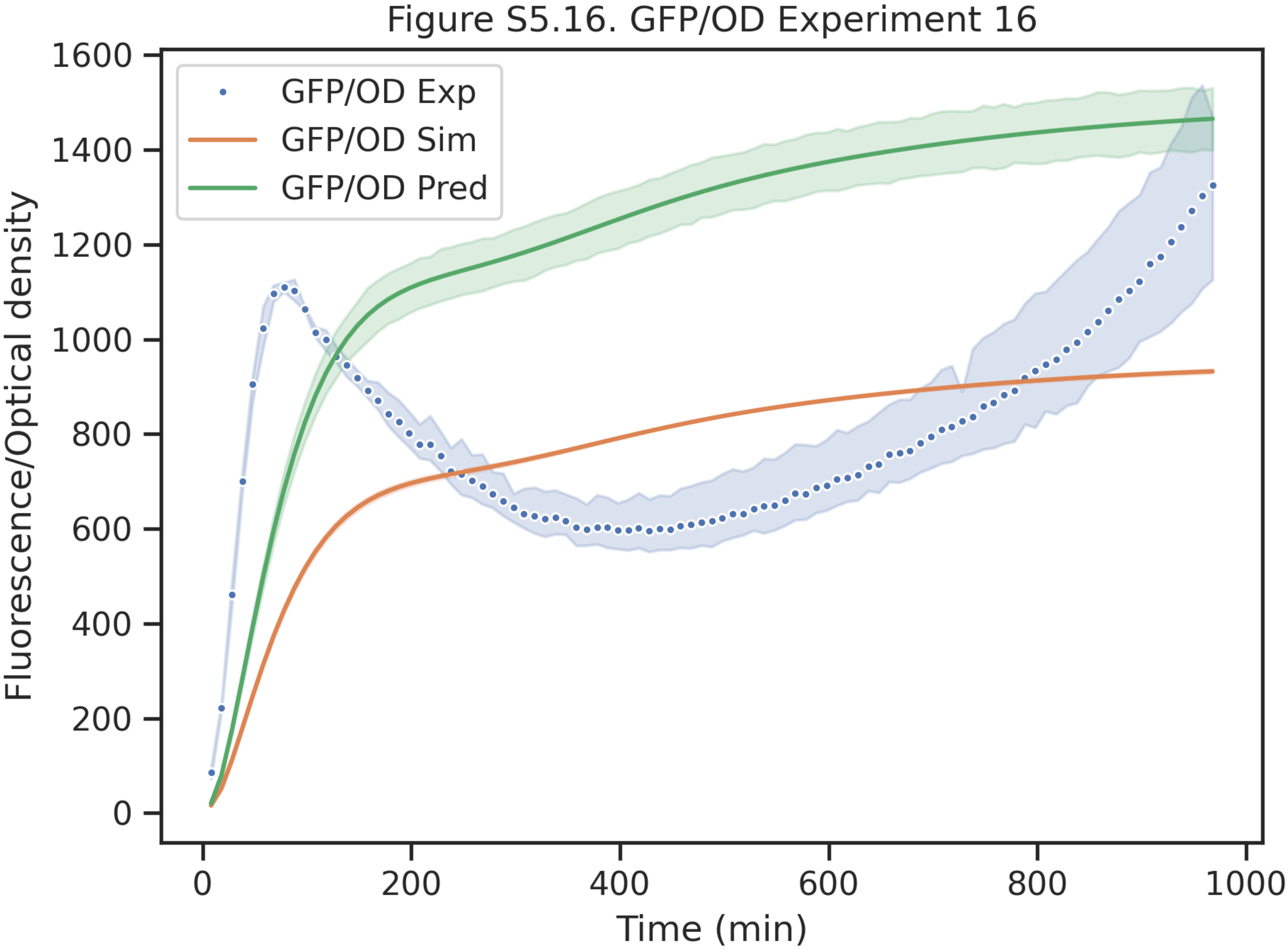

Figure S5.16. GFP/OD Experiment 16
GFP/OD Exp
GFP/OD Sim
GFP/OD Pred
Fluorescence/Optical density
Time (min)
0
200
400
600
800
1000
1200
1400
1600
0
200
400
600
800
1000

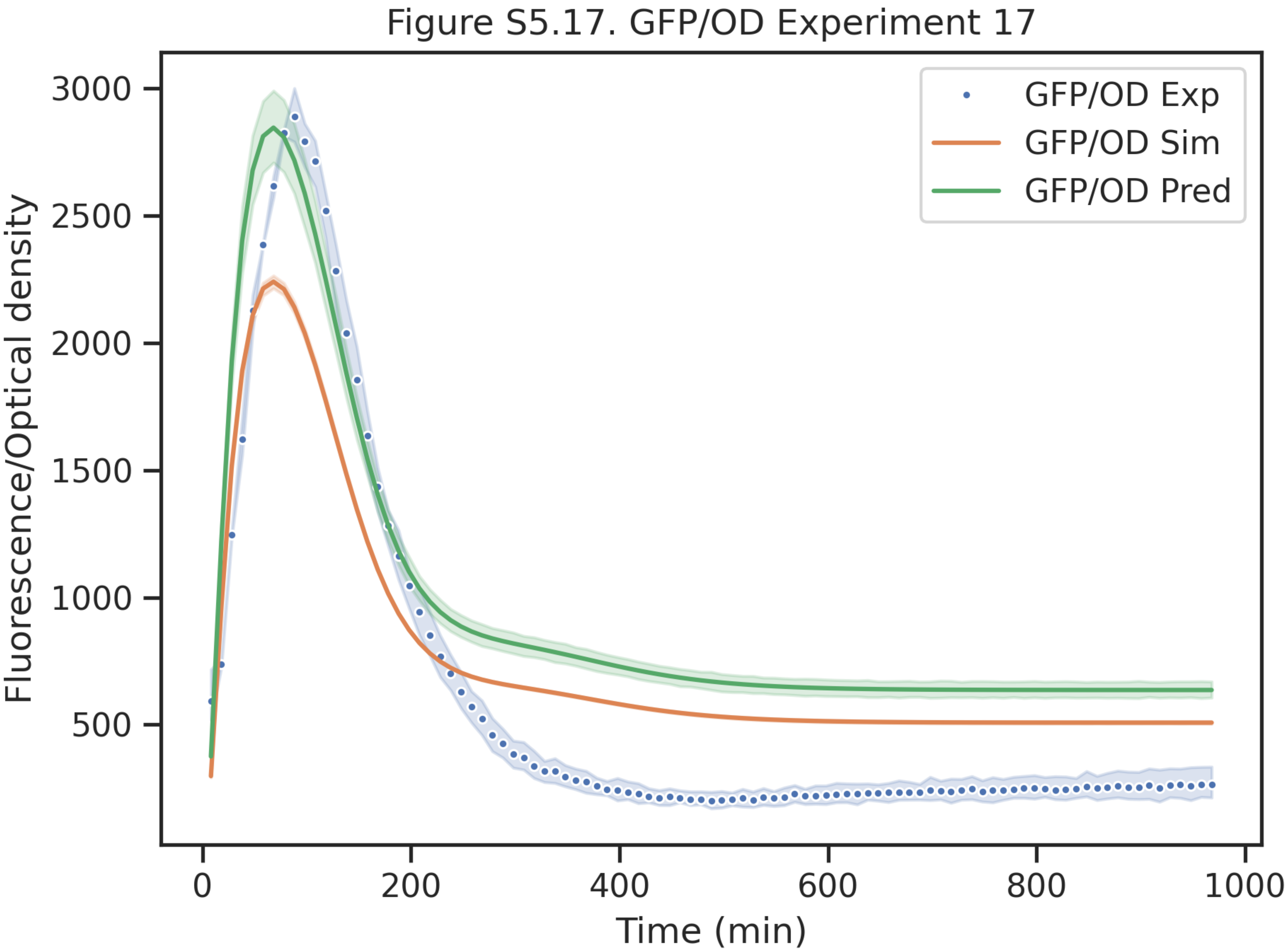
Figure S5.17. GFP/OD Experiment 17
GFP/OD Exp
GFP/OD Sim
GFP/OD Pred
Fluorescence/Optical density
Time (min)
3000
2500
2000
1500
1000
500
0
200
400
600
800
1000

Figure S5.18. GFP/OD Experiment 18

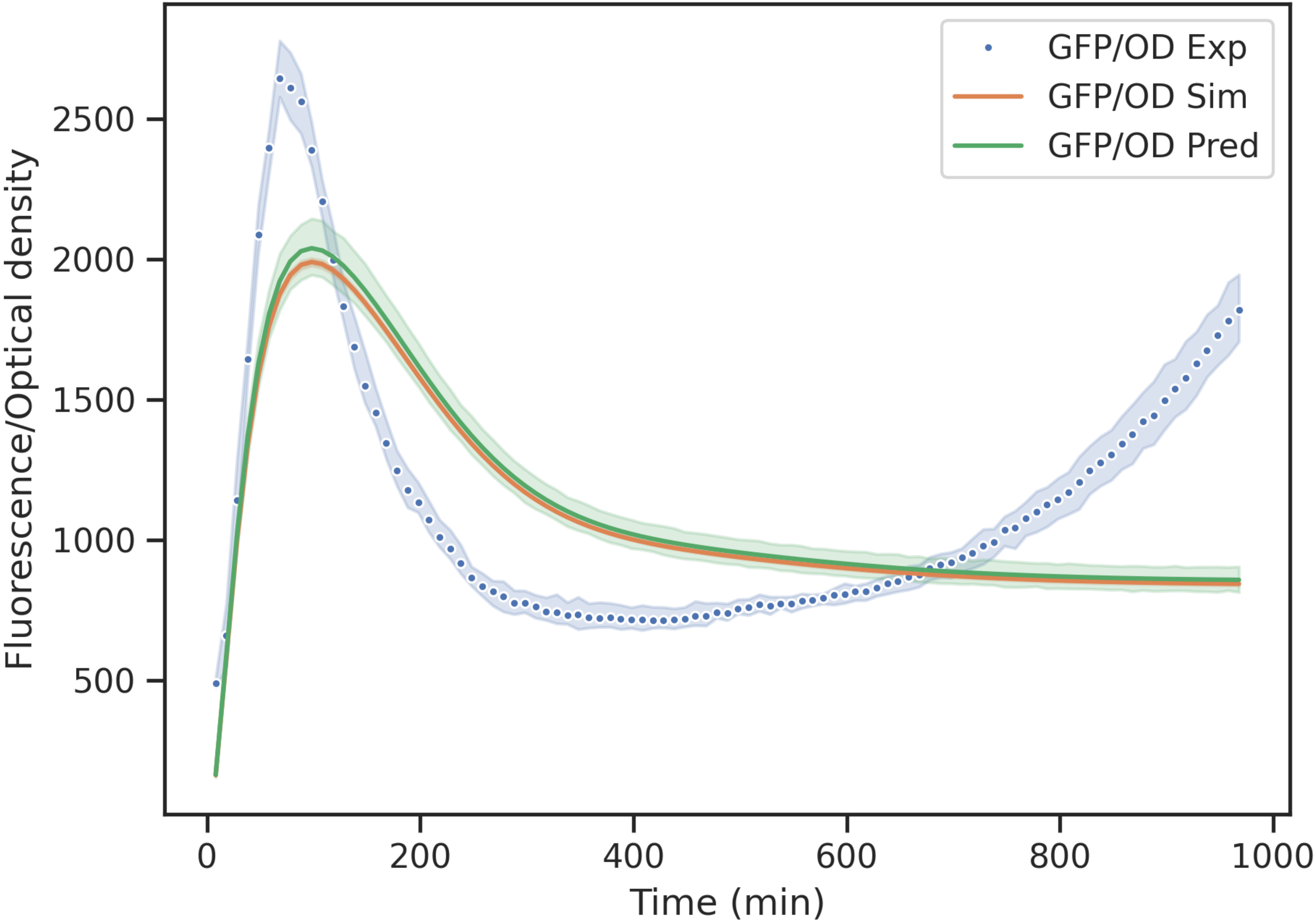

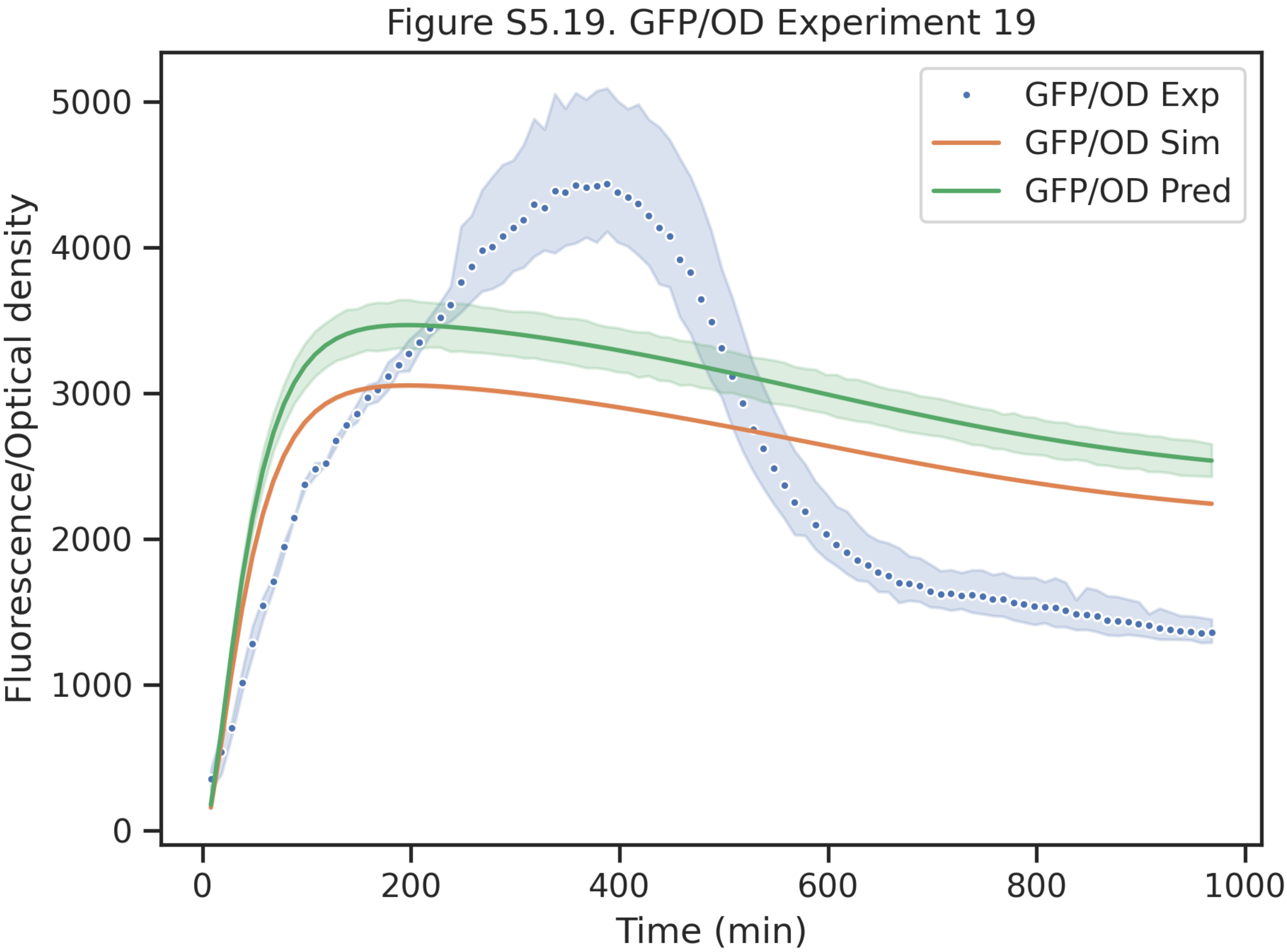
Figure S5.19. GFP/OD Experiment 19
GFP/OD Exp
GFP/OD Sim
GFP/OD Pred
Fluorescence/Optical density
Time (min)
0
1000
2000
3000
4000
5000
0
200
400
600
800
1000

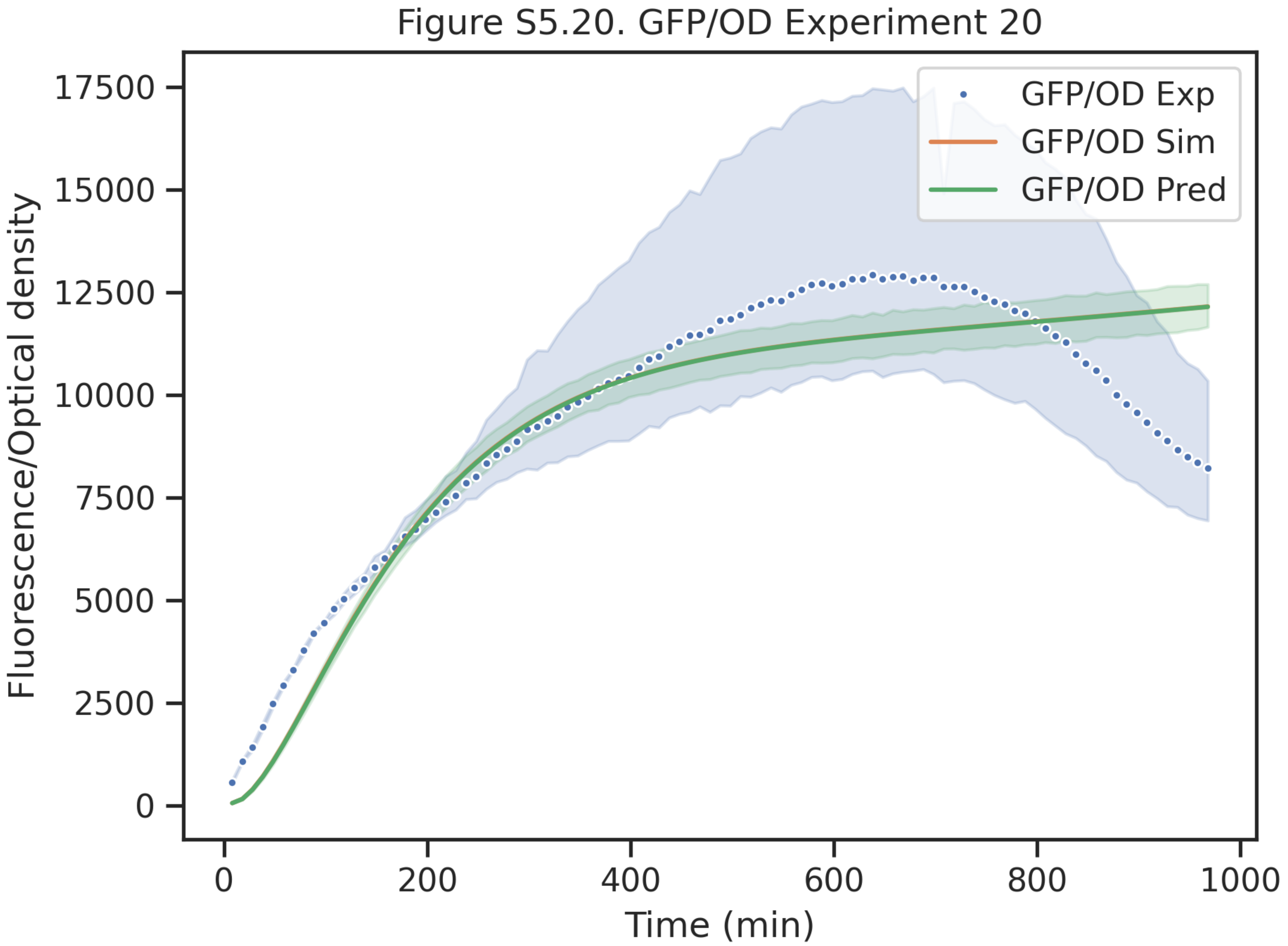
Figure S5.20. GFP/OD Experiment 20
GFP/OD Exp
GFP/OD Sim
GFP/OD Pred
Fluorescence/Optical density
Time (min)
0
2500
5000
7500
10000
12500
15000
17500
0
200
400
600
800
1000

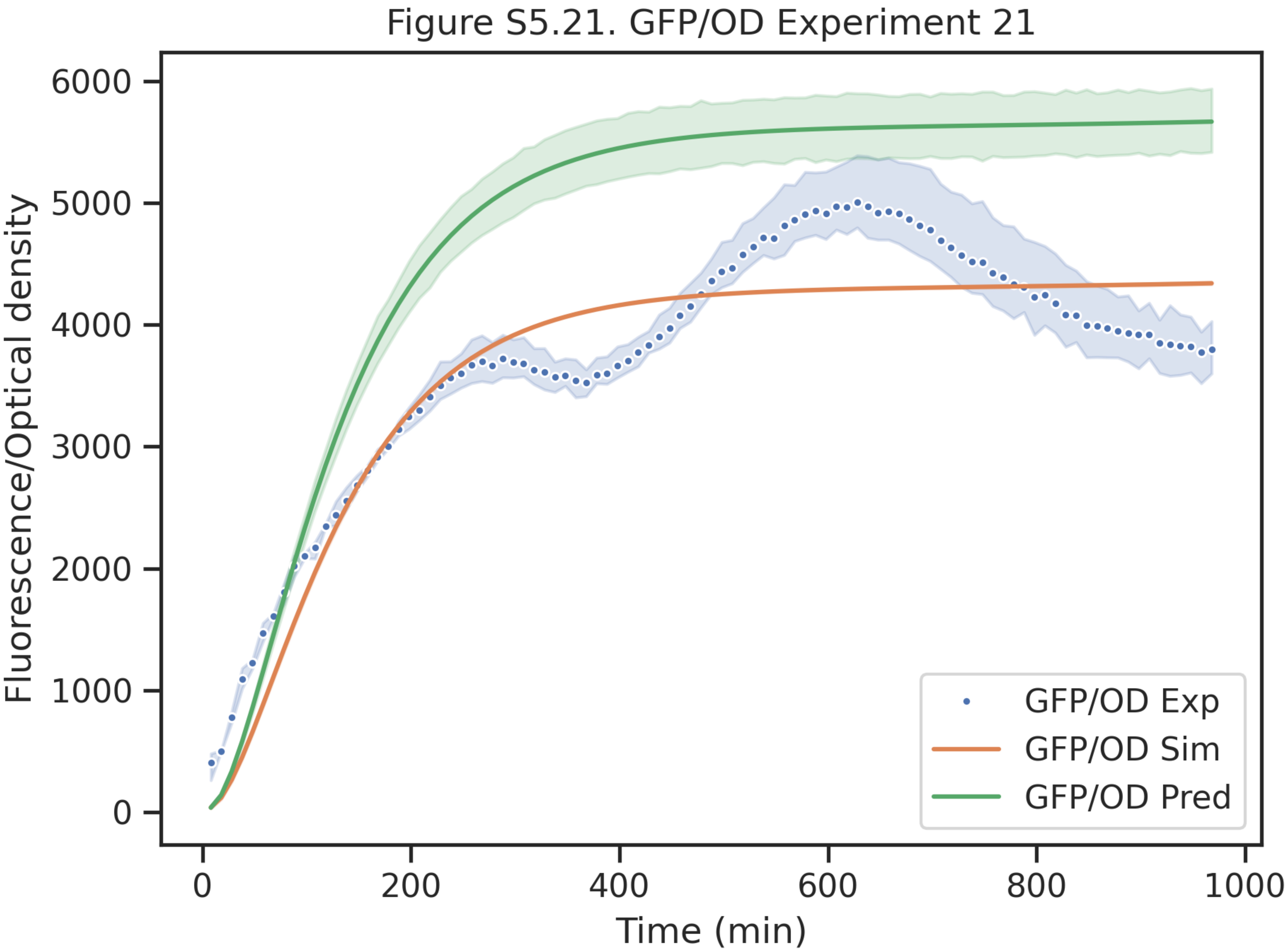
Figure S5.21. GFP/OD Experiment 21
Fluorescence/Optical density
Time (min)
0
1000
2000
3000
4000
5000
6000
0
200
400
600
800
1000
GFP/OD Exp
GFP/OD Sim
GFP/OD Pred

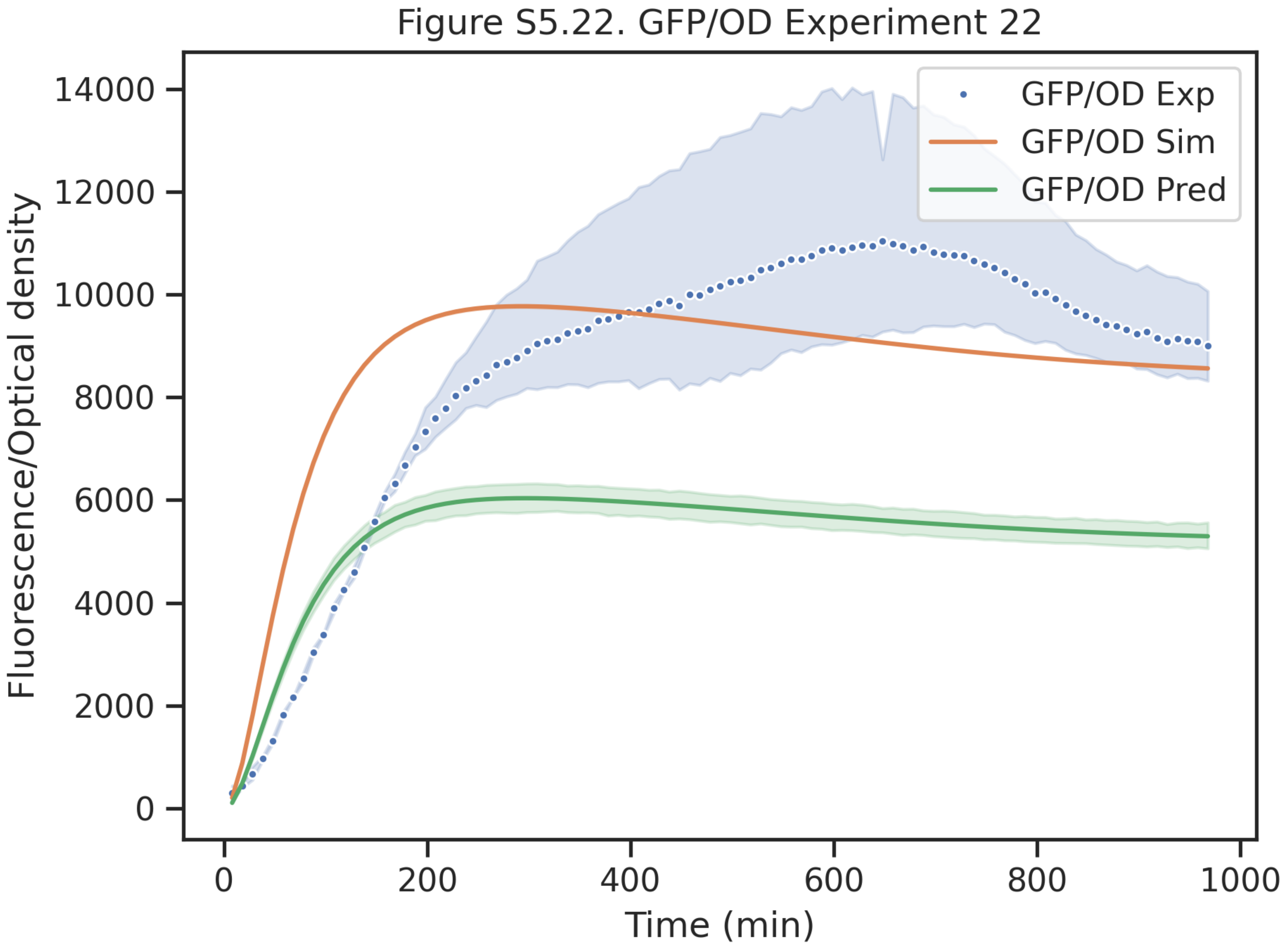
Figure S5.22. GFP/OD Experiment 22
GFP/OD Exp
GFP/OD Sim
GFP/OD Pred
Fluorescence/Optical density
Time (min)
0
2000
4000
6000
8000
10000
12000
14000
0
200
400
600
800
1000

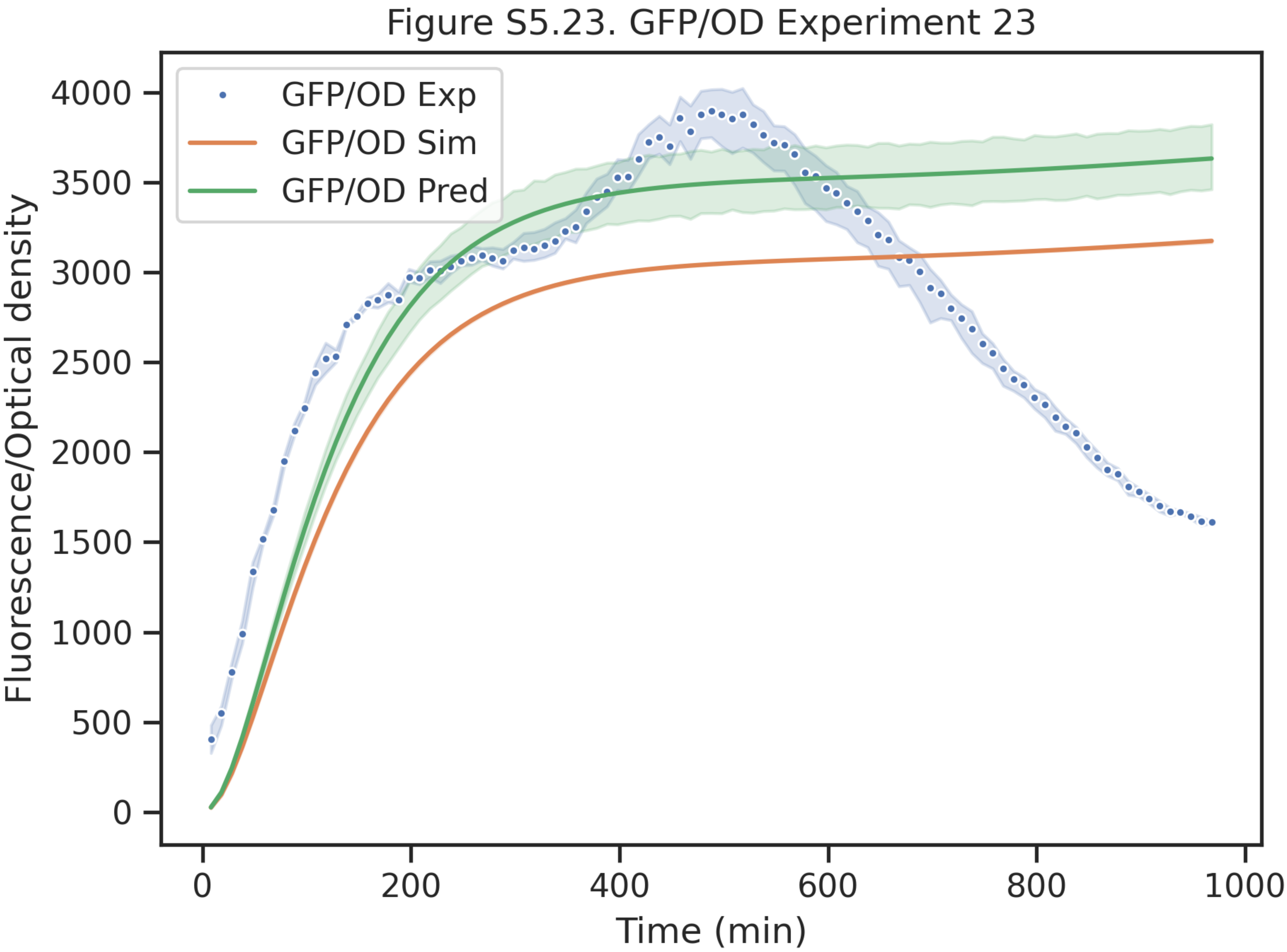
Figure S5.23. GFP/OD Experiment 23
GFP/OD Exp
GFP/OD Sim
GFP/OD Pred
Fluorescence/Optical density
Time (min)
0
500
1000
1500
2000
2500
3000
3500
4000
0
200
400
600
800
1000

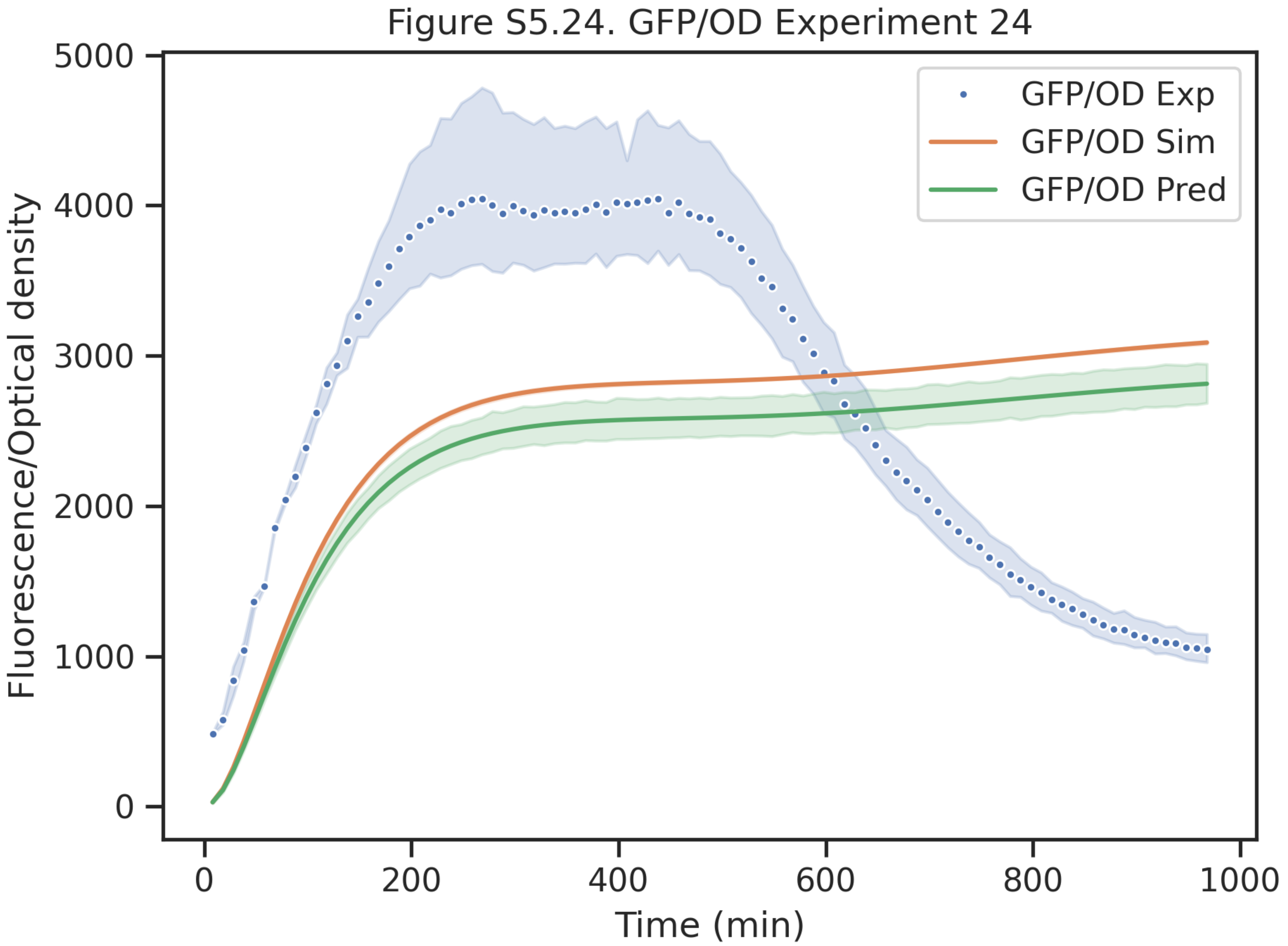

Figure S5.24. GFP/OD Experiment 24
Fluorescence/Optical density
Time (min)
0
1000
2000
3000
4000
5000
0
200
400
600
800
1000
GFP/OD Exp
GFP/OD Sim
GFP/OD Pred

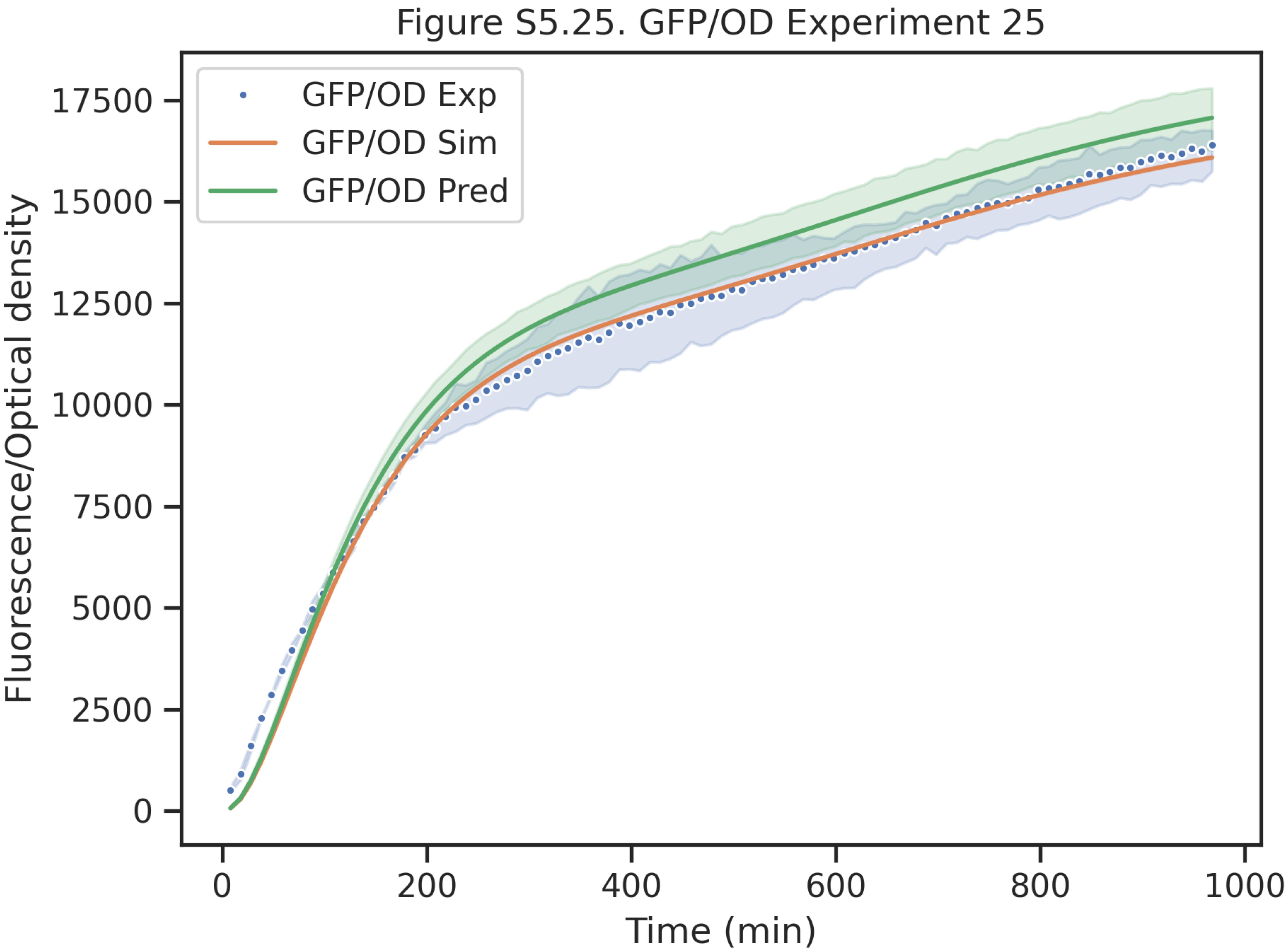
Figure S5.25. GFP/OD Experiment 25
GFP/OD Exp
GFP/OD Sim
GFP/OD Pred
Fluorescence/Optical density
Time (min)
0
2500
5000
7500
10000
12500
15000
17500
0
200
400
600
800
1000

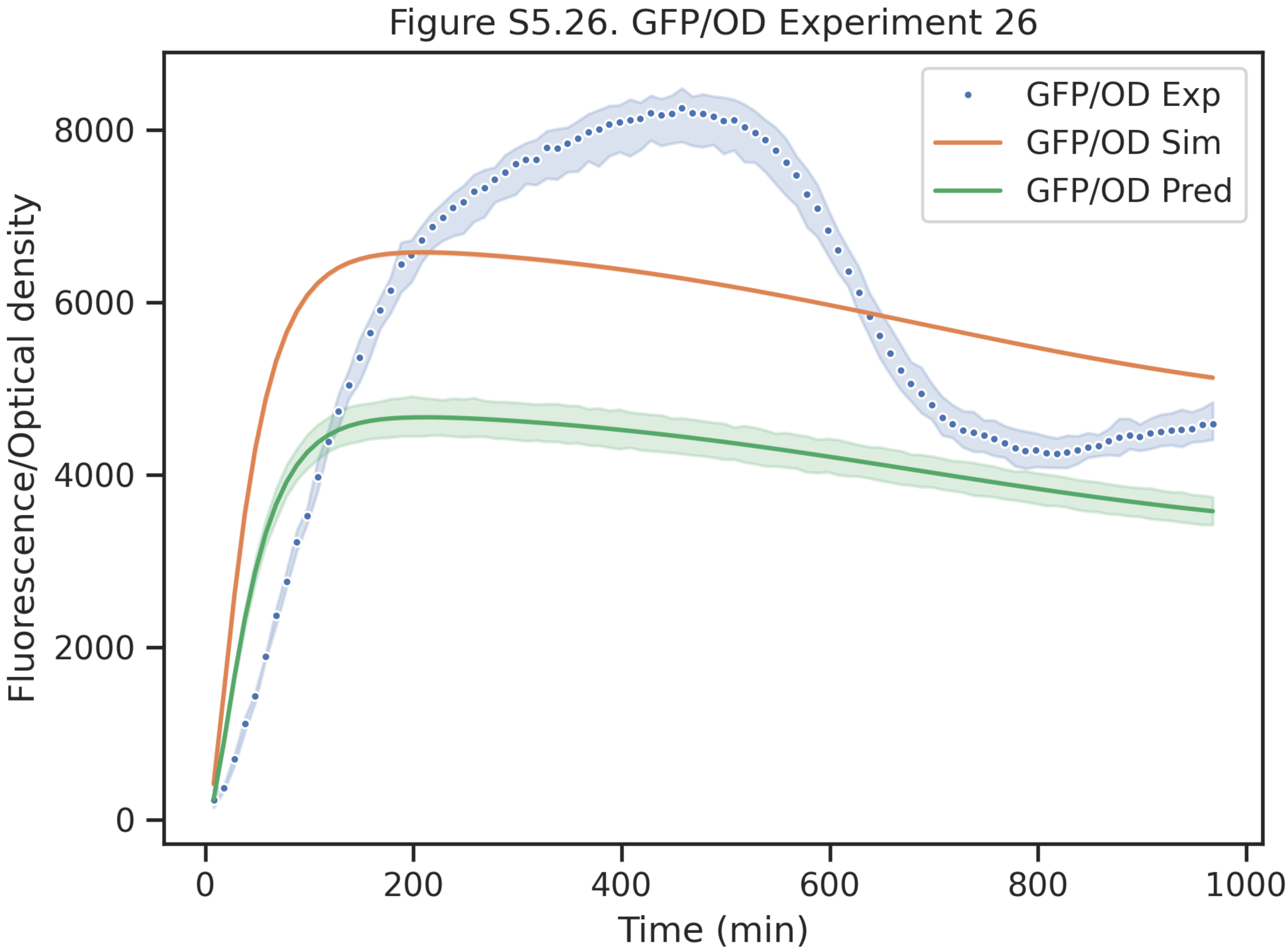

Figure S5.26. GFP/OD Experiment 26
GFP/OD Exp
GFP/OD Sim
GFP/OD Pred
Fluorescence/Optical density
Time (min)
0
2000
4000
6000
8000
0
200
400
600
800
1000

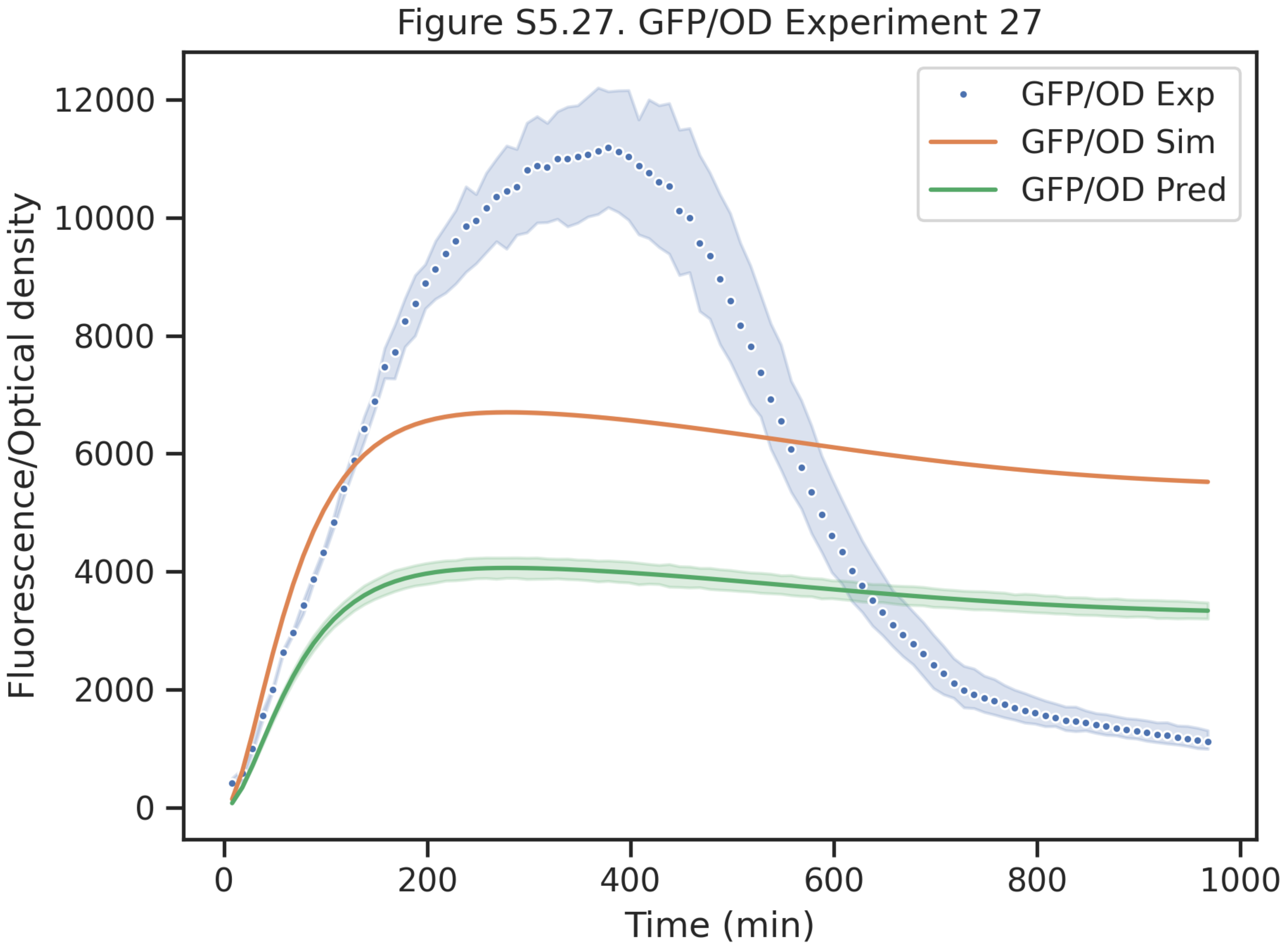

Figure S5.27. GFP/OD Experiment 27
GFP/OD Exp
GFP/OD Sim
GFP/OD Pred
Fluorescence/Optical density
Time (min)
0
2000
4000
6000
8000
10000
12000
0
200
400
600
800
1000

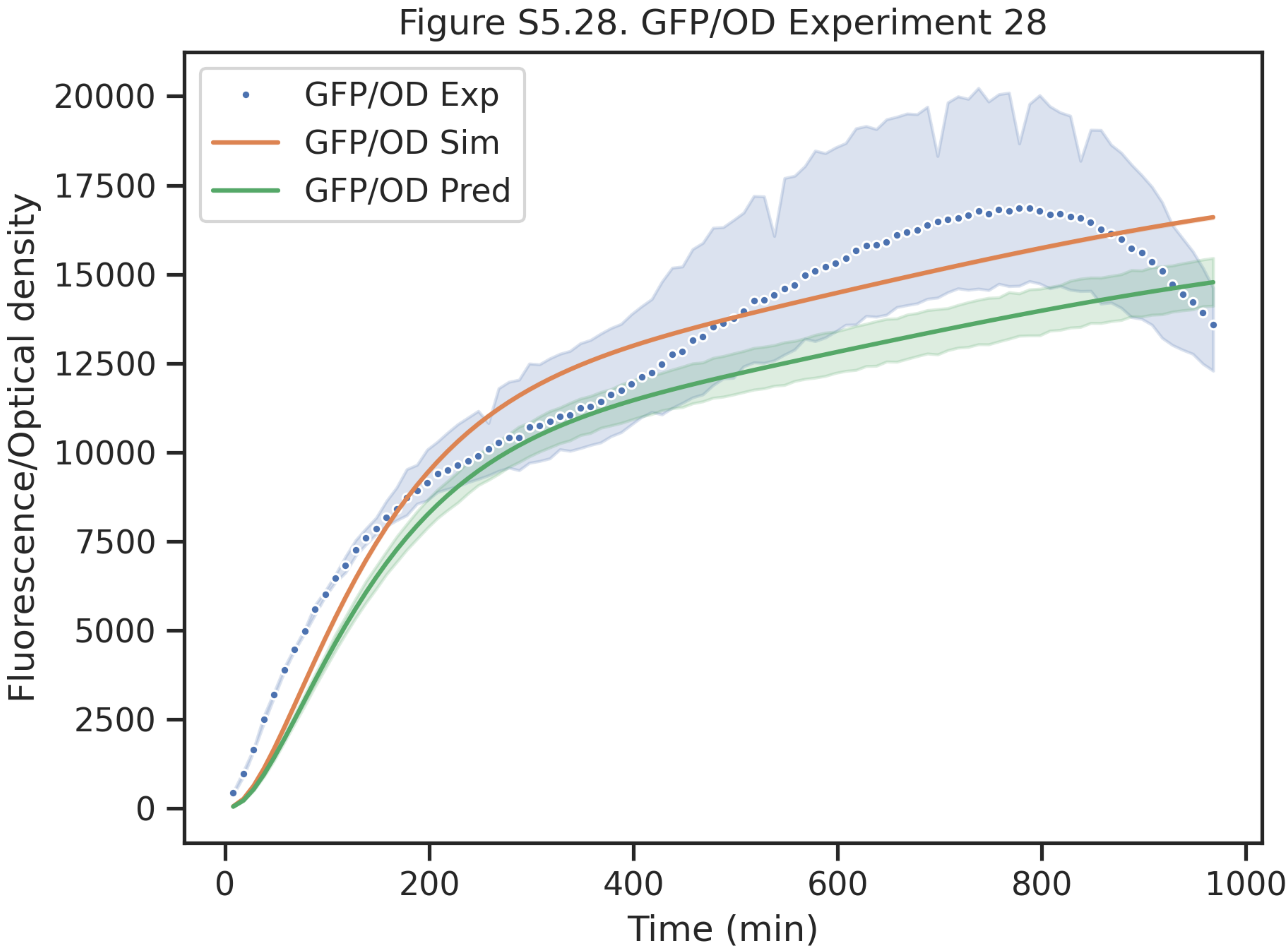
Figure S5.28. GFP/OD Experiment 28
GFP/OD Exp
GFP/OD Sim
GFP/OD Pred
Fluorescence/Optical density
Time (min)
0
2500
5000
7500
10000
12500
15000
17500
20000
0
200
400
600
800
1000

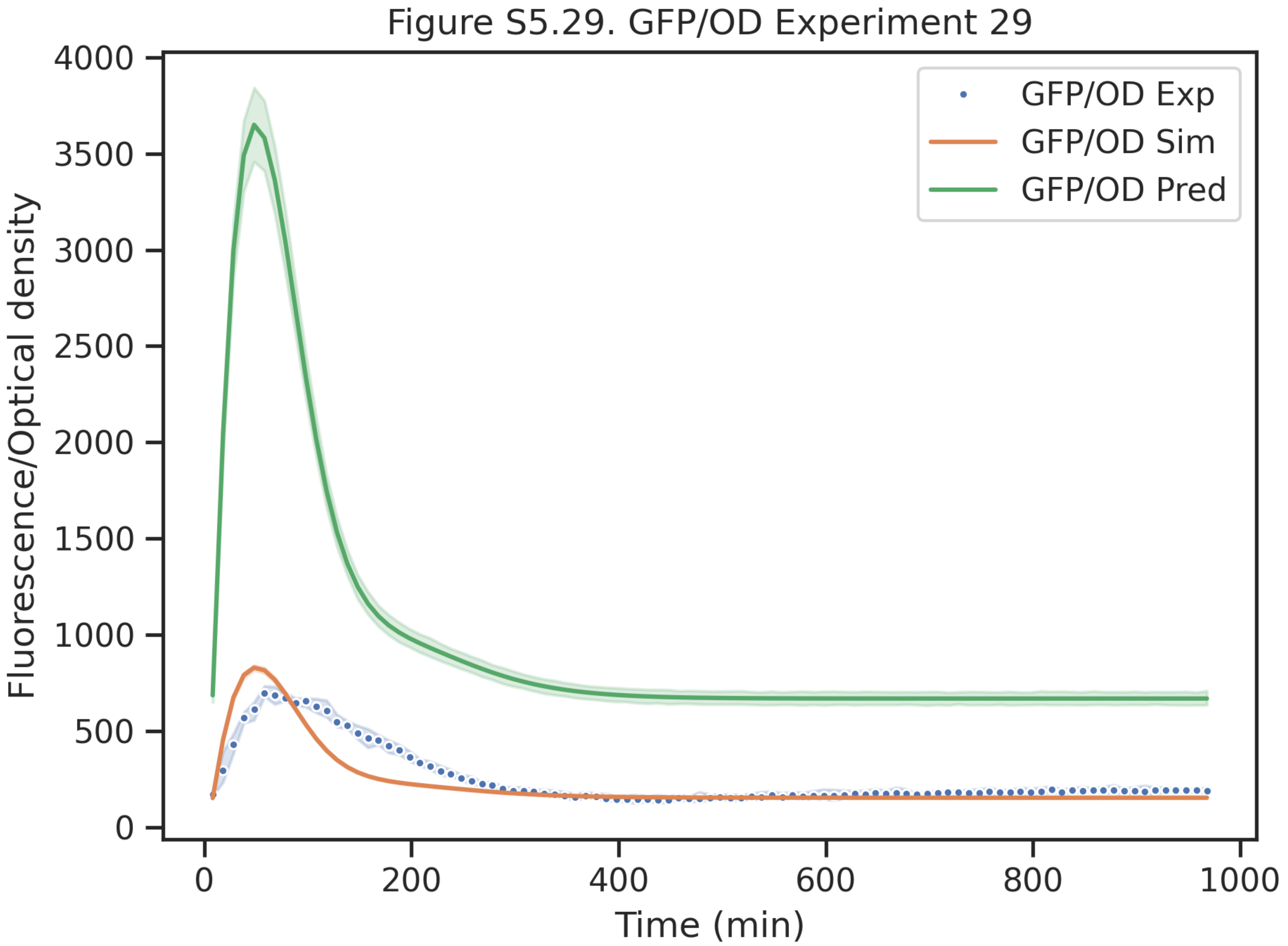
Figure S5.29. GFP/OD Experiment 29
GFP/OD Exp
GFP/OD Sim
GFP/OD Pred
Fluorescence/Optical density
Time (min)
0
500
1000
1500
2000
2500
3000
3500
4000
0
200
400
600
800
1000

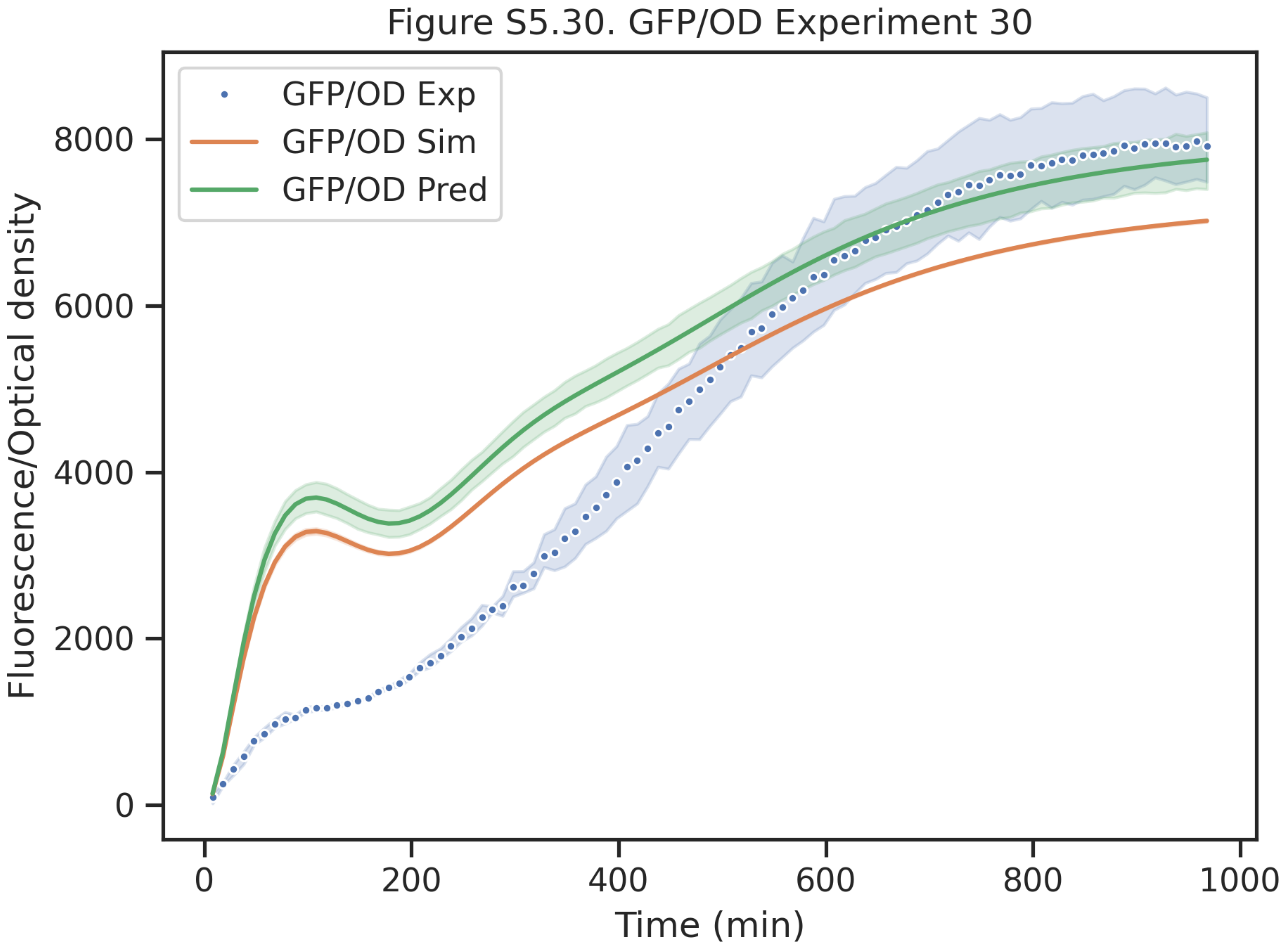

Figure S5.30. GFP/OD Experiment 30
GFP/OD Exp
GFP/OD Sim
GFP/OD Pred
Fluorescence/Optical density
Time (min)
0
2000
4000
6000
8000
0
200
400
600
800
1000

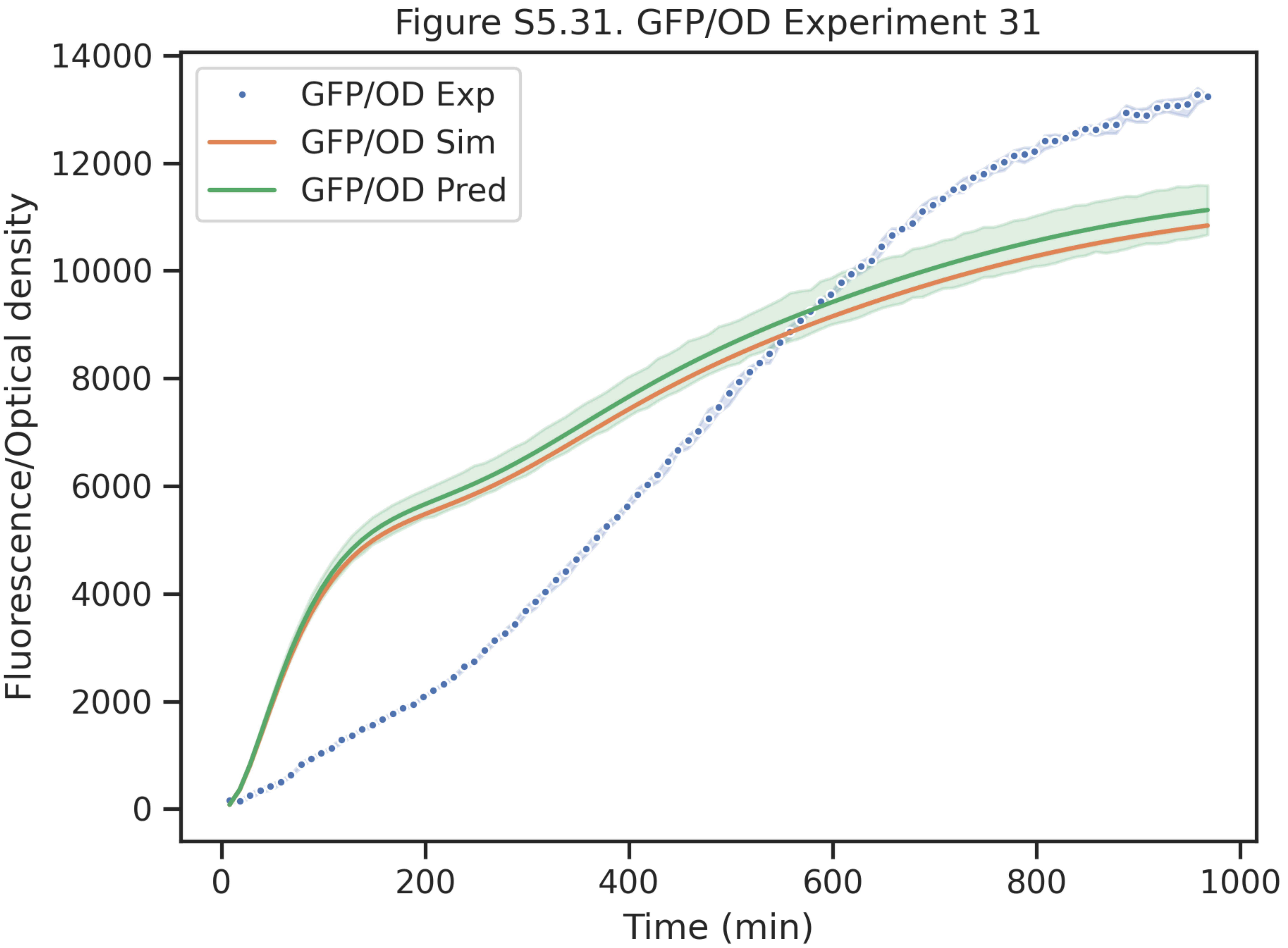
Figure S5.31. GFP/OD Experiment 31
GFP/OD Exp
GFP/OD Sim
GFP/OD Pred
Fluorescence/Optical density
Time (min)
0
2000
4000
6000
8000
10000
12000
14000
0
200
400
600
800
1000

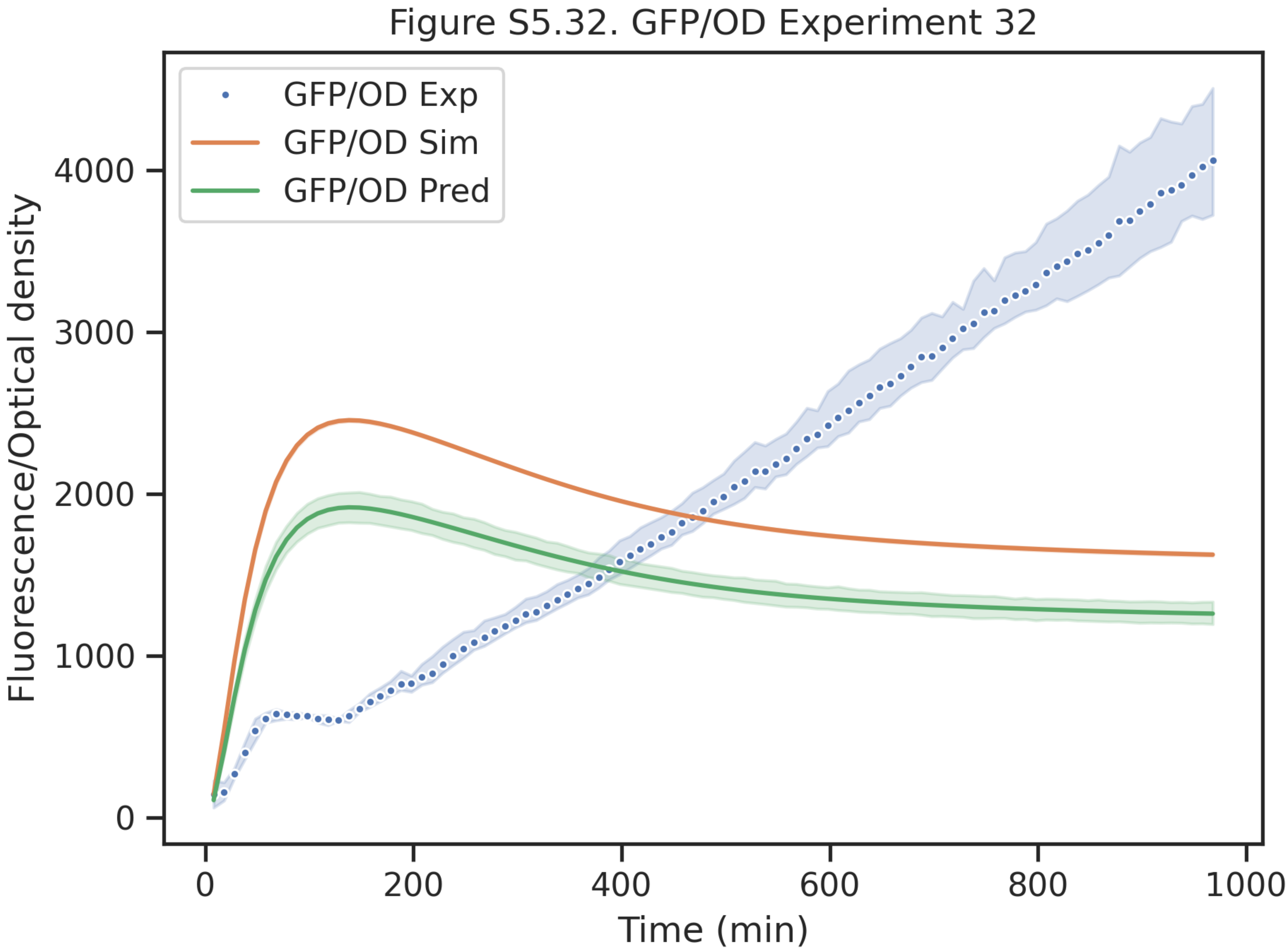
Figure S5.32. GFP/OD Experiment 32
GFP/OD Exp
GFP/OD Sim
GFP/OD Pred
4000
3000
2000
1000
0
0
200
400
600
800
1000
Time (min)
Fluorescence/Optical density

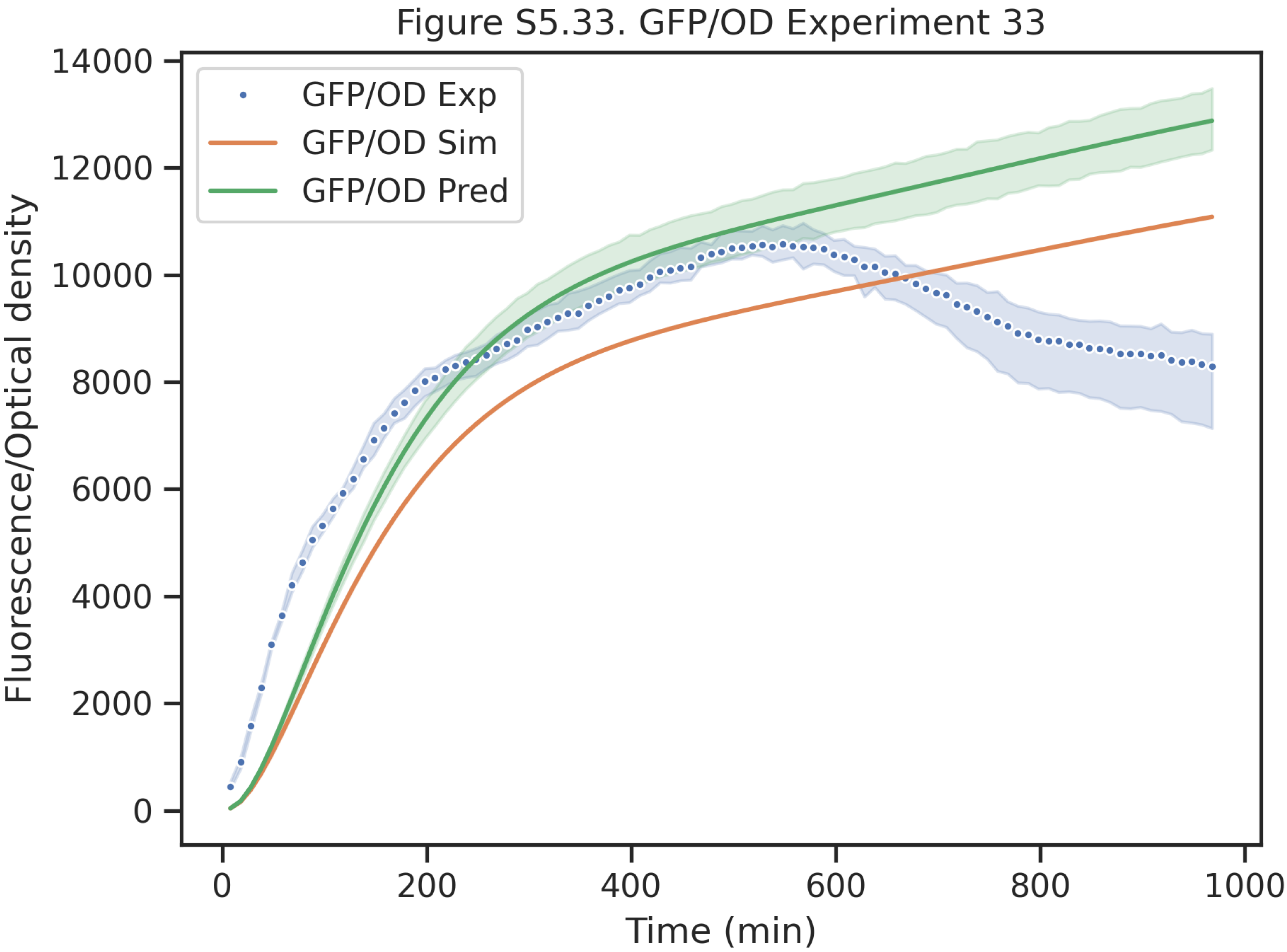

Figure S5.33. GFP/OD Experiment 33
GFP/OD Exp
GFP/OD Sim
GFP/OD Pred
14000
12000
10000
8000
6000
4000
2000
0
Fluorescence/Optical density
0
200
400
600
800
1000
Time (min)

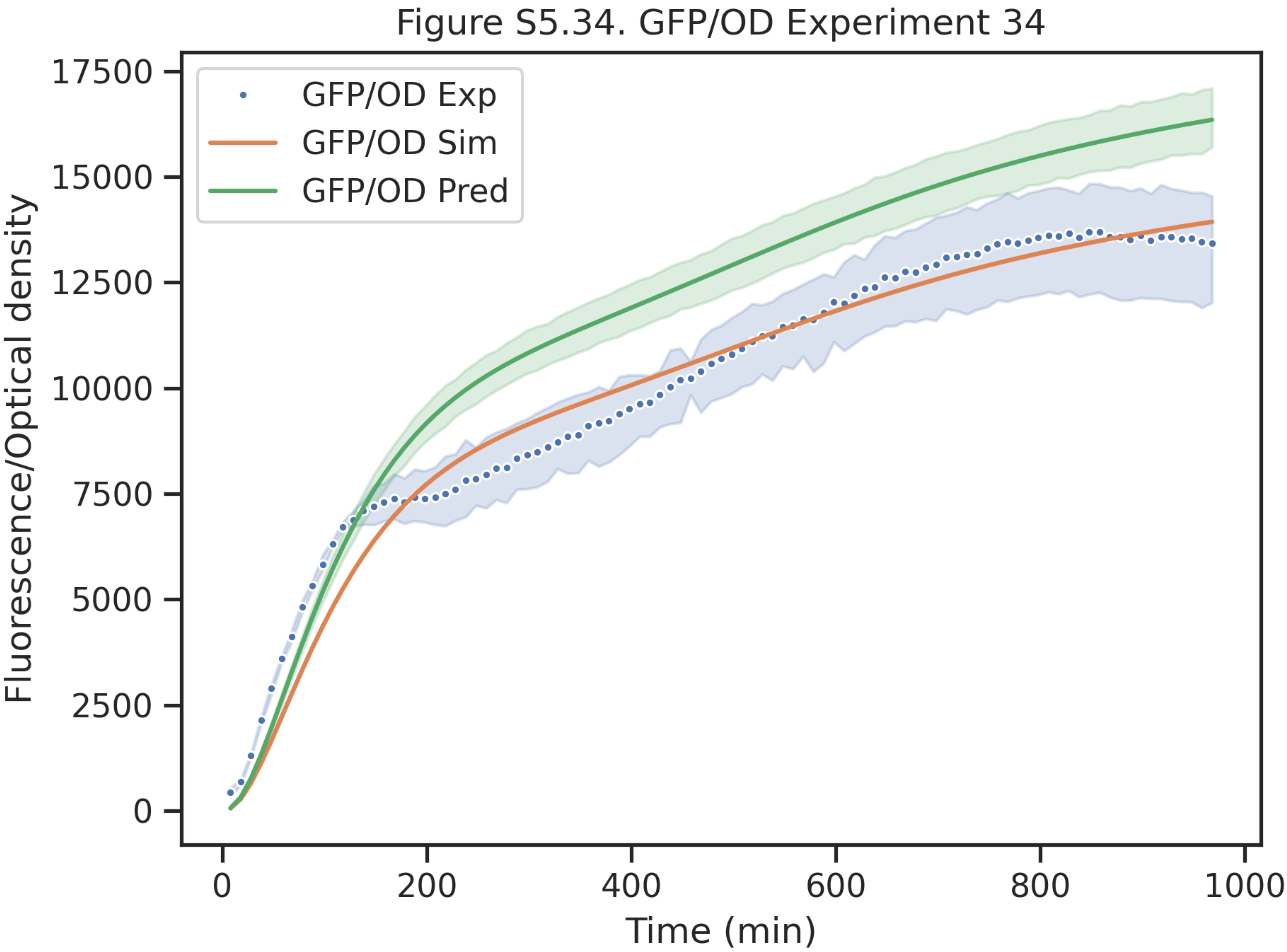
Figure S5.34. GFP/OD Experiment 34
GFP/OD Exp
GFP/OD Sim
GFP/OD Pred
Fluorescence/Optical density
Time (min)
0
2500
5000
7500
10000
12500
15000
17500
0
200
400
600
800
1000

Figure S5.35. GFP/OD Experiment 35

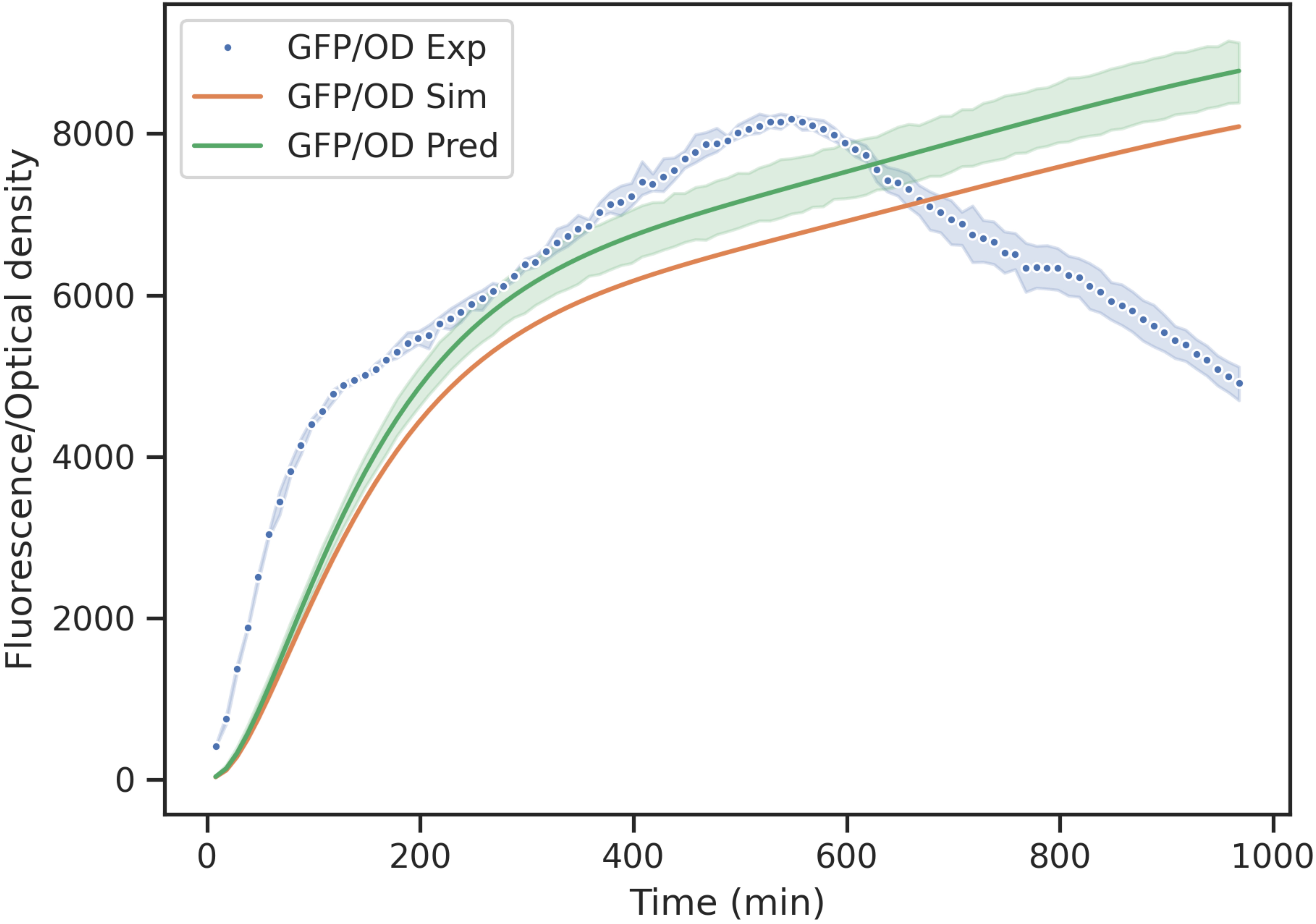

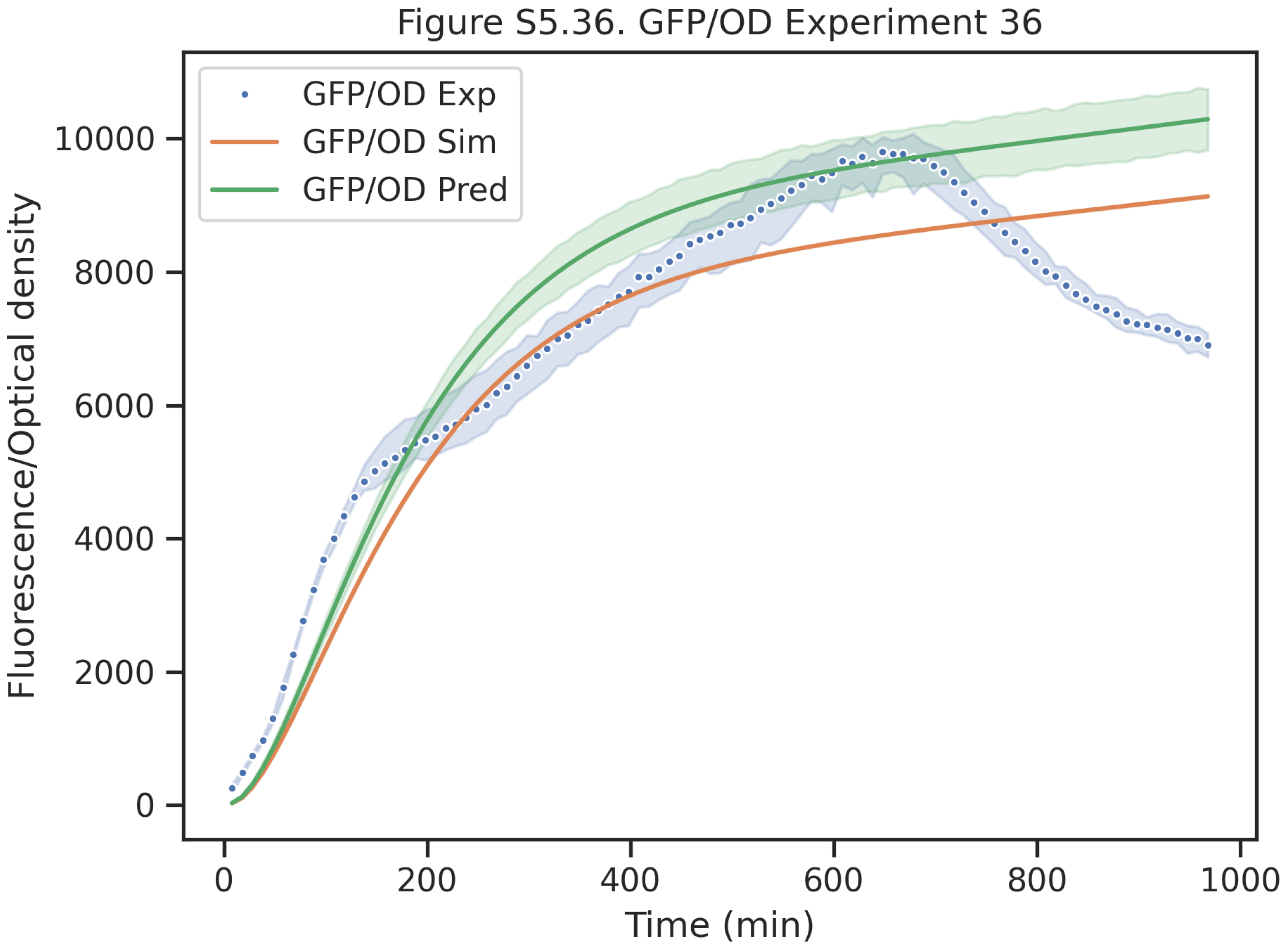

Figure S5.36. GFP/OD Experiment 36
GFP/OD Exp
GFP/OD Sim
GFP/OD Pred
Fluorescence/Optical density
0
2000
4000
6000
8000
10000
0
200
400
600
800
1000
Time (min)

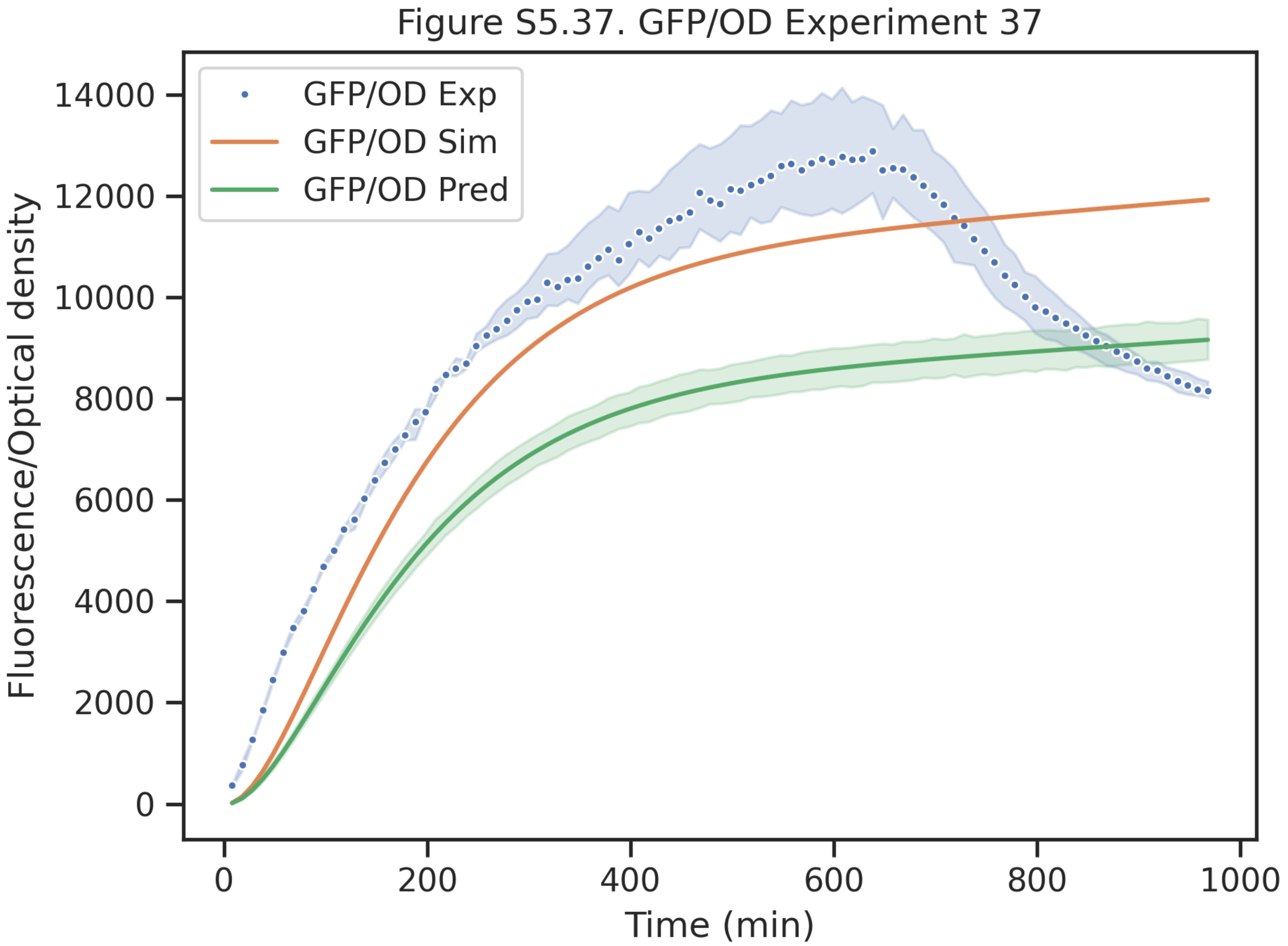

Figure S5.37. GFP/OD Experiment 37
GFP/OD Exp
GFP/OD Sim
GFP/OD Pred
Fluorescence/Optical density
Time (min)
0
2000
4000
6000
8000
10000
12000
14000
0
200
400
600
800
1000

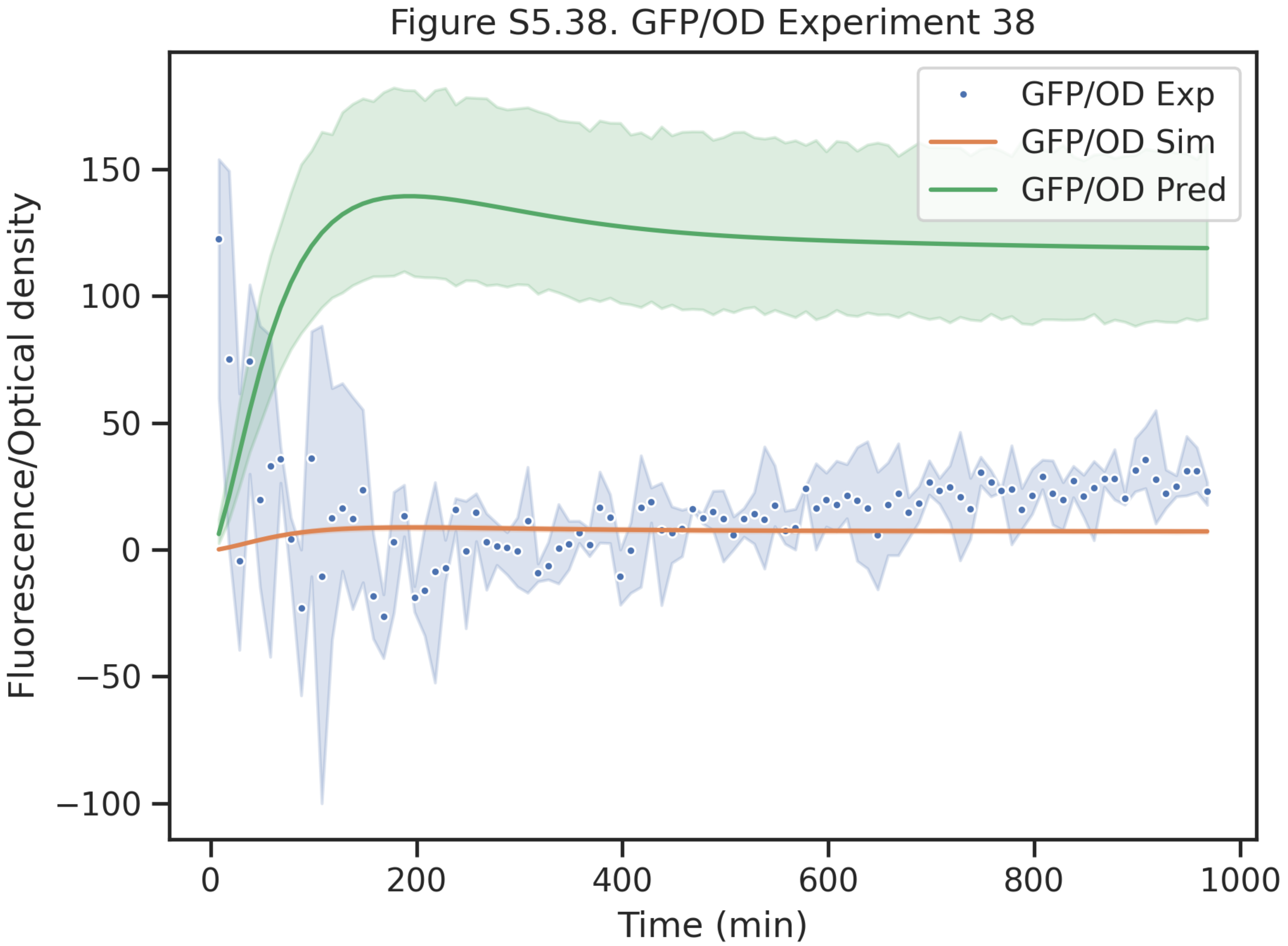
Figure S5.38. GFP/OD Experiment 38
GFP/OD Exp
GFP/OD Sim
GFP/OD Pred
Fluorescence/Optical density
Time (min)
150
100
50
0
−50
−100
0
200
400
600
800
1000

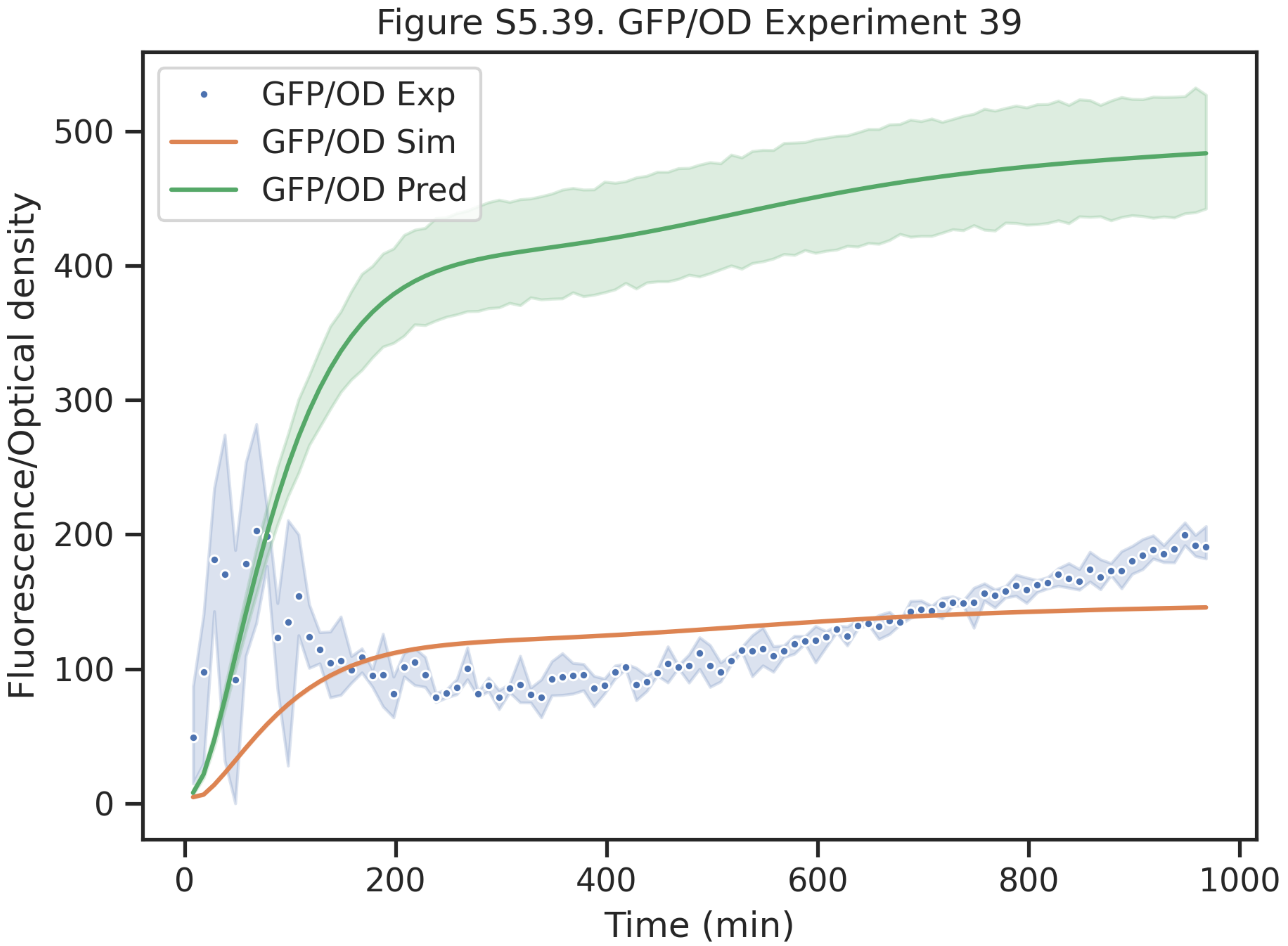

Figure S5.39. GFP/OD Experiment 39
GFP/OD Exp
GFP/OD Sim
GFP/OD Pred
Fluorescence/Optical density
Time (min)
0
100
200
300
400
500
0
200
400
600
800
1000

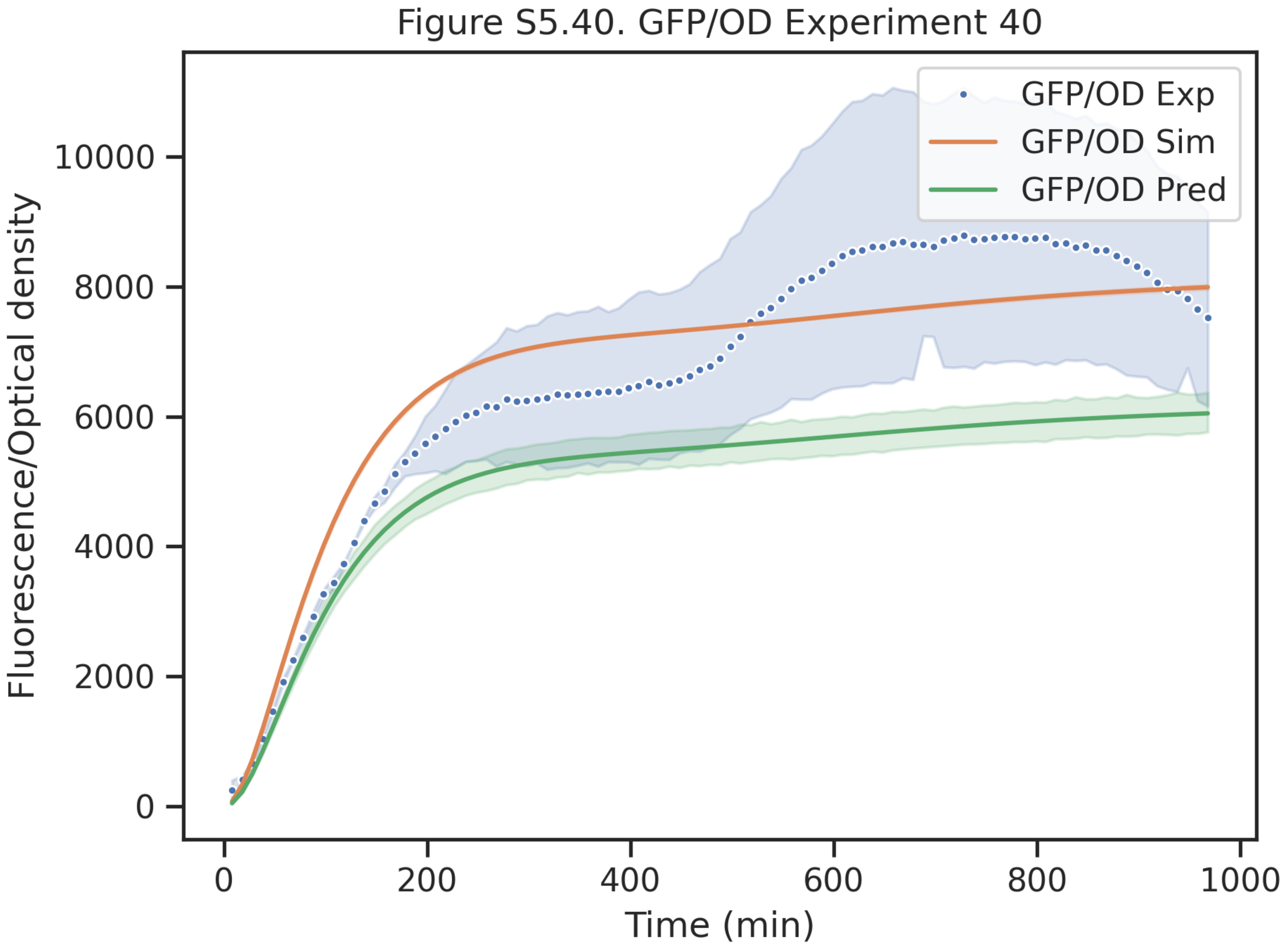

Figure S5.40. GFP/OD Experiment 40
GFP/OD Exp
GFP/OD Sim
GFP/OD Pred
Fluorescence/Optical density
0
2000
4000
6000
8000
10000
0
200
400
600
800
1000
Time (min)

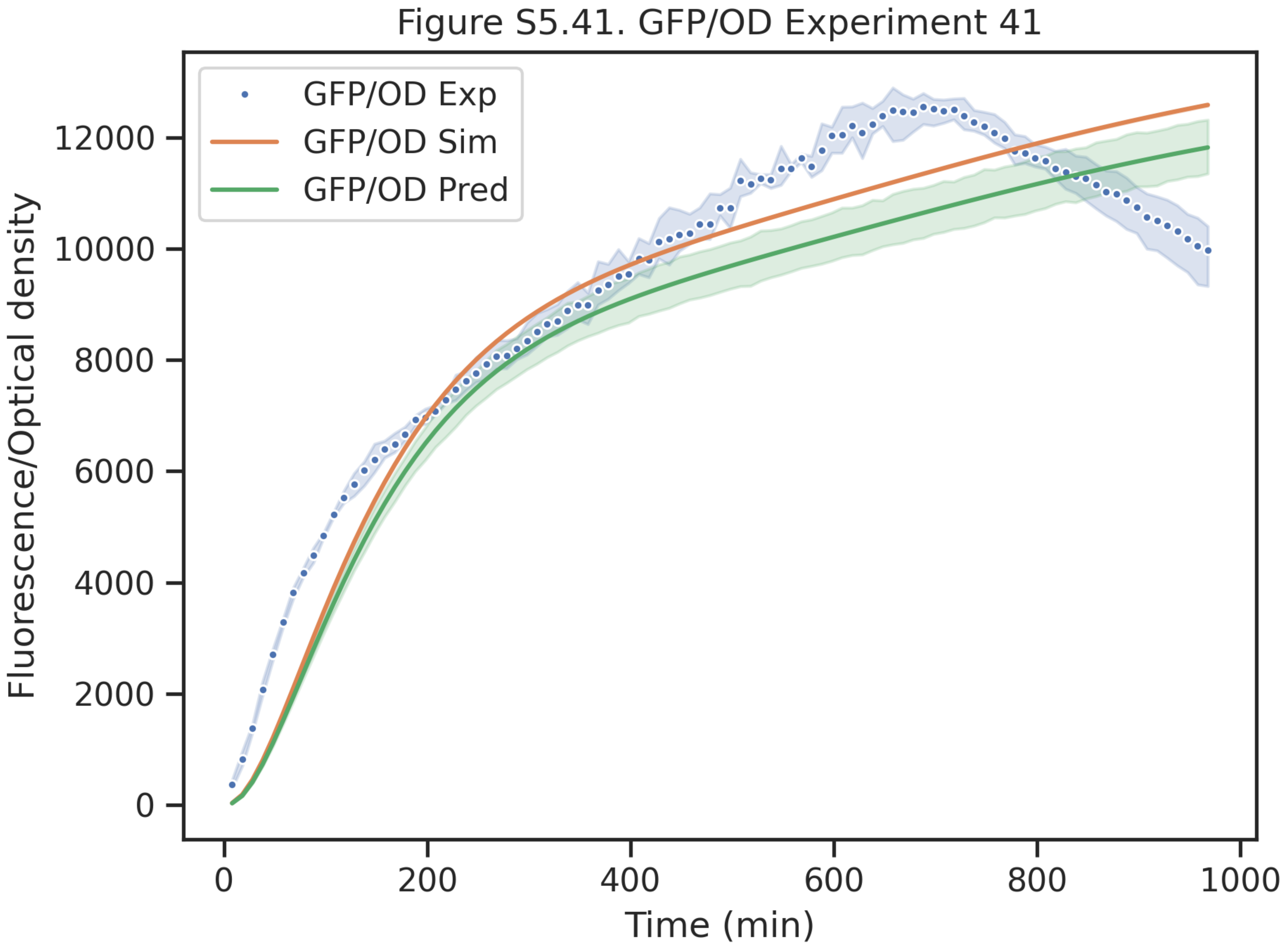

Figure S5.41. GFP/OD Experiment 41
GFP/OD Exp
GFP/OD Sim
GFP/OD Pred
Fluorescence/Optical density
Time (min)
0
2000
4000
6000
8000
10000
12000
0
200
400
600
800
1000

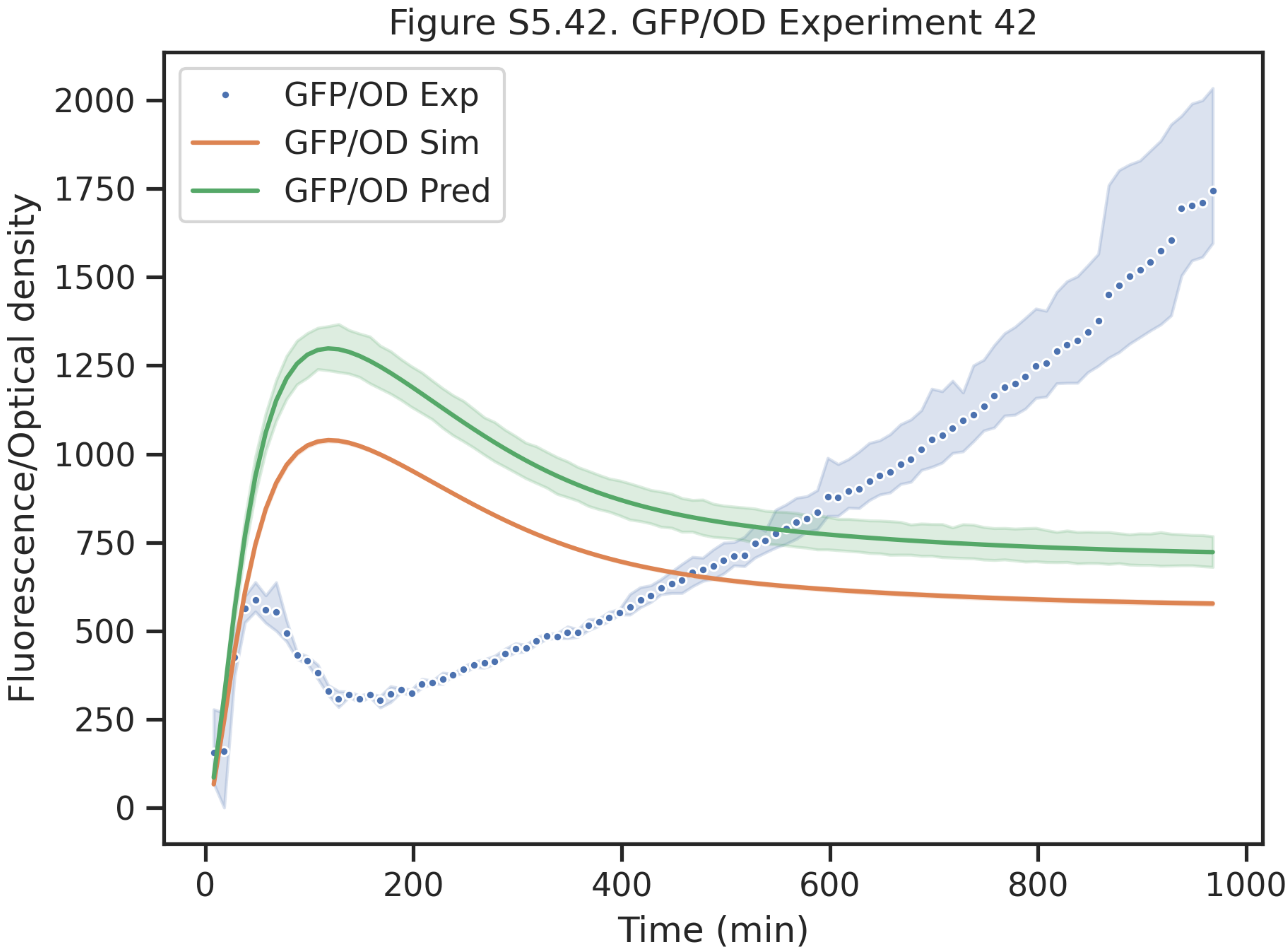

Figure S5.42. GFP/OD Experiment 42
GFP/OD Exp
GFP/OD Sim
GFP/OD Pred
Fluorescence/Optical density
0
250
500
750
1000
1250
1500
1750
2000
Time (min)
0
200
400
600
800
1000

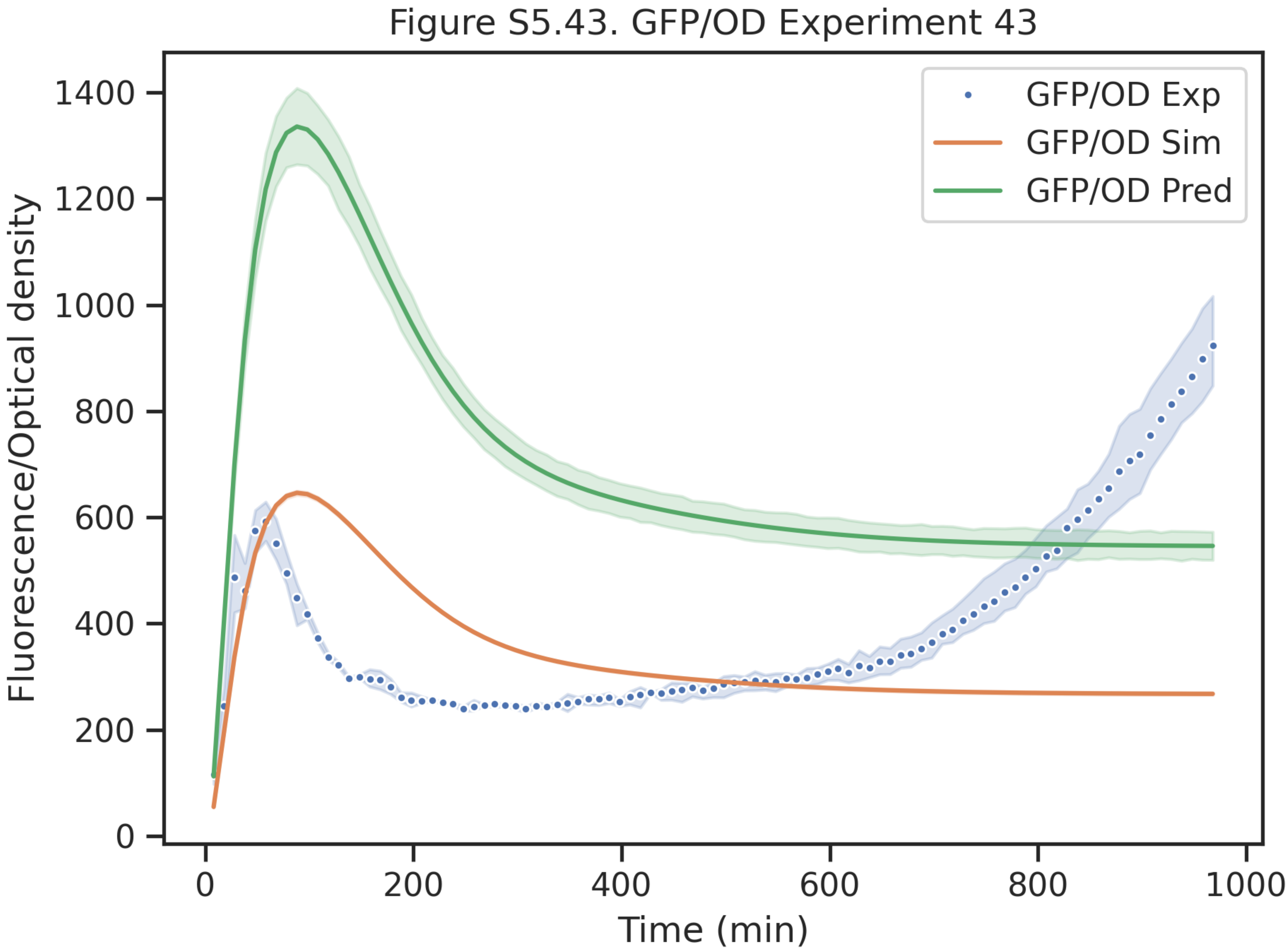
Figure S5.43. GFP/OD Experiment 43
Fluorescence/Optical density
0
200
400
600
800
1000
1200
1400
Time (min)
0
200
400
600
800
1000
GFP/OD Exp
GFP/OD Sim
GFP/OD Pred

Figure S5.44. GFP/OD Experiment 44

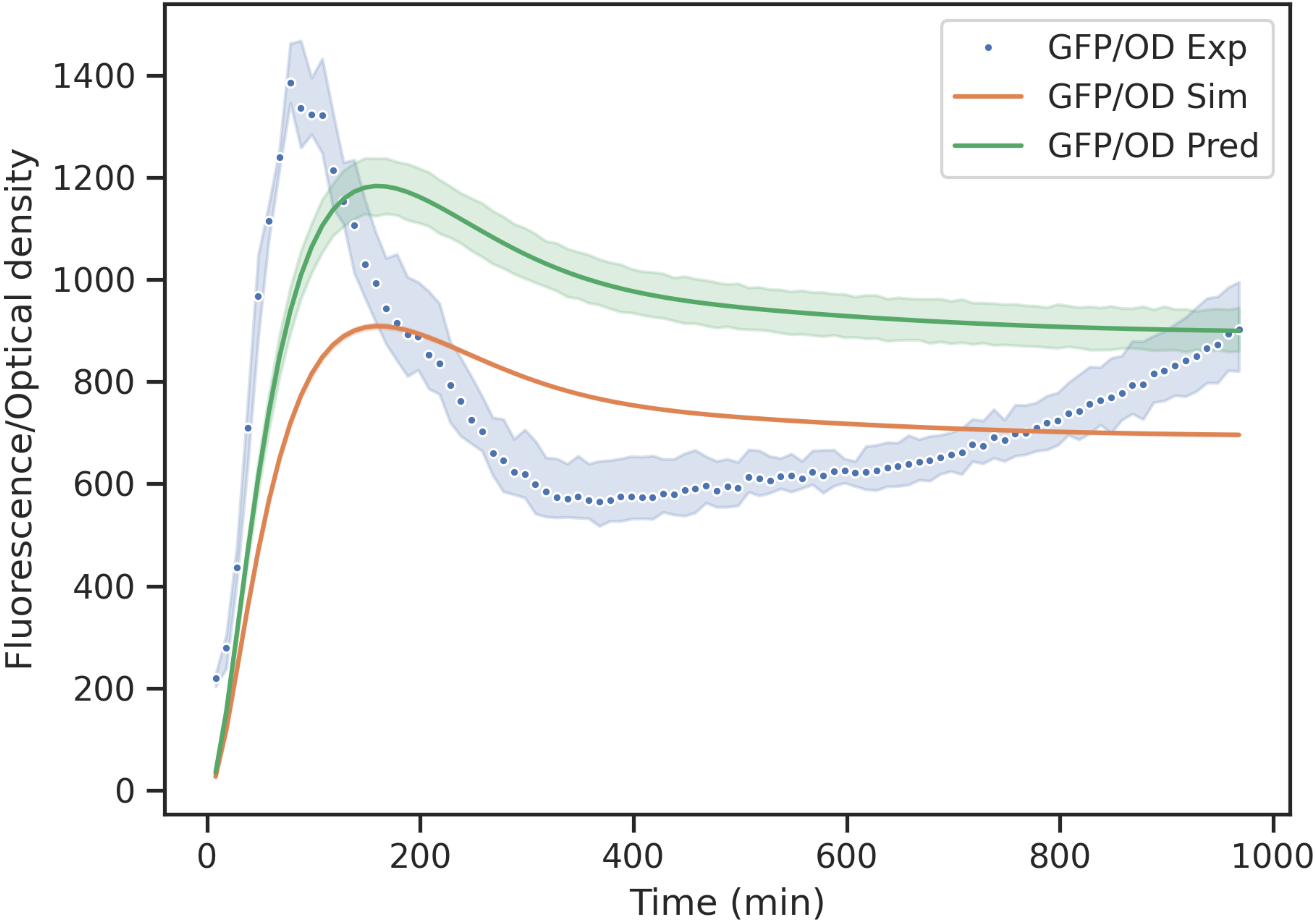

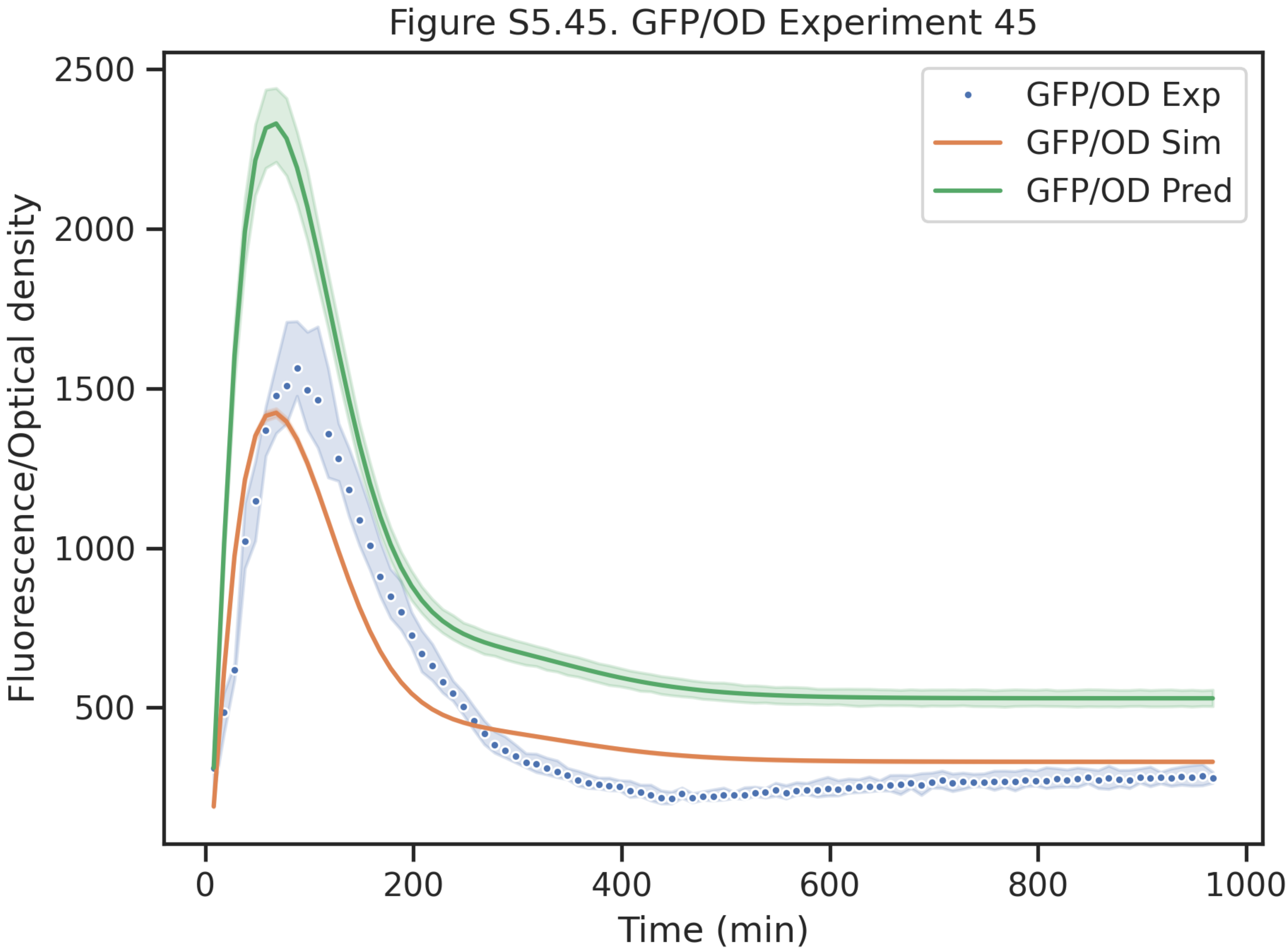

Figure S5.45. GFP/OD Experiment 45
GFP/OD Exp
GFP/OD Sim
GFP/OD Pred
Fluorescence/Optical density
2500
2000
1500
1000
500
0
200
400
600
800
1000
Time (min)

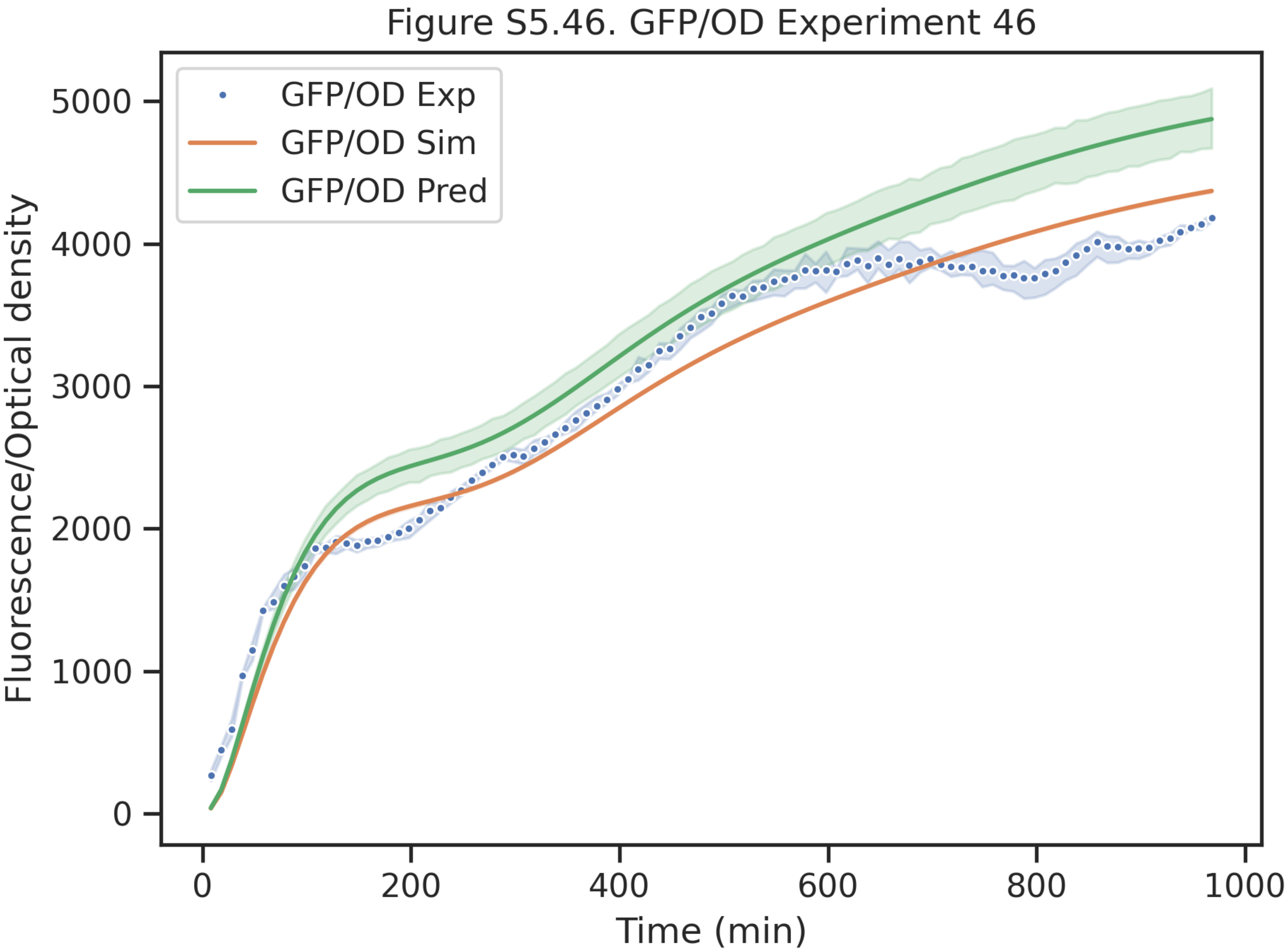
Figure S5.46. GFP/OD Experiment 46
GFP/OD Exp
GFP/OD Sim
GFP/OD Pred
Fluorescence/Optical density
Time (min)
0
1000
2000
3000
4000
5000
0
200
400
600
800
1000

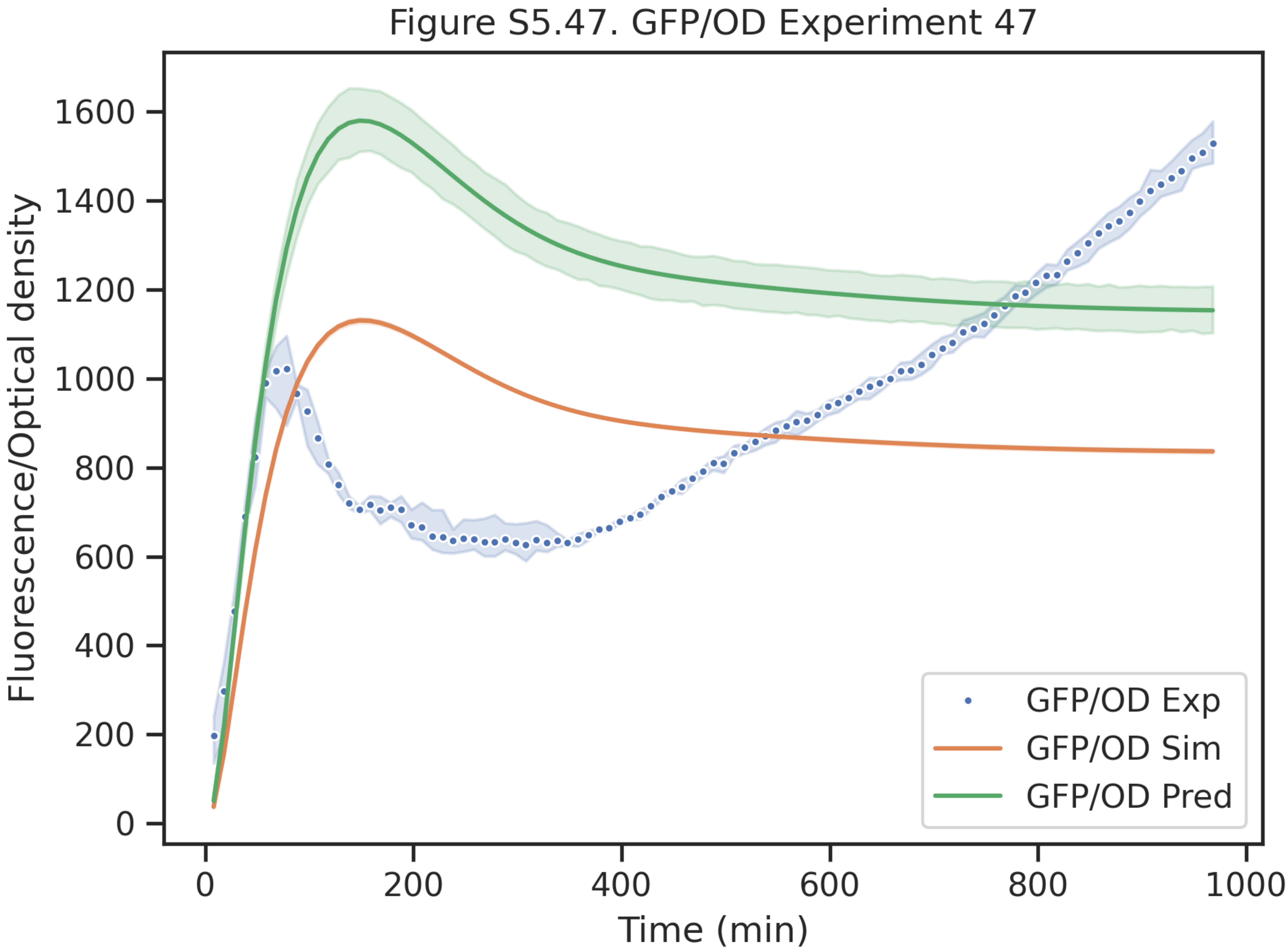
Figure S5.47. GFP/OD Experiment 47
Fluorescence/Optical density
Time (min)
0
200
400
600
800
1000
1200
1400
1600
0
200
400
600
800
1000
GFP/OD Exp
GFP/OD Sim
GFP/OD Pred

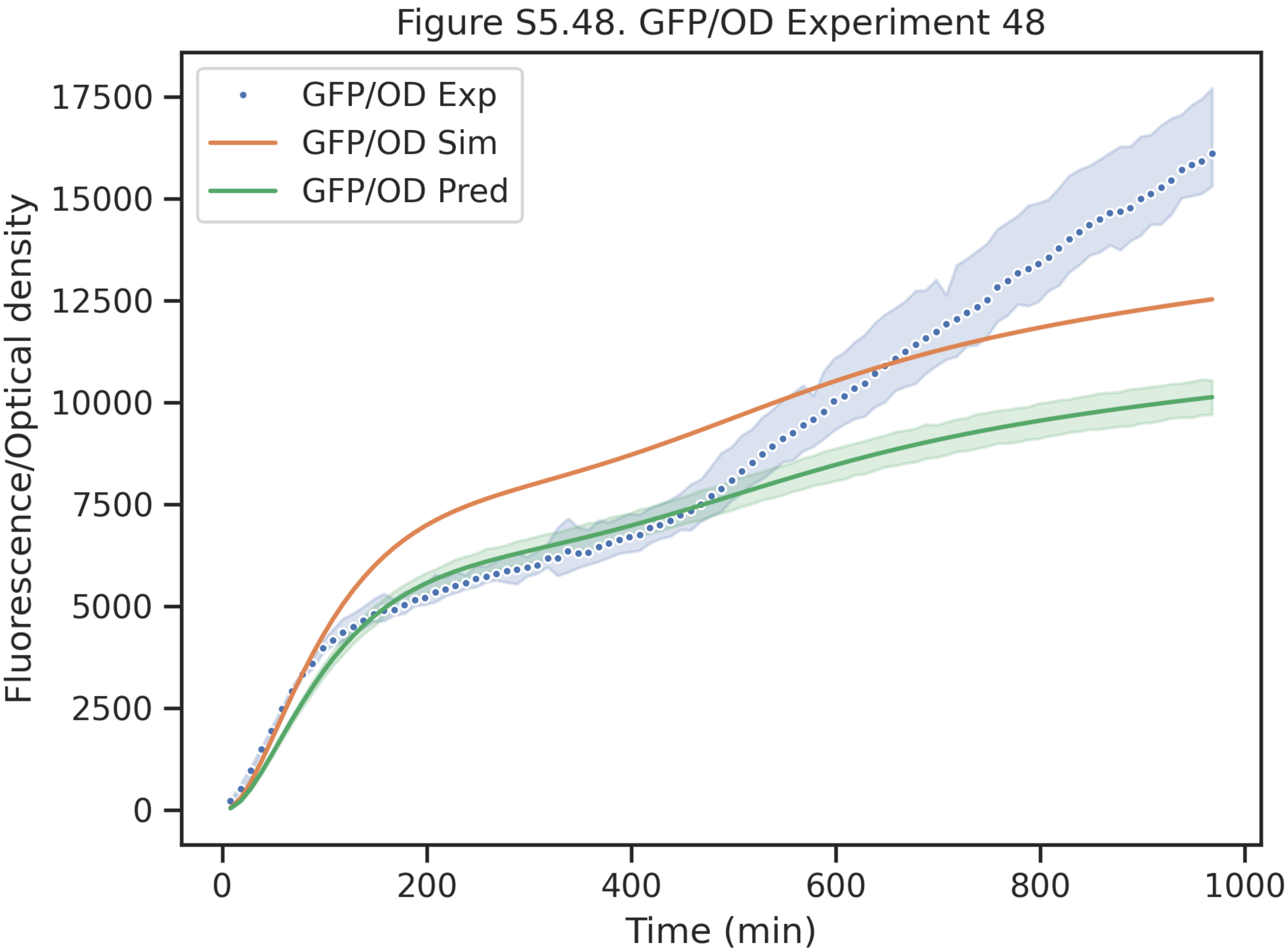

Figure S5.48. GFP/OD Experiment 48
GFP/OD Exp
GFP/OD Sim
GFP/OD Pred
Fluorescence/Optical density
Time (min)
0
2500
5000
7500
10000
12500
15000
17500
0
200
400
600
800
1000

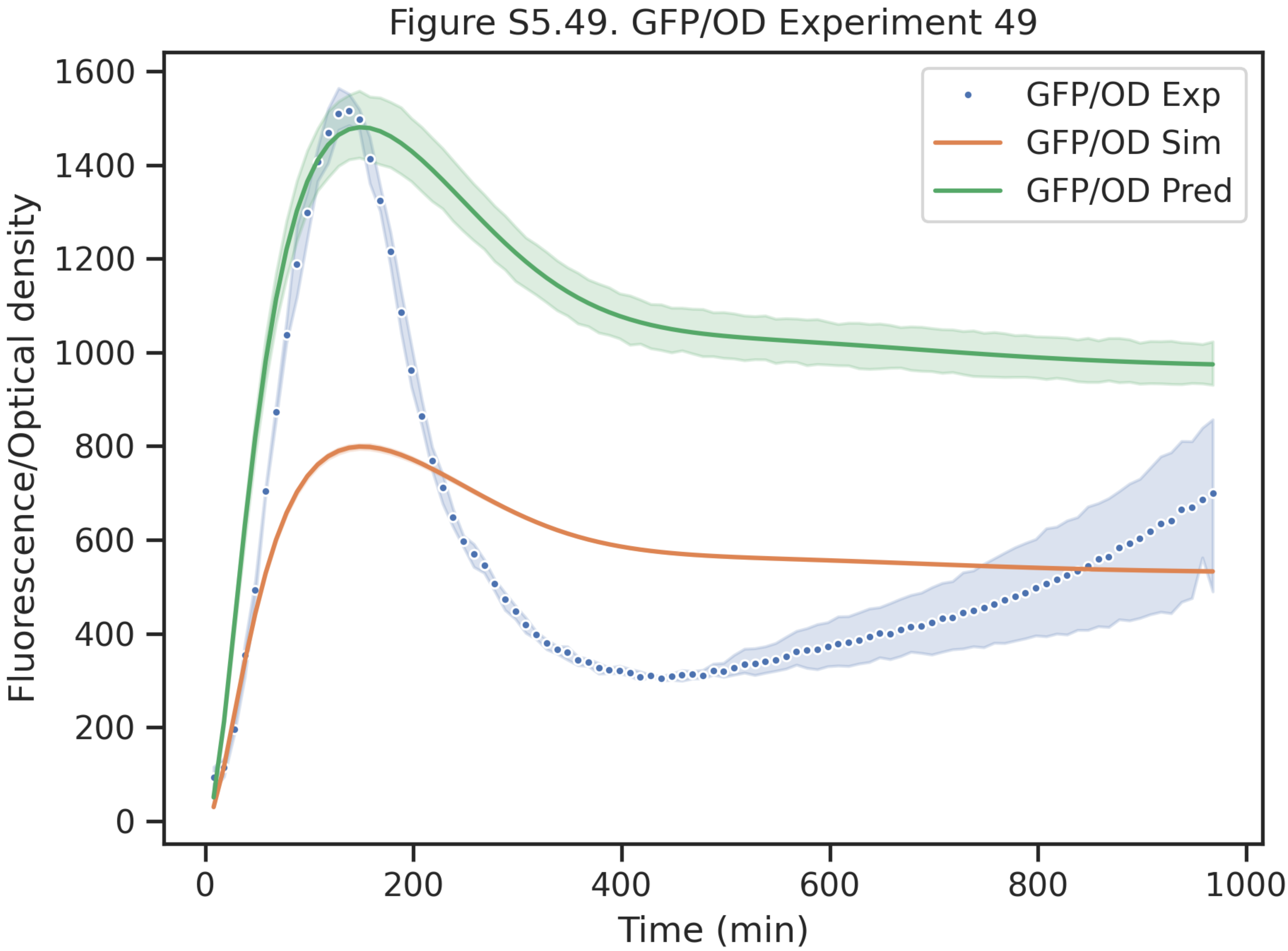

Figure S5.49. GFP/OD Experiment 49
GFP/OD Exp
GFP/OD Sim
GFP/OD Pred
Fluorescence/Optical density
Time (min)
0
200
400
600
800
1000
1200
1400
1600

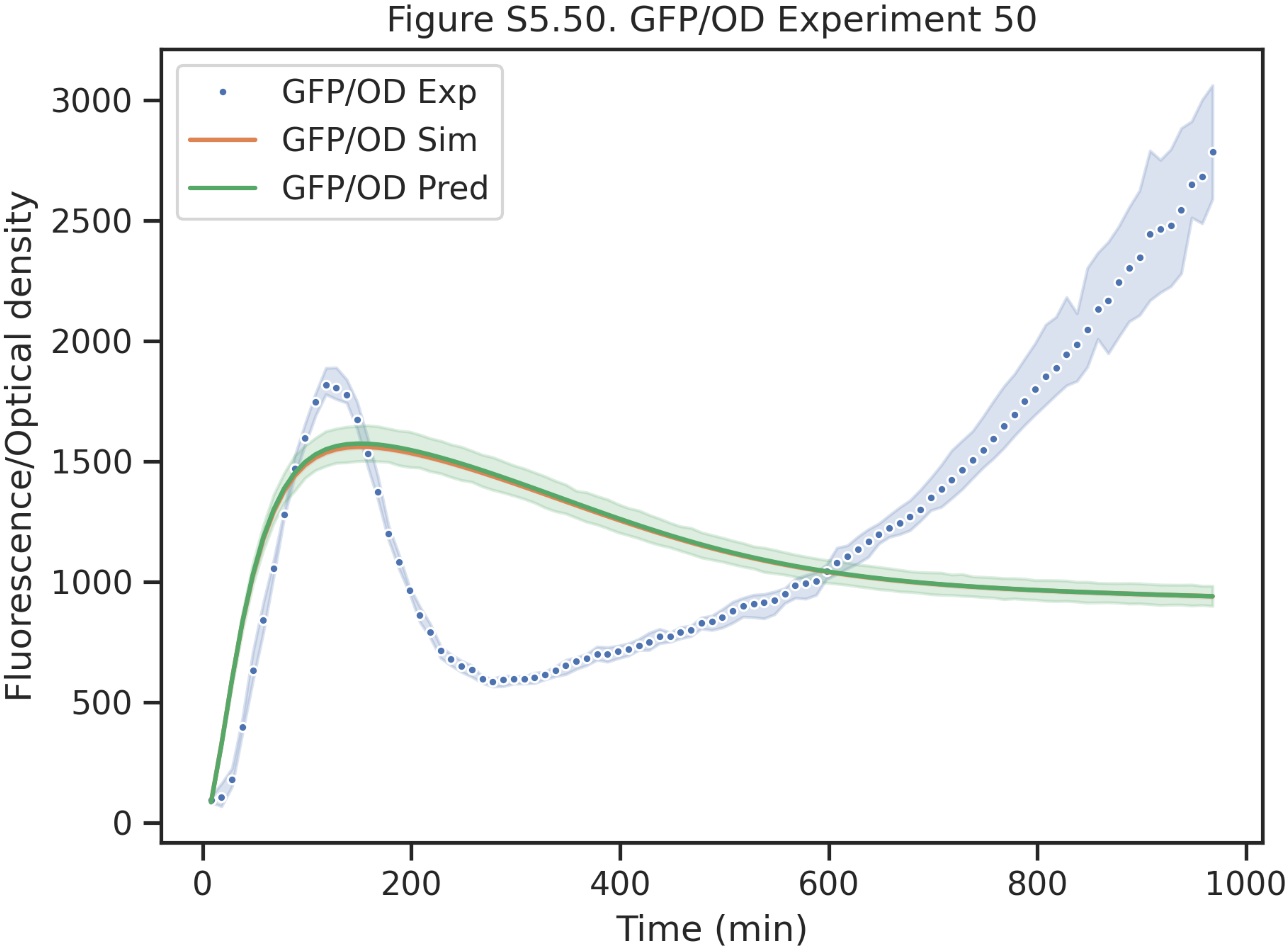

Figure S5.50. GFP/OD Experiment 50
GFP/OD Exp
GFP/OD Sim
GFP/OD Pred
Fluorescence/Optical density
Time (min)
0
500
1000
1500
2000
2500
3000
0
200
400
600
800
1000

Figure S5.51. GFP/OD Experiment 51

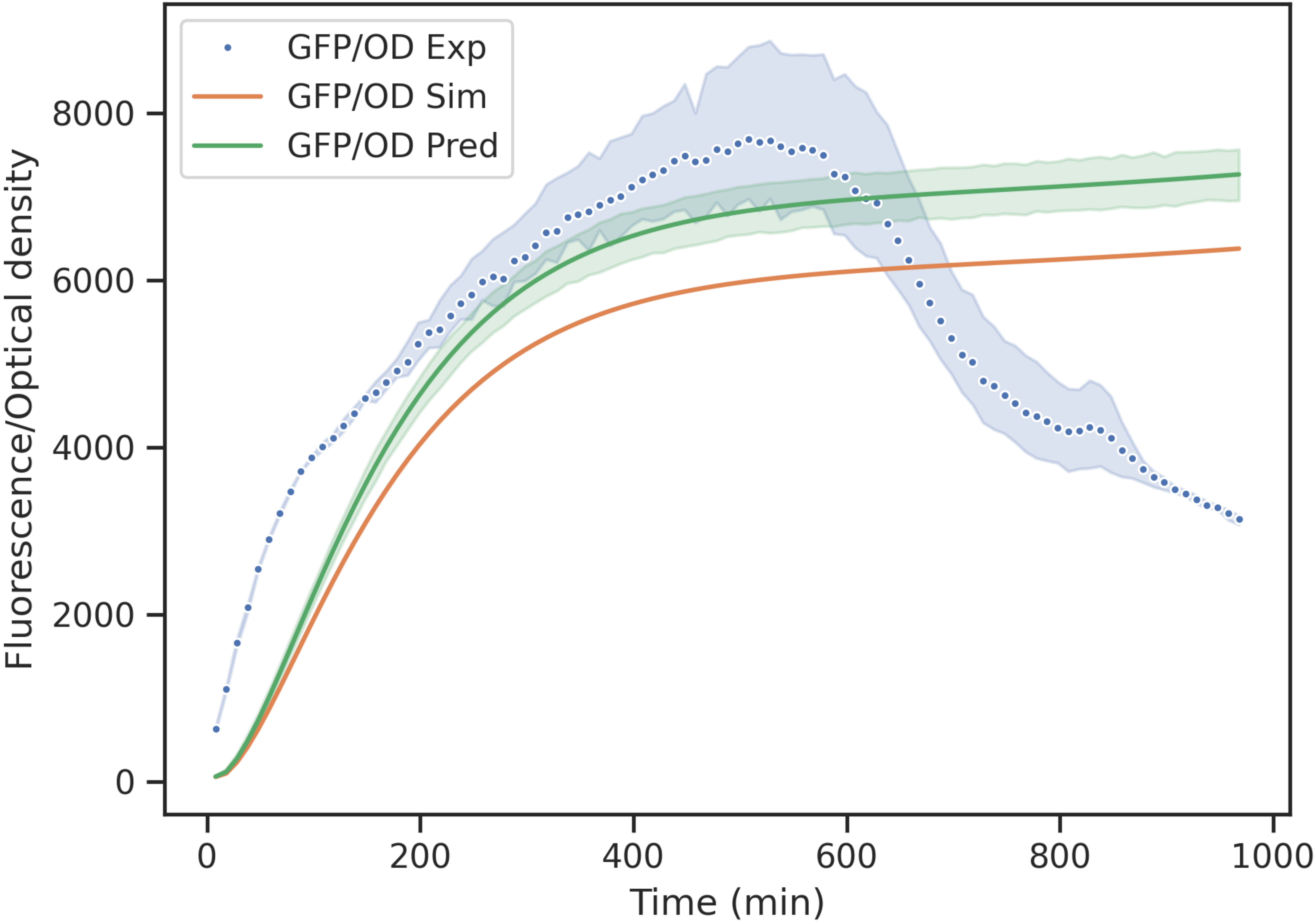

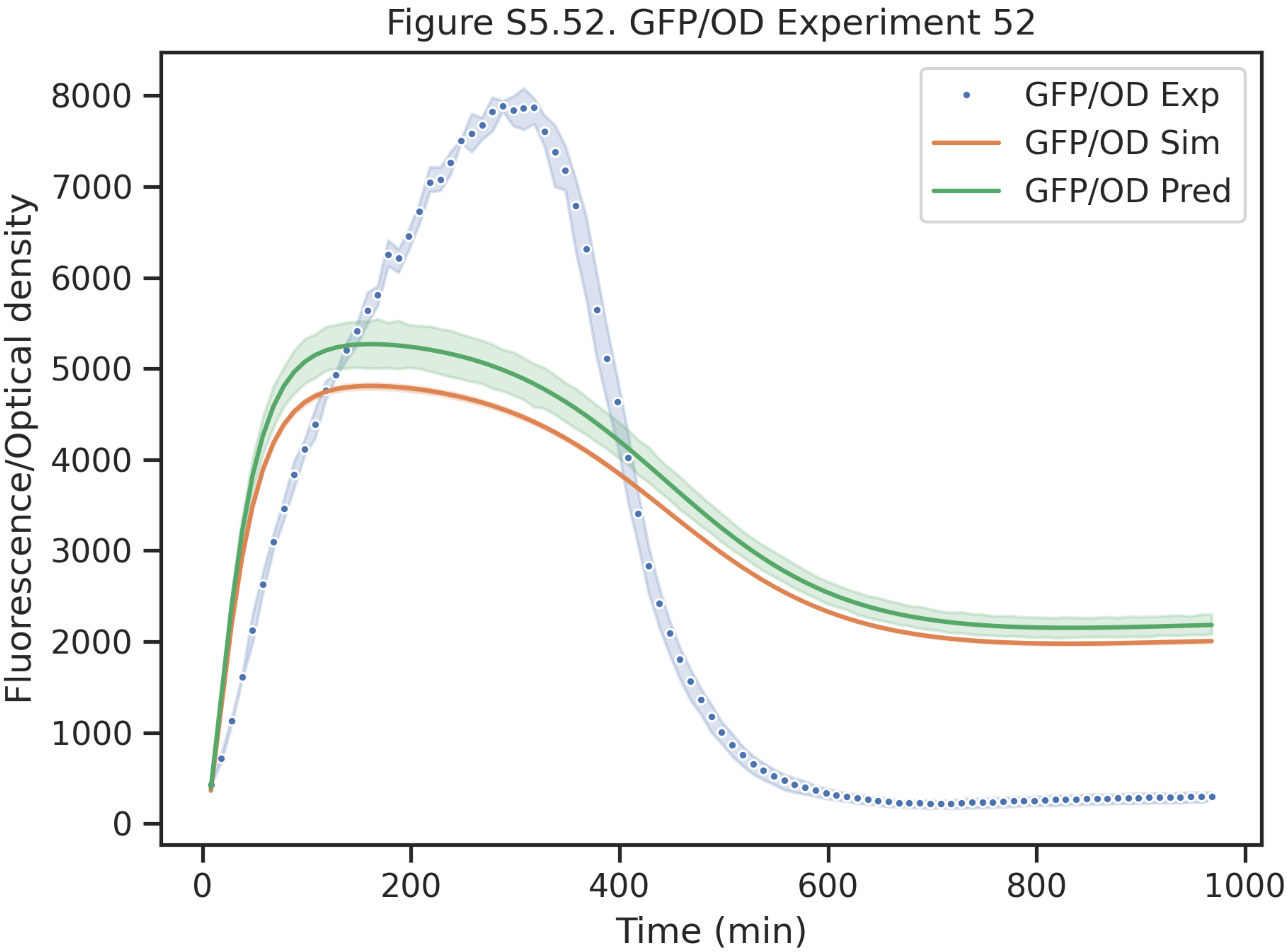

Figure S5.52. GFP/OD Experiment 52
GFP/OD Exp
GFP/OD Sim
GFP/OD Pred
Fluorescence/Optical density
Time (min)
0
1000
2000
3000
4000
5000
6000
7000
8000
0
200
400
600
800
1000

Figure S5.53. GFP/OD Experiment 53

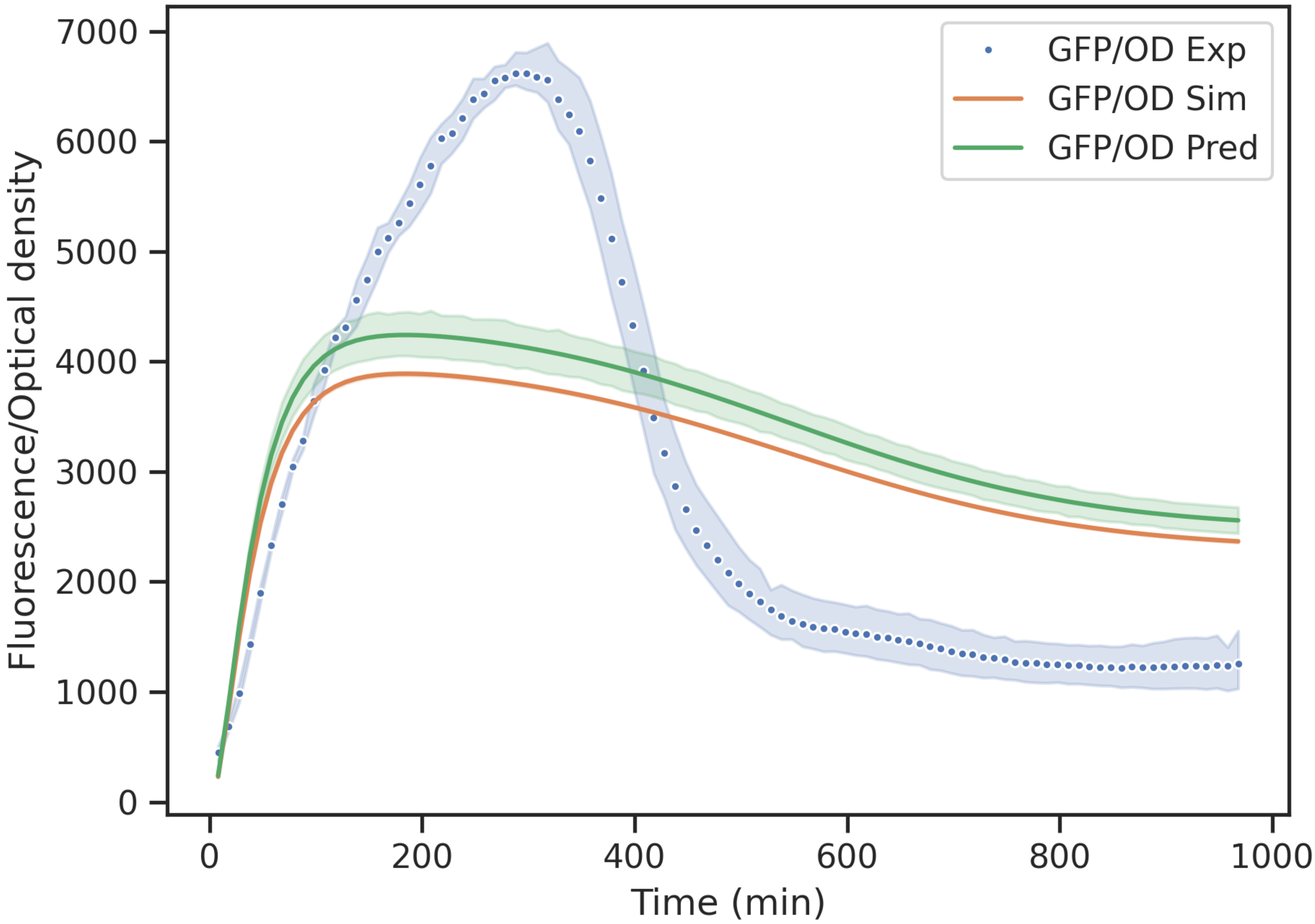

Figure S5.54. GFP/OD Experiment 54

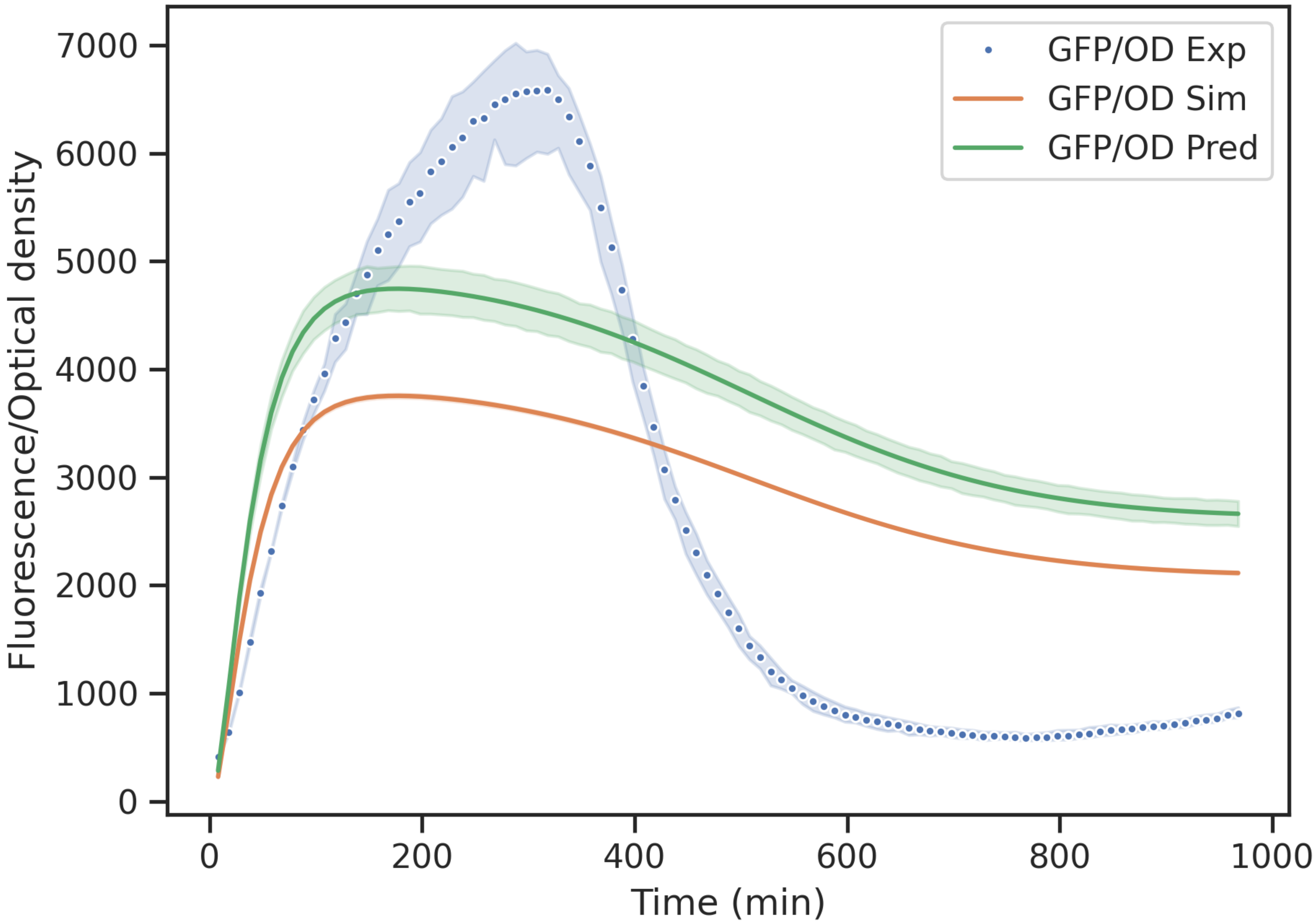

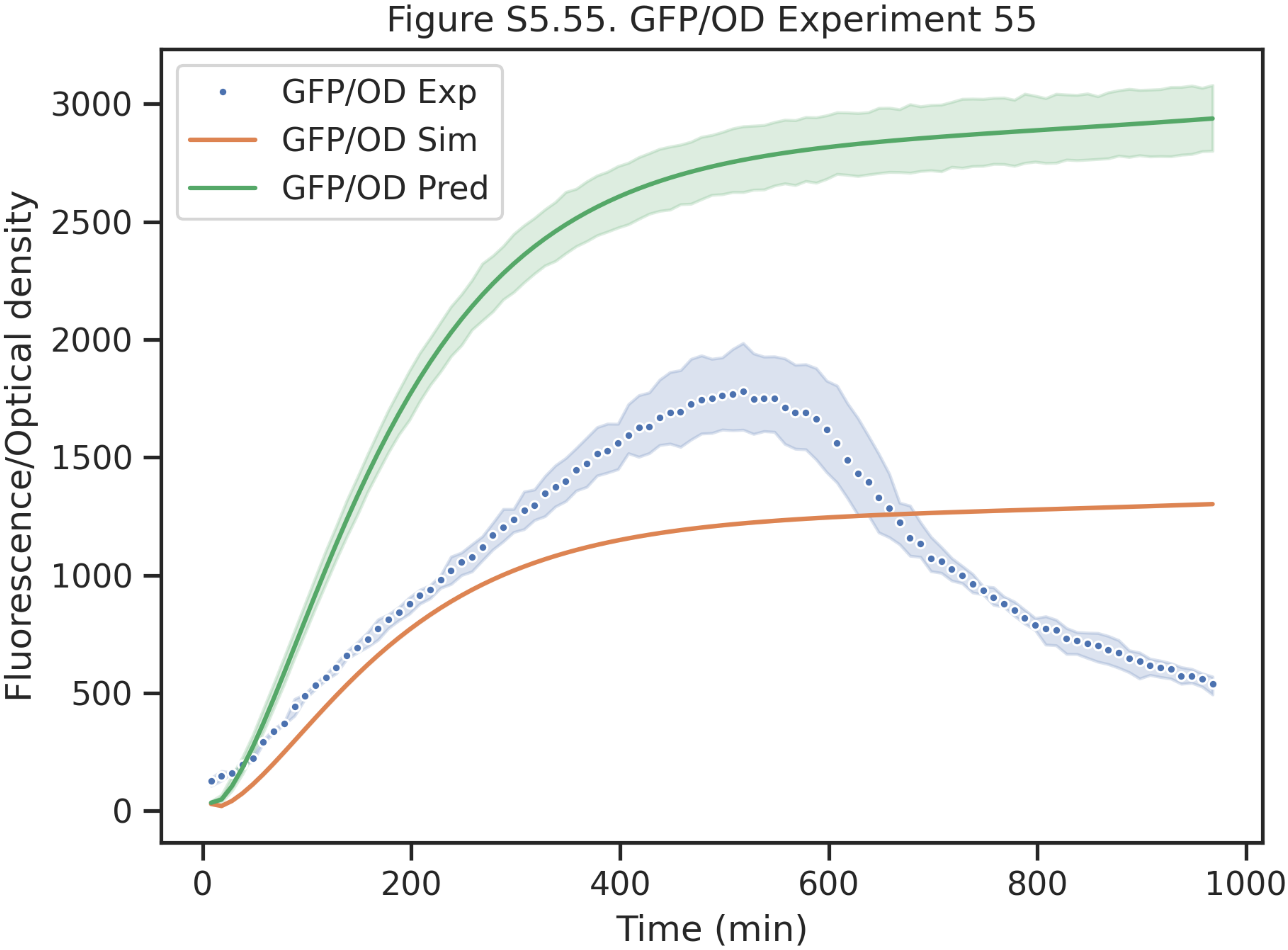
Figure S5.55. GFP/OD Experiment 55
GFP/OD Exp
GFP/OD Sim
GFP/OD Pred
Fluorescence/Optical density
Time (min)
0
500
1000
1500
2000
2500
3000
0
200
400
600
800
1000

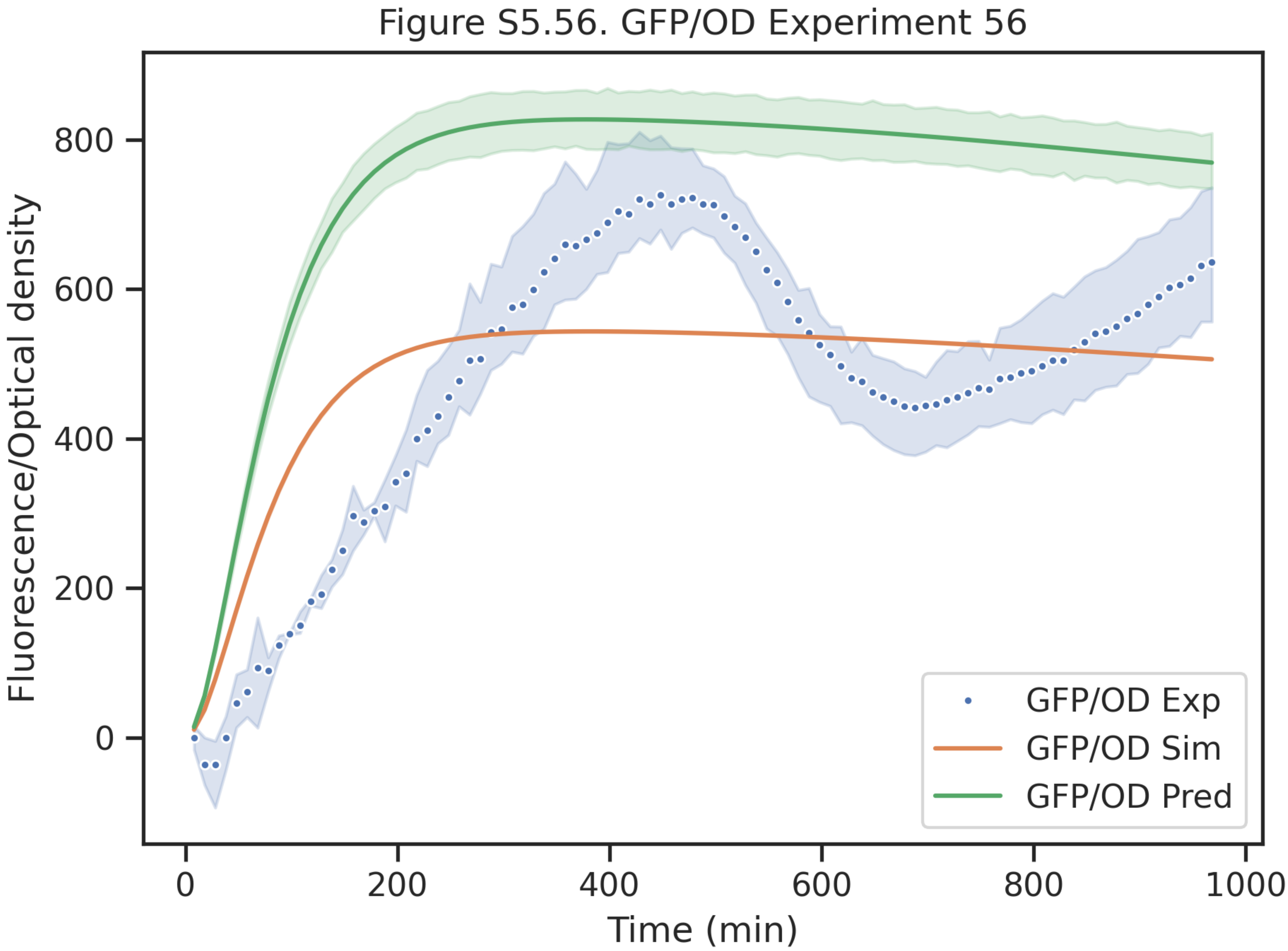
Figure S5.56. GFP/OD Experiment 56
Fluorescence/Optical density
Time (min)
0
200
400
600
800
1000
GFP/OD Exp
GFP/OD Sim
GFP/OD Pred

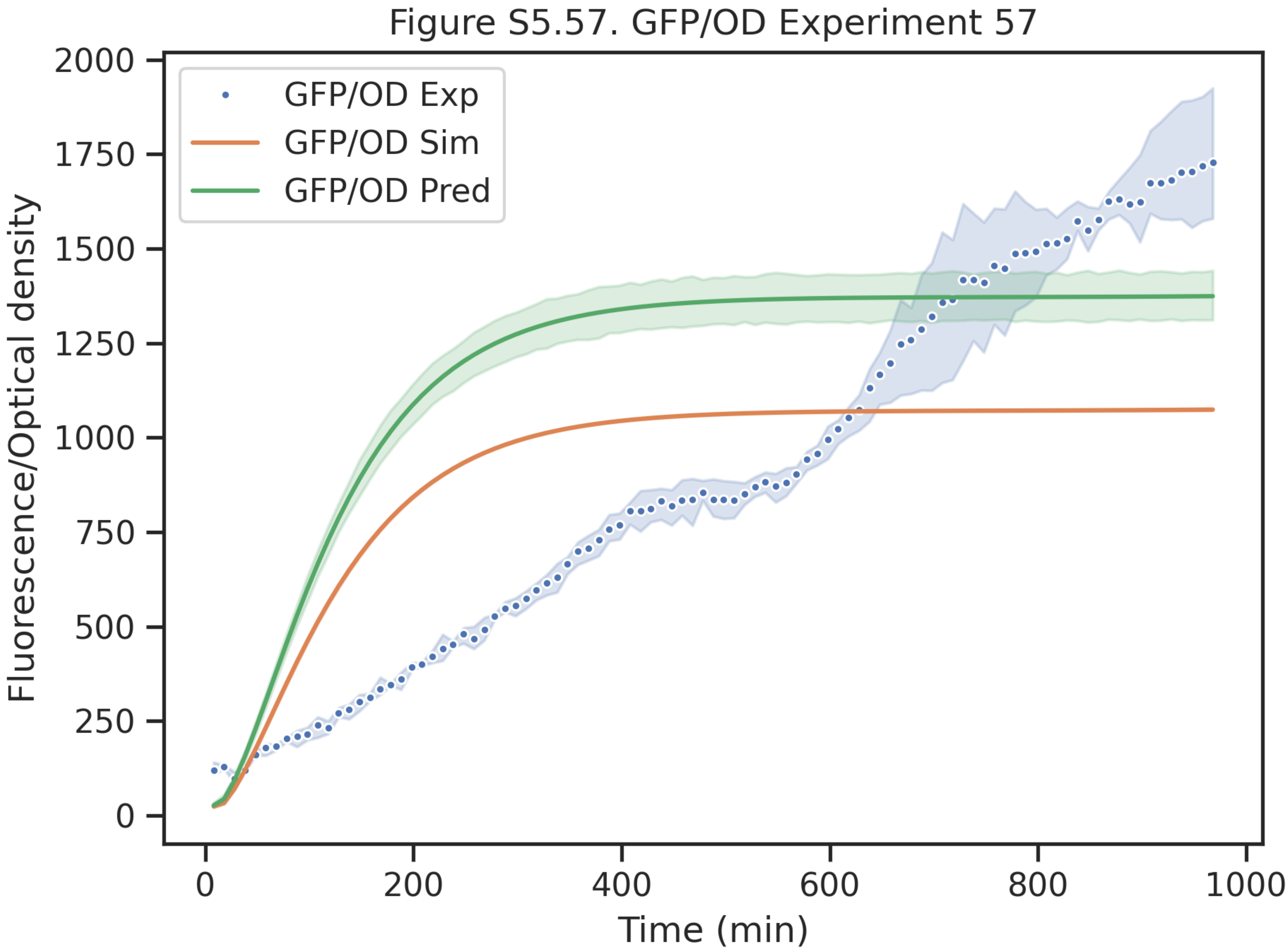

Figure S5.57. GFP/OD Experiment 57
GFP/OD Exp
GFP/OD Sim
GFP/OD Pred
Fluorescence/Optical density
Time (min)
0
250
500
750
1000
1250
1500
1750
2000
0
200
400
600
800
1000

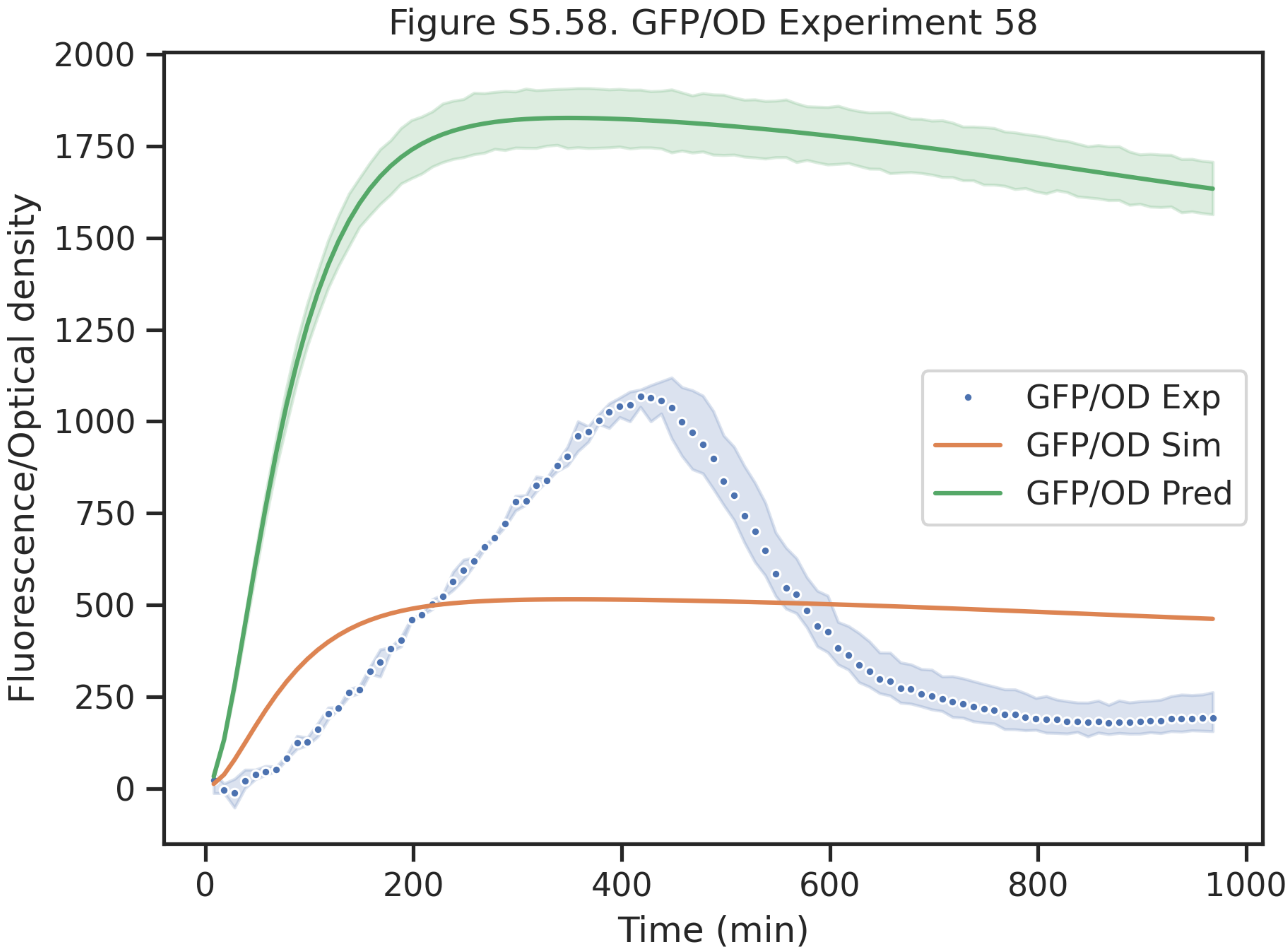

Figure S5.58. GFP/OD Experiment 58
Fluorescence/Optical density
Time (min)
2000
1750
1500
1250
1000
750
500
250
0
0
200
400
600
800
1000
GFP/OD Exp
GFP/OD Sim
GFP/OD Pred

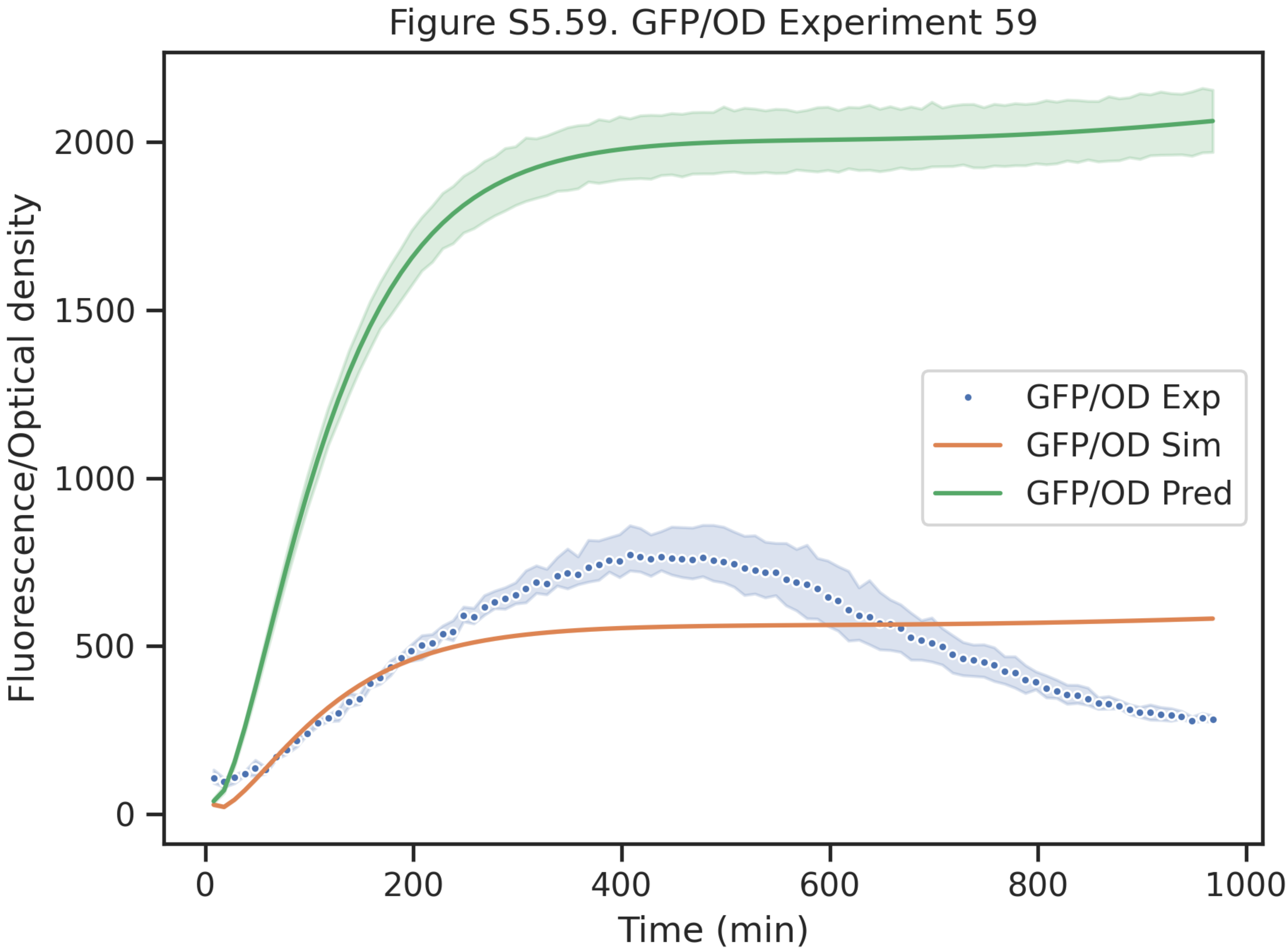

Figure S5.59. GFP/OD Experiment 59
Fluorescence/Optical density
Time (min)
0
500
1000
1500
2000
0
200
400
600
800
1000
GFP/OD Exp
GFP/OD Sim
GFP/OD Pred

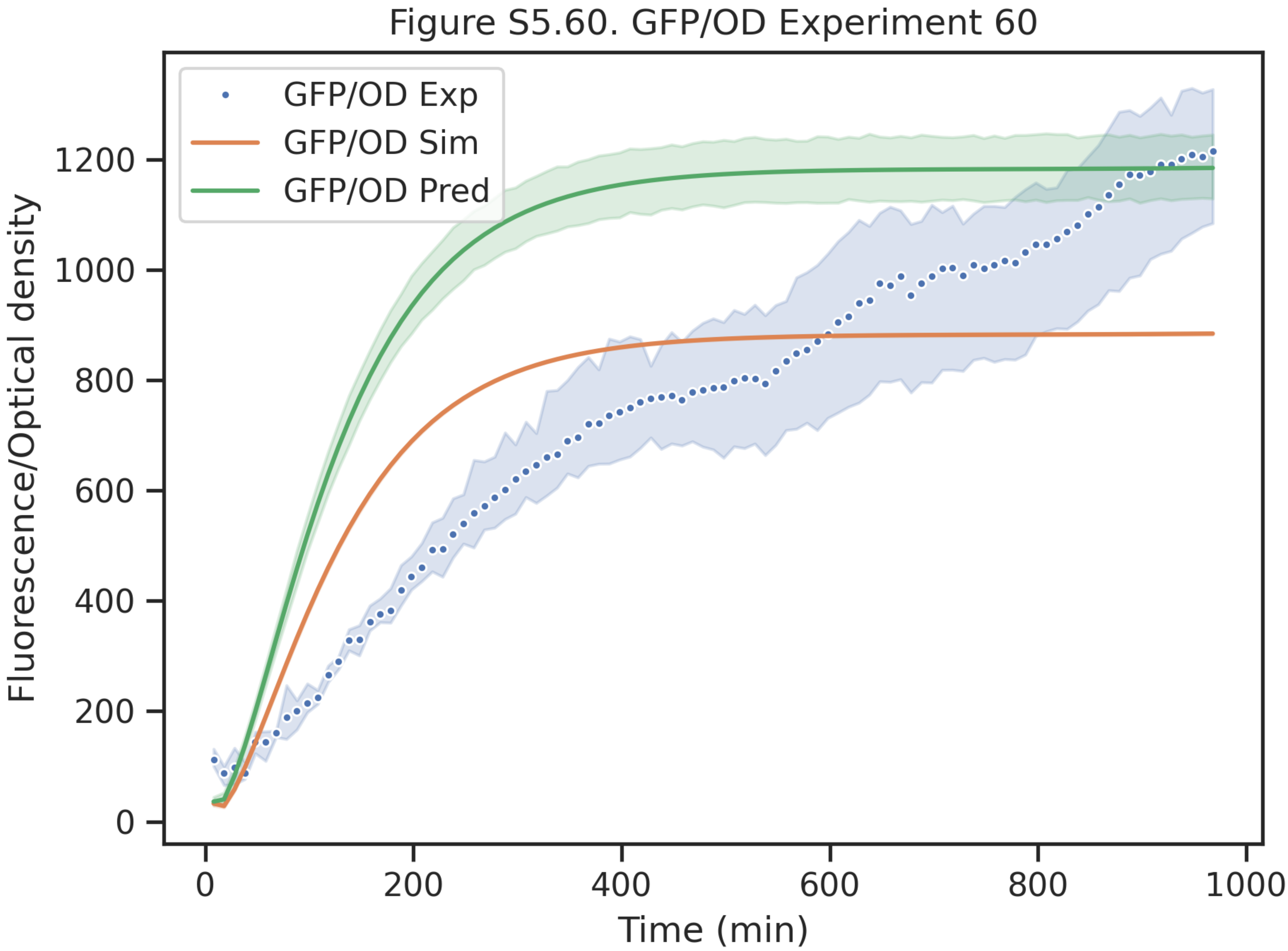
Figure S5.60. GFP/OD Experiment 60
GFP/OD Exp
GFP/OD Sim
GFP/OD Pred
Fluorescence/Optical density
Time (min)
0
200
400
600
800
1000
1200

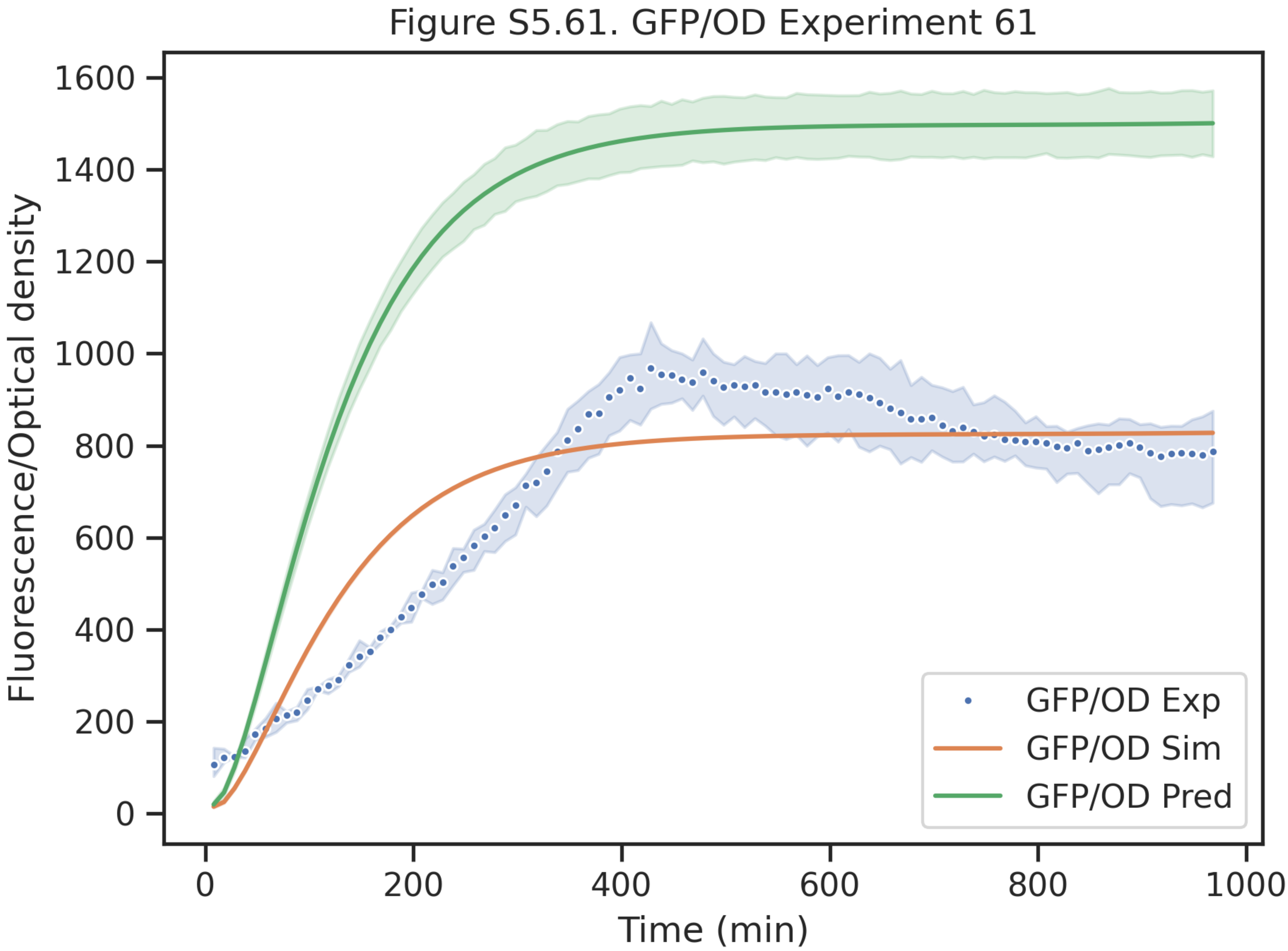
Figure S5.61. GFP/OD Experiment 61
Fluorescence/Optical density
Time (min)
0
200
400
600
800
1000
1200
1400
1600
0
200
400
600
800
1000
GFP/OD Exp
GFP/OD Sim
GFP/OD Pred

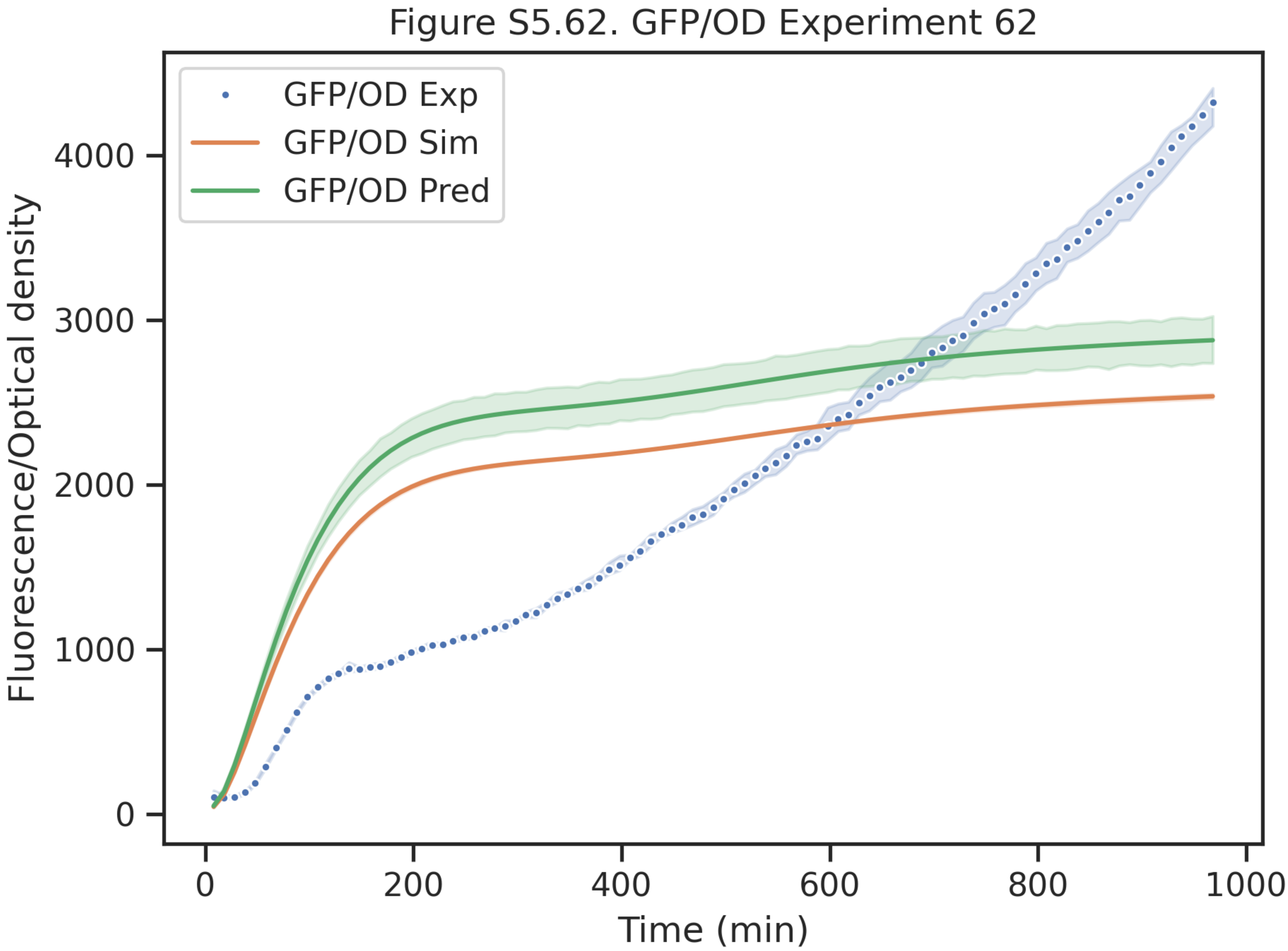
Figure S5.62. GFP/OD Experiment 62
GFP/OD Exp
GFP/OD Sim
GFP/OD Pred
Fluorescence/Optical density
0
1000
2000
3000
4000
0
200
400
600
800
1000
Time (min)

Figure S5.63. GFP/OD Experiment 63

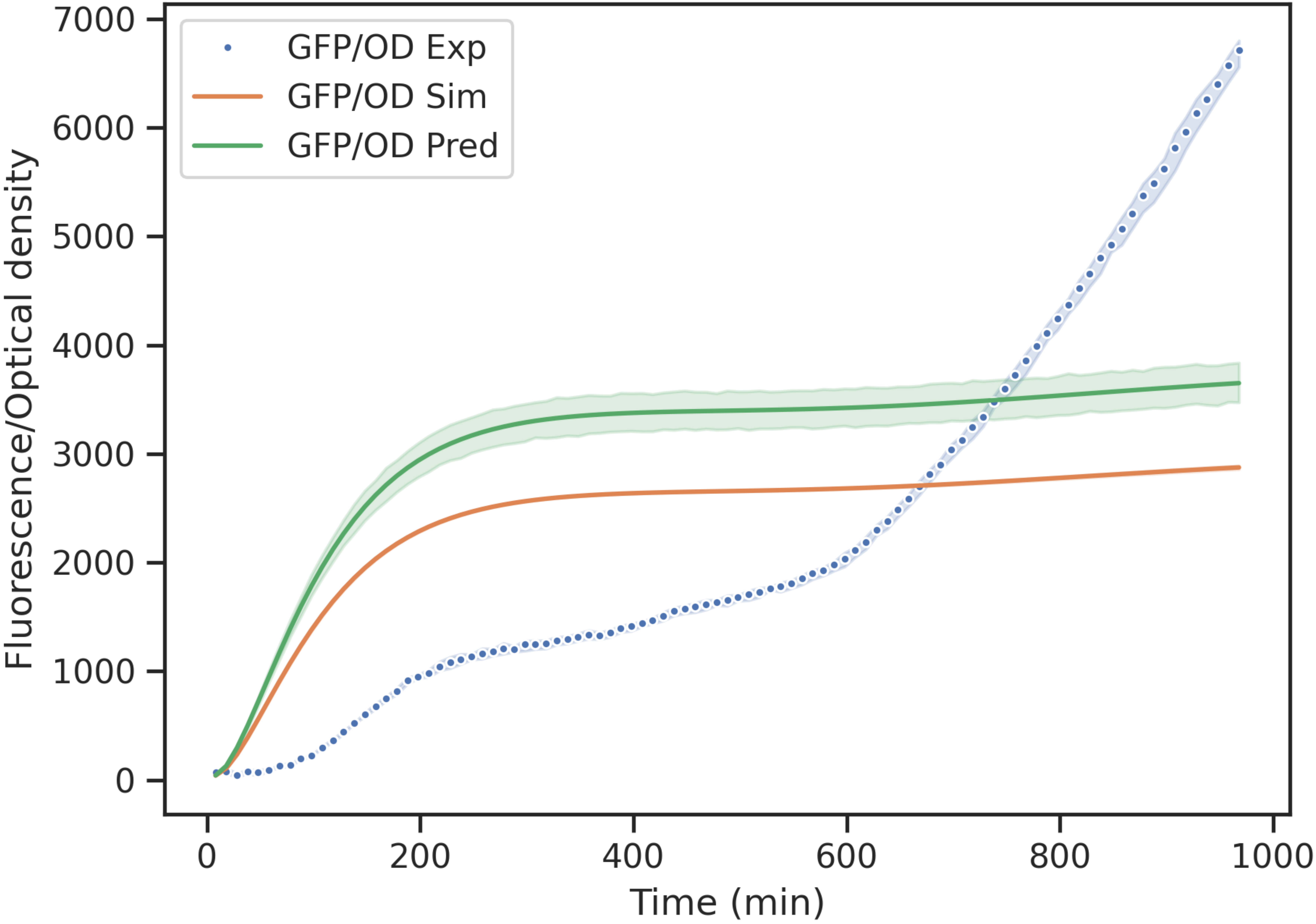

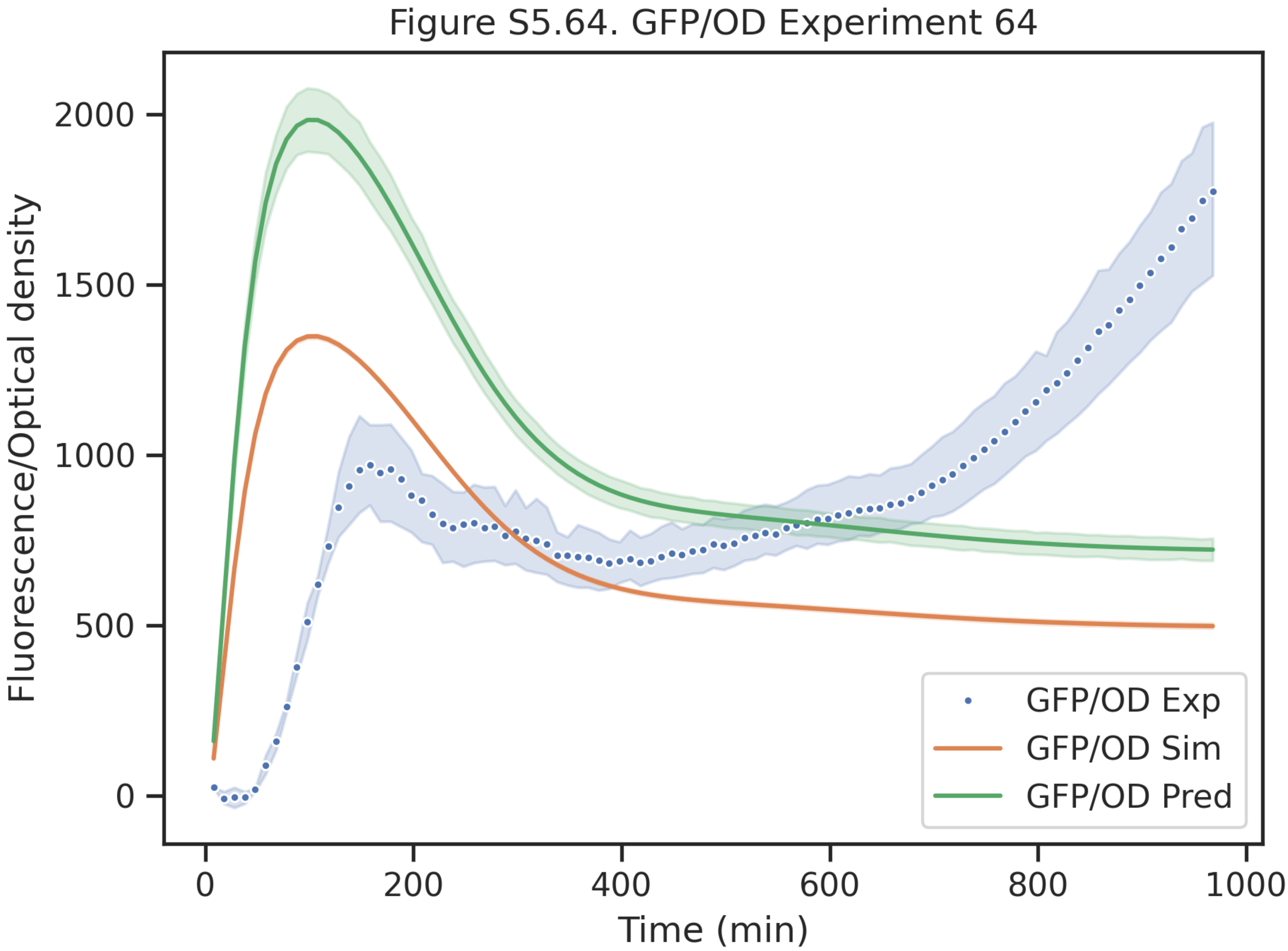
Figure S5.64. GFP/OD Experiment 64
Fluorescence/Optical density
2000
1500
1000
500
0
0
200
400
600
800
1000
Time (min)
GFP/OD Exp
GFP/OD Sim
GFP/OD Pred
